# Epigenomic modifications induced by hatchery rearing persist in germ line cells of adult salmon after their oceanic migration

**DOI:** 10.1111/eva.13235

**Published:** 2021-05-04

**Authors:** Maeva Leitwein, Martin Laporte, Jeremy Le Luyer, Kayla Mohns, Eric Normandeau, Ruth Withler, Louis Bernatchez

**Affiliations:** ^1^ Institut de Biologie Intégrative et des Systèmes (IBIS) Université Laval Québec QC Canada; ^2^ Ifremer IRD Institut Louis‐Malardé Univ Polynésie Française, EIO Tahiti France; ^3^ Department of Fisheries and Oceans Canada Pacific Biological Station Nanaimo BC Canada

**Keywords:** conservation, developmental plasticity, epigenomic, fisheries, fitness, hatchery, salmonid

## Abstract

Human activities induce direct or indirect selection pressure on natural population and may ultimately affect population's integrity. While numerous conservation programs aimed to minimize human‐induced genomic variation, human‐induced environmental variation may generate epigenomic variation potentially affecting fitness through phenotypic modifications. Major questions remain pertaining to how much epigenomic variation arises from environmental heterogeneity, whether this variation can persist throughout life, and whether it can be transmitted across generations. We performed whole genome bisulfite sequencing (WGBS) on the sperm of genetically indistinguishable hatchery and wild‐born migrating adults of Coho salmon (*Oncorhynchus kisutch*) from two geographically distant rivers at different epigenome scales. Our results showed that coupling WGBS with fine‐scale analyses (local and chromosomal) allows the detection of parallel early‐life hatchery‐induced epimarks that differentiate wild from hatchery‐reared salmon. Four chromosomes and 183 differentially methylated regions (DMRs) displayed a significant signal of methylation differentiation between hatchery and wild‐born Coho salmon. Moreover, those early‐life epimarks persisted in germ line cells despite about 1.5 year spent in the ocean following release from hatchery, opening the possibility for transgenerational inheritance. Our results strengthen the hypothesis that epigenomic modifications environmentally induced during early‐life development persist in germ cells of adults until reproduction, which could potentially impact their fitness.

## 1 INTRODUCTION

Understanding the evolutionary consequences of anthropogenic pressures on species biodiversity is needed to establish suitable conservation programs (Allendorf, 2017; Allendorf & Hard, 2009; Allendorf et al., 2010; Benestan et al., 2016; Frankham, 2008; Hendry et al., 2008; Palumbi, 2001). Human activities may induce direct selection pressures on natural populations or indirect selection pressures by altering the species environment (Hendry et al., 2008; Laporte et al., 2016). Modifications in species’ environment can also cause epigenomic modifications (Angers et al., 2010; Faulk & Dolinoy, 2011; Metzger & Schulte, 2017) including DNA methylation, histone modifications, small RNA sequences, and nucleosome positioning (Laubach et al., 2018; Lowdon et al., 2016; Richards et al., 2017). Such epigenomic modifications may in turn affect gene expression, especially during embryogenesis (Faulk & Dolinoy, 2011), and generate lifelong phenotypic variation, as reported for a variety of organisms (Verhoeven et al., 2016). Consequently, such human‐induced environmental change may modulate the individual phenotypes and consequently affect both the effect of natural selection and fitness (Angers et al., 2010; Aubin‐Horth & Renn, 2009; Laubach et al., 2018; Pfennig et al., 2010; Scoville & Pfrender, 2010). Major questions remain about how much epigenomic variation arises from environmental heterogeneity, whether this variation can persist throughout life, and whether such variation is reset between generations or, alternately, can be transmitted from one generation to the next (Danchin et al., 2019; Vineis et al., 2017). Inheritance of environmentally induced epigenomic variation, the part that is not erased during gametogenesis or embryonic development, represents a molecular mechanism of potential evolutionary significance in natural populations (Aller et al., 2018; Jablonka & Raz, 2009; Lind & Spagopoulou, 2018). Indeed, the persistence of epigenomic variation acquired during early‐life development up to adulthood and its transmission via germ line cells may have either adaptive and maladaptive effects in subsequent generations (Aller et al., 2018; Lind & Spagopoulou, 2018; Verhoeven et al., 2016; Vineis et al., 2017). Elucidating the differential effects of experiencing distinct human‐induced environments during development on the epigenome and the persistence of this alteration throughout life and meiotic cell line differentiation represents a major step toward understanding the evolutionary role of epigenetic variation (Angers et al., 2010; Richards et al., 2017; Verhoeven et al., 2016).

Anadromous salmon and trout of the genus *Oncorhynchus* are species of great socio‐economic interest on the Pacific Coast of North America that have been heavily impacted by human activities, through supplemented by extensive hatchery production in numerous river systems (Flagg & Nash, 1999). These species represent excellent systems for assessing the effects of epigenomic variation and its persistence through time for animals experiencing very different juvenile and adult environments. Indeed, juveniles are born and rear in freshwater until undertaking a marine feeding migration that lasts one to three years depending on species prior to return to freshwater for reproduction (Alerstam et al., 2003). Moreover, hatchery‐born salmon often display maladaptive traits in the natural environment, resulting in reduced survival and reproductive success despite weak genetic differentiation between hatchery and wild‐born fish allowing studying the effect of epigenomic variation without strong underlying genomic effects. (Araki & Schmid, 2010; Chittenden et al., 2008, 2010; Christie et al., 2014, 2016; Evans et al., 2014; Irvine et al., 2013; Neff et al., 2015; Zimmerman et al., 2015). For Coho salmon (*Oncorhynchus kisutch*), a lower reproductive success for hatchery‐born individuals spawning in the wild has been documented (Neff et al., 2015). Furthermore, a recent study documented the absence of genome‐wide genetic differentiation between wild and hatchery‐produced juvenile Coho salmon within a given river systems, but parallel epigenetic differentiation involving the same differentially methylated regions (DMRs) in muscle tissue between hatchery and wild Coho from two geographically distinct watersheds (Le Luyer et al., 2017). These observations support the hypothesis of an epigenomic basis for developmental plasticity that could affect the fitness of hatchery‐born salmon. Similar early life, environmentally induced epigenetic modifications have also been observed in the sperm of adult Steelhead trout (*Oncorhynchus mykiss*; Gavery et al., 2018) and Atlantic salmon (*Salmo salar*; Rodriguez Barreto et al., 2019), suggesting the persistence of epigenetic modification induced during hatchery rearing until adulthood after a long period of time spent in the open ocean, with potential for transgenerational inheritance. A follow‐up study on Steelhead based on simulated wild and hatchery environments did not reveal significant differences in patterns of sperm methylation due to the rearing treatment, therefore suggesting limited potential for intergenerational transmission (Gavery et al., 2019). Therefore, there is still disagreement in the literature which raised unresolved questions pertaining to the temporal persistence of hatchery‐induced epigenetic reprogramming. All of these previous studies utilized reduced representation (RRBS) methods, which provide only a limited subsample of the epigenome.

For comparative purposes with previous studies, and also because it is the most widespread mechanism of epigenetic modifications (Angers et al., 2020), we focused on the analysis of DNA methylation. We also analyzed more than one river system which allowed testing for parallelism of similar epigenomic modifications. Then, we assessed epigenomic variation over the entire epigenome by means of Whole Genome Bisulfite Sequencing (WGBS) with the goal of assessing whether (i) environmentally induced epigenomic modifications in early development persist throughout life after spending at least one year in the open ocean and (ii) the modifications are transmitted through germ lines cells, and thus potentially heritable. To do so, we compared methylation profiles in sperm from wild and hatchery‐born adult Coho salmon from two geographically remote rivers at three levels: epigenomic, epichromosomal, and local differentially methylated regions (DMRs). Analyzing fish of the same age from two hatchery river systems allowed circumventing interpretation issues caused by variation in relatedness or other factors that may occur within population. Finally, hatchery‐induced DNA methylation persisted in germ cells which could provide a mechanism for at least partial transgenerational inheritance of epigenetic modifications caused by hatchery rearing.

## 2 METHODS

### 2.1 Sampling

Coho salmon milt (sperm) samples were collected in Quinsam and Conuma hatcheries, British Columbia, Canada. Both hatcheries are part of the Salmon Enhancement Progam (SEP) and are separated by approximately 100 km (located on watersheds on the northeast (Quinsam) and northwest (Conuma) sides of Vancouver Island). The hatcheries operate with a primary production strategy (PPS) [(see Le Luyer et al. (2017) for more details)]. Briefly, the aim of such “integrated” hatchery programs is to use all local returning fish, both wild and hatchery, as broodstock to minimize genetic differentiation between the hatchery and wild spawning environments. In this study, returning adult males were sampled before spawning at Quinsam and Conuma hatcheries, on November 1 and 2, 2017, respectively. At both sites, hatchery‐born and wild fish were distinguished by the presence (wild) or absence (hatchery) of their adipose fins, as hatchery‐produced fish have their adipose fin clipped off before release. Milt was collected from 24 mature male Coho salmon, including 12 individuals from each of Quinsam hatchery (seven wild and five hatchery males) and Conuma hatchery (six wild and six hatchery males) that had spent 6 or 18 months in the sea before return to their river of origin for spawning. Sampled fish had swum voluntarily into a hatchery concrete holding pond with flowing water, been held for approximately two weeks, and were transferred to a large tank with flowing water on the day of sampling (Nov. 1–2, 2017). Fish were euthanized and sampled for several tissues, including milt. All samples were stored in the fridge (4°C) for 24–48 h and then transferred into a −80°C freezer.

### 2.2 DNA extraction and whole genome bisulfite sequencing

Genomic DNA was extracted following a protocol of universal and rapid salt‐extraction (Aljanabi & Martinez, 1997). DNA quality control, library preparation, and 100 bp paired‐end sequencing on an Illumina HiSeqX (two individuals per lane) were performed at the McGill University and Génome Québec Innovation Centre (Montréal, QC).

### 2.3 Methylation calling

The WGBS reads were trimmed and quality filtered (min quality = 25, min length = 100 bp) with fastp (https://github.com/OpenGene/fastp [Chen et al., 2018]). In order to avoid confusing false epigenetic variation with existing C‐T polymorphisms, we masked the reference genome (NCBI assembly GCA_002021735.1; Okis_V1) from C‐T polymorphism identified with whole genome resequencing of 20 Coho salmon (940,406 SNPs, maf = 0.05) from four British Columbia rivers, using BEDtools *maskfasta* v2.26.0 (Quinlan & Hall, 2010) as in Le Luyer et al. (2017). WGBS trimmed reads were mapped against the masked Coho genome with WALT v1.0 (4; https://github.com/smithlabcode/walt [Chen et al., 2016]) by using default parameters and a maximum allowed mapping for a read (−k) of 10. The symmetric CpG methylation levels of individuals were estimated with MethPipe v.3.4.3 (https://github.com/smithlabcode/methpipe). All symmetric CpG sites with <10× coverage were removed. The relationship between the number of symmetric CpG sites and the linkage group length was assessed by performing linear mixed models in the R package “LME4” (Bates et al., 2015: 4). The number of symmetric CpG sites was treated as a dependent variable whereas the linkage group length was designated an explanatory term and individuals were treated as random effects in the model. The explanatory and dependent variables were scaled (i.e., center and reduced). We computed the conditional *R* ^2^ to quantify the proportions of variance explained by the explanatory variable and individual effects.

### 2.4 Genotyping for genetic data

For genomic analysis, SNPs were called with *Freebayes* v1.3.2‐38‐g71a3e1c (Garrison & Marth, 2012) from the WGBS mapped reads considering reads with good alignment with a minimum mapping quality of 20 and a minimum coverage of 10. We then used *VCFtools* (Danecek et al., 2011) to kept biallelic markers with a minimum and maximum depth of coverage between 5× and 100×, a minimum allele frequency of 0.01, a minimum quality of 20, and a maximum of 20% of missing data. Then all C/T and A/G polymorphism were removed to avoid false positives due to the bisulfite treatment. We used the function “*daisy*” available in the ape R package (Paradis et al., 2004) to calculate an Euclidian distance matrix. Then, the function “*pcoa*” in the ape R package (Paradis et al., 2004) was used to perform a principal coordinates analysis (PCoA) based on the distance‐based matrix; to be consistent and comparable to the previous study (Le Luyer et al., 2017) only the axes representing at least 3% of the variation were retained for the distance‐based redundancy analysis (db‐RDA). Then, the 22 retained axes were used as response matrix with the rearing environment (hatchery vs. wild) and river of origin (Quinsam vs. Conuma) variables used as explanatory matrix in the db‐RDA, using the function “*ordistep”* in the vegan R package (Oksanen et al., 2019). The genetic differentiation (*F*_ST_) between individuals from both rivers and rearing environment (wild vs. hatchery) was estimated with *VCFtools* (Danecek et al., 2011) based on Weir and Cockerham's calculation. *T*‐test was performed in order to test if genetic differentiation were significantly different between rivers and rearing environment.

### 2.5 Environmental effects on individual methylation profile

For each individual, a genome‐wide mean methylation was estimated over 1 kb windows (step = 1 kb, size = 1 kb), and only 1 kb regions with at least 3 symmetric CpGs were kept (Le Luyer et al., 2017). The windows approach was performed in order to reduce computing time. We then calculated an Euclidian distance matrix with the function “*daisy*” available in the ape R package (Paradis et al., 2004). Principal coordinates analysis (PCoA) was performed on the distance‐based matrix with the function “*pcoa”* in the ape R package (Paradis et al., 2004); only the axes representing at least 3% of the variation were retained for the distance‐based redundancy analysis (db‐RDA). Then, the 19 retained axes were used as response matrix with the rearing environment (hatchery vs. wild) and river of origin (Quinsam vs. Conuma) variables used as explanatory matrix in the db‐RDA, using the function “*ordistep*” in the vegan R package (Oksanen et al., 2019). The effect of each significant variable (river and rearing environment) was examined in partial db‐RDA for controlling the shared effect between variables (river and rearing environment).

To assess fine‐scale information in the genome, we looked at local differentiation within a chromosome by performing separate analyses for each chromosome. Thus, db‐RDA was performed for each chromosome after the PCoA and the distance‐based matrix transformation. Partial db‐RDAs were then performed to test for the effect of each significant variable (river and rearing environment) controlled by the other variable. The correlation between the *R* ^2^ of each model and linkage group size was tested using Spearman test in R (Team 2014).

### 2.6 Detection of differentially methylated regions

The identification of differentially methylated regions (DMRs) between hatchery‐born and wild individuals was based on the dispersion shrinkage method implemented in the package DSS (Dispersion Shrinkage for Sequencing data (Park & Wu, 2016). We used a multifactor generalized linear model in which the symmetric CpG methylation level was treated as the dependent variable and the environmental variables (rearing environment and river) as explanatory terms, with their interaction also included in the model (methylation data ~ rearing environment + river + rearing environment:river). In order to call DMRs, we set a minimum sequenced length of 100 bp containing at least 10 CpGs sites, a *p*‐value threshold (*p*.*threshold*) of 0.01 in order to keep DMR with strong signals (Rougeux et al., 2019), a maximum distance of 50 bp to merge two DMR (*dis*.*merge*) and a threshold of 0.4 for percentage of CG sites with significant p‐value (*pct*.*sig*). The same approach was used to call DMRs between rivers (Conuma and Quinsam). To control for false‐positive discovery, we performed 100 random assignations of individuals to the hatchery or wild‐born groups within each river (it should be noted here that some individuals might be assigned to their true groups during random assignation) and then call parallel DMRs between hatchery and wild‐born individuals. Figure S3 displayed the number of DMRs found at each run with a mean of 107.6 [IC 95%: 65.95–183.025] and indicates a *p* < 0.01 to find 212 DMRs or more in our dataset, which supports the observation that our DMRs are unlikely to be the results of false discovery rate. Finally, with the goal of removing potential false discovery DMRs, we kept only DMRs with a mean methylation differentiation between wild and hatchery higher than 10%. The correlations between the number and length of DMRs and the chromosome size were tested using Spearman test in R (Team, 2014). The mean read depth and mean percentage of methylation per rearing environment were plot for each CpG within and around (200 bp for graphic representation) of each DMR with R (Team, 2014). In order to visualize individual variation, individual methylation was also plotted for each DMRs and its surrounding region. Partial redundancy analyses (pRDA) were performed in order to quantify the proportion of CpGs variation within DMRs explained either by the rearing environment or the genetic variation among individuals, using the function “*rda*” and “*varpart*” available in the vegan R package (Paradis et al., 2004). Genetic variation was estimated from 7151 SNPs obtained from our dataset after filtering for CT and GA polymorphism and removing SNPs with more than three individuals with missing data. Principal coordinates analyses on Euclidean distance from CpGs variation of both DMRs types (Rearing environment and River of origin) and 0–1–2 SNPs matrix were produced to estimate their relative proportion of variation using the function “*pcoa*” and “*dist*” in the ape R package (Paradis et al., 2004). A broken‐stick distribution was used to select the number of axes to keep for DMRs‐CpGs variation, while 14 axes representing 90% of the genetic variation were retained.

### 2.7 Annotation

We annotated the Coho salmon genome (*Oncorhynchus kisutch*, https://www.ncbi.nlm.nih.gov/genome/?term=txid8019[orgn]) with the published transcripts accompanying this genome using GAWN v0.3.3 (https://github.com/enormandeau/gawn) in order to find transcripts that were close to DMRs. GAWN used GMAP version 2018‐07‐04 to align the transcripts to the genome. For DMRs associated with the rearing environments, we identified the overlapping transcripts by using BEDtools intersect v2.26.0 (26). We also performed the analyses again after adding 5 kbp around each DMR to include potential genes associated with, but not directly overlapping, the DMRs. We then used Go_enrichment (https://github.com/enormandeau/go_enrichment) to annotate the same transcriptome by blasting the transcripts on the swissprot database (blast tools 2.7.1, swissprot from 2018‐05‐01). We proceeded to test for the presence of over‐ and under‐represented GO terms using GOAtools (v0.6.1, ‐‐*p* val = .05). We filtered the outputs of GOAtools to keep only GO terms at a level of 3 or less and an FDR value ≤0.1.

## 3 RESULTS

Whole Genome Bisulfite Sequencing (WGBS) performed on 24 adult male Coho salmon sperm samples produced a mean of 189 ± 20 million of reads per individual of which 92 ± 11 million of reads per individual (49%) were successfully mapped to the reference genome masked for cytosine to thymine (C‐T) polymorphism. A mean of 11 ± 2.8 million of symmetric cytosine‐phosphate‐guanine context (CpGs) per individual with at least 10X coverage was retrieved, with a mean of 0.3 ± 0.1 million of symmetric CpG per chromosome. The number of symmetric CpG was strongly correlated with chromosome length (*R* ^2^ conditional = 0.95, Figure S1).

### 3.1 No evidence for genome‐wide genetic differentiation

No significant genome‐wide genetic differentiation was observed between wild and hatchery‐born individuals within each river. Thus, after filtering for CT/AG polymorphism and quality, we obtained a total of 23,442 SNPs on which we performed a distance‐based redundancy analysis (db‐RDA) on Euclidean distance to test for the effect of river and rearing environment on genetic variance partitioning. The genetic variation was significantly explained by the river of origin (*p* < 0.001) with an adjusted *R* ^2^ of 0.025, but not by rearing environment (*p* = 0.23). Moreover, the extent of genetic differentiation (*F*_ST_) was ten times greater between Coho from Conuma and Quinsam rivers (*F*_ST_ = 0.013) than between wild and hatchery individuals (*F*_ST_ < 0.001; *t*‐test *p* < 0.001). *F*_ST_ < 0.001 indicates that hatchery and wild Coho within a same river are genetically indistinguishable, as previously reported (Le Luyer et al., 2017).

### 3.2 Rearing environment effect on epigenomic variation

We assessed the mean methylation level over non‐overlapping windows of 1 kb along the genome. The db‐RDA performed at a global scale (whole genome) to evaluate overall pattern of methylation variation among adult males using rearing environment and river of origin as explanatory variables was significant (*p* < 0.002) with an adj. *R* ^2^ of 0.02 (Figure S2). However, partial db‐RDA confirmed that genome‐wide epigenetic variation was explained more by the river of origin [adj. *R* ^2^ = 0.013, *p* = 0.01] than rearing environment [adj. *R* ^2^ = 0.008, *p* = 0.07] (Figure S2).

**FIGURE 1:**
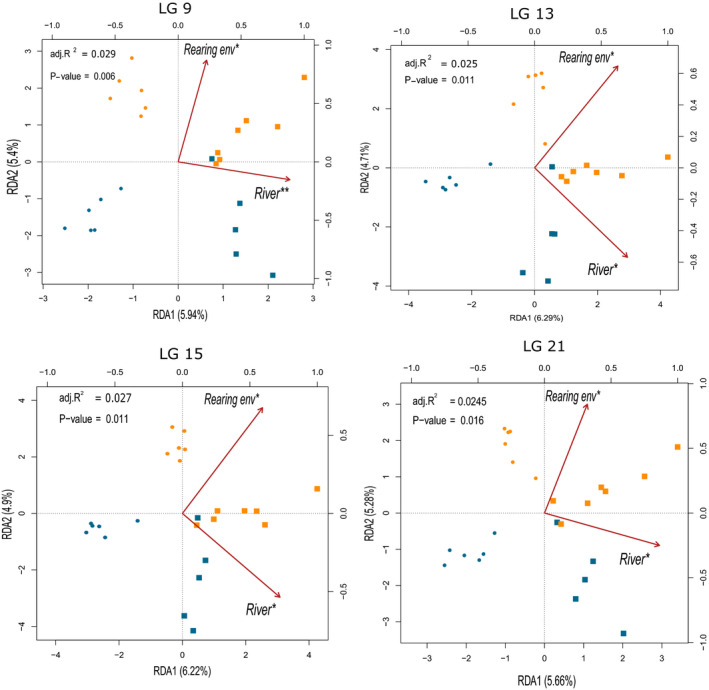
Local distance‐based analysis (db‐RDA) performed with average methylation level through 1 kb window‐size on Coho salmon milt samples for chromosomes 9, 13, 15, and 21. Symbols represent the river: circles for Conuma and squares for Quinsam. Colors represent rearing environment: blue for hatchery‐born and orange for wild individuals. All db‐RDA were significant and explained 1.1%–2.9% of the global DNA methylation variation (adj. *R* ^2^ is displayed in upper left of each graph). Partial db‐RDA revealed that river of origin and rearing environment explained, respectively, 1.4%–1.7% and 1.1%–1.2% of the variation after controlling for the other variable

The same analytical approach was applied at the chromosomal scale, whereby db‐RDA was performed for each of the 30 chromosomes independently. This analysis showed that similar epigenetic modifications induced by hatchery‐rearing environment persisted on specific chromosomes and in parallel in the two river systems until adulthood after more than one year of growth spent in the open ocean. Thus, components of epigenetic alteration caused by hatchery rearing that have persisted in parallel among adult males from both rivers of origin were not randomly distributed but significantly concentrated on specific chromosomes. Models were significant (*p* < 0.05) for 28 chromosomes with an adj. *R* ^2^ ranging from 0.009 to 0.029 (Table S1 for details). Rearing environment significantly explained epigenetic variance observed on four chromosomes presented in Figure 1 (*p* < 0.05; Table S1). Rearing environment significantly explained the epigenomic variation for LG 9, 13, 15, and 21 with an adj. *R* ^2^ of 0.012 (*p* = 0.03), 0.012 (*p* = 0.04), 0.012 (*p* = 0.04), and 0.011 (*p* = 0.05), respectively (Table S1). Moreover, there was no significant relationship between the adj. *R* ^2^ and chromosome size (*ρ*_Spearman_ = −0.28; *p* = 0.123), indicating that the extent of persisting epigenetic differentiation between hatchery and wild adult males is not directly affected by the number of CpG sites occurring on a given chromosome.

### 3.3 Comparison of DMRs induced by rearing environment between life stages

**FIGURE 2:**
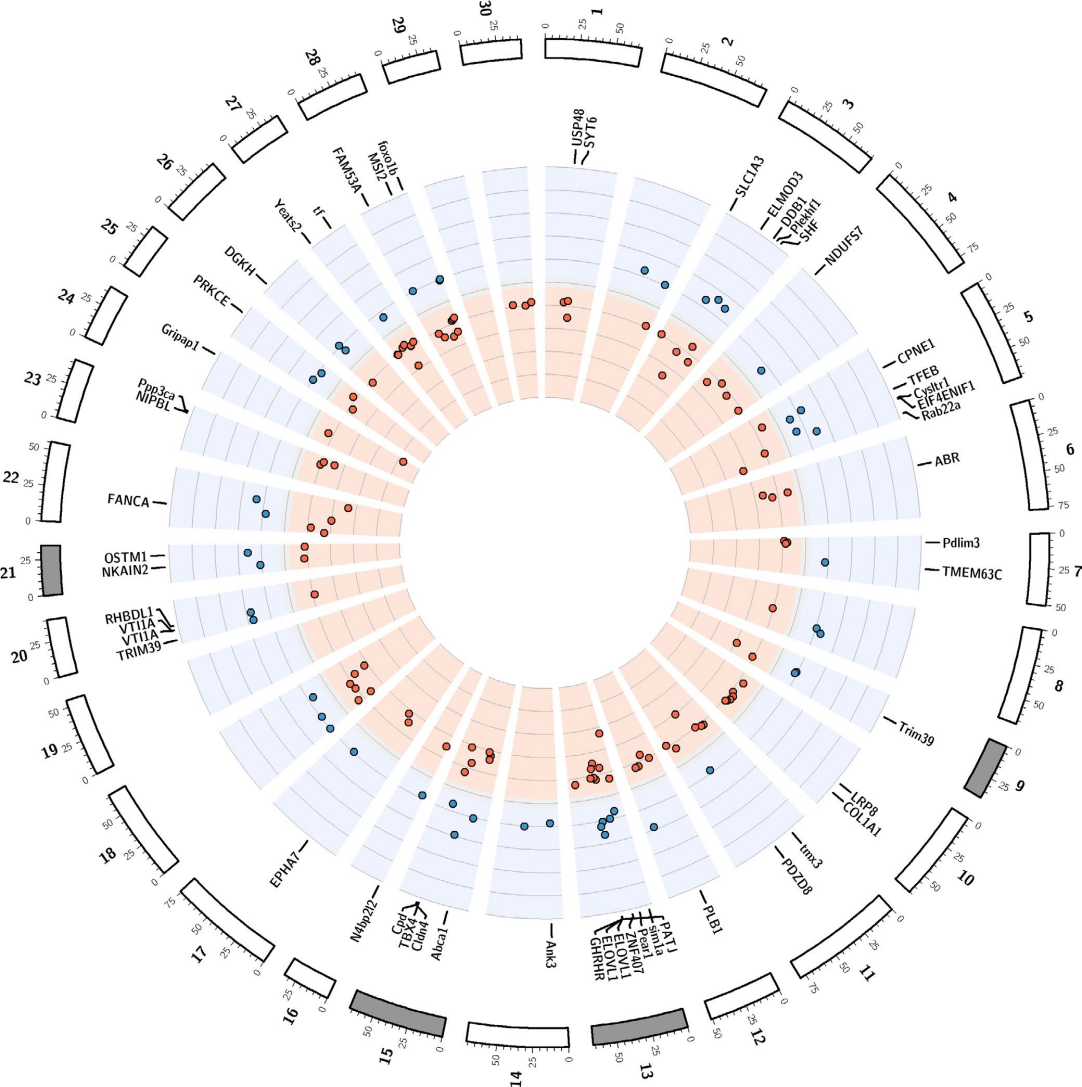
Circle plot showing the 183 differentially methylated regions (DMRs) between hatchery and wild adult fish in milt tissue Only the chromosomes (*n* = 30) containing DMRs are represented. Red points indicate hypermethylated and blue points hypomethylated DMRs for the hatchery fish. The overlapping transcripts are represented and details can be found in Table S4. Chromosomes for which db‐RDA were significant are presented in gray

**FIGURE 3:**
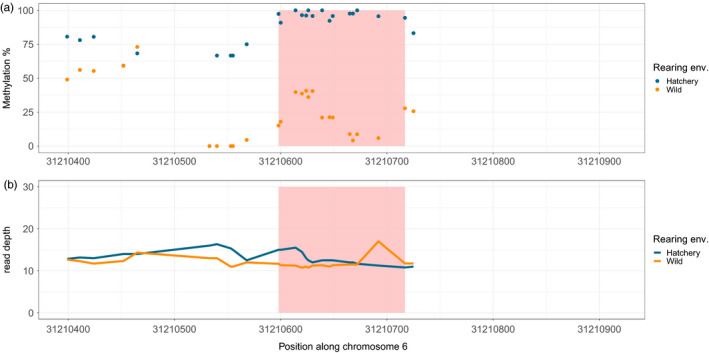
Mean percentage of methylation (a) and read depth (b) for hatchery (blue) and wild‐born (yellow) individuals for each CpGs among and around (200 bp) DMRs 61 (pink area) on chromosome 6

A total of 212 DMRs (min. length 100, minCG 10, *p*_threshold_ = 0.01) were identified between wild and hatchery‐born adult males (Table S2), ranging from 105 to 1429 bp in size and distributed among 29 chromosomes. Randomizations have been performed to fully control for false positive DMRs discovery, the probability to obtain 212 parallel DMRs was <0.01 (see Section 2 for details). There was no correlation between DMR length and chromosome size (*ρ*_Spearman_ = −0.007; *p* = 0.912) but the number of DMRs increased with chromosome size (*ρ*_Spearman_ = 0.36; *p* = 0.05). The mean difference between hatchery and wild‐born methylation ranged from 0.4% to 73% we kept a total of 183 DMRs with a mean methylation difference higher than 10% for subsequent analyses (Figure 2 and Table S2). All DMRs and the 200 bp region around them are presented in Appendix S1; as an example, DMR 61 on chromosome 6 displaying the higher differentiation between the mean methylation of hatchery and wild‐born individuals (73%) is presented in Figure 3. In order to highlight the individual variation, the individual methylation value per CpGs for each of the 183 DMRs, is presented in Appendix S2. As previously observed at the smolt (juvenile) stage, we found a significant over‐representation of hypermethylated DMRs (*n* = 116) relative to hypomethylated DMRs (*n* = 67) in the sperm of hatchery‐born adult males in both rivers (χ^2^ = 61.84, *p* < 0.001). Finally, we performed partial redundancy analyses where genetic variation was estimated from 7151 filtered SNPs to ensure that the rearing environment significantly explains the variation of observed DMRs between hatchery and wild‐born Coho, as opposed to the sole genetic effect. We observed that rearing environment explained 51.4% of DMRs CpGs variation after controlling by genetic variation, while genetic variation explained 44.1% of that variation.

In parallel, a total of 305 DMRs (min. length 100, minCG 10, *p*_threshold_ = 0.01) were identified between Conuma and Quinsam river ranging from 103 to 1562 bp in size and distributed among the 30 chromosomes (Table S3). However, 90.4% of the CpG variation within the 305 DMRs identified between rivers was explained by the genetic variation, whereas no variation was significantly explained by the rearing environment.

### 3.4 Functional annotation of DMRs

Among the 183 DMRs found between wild and hatchery adult males, 58 overlapped with unique transcripts. We observed a significant over‐representation of hypermethylated DMRs (*n* = 36) relatively to hypomethylated DMRs (*n* = 22) in the sperm of hatchery fish (χ^2^ = 19.19, *p *= 6.8e‐5; Table S4). The gene ontology (GO) analysis did not reveal significant enrichment of biological processes but three transcripts were associated with brain development, seven with cellular homeostasis, three with immune response, and eight with multicellular organism development and apoptosis processes.

## 4 DISCUSSION

Environmentally induced epigenomic modifications through human activities may generate adaptive or maladaptive plasticity (Angers et al., 2010; Aubin‐Horth & Renn, 2009; Laubach et al., 2018; Scoville & Pfrender, 2010; Vogt, 2017) and persistence of early‐life maladaptive epimarks until reproduction can affect individual fitness (Vineis et al., 2017). Moreover, post‐meiotic retention of the epigenetic modifications in gametes opens the possibility of transgenerational transmission with potential evolutionary consequences within populations (Donkin & Barrès, 2018; Kekäläinen et al., 2018; Rodriguez Barreto et al., 2019). Consequently, assessing the effects of early‐life‐induced epigenomic modifications is of prime importance to establish conservation management strategies, especially since hatchery‐produced salmonids commonly perform poorly compared to their wild congeners (Chittenden et al., 2008, 2010; Irvine et al., 2013; Zimmerman et al., 2015) even in absence of significant genetic differentiation between them (Le Luyer et al., 2017). Indeed, hatchery‐reared Coho salmon display lower swimming endurance, survival, and predator avoidance (Chittenden et al., 2010) and lower reproductive success (Neff et al., 2015) than their wild counterparts. Results of Le Luyer et al. (2017) previously identified DMRs associated with genes functionally relevant to swimming capacity and cognition in hatchery‐reared juveniles, which supported the hypothesis that epigenomic modifications induced early in life by the rearing environment may be partly responsible for the reduced performance, yet conclusions could not be transposed to returning adults. These findings established a possible role for environmentally induced epigenomic modifications affecting performance of juvenile Coho in nature. Here, performing WGBS on adult male Coho salmon returning from their oceanic migration, we provided evidence for parallel (shared) differentiation of DNA methylation in the sperm of hatchery and wild salmon from two independent river systems. These differences were not distributed randomly across the genome but were significantly associated with four specific chromosomes. Remarkably, epigenetic differentiation between hatchery‐reared and wild Coho occurred without significant genetic difference between them. This provides evidence that epigenome modification occurring during early development in a hatchery can persist after more than one year spent at sea until adulthood. As such, our results support the view that epigenomic modifications occurring during hatchery‐rearing represent a potential molecular mechanism explaining the previously documented reduced fitness of hatchery‐born adult Coho salmon (Christie et al., 2014; Neff et al., 2015). Moreover, the fact that hatchery‐wild epigenetic differentiation at specific chromosomes was maintained throughout meiotic divisions opens the possibility of transgenerational inheritance through male germ cells. This highlights the importance for hatchery management strategies to create environmental conditions as close as possible to wild conditions in order to reduce epigenomic differences between wild and hatchery‐born Coho to ultimately reduce fitness differences.

In Coho salmon, development of spermatogonia (i.e., differentiation of primordial germ cells into testicular tissue) occurs early in larval development, that is several weeks after hatching (Devlin & Nagahama, 2002). This strengthens the hypothesis that DNA methylation reprogramming induced as early as embryogenesis might provide a hatchery‐induced epigenetic memory that could persist until adulthood (Vineis et al., 2017). The finding of epigenomic modifications in sperm, after meiotic cell division, also indicates potential for transgenerational inheritance of the environmentally induced paternal methylome. Previous studies on early embryos in zebrafish (*Danio rerio*) showed that DNA methylation was inherited by the progeny from the sperm methylome in spite of a whole DNA reprogramming during embryogenesis (Jiang et al., 2013). More recently, the study Barreto et al. (2019) on Atlantic salmon (*S. salar*) revealed that a small number of methylation marks associated with captive‐rearing in sperm cells remain in the offspring beyond developmental reprogramming. Our study adds to these previous observations in controlled conditions by extending to an analysis of adult fish in the wild returning from their marine migration. Moreover, we also document that similar epigenetic modifications have been retained and transmitted to germ cells independently in different hatcheries. Taken together, if the epigenome of males is actually transmitted to the next generation, this could represent one way by which captive‐rearing may negatively impact the fitness of progeny. This is consequential for salmon conservation and the management of extensive hatchery production and supplementation of salmonids throughout the world as they strengthen the hypothesis that epigenomic modifications caused by environmental conditions during early‐life stage can be transmitted to the next generation and may partly account for the reduced fitness of hatchery‐produced fish in nature, even in absence of genetic differences between wild and hatchery fish (Christie et al., 2016). A possible link between the paternal rearing environment and progeny adaptation or maladaptation to its rearing environment is of fundamental evolutionary consequence for natural populations. This is also fundamental for conservation programs involving habitat alteration for breeding adults. In a situation where there is no evidence for genetic differences between hatchery‐reared and wild fish from the same river (as here, see also Christie et al. (Christie et al., 2016)), our results further support the possibility that the fitness of hatchery‐reared salmonids once released in the wild could be increased by adapting management strategies aiming at minimizing environmental differences between hatchery and wild rearing conditions. Indeed, it has been documented that maladaptive effect of captivity can emerge after a single generation spent in hatchery (Christie et al., 2016), highlighting the importance of early‐life rearing conditions. For example, reducing the density of fish in those captive environment could be a first step toward an improved hatchery‐rearing environment (i.e., closer to the natural environment; Thompson & Blouin, 2015) as well as replicating intricacies of natural breeding behavior (Thériault et al., 2011).

The fact that the variance in methylation at the genome scale explained by rearing environment and shared between populations from both rivers is only marginally significant is not unexpected given that salmon have 30 chromosomes and it is entirely plausible that epimarks acquired during early development do not persist equally among all chromosomes. Also, while DMRs are evenly distributed across chromosomes, retention of parallel epigenetic differences between wild and hatchery‐born adult Coho are mainly found on four specific chromosomes. This could be the result of stronger local effects allowing the detection of a significant signal at the chromosomal level. Moreover, we observed a significant enrichment for hypermethylated DMRs in hatchery‐born salmon. This pattern is also consistent with previous studies that reported significantly more hypermethylated than hypomethylated DMRs for hatchery‐born salmon (i.e., for juvenile Coho salmon (Le Luyer et al., 2017), adult Steelhead trout (Gavery et al., 2018), adult Altantic salmon (Rodriguez Barreto et al., 2019)).

It is noteworthy that the proportion of epigenetic variance explained by the rearing environment and shared between river systems (*R* ^2^ = 1.2%) on those four chromosomes is almost equal to that explained by the river of origin which is *R* ^2^ = 1.5% despite the fact that there is a clear genetic differentiation between salmon from both rivers but absence of significant genetic difference between hatchery vs. wild fish within each river. Also, the absence of genotypic differentiation between wild and hatchery‐born Coho and the fact that shared DMRs were observed in genetically distinct populations rule out the possibility that the observed modifications reflect relatedness among individuals. By showing that hatchery salmon are genetically much more similar (if not identical) to the wild counterpart from the same river than to hatchery salmon from the second river, our results strengthen the likelihood that early‐life hatchery‐rearing conditions may account for observed fitness differentials between wild and hatchery‐born individuals (Chittenden et al., 2008, 2010; Irvine et al., 2013; Zimmerman et al., 2015). Genetic variation explained 44.1% of methylation of DMRs between hatchery and wild‐born Coho. Partial genetic control of methylation variation was expected considering that more than 97% of CpGs variation was associated to genetic basis in humans (Zaghlool et al., 2016), and that important genetic variation associated to DNA methylation has been reported in whitefish (*Coregonus* sp.), another member of the salmonid family (Rougeux et al., 2019). Nevertheless, genetic variation did not explain the majority of the DMRs variation given that rearing environment explained 51.4% by itself, which supports the hypothesis that the rearing environment plays an important role in inducing these DMRs. Conversely, individual genetic variation explained 90.4% of the methylation variation for the DMRs associated to the river of origin. This highlights both the interplay between individual's genetic variation and methylation as previously documented in *Coregonus* species (Laporte et al., 2019; Rougeux et al., 2019) and the importance of the rearing environment in inducing methylation reprogramming.

Discrepancy between our results and those of Gavery et al. (2019) who observed no significant difference in the sperm methylome due to the rearing treatment in experimental conditions could be explained by several and non‐exclusive factors. First, as previously performed by Le Luyer et al. (2017), Gavery et al. (2019) used the reduced representation of the methylome (RRBS), screening about 5% of the genome, compared to the whole genome bisulfite sequencing (WGBS) used in our study. The latter approach provides a higher resolution that may have allowed locating DMRs found on specific chromosomes that could have been missed using RRBS. For instance, Le Luyer et al. (2017) identified 395,000 CpG sites using RRBS, in contrast to approximately 11,000,000 CpG sites identified here using WGBS, that is about 28 times more. Additionally, it is possible that the simulated stream and hatchery environments did not induce the same type of epigenomic modifications (i.e., reversible or permanent) as observed in wild and hatchery environments. This is supported by the fact that Gavery et al. (2018) observed epigenetic differences between wild and hatchery‐born Steelhead trout where wild fish were born and reared in their natural environment. Also, subsequent common growth in a tank compared to common growth in the sea may have different effects on the methylome. Moreover, all of the eggs used in the Gavery et al. (2019) experiment were fertilized and grown to the “eyed egg” stage in the hatchery. It may be that much of the hatchery‐induced embryonic methylome occurs in this early developmental period, which is supported by the study of Barreto et al. (Rodriguez Barreto et al., 2019). Finally, it is also possible that the effect of hatchery rearing on epigenetic reprogramming varies among species, thus calling for more empirical studies on other species. This highlights the sensitivity of epigenomic modifications and the importance to consider it in conservation biology and for future management strategies.

Among the 183 parallel DMRs found in this study, 58 were located within gene body (Baerwald et al., 2016; Le Luyer et al., 2017), which suggests a potential role in regulating gene expression. For example, early brain development is a critical period and environmental stress occurring during this critical period may drastically impact fish (Browman, 1989). Indeed, reduced brain size has been documented in hatchery‐reared rainbow trout compared to their wild congeners (Marchetti & Nevitt, 2003). Hypothetically, reduced brain development could reflect the hatchery hypermethylated regions associated with brain development in the current study (Table S4) and result in greater vulnerability to predation and lower survival in the wild (Marchetti & Nevitt, 2003). Similarly, wild Coho salmon were less resistant to disease after seven months of hatchery rearing (Salonius & Iwama, 1993) and hatchery‐reared individuals displayed lower swimming performance than wild Coho after seawater exposure (Brauner et al., 1994). Hypermethylation of genomic regions associated with immune response could underlie the disease susceptibility observed in hatchery fish and attributed to stress factors in the hatchery environment (Rehman et al., 2017; Salonius & Iwama, 1993).

Conversely, hypomethylated regions associated with cell maturation and lipid metabolic process observed in hatchery individuals could explain the difference in body morphology observed between wild and hatchery‐reared Coho salmon individuals (Swain et al., 1991). In Chinook salmon (*O*. *tshawytscha*) hatchery individuals matured precociously compared to wild individuals (Larsen et al., 2006), a possible outcome of the hypomethylated regions containing cell maturation genes found in our study. Hypomethylated sperm DMRs associated with metabolism were previously observed in juvenile white muscle. However, as no significant gene ontology enrichment was observed in this study, identification of the possible roles of the hypo‐ or hypermethylated transcripts remains hypothetical. Admittedly, these scenarios remain hypothetical until further studies providing evidence for variation in gene expression related to methylation level are performed to confirm the epigenetic regulation of genes important to individual fitness in fish.

To conclude, we documented the parallel persistence after more than one year spent at sea of similar environmentally induced epigenomic reprogramming acquired during early development in genetically distinct hatchery‐reared Coho salmon from two populations and demonstrated the potential for its inheritance through male germ cells. Whereas parallel environmentally induced epigenomic variation at the global scale (i.e., whole epigenome) was only marginally significant, we were able to detect epigenomic variation caused by rearing environment both at specific chromosomes and independent of chromosome size and at a local scale with 183 significant DMRs observed. As such, this study illustrates the benefits of performing whole genome bisulfite sequencing (WGBS) to dissect patterns of epigenomic variation at various genomic scales. Our results are also important for salmon conservation as they add further support to the view that environmental conditions during early development may account for at least some of the reduced fitness of the hatchery salmon in the wild which may also be transmitted to the next generation. Our study also further illustrates the relevance and importance of considering not only the genetic consequences, but also the epigenetic consequences of captive breeding in evaluating the costs and benefits of large‐scale supplementation programs to enhance wild populations.

## Supporting information

Fig S1

Fig S2

Fig S3

Table S1‐S3

Table S4

Appendix S1

Appendix S2

## Data Availability

The sequences reported in this paper will be available in the National Center for Biotechnology Information Sequence Read Archive, https://www.ncbi.nlm.nih.gov/sra/ (BioProject accession no. PRJNA678281).
