# Supplementary figures and images for: Epigenomic modifications induced by hatchery rearing persist in germ line cells of adult salmon after their oceanic migration
Source: Evol Appl. 2021 May 4;14(10):2402–13. doi: 10.1111/eva.13235 (PMC8549618; doi:10.1111/eva.13235)

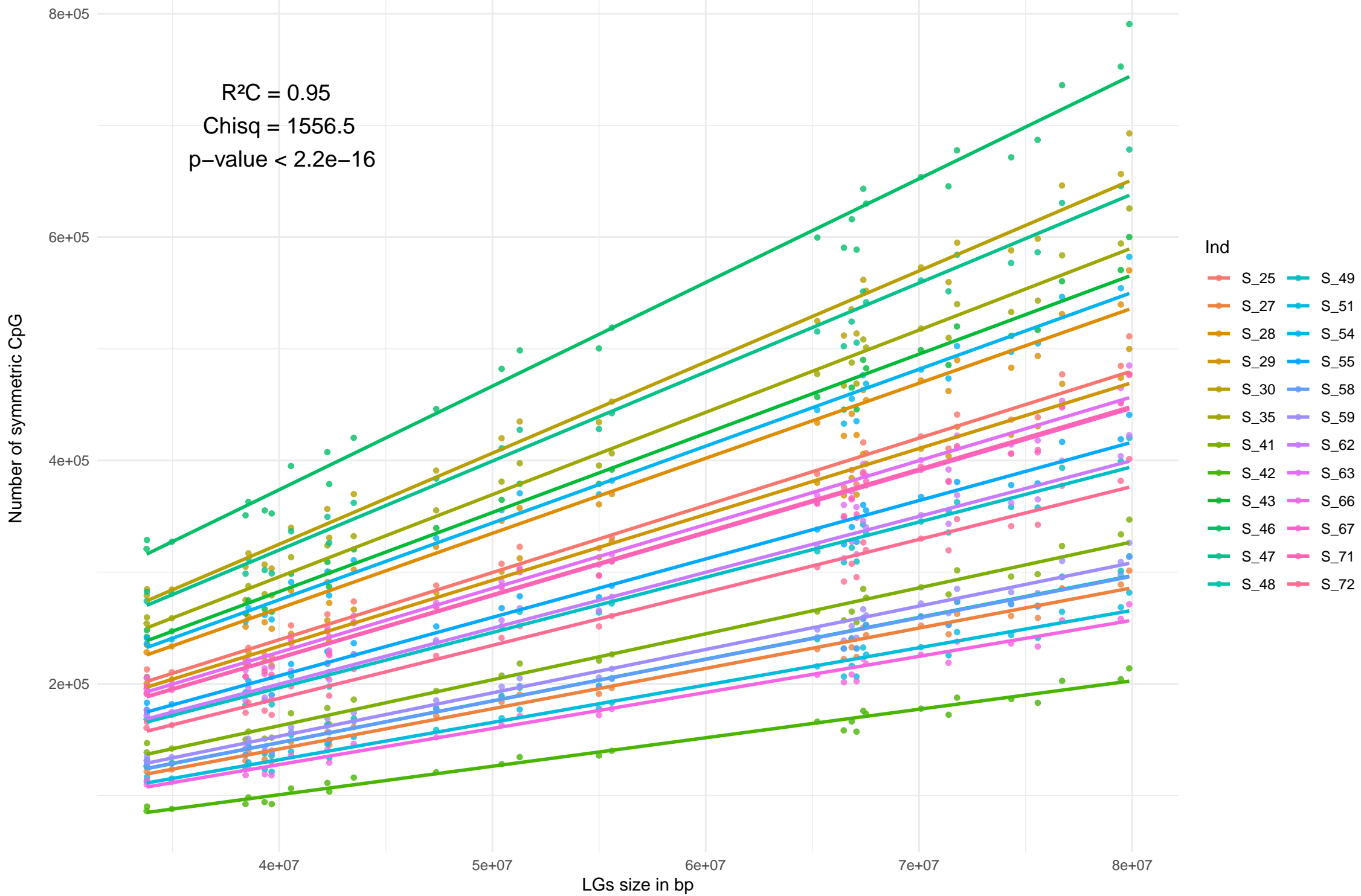

Supplement: Supplementary file 1 — Fig S1 [file EVA-14-2402-s004.pdf]

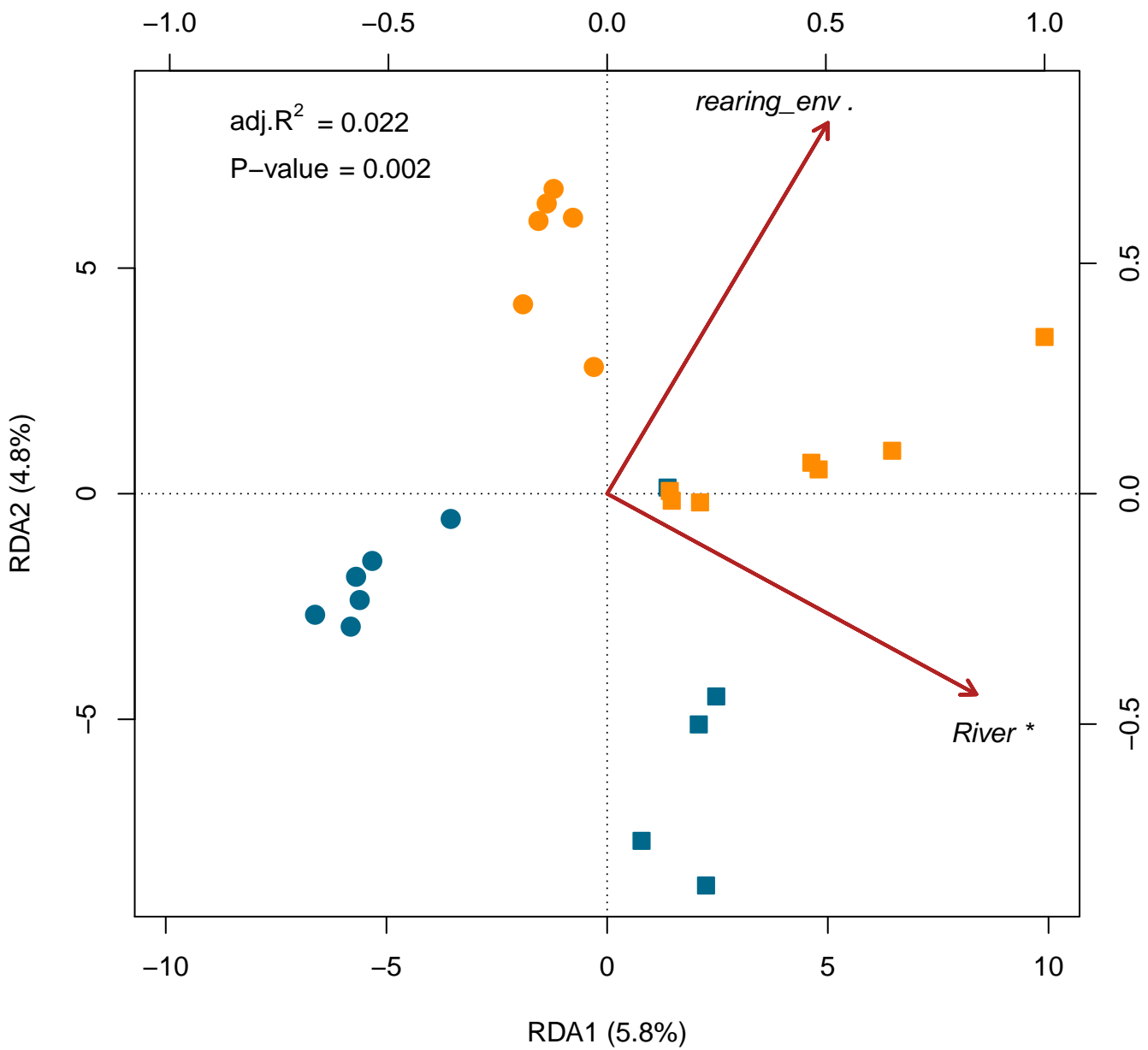

Supplement: Supplementary file 2 — Fig S2 [file EVA-14-2402-s005.pdf]

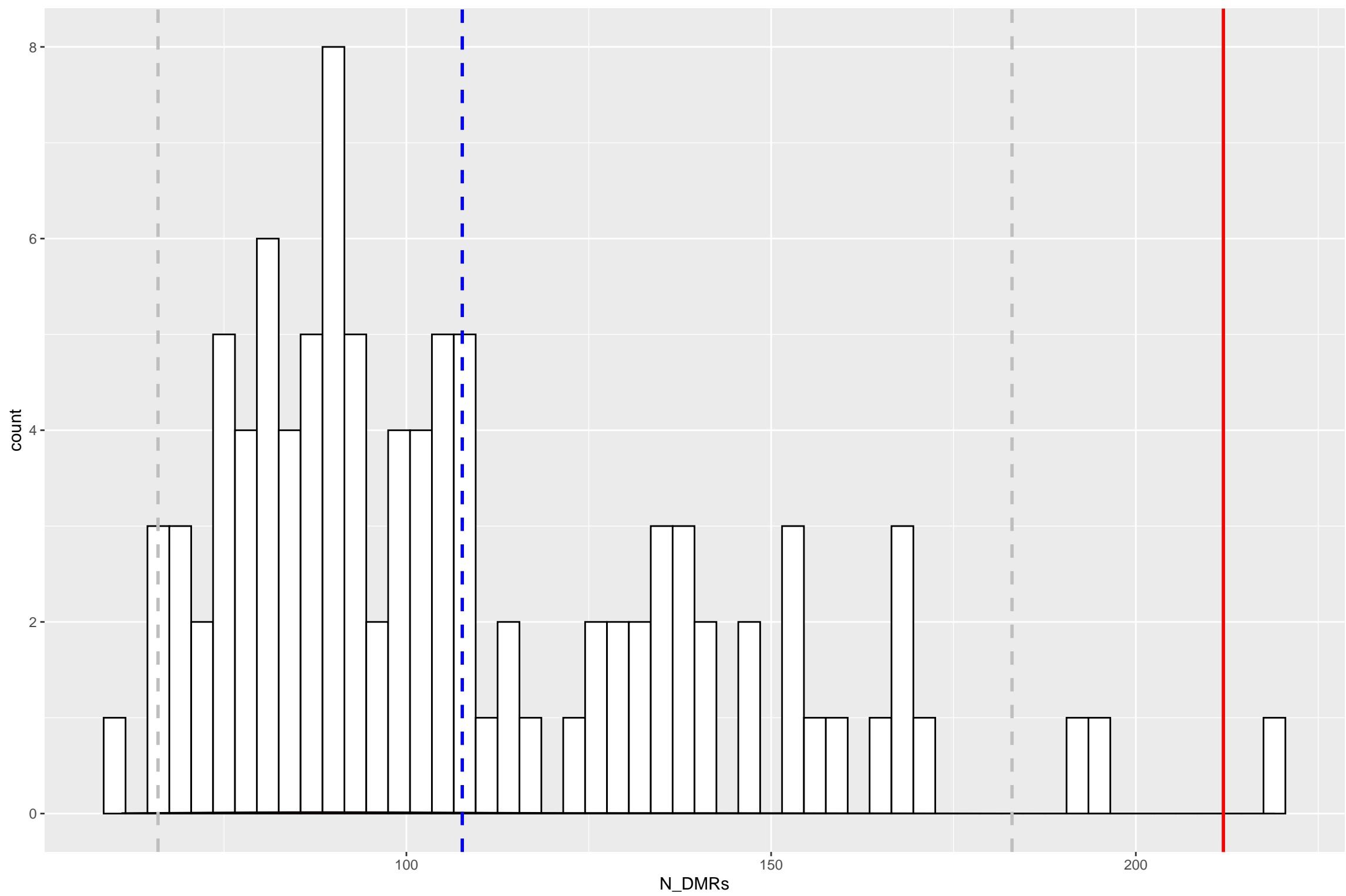

Supplement: Supplementary file 3 — Fig S3 [file EVA-14-2402-s006.pdf]
