# Supplementary material for: Epigenomic modifications induced by hatchery rearing persist in germ line cells of adult salmon after their oceanic migration
Source: Evol Appl. 2021 May 4;14(10):2402–13. doi: 10.1111/eva.13235 (PMC8549618; doi:10.1111/eva.13235)
Supplement: Supplementary file 7 — Appendix S2 [file EVA-14-2402-s001.pdf]

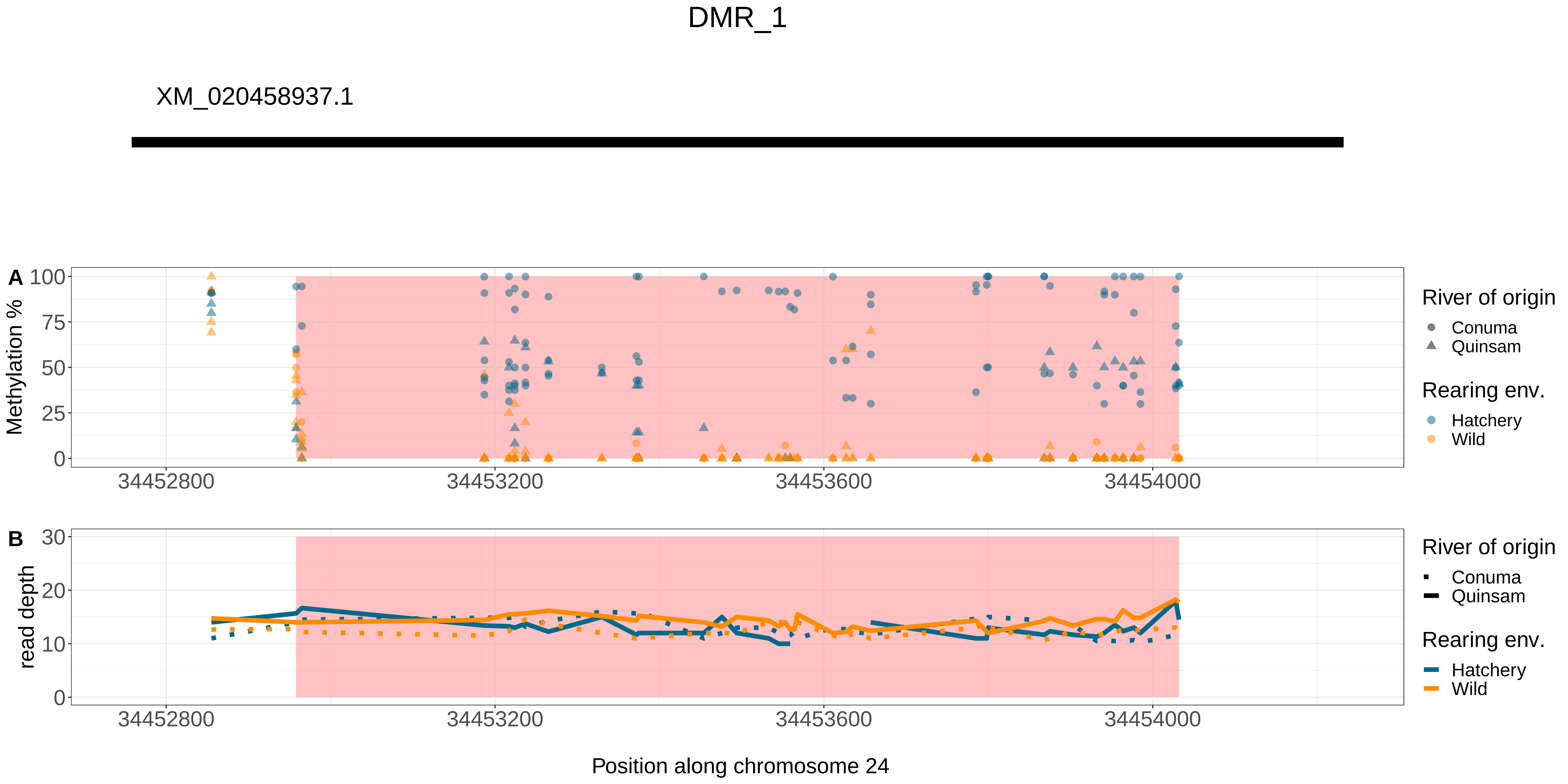
DMR_1
XM_020458937.1
A
Methylation %
100
75
50
25
0
34452800
34453200
34453600
34454000
River of origin
Conuma
Quinsam
Rearing env.
Hatchery
Wild
B
read depth
30
20
10
0
34452800
34453200
34453600
34454000
River of origin
Conuma
Quinsam
Rearing env.
Hatchery
Wild
Position along chromosome 24

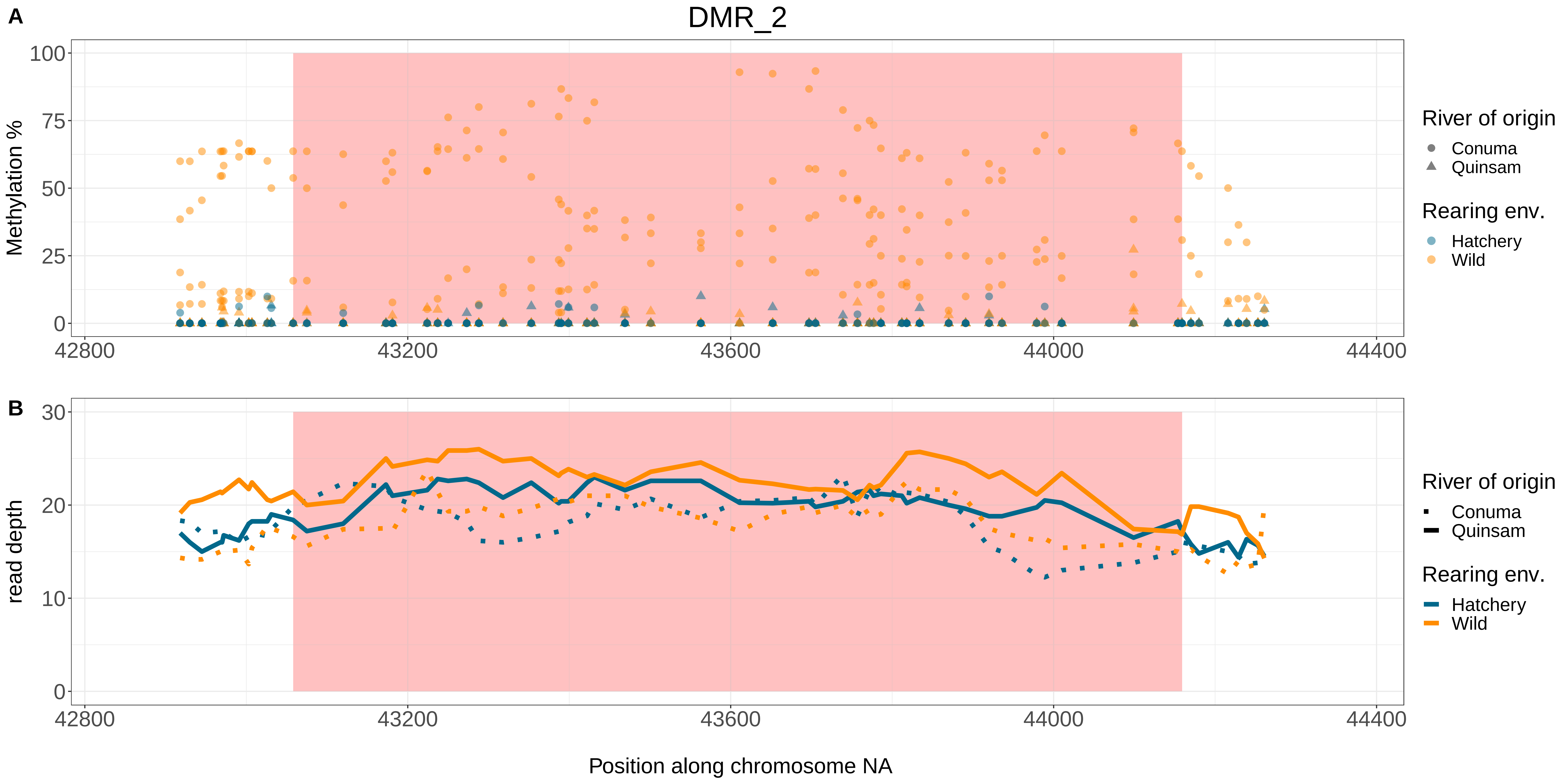

A
DMR_2
Methylation %
100
75
50
25
0
42800
43200
43600
44000
44400
River of origin
Conuma
Quinsam
Rearing env.
Hatchery
Wild
B
read depth
30
20
10
0
42800
43200
43600
44000
44400
River of origin
Conuma
Quinsam
Rearing env.
Hatchery
Wild
Position along chromosome NA

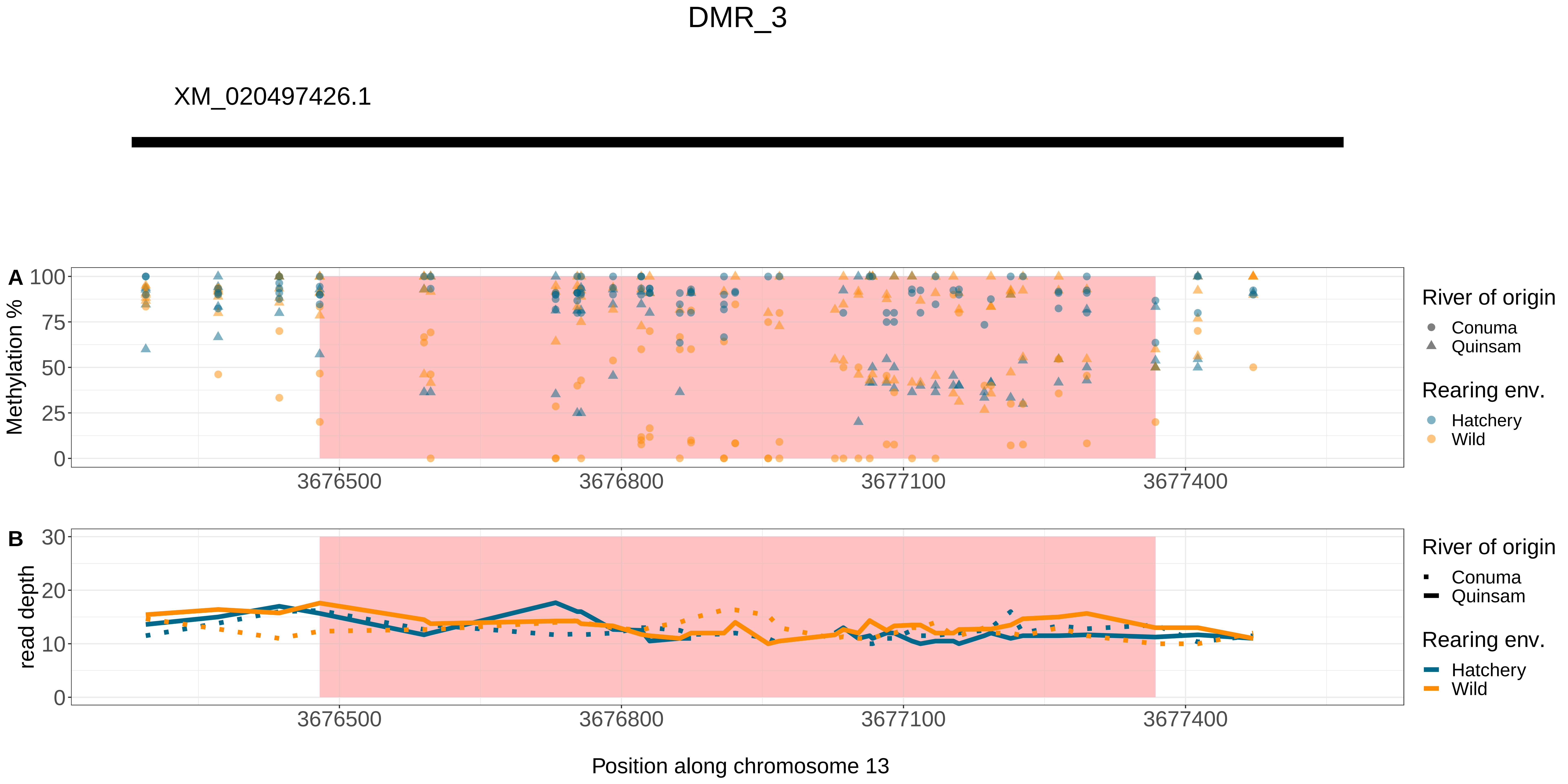
DMR_3
XM_020497426.1
A
Methylation %
100
75
50
25
0
3676500
3676800
3677100
3677400
River of origin
Conuma
Quinsam
Rearing env.
Hatchery
Wild
B
read depth
30
20
10
0
Position along chromosome 13

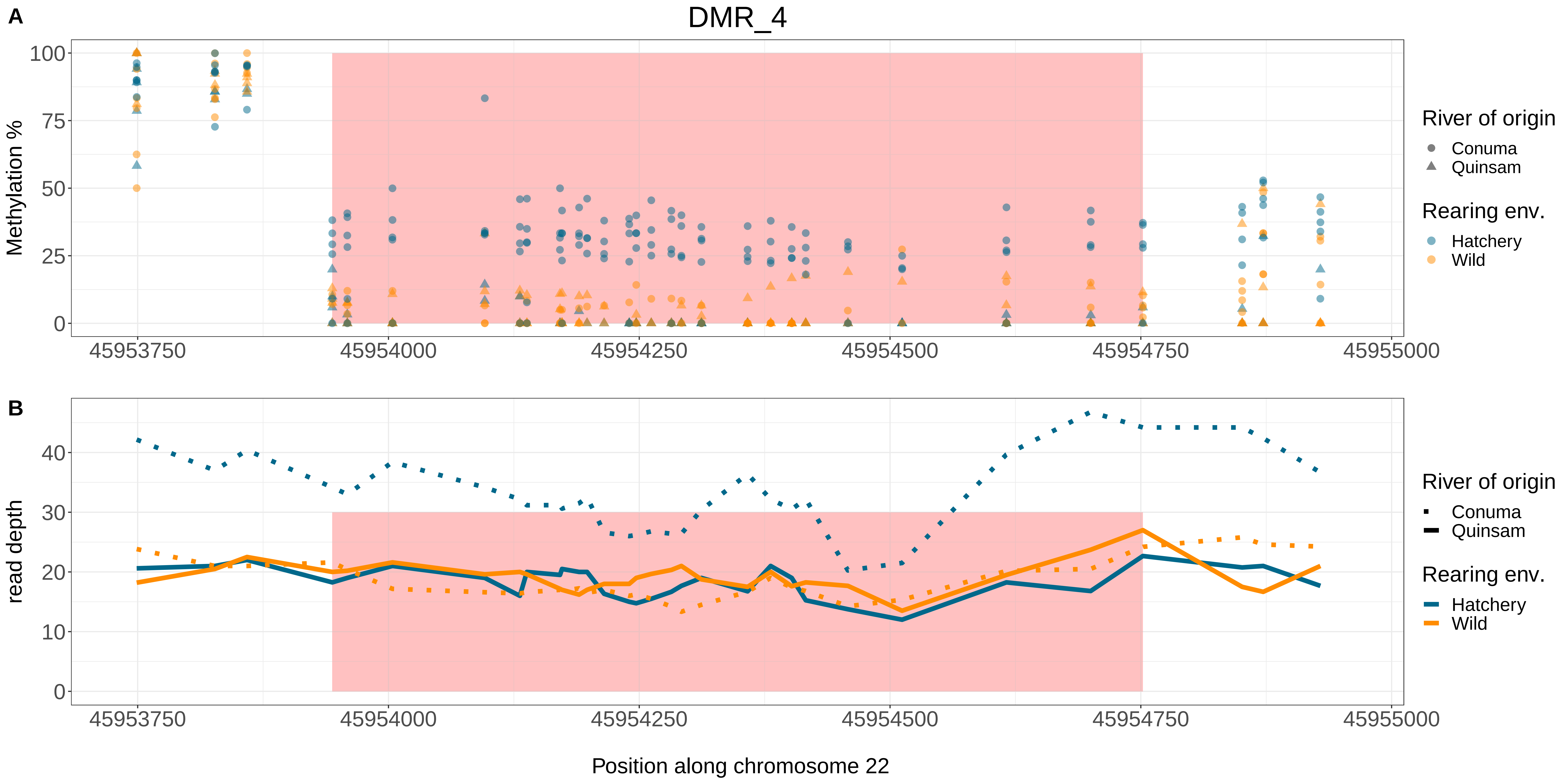

A
DMR_4
Methylation %
100
75
50
25
0
45953750
45954000
45954250
45954500
45954750
45955000
River of origin
Conuma
Quinsam
Rearing env.
Hatchery
Wild
B
read depth
40
30
20
10
0
45953750
45954000
45954250
45954500
45954750
45955000
River of origin
Conuma
Quinsam
Rearing env.
Hatchery
Wild
Position along chromosome 22

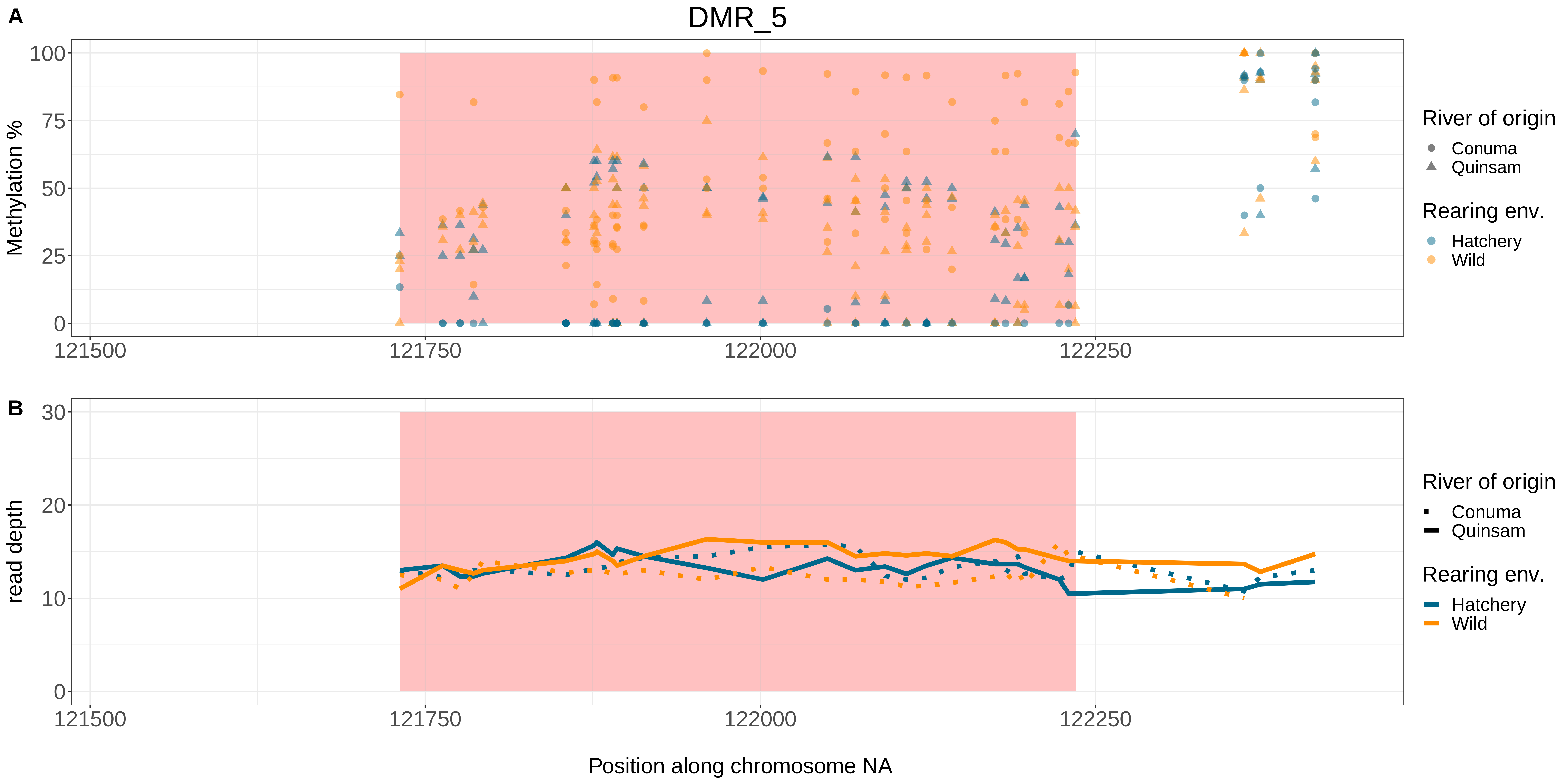
A
DMR_5
Methylation %
100
75
50
25
0
121500
121750
122000
122250
River of origin
Conuma
Quinsam
Rearing env.
Hatchery
Wild
B
read depth
30
20
10
0
121500
121750
122000
122250
River of origin
Conuma
Quinsam
Rearing env.
Hatchery
Wild
Position along chromosome NA

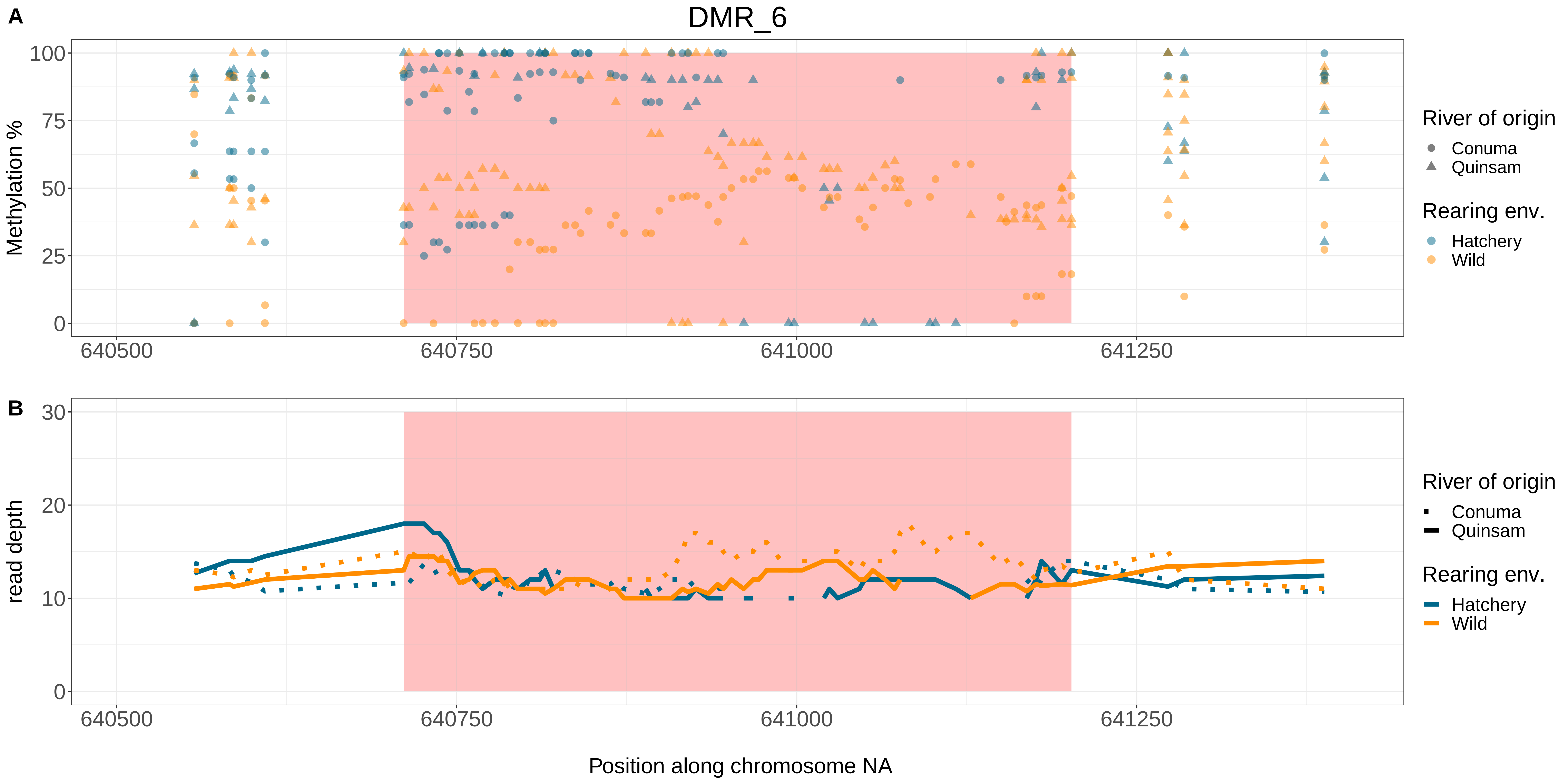

A
DMR_6
Methylation %
100
75
50
25
0
640500
640750
641000
641250
River of origin
Conuma
Quinsam
Rearing env.
Hatchery
Wild
B
read depth
30
20
10
0
640500
640750
641000
641250
River of origin
Conuma
Quinsam
Rearing env.
Hatchery
Wild
Position along chromosome NA

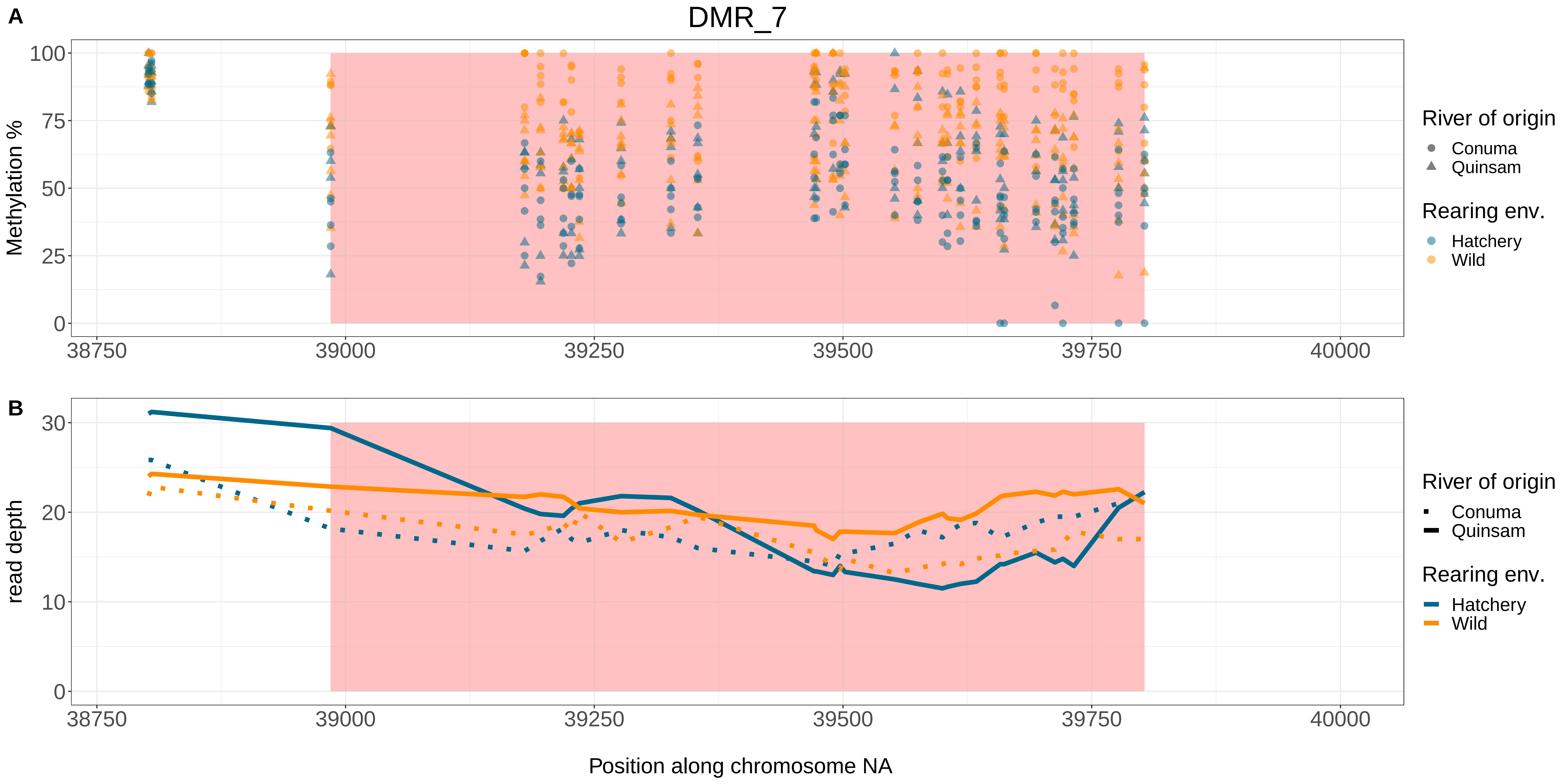

A
DMR_7
Methylation %
100
75
50
25
0
38750
39000
39250
39500
39750
40000
River of origin
Conuma
Quinsam
Rearing env.
Hatchery
Wild
B
read depth
30
20
10
0
38750
39000
39250
39500
39750
40000
River of origin
Conuma
Quinsam
Rearing env.
Hatchery
Wild
Position along chromosome NA

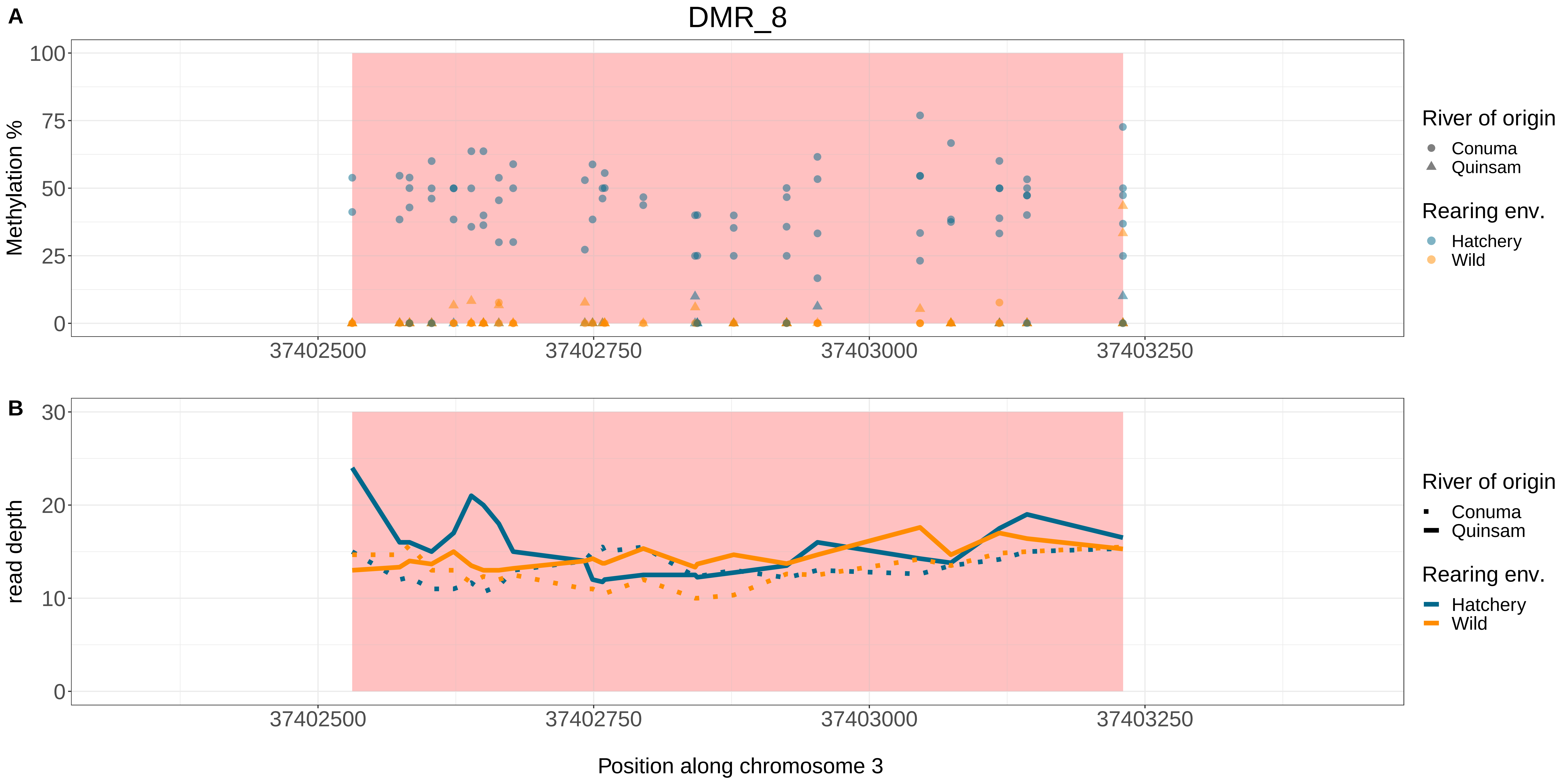

A
DMR_8
Methylation %
100
75
50
25
0
37402500
37402750
37403000
37403250
River of origin
Conuma
Quinsam
Rearing env.
Hatchery
Wild
B
read depth
30
20
10
0
37402500
37402750
37403000
37403250
River of origin
Conuma
Quinsam
Rearing env.
Hatchery
Wild
Position along chromosome 3

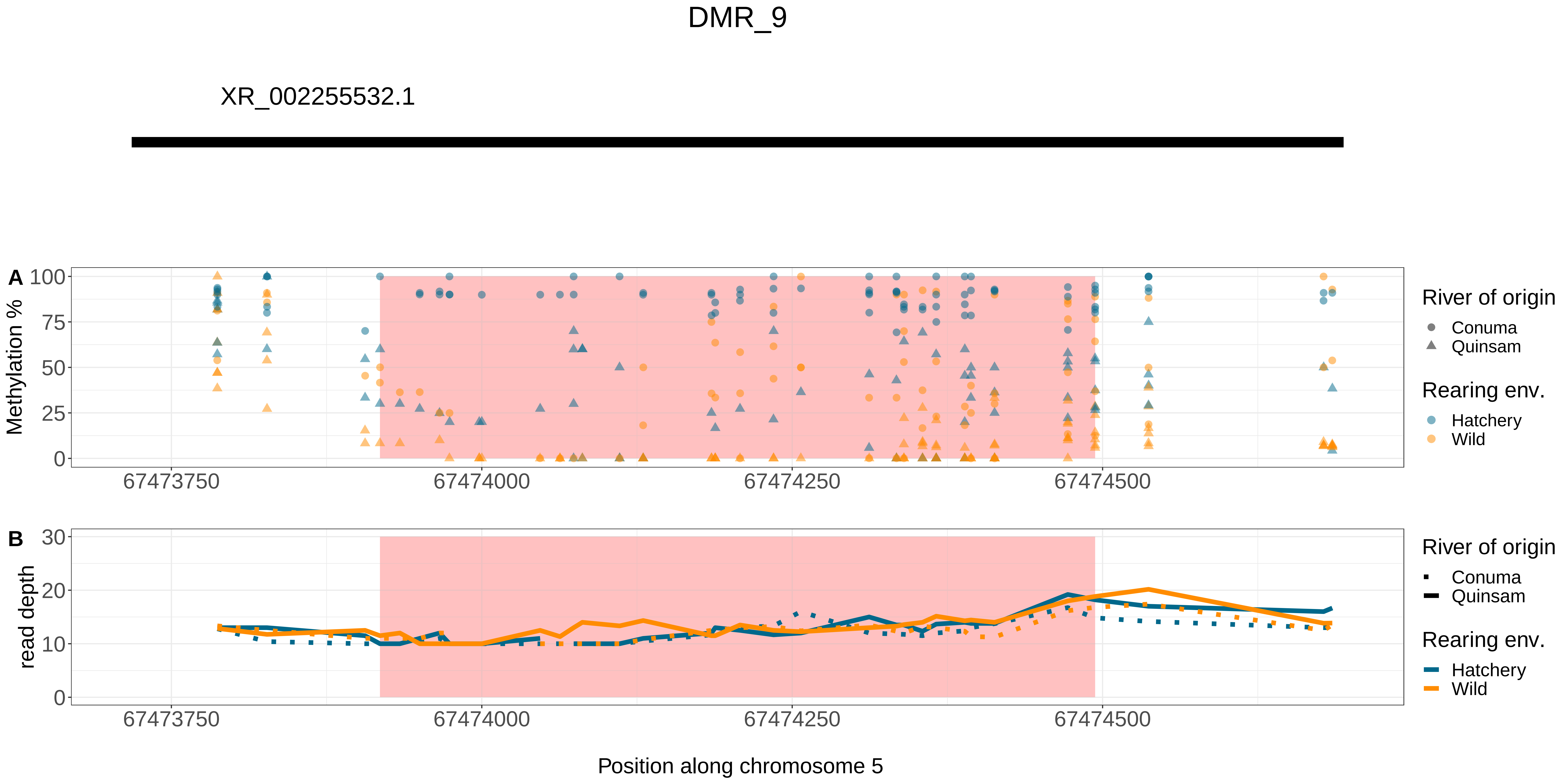

DMR_9
XR_002255532.1
A
B
Methylation %
read depth
100
75
50
25
0
30
20
10
67473750
67474000
67474250
67474500
Position along chromosome 5
River of origin
Conuma
Quinsam
Rearing env.
Hatchery
Wild

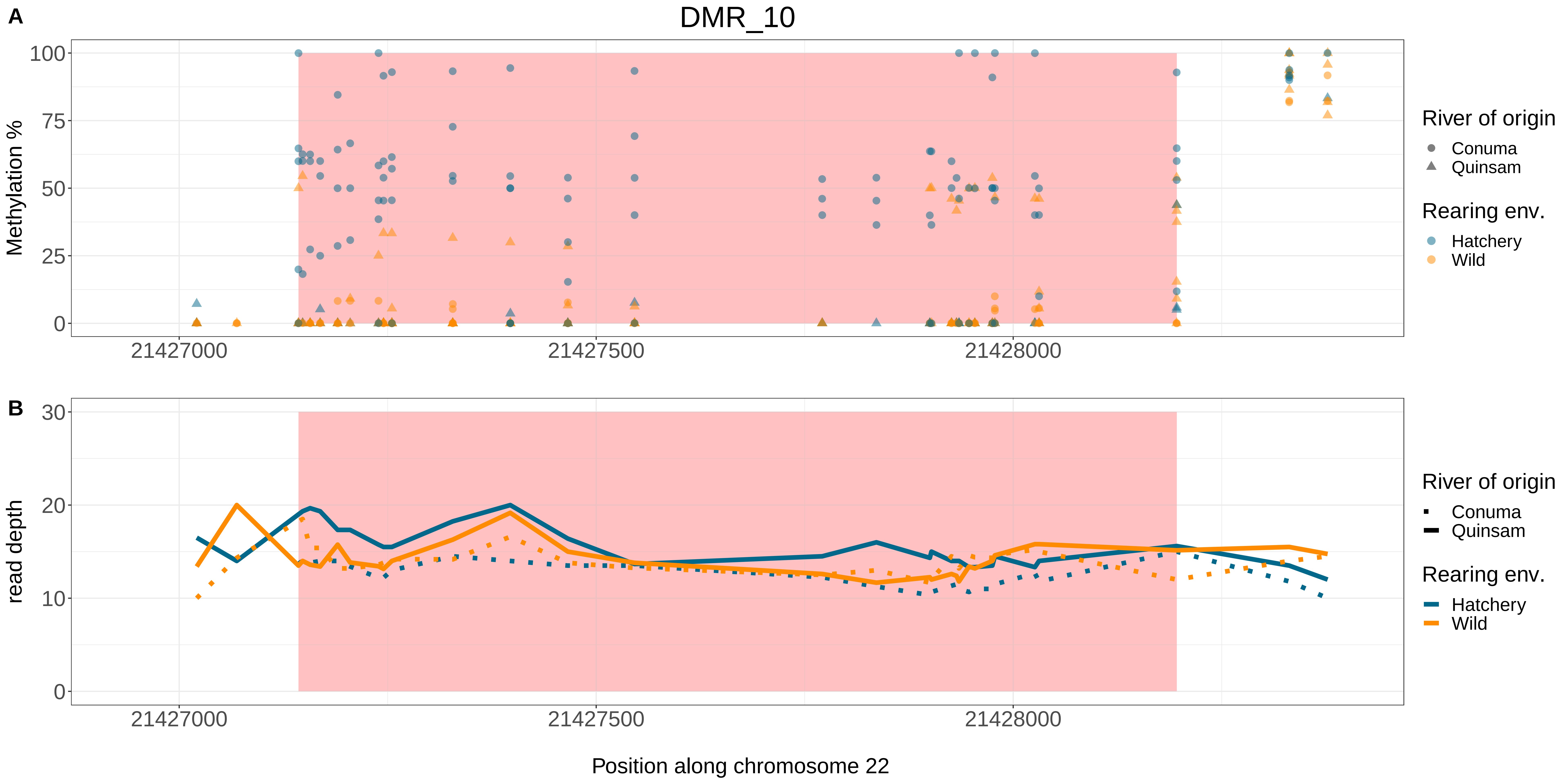

A
DMR_10
Methylation %
100
75
50
25
0
21427000
21427500
21428000
River of origin
Conuma
Quinsam
Rearing env.
Hatchery
Wild
B
read depth
30
20
10
0
21427000
21427500
21428000
River of origin
Conuma
Quinsam
Rearing env.
Hatchery
Wild
Position along chromosome 22

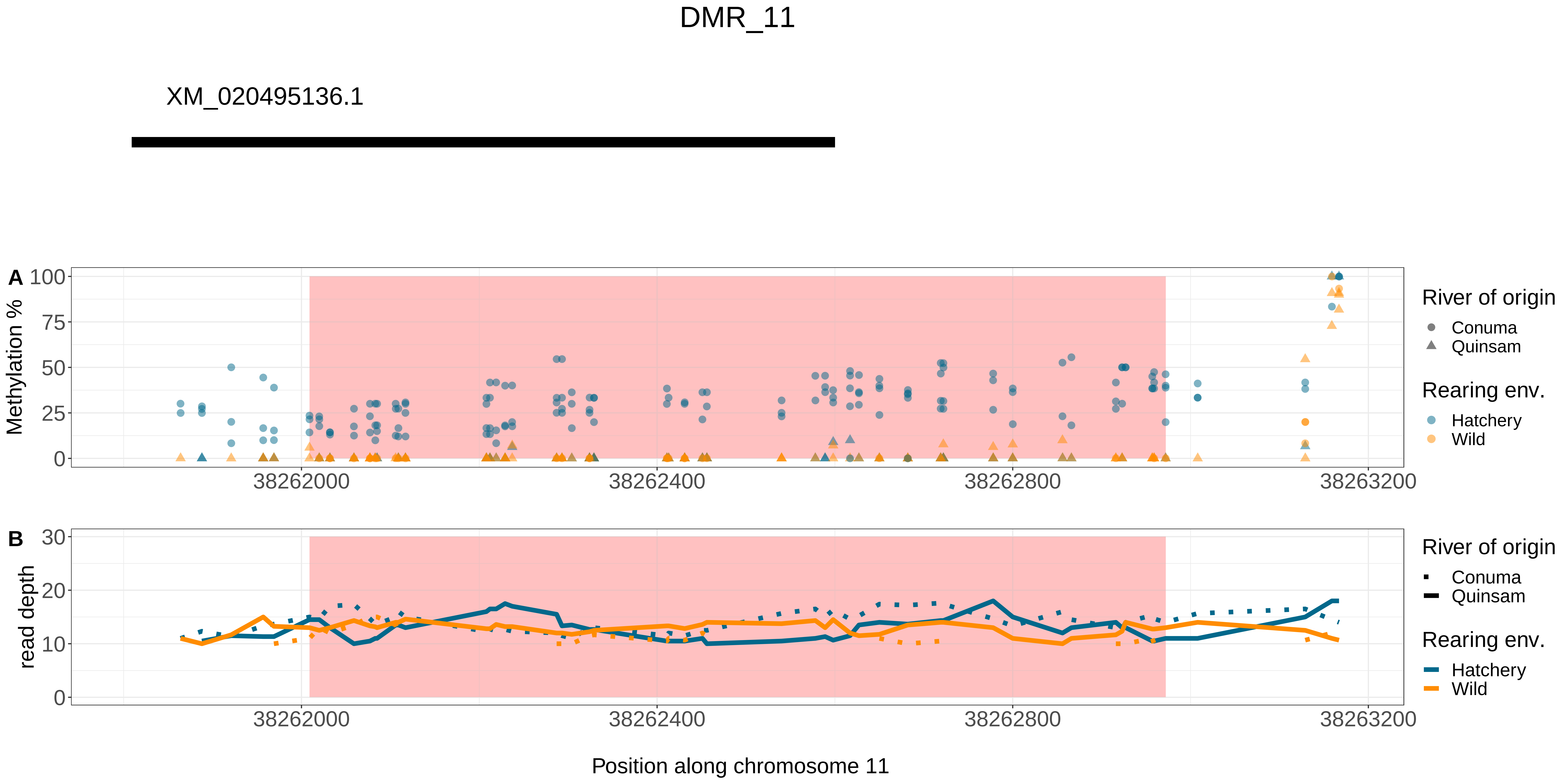

DMR_11
XM_020495136.1
A
Methylation %
100
75
50
25
0
38262000
38262400
38262800
38263200
River of origin
Conuma
Quinsam
Rearing env.
Hatchery
Wild
B
read depth
30
20
10
0
38262000
38262400
38262800
38263200
River of origin
Conuma
Quinsam
Rearing env.
Hatchery
Wild
Position along chromosome 11

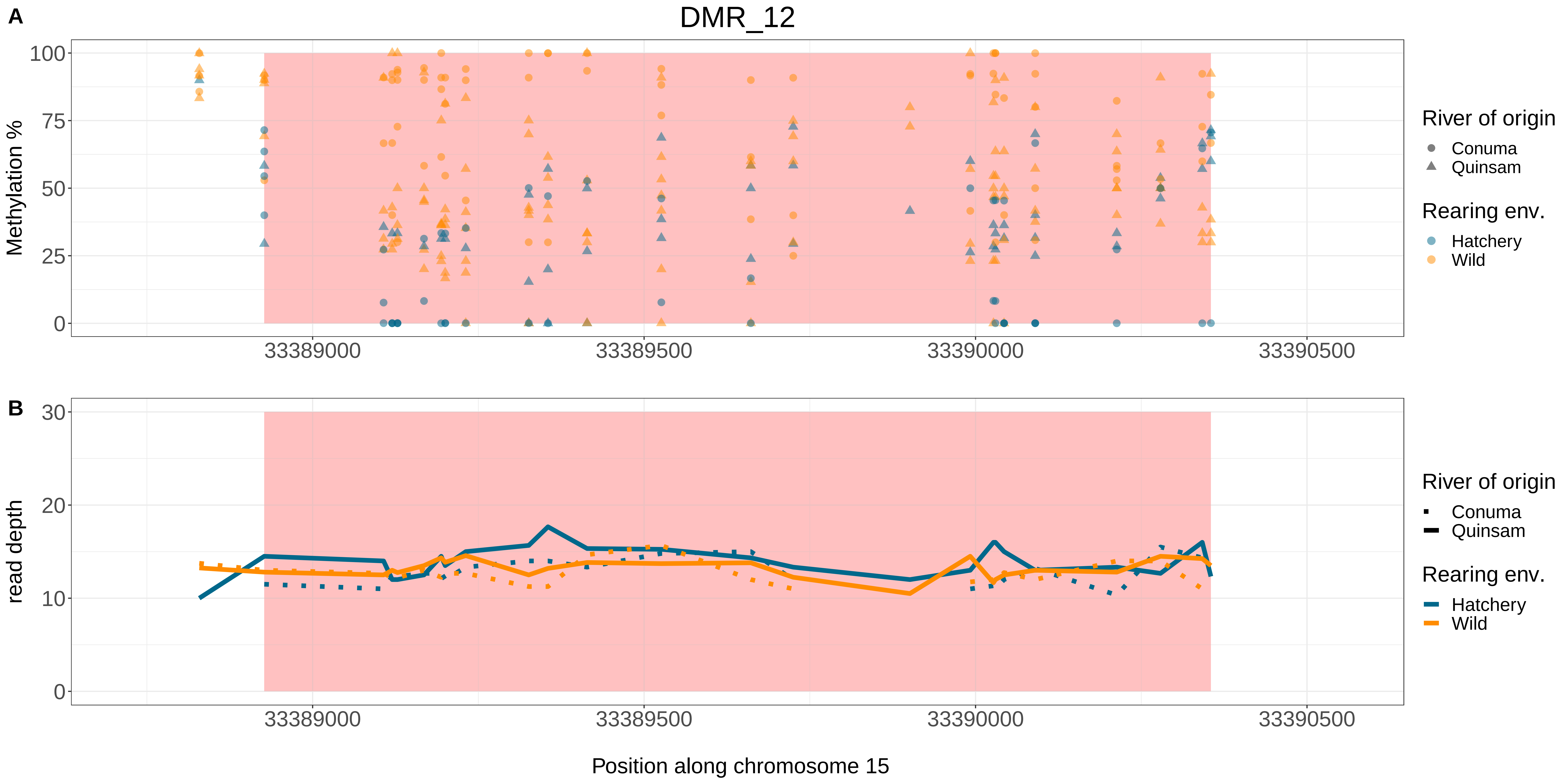

A
DMR_12
Methylation %
100
75
50
25
0
33389000
33389500
33390000
33390500
River of origin
Conuma
Quinsam
Rearing env.
Hatchery
Wild
B
read depth
30
20
10
0
33389000
33389500
33390000
33390500
River of origin
Conuma
Quinsam
Rearing env.
Hatchery
Wild
Position along chromosome 15

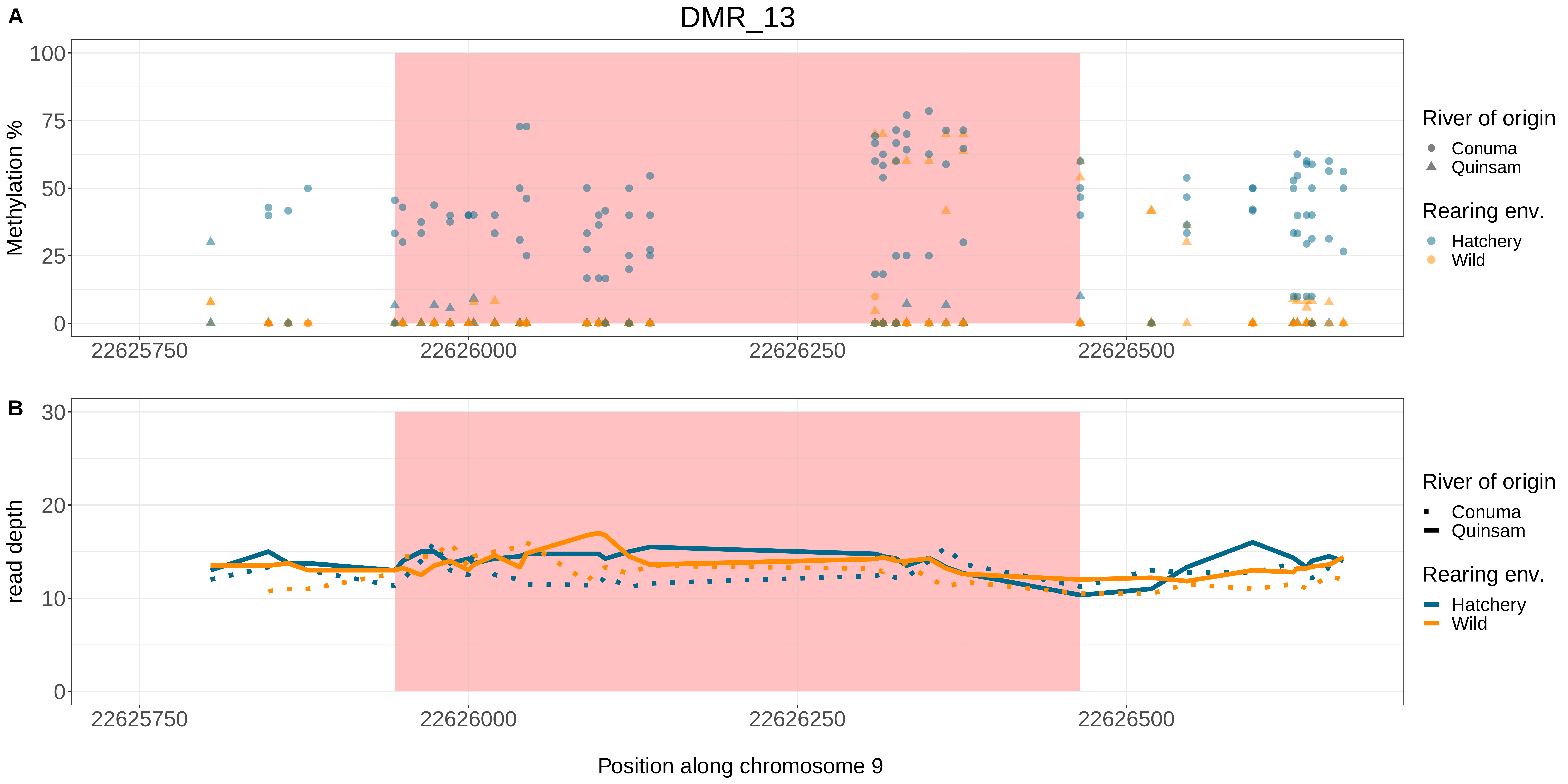

A
DMR_13
Methylation %
100
75
50
25
0
22625750
22626000
22626250
22626500
River of origin
Conuma
Quinsam
Rearing env.
Hatchery
Wild
B
read depth
30
20
10
0
22625750
22626000
22626250
22626500
River of origin
Conuma
Quinsam
Rearing env.
Hatchery
Wild
Position along chromosome 9

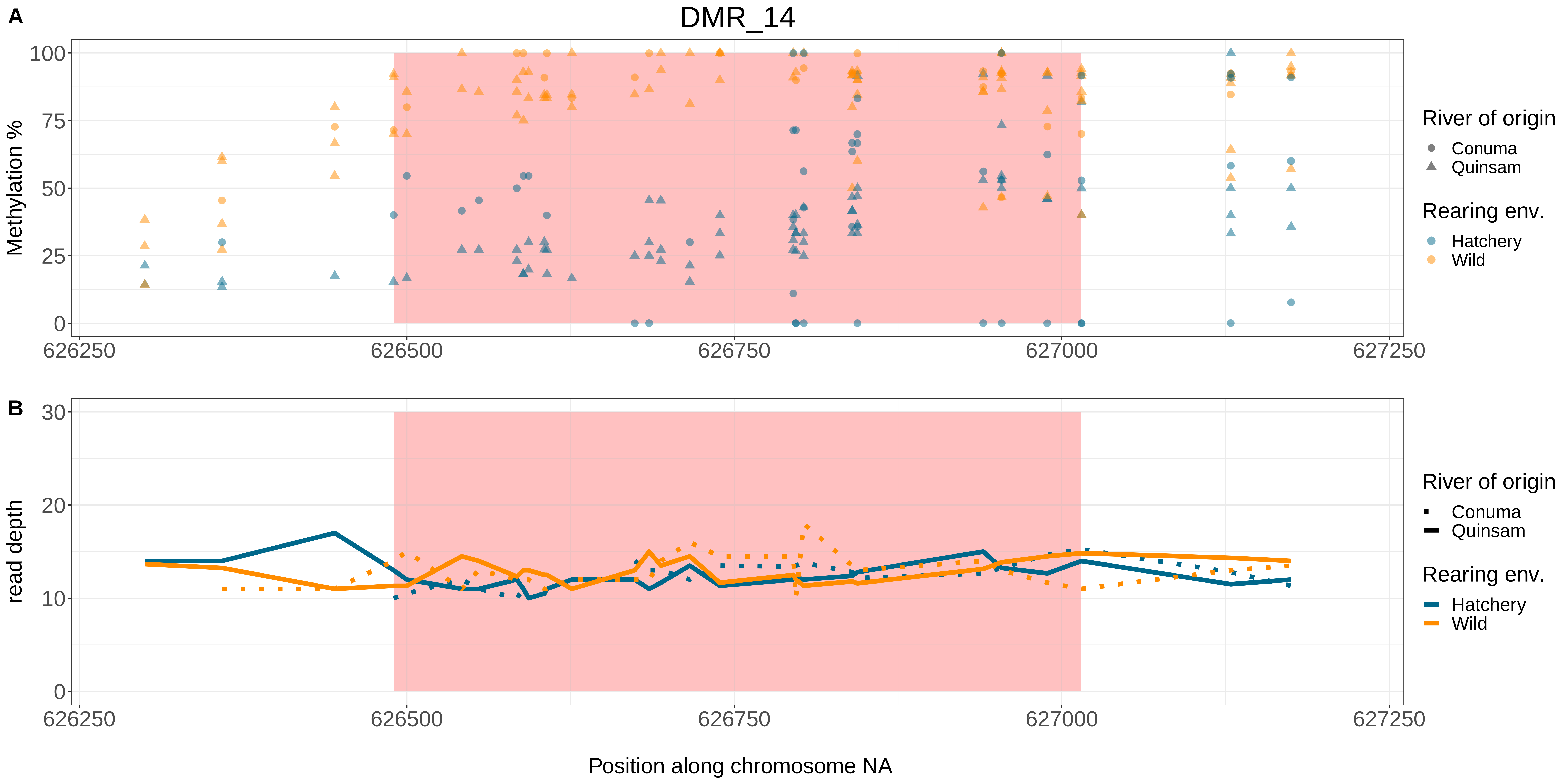

A
DMR_14
Methylation %
100
75
50
25
0
626250
626500
626750
627000
627250
River of origin
Conuma
Quinsam
Rearing env.
Hatchery
Wild
B
read depth
30
20
10
0
626250
626500
626750
627000
627250
River of origin
Conuma
Quinsam
Rearing env.
Hatchery
Wild
Position along chromosome NA

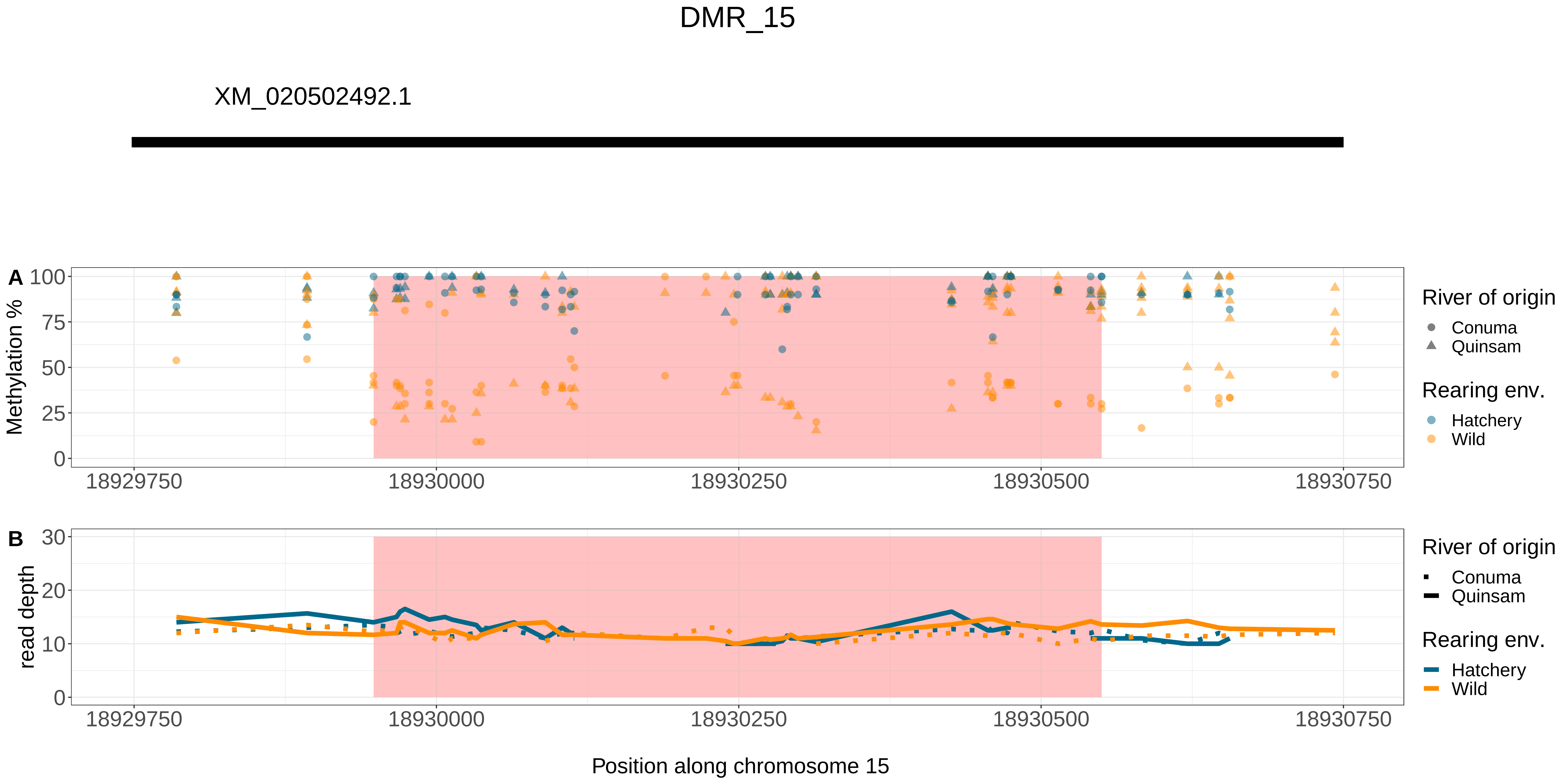

DMR_15
XM_020502492.1
A
Methylation %
100
75
50
25
0
18929750
18930000
18930250
18930500
18930750
River of origin
Conuma
Quinsam
Rearing env.
Hatchery
Wild
B
read depth
30
20
10
0
18929750
18930000
18930250
18930500
18930750
River of origin
Conuma
Quinsam
Rearing env.
Hatchery
Wild
Position along chromosome 15

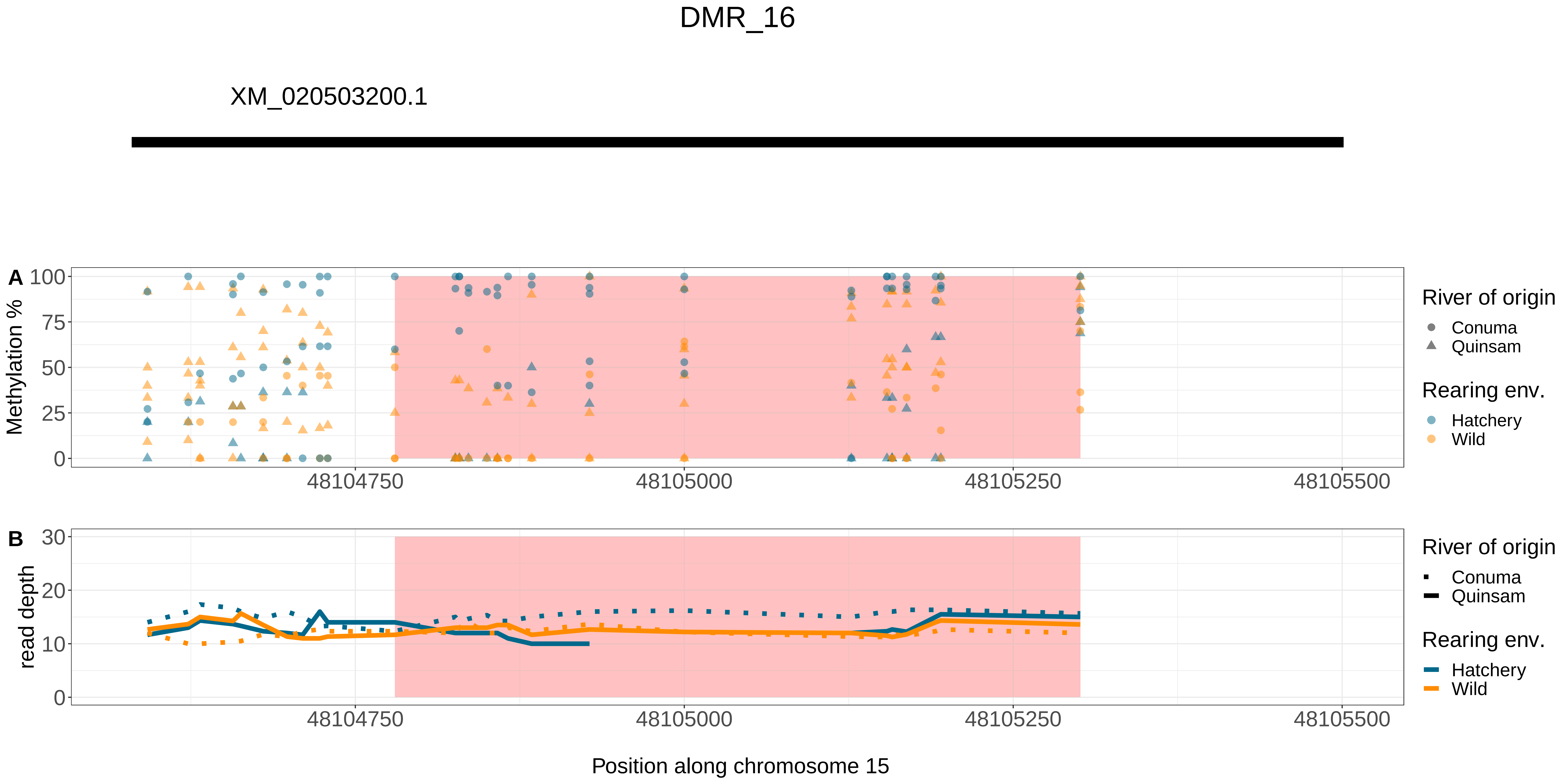
DMR_16
XM_020503200.1
A
Methylation %
100
75
50
25
0
48104750
48105000
48105250
48105500
River of origin
Conuma
Quinsam
Rearing env.
Hatchery
Wild
B
read depth
30
20
10
0
48104750
48105000
48105250
48105500
River of origin
Conuma
Quinsam
Rearing env.
Hatchery
Wild
Position along chromosome 15

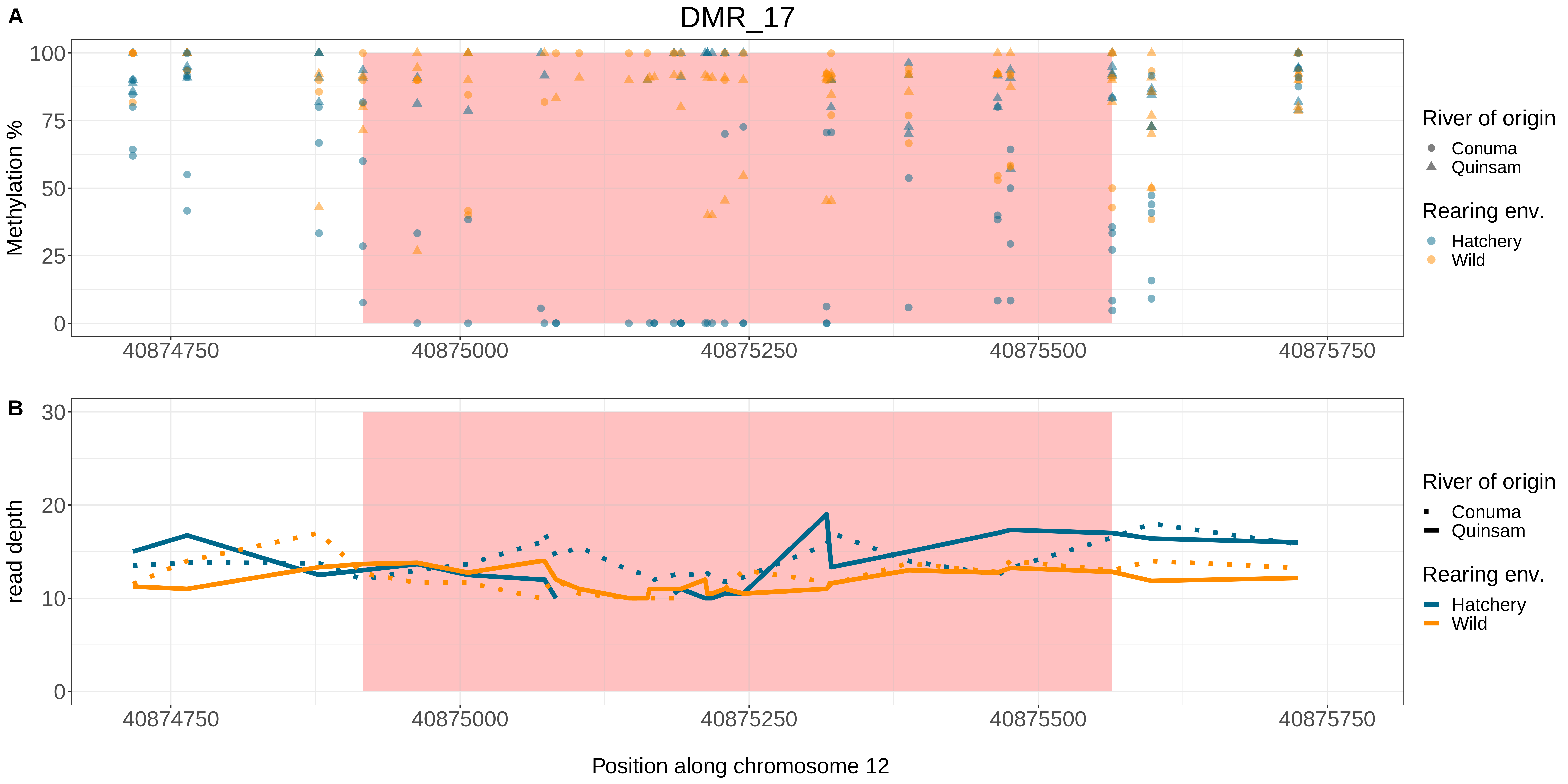

A
DMR_17
Methylation %
100
75
50
25
0
40874750
40875000
40875250
40875500
40875750
River of origin
Conuma
Quinsam
Rearing env.
Hatchery
Wild
B
read depth
30
20
10
0
40874750
40875000
40875250
40875500
40875750
River of origin
Conuma
Quinsam
Rearing env.
Hatchery
Wild
Position along chromosome 12

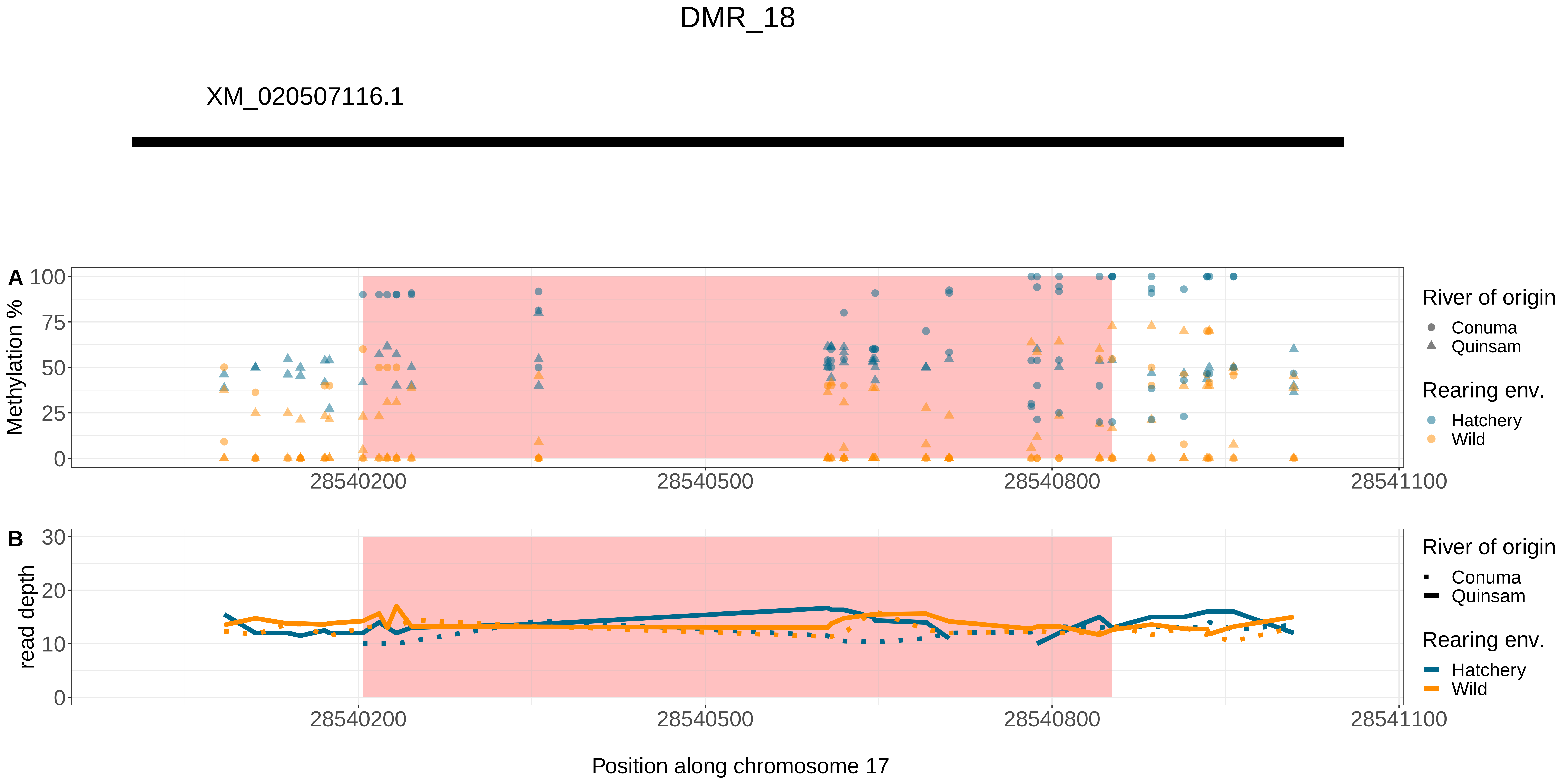

DMR_18
XM_020507116.1
A
Methylation %
100
75
50
25
0
28540200
28540500
28540800
28541100
River of origin
Conuma
Quinsam
Rearing env.
Hatchery
Wild
B
read depth
30
20
10
0
28540200
28540500
28540800
28541100
River of origin
Conuma
Quinsam
Rearing env.
Hatchery
Wild
Position along chromosome 17

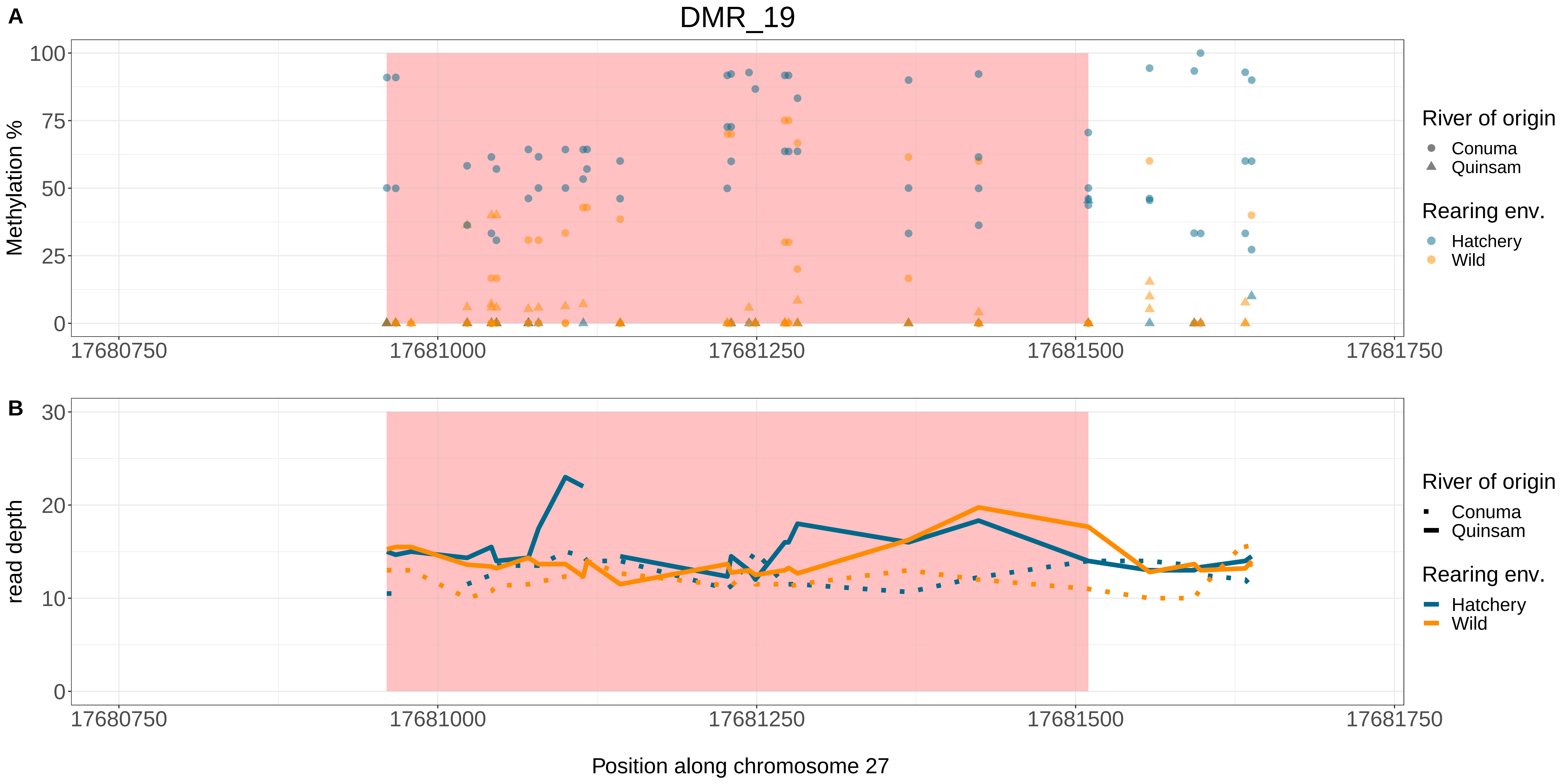

DMR_19
A
Methylation %
100
75
50
25
0
17680750
17681000
17681250
17681500
17681750
River of origin
Conuma
Quinsam
Rearing env.
Hatchery
Wild
B
read depth
30
20
10
0
17680750
17681000
17681250
17681500
17681750
River of origin
Conuma
Quinsam
Rearing env.
Hatchery
Wild
Position along chromosome 27

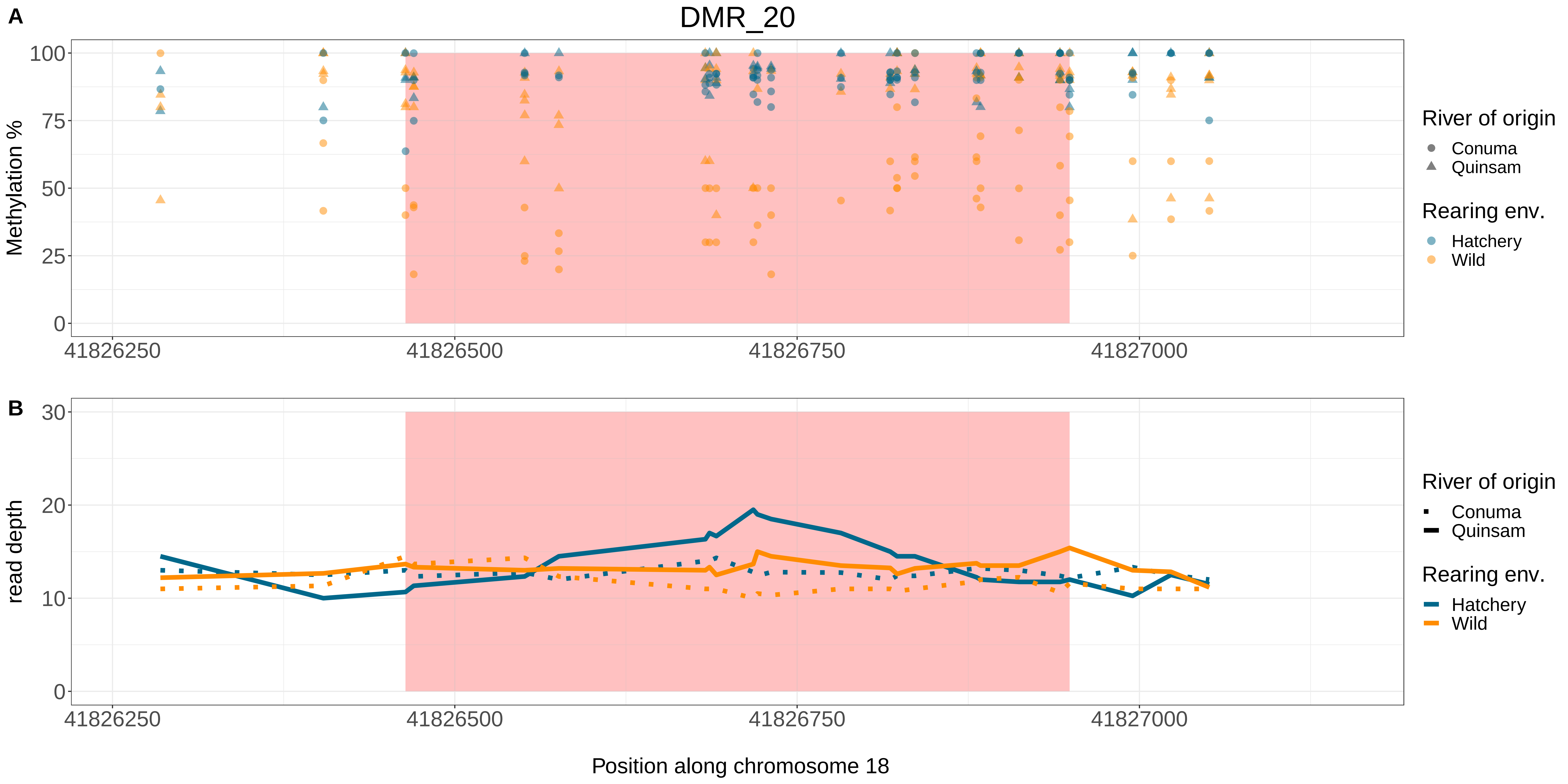

A
DMR_20
Methylation %
100
75
50
25
0
41826250
41826500
41826750
41827000
River of origin
Conuma
Quinsam
Rearing env.
Hatchery
Wild
B
read depth
30
20
10
0
41826250
41826500
41826750
41827000
River of origin
Conuma
Quinsam
Rearing env.
Hatchery
Wild
Position along chromosome 18

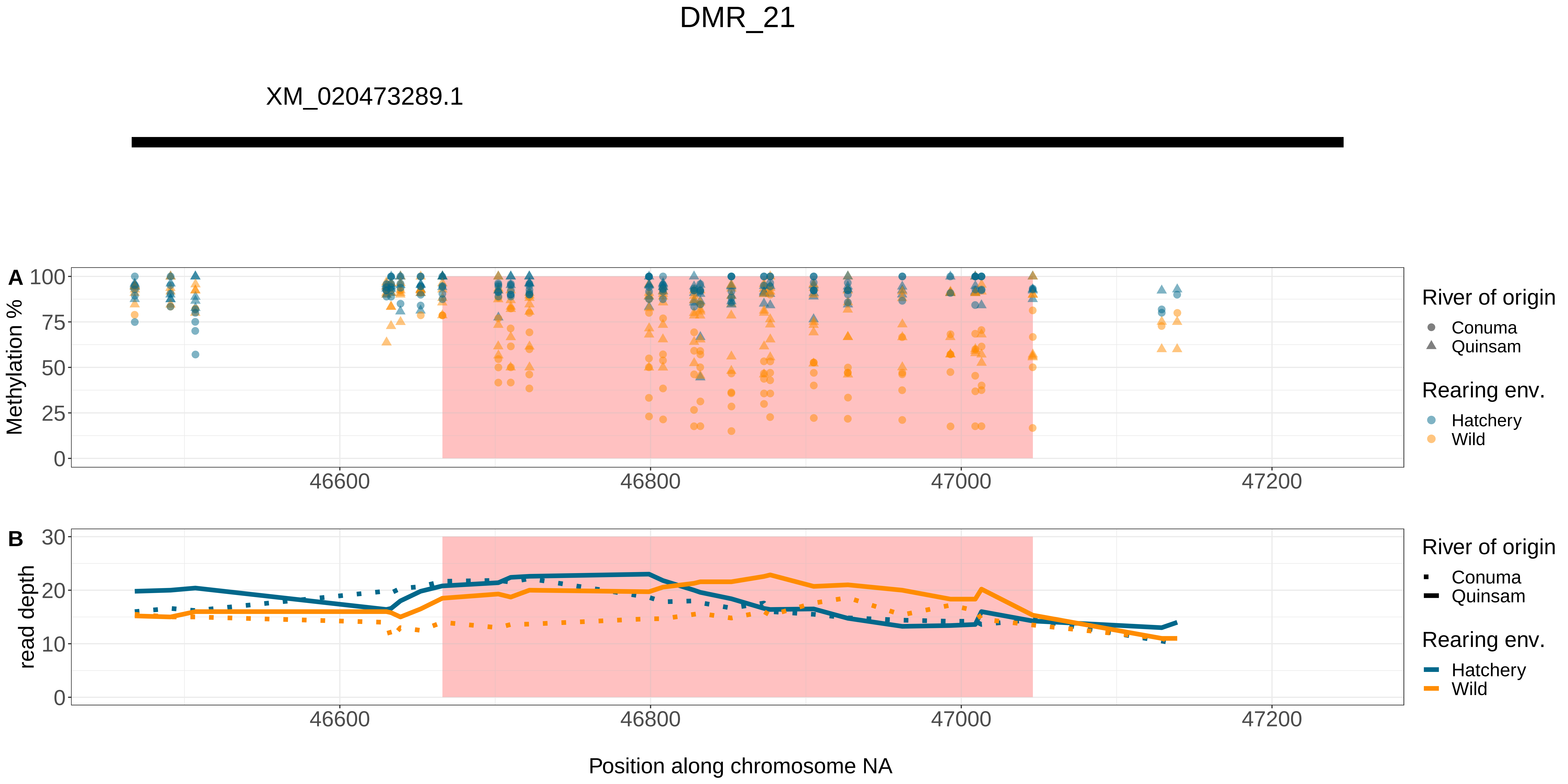

DMR_21
XM_020473289.1
A
Methylation %
100
75
50
25
0
46600
46800
47000
47200
River of origin
Conuma
Quinsam
Rearing env.
Hatchery
Wild
B
read depth
30
20
10
0
46600
46800
47000
47200
River of origin
Conuma
Quinsam
Rearing env.
Hatchery
Wild
Position along chromosome NA

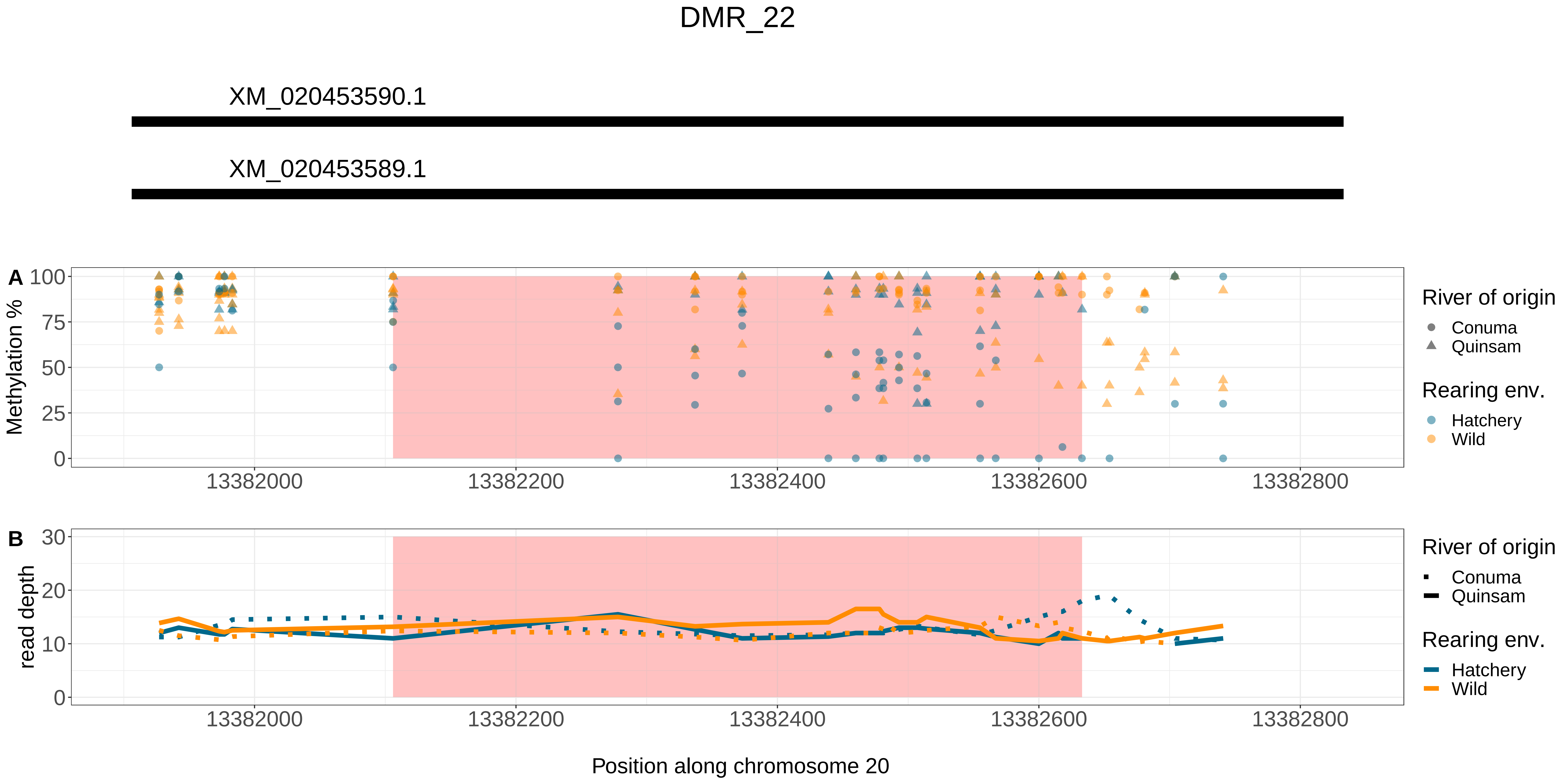

DMR_22
XM_020453590.1
XM_020453589.1
A
Methylation %
100
75
50
25
0
13382000
13382200
13382400
13382600
13382800
B
read depth
30
20
10
0
Position along chromosome 20
River of origin
Conuma
Quinsam
Rearing env.
Hatchery
Wild

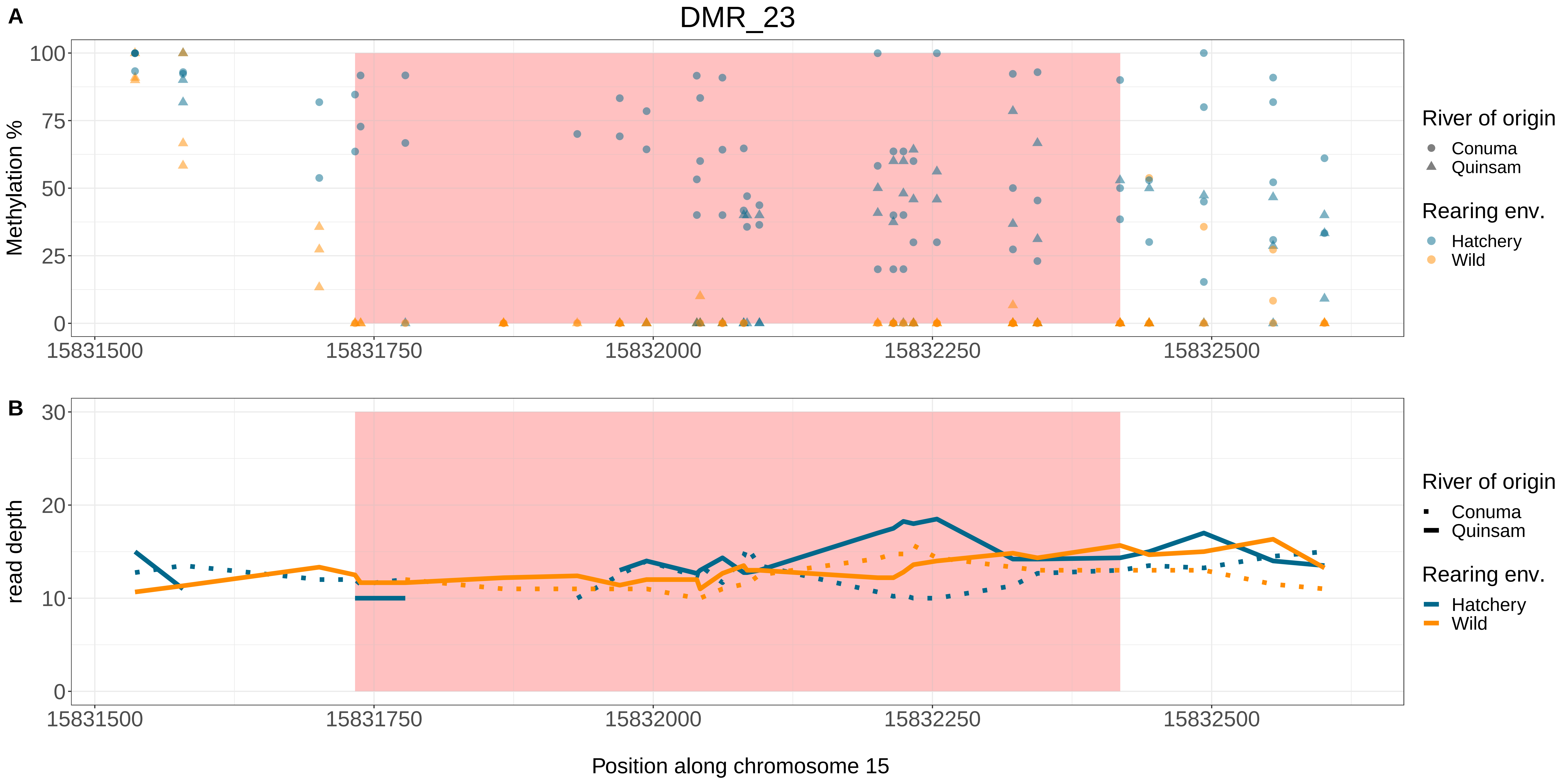

A
DMR_23
Methylation %
100
75
50
25
0
15831500
15831750
15832000
15832250
15832500
River of origin
Conuma
Quinsam
Rearing env.
Hatchery
Wild
B
read depth
30
20
10
0
15831500
15831750
15832000
15832250
15832500
River of origin
Conuma
Quinsam
Rearing env.
Hatchery
Wild
Position along chromosome 15

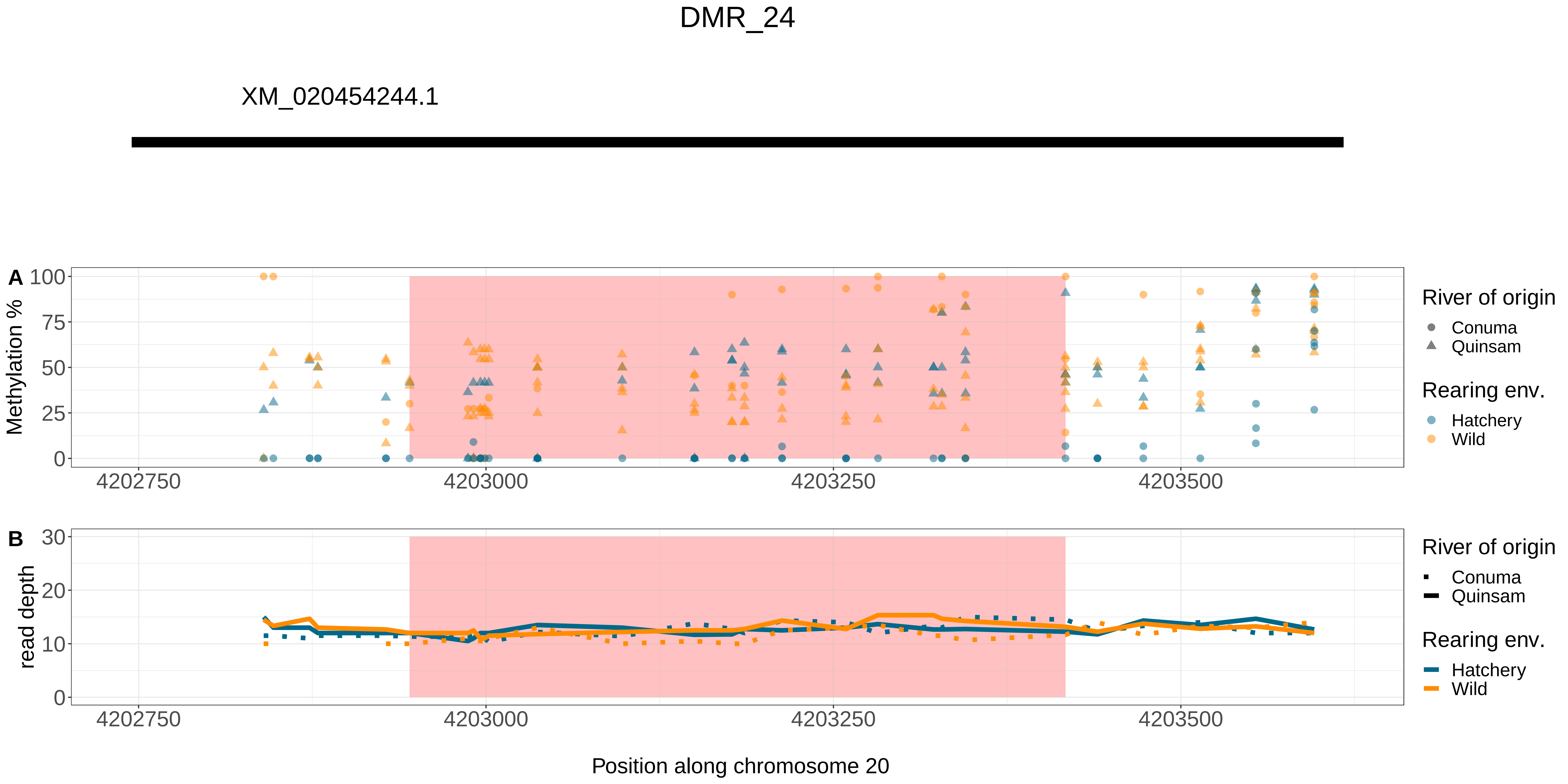

DMR_24
XM_020454244.1
A
B
Methylation %
100
75
50
25
0
read depth
30
20
10
0
4202750
4203000
4203250
4203500
Position along chromosome 20
River of origin
Conuma
Quinsam
Rearing env.
Hatchery
Wild

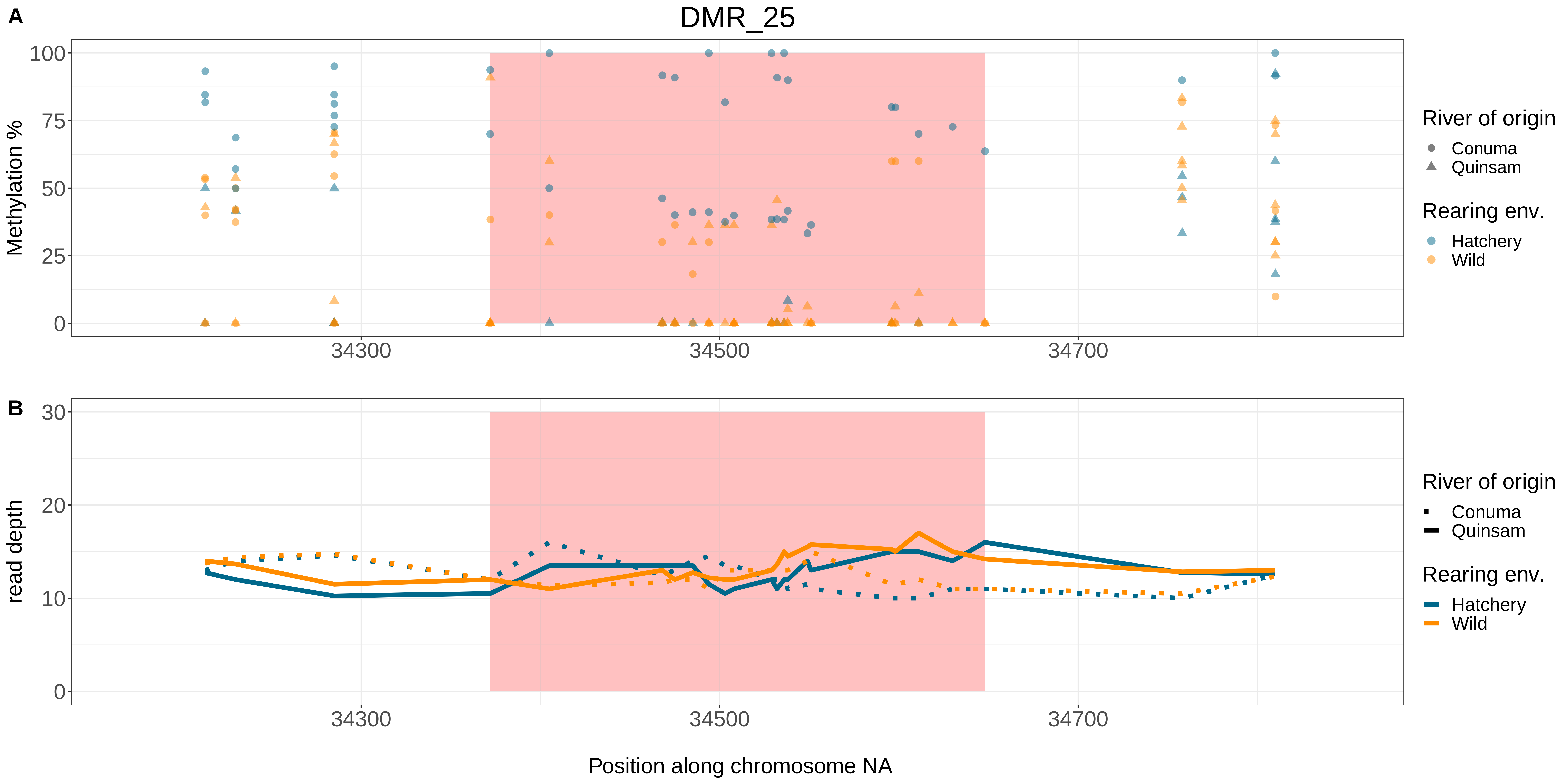
A
DMR_25
Methylation %
100
75
50
25
0
34300
34500
34700
River of origin
Conuma
Quinsam
Rearing env.
Hatchery
Wild
B
read depth
30
20
10
0
34300
34500
34700
River of origin
Conuma
Quinsam
Rearing env.
Hatchery
Wild
Position along chromosome NA

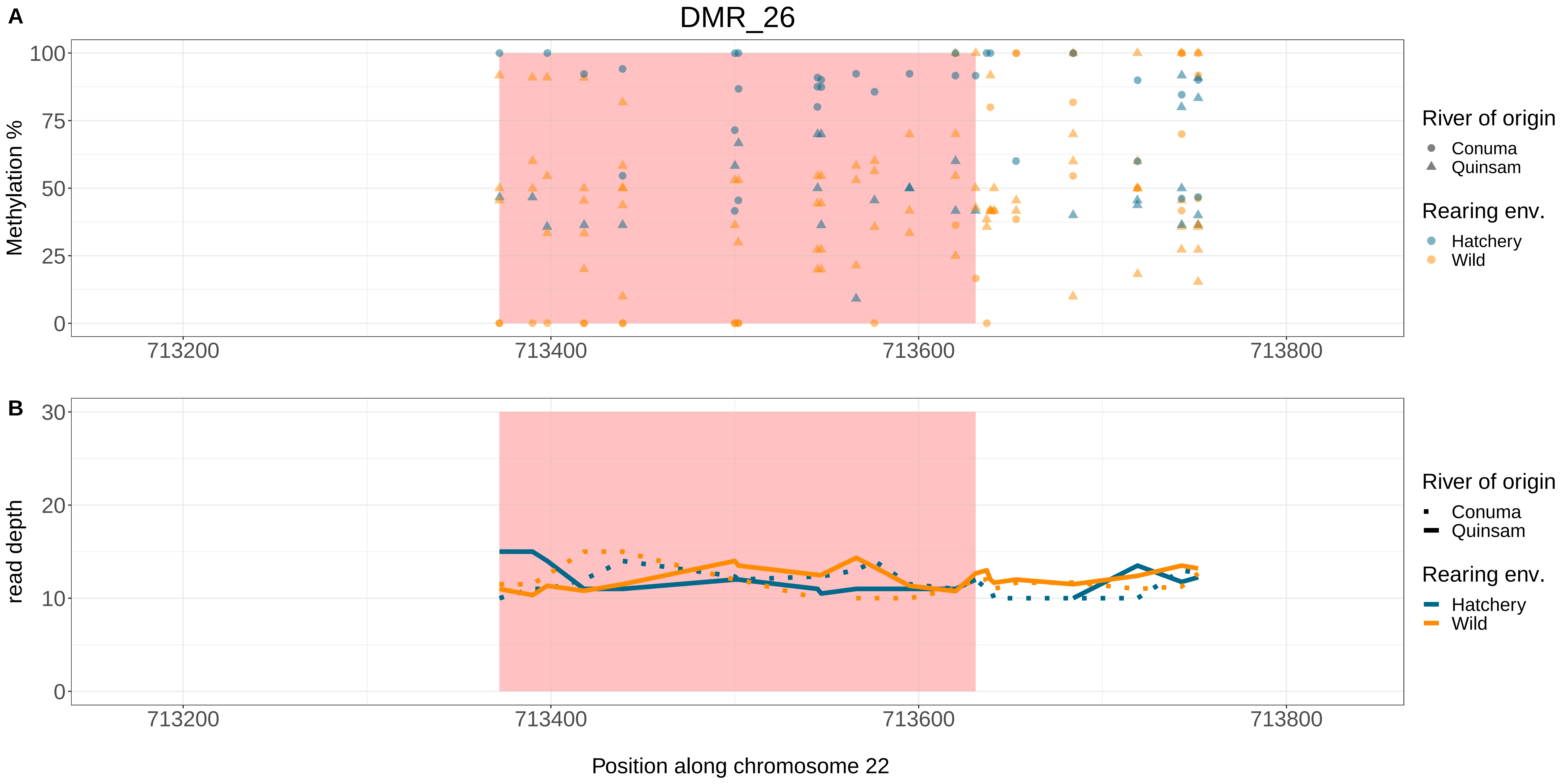
A
DMR_26
Methylation %
100
75
50
25
0
713200
713400
713600
713800
River of origin
Conuma
Quinsam
Rearing env.
Hatchery
Wild
B
read depth
30
20
10
0
713200
713400
713600
713800
River of origin
Conuma
Quinsam
Rearing env.
Hatchery
Wild
Position along chromosome 22

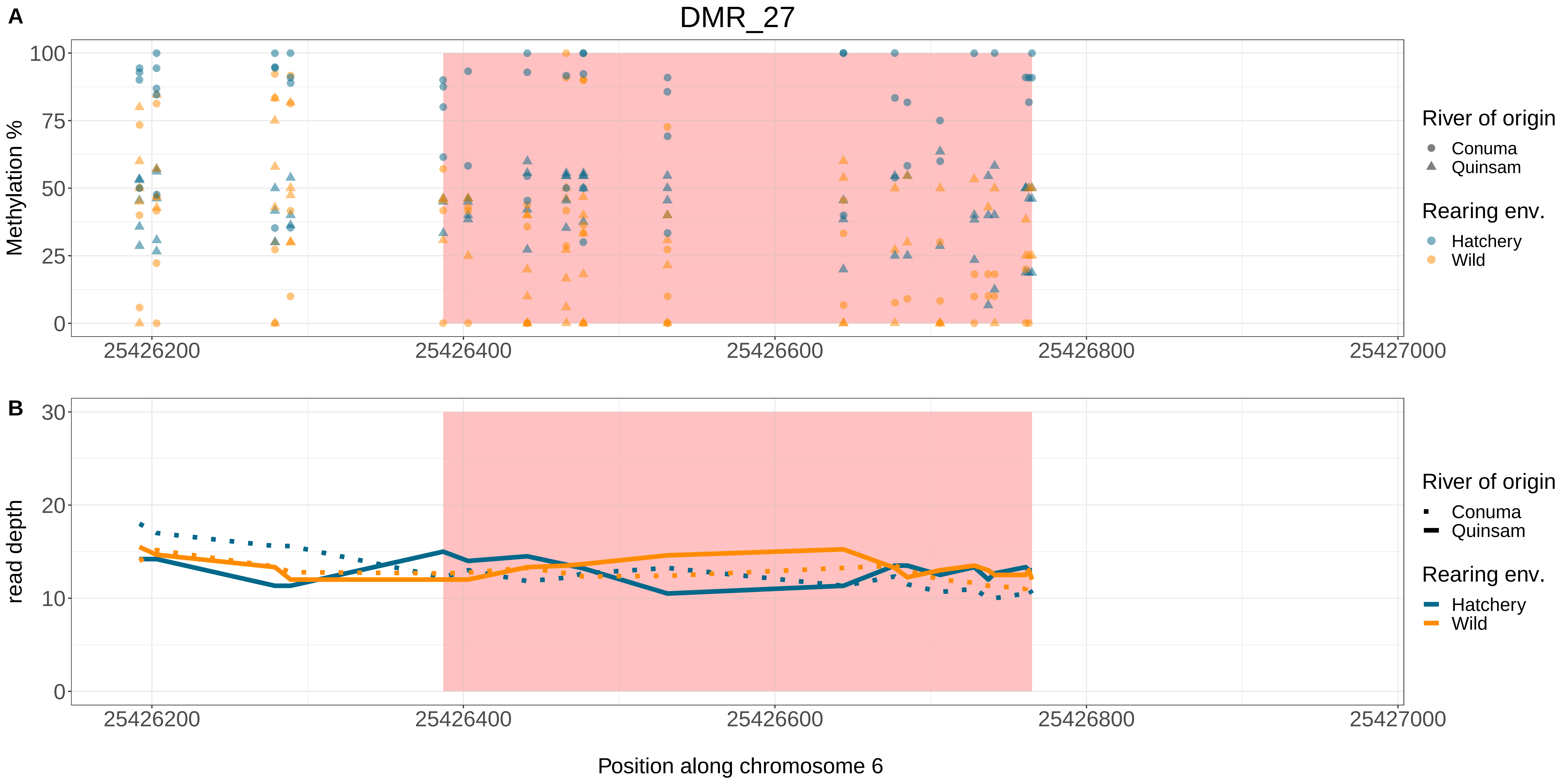

A
DMR_27
Methylation %
100
75
50
25
0
25426200
25426400
25426600
25426800
25427000
River of origin
Conuma
Quinsam
Rearing env.
Hatchery
Wild
B
read depth
30
20
10
0
Position along chromosome 6

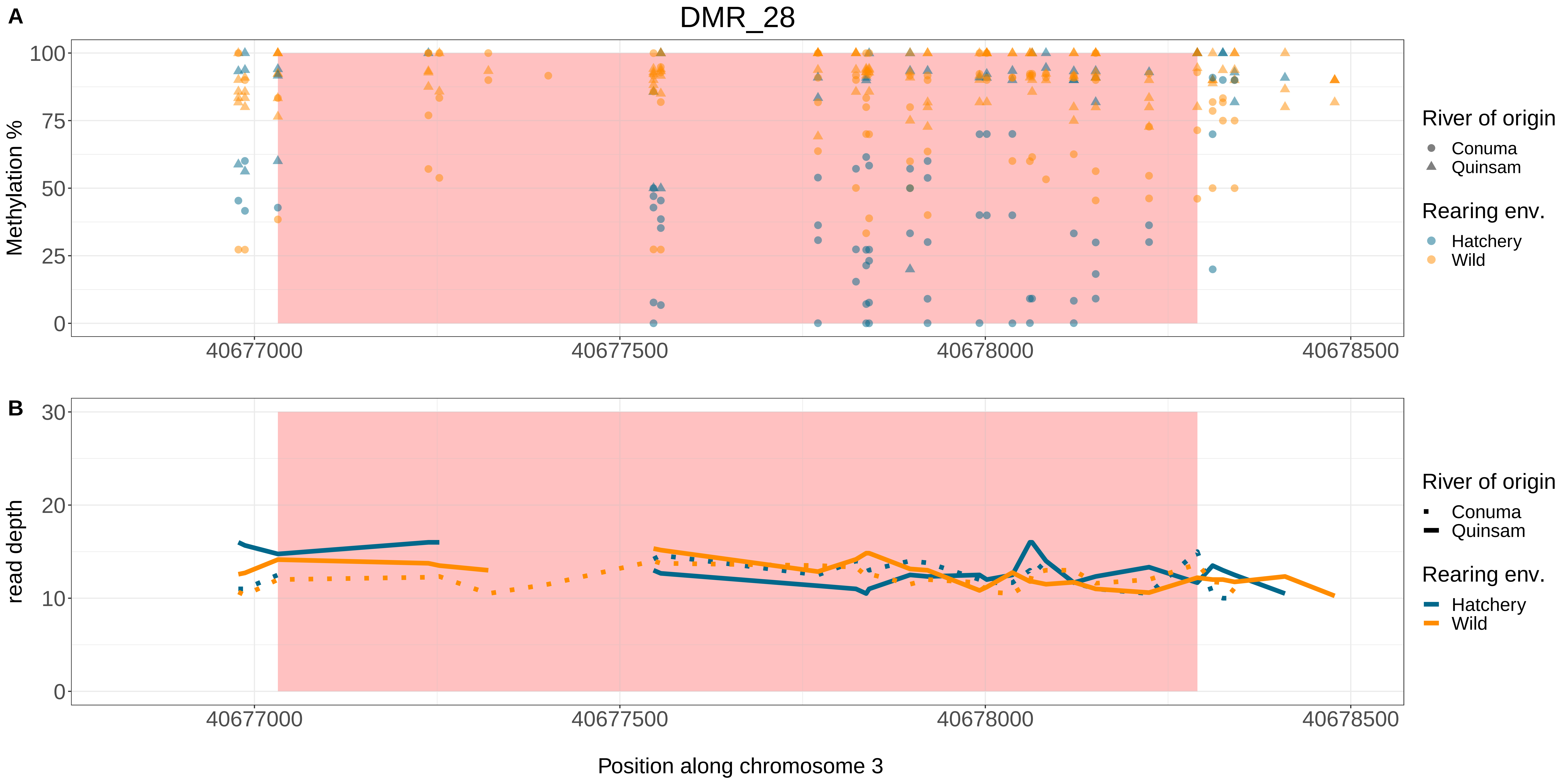

A
DMR_28
Methylation %
100
75
50
25
0
40677000
40677500
40678000
40678500
River of origin
Conuma
Quinsam
Rearing env.
Hatchery
Wild
B
read depth
30
20
10
0
40677000
40677500
40678000
40678500
River of origin
Conuma
Quinsam
Rearing env.
Hatchery
Wild
Position along chromosome 3

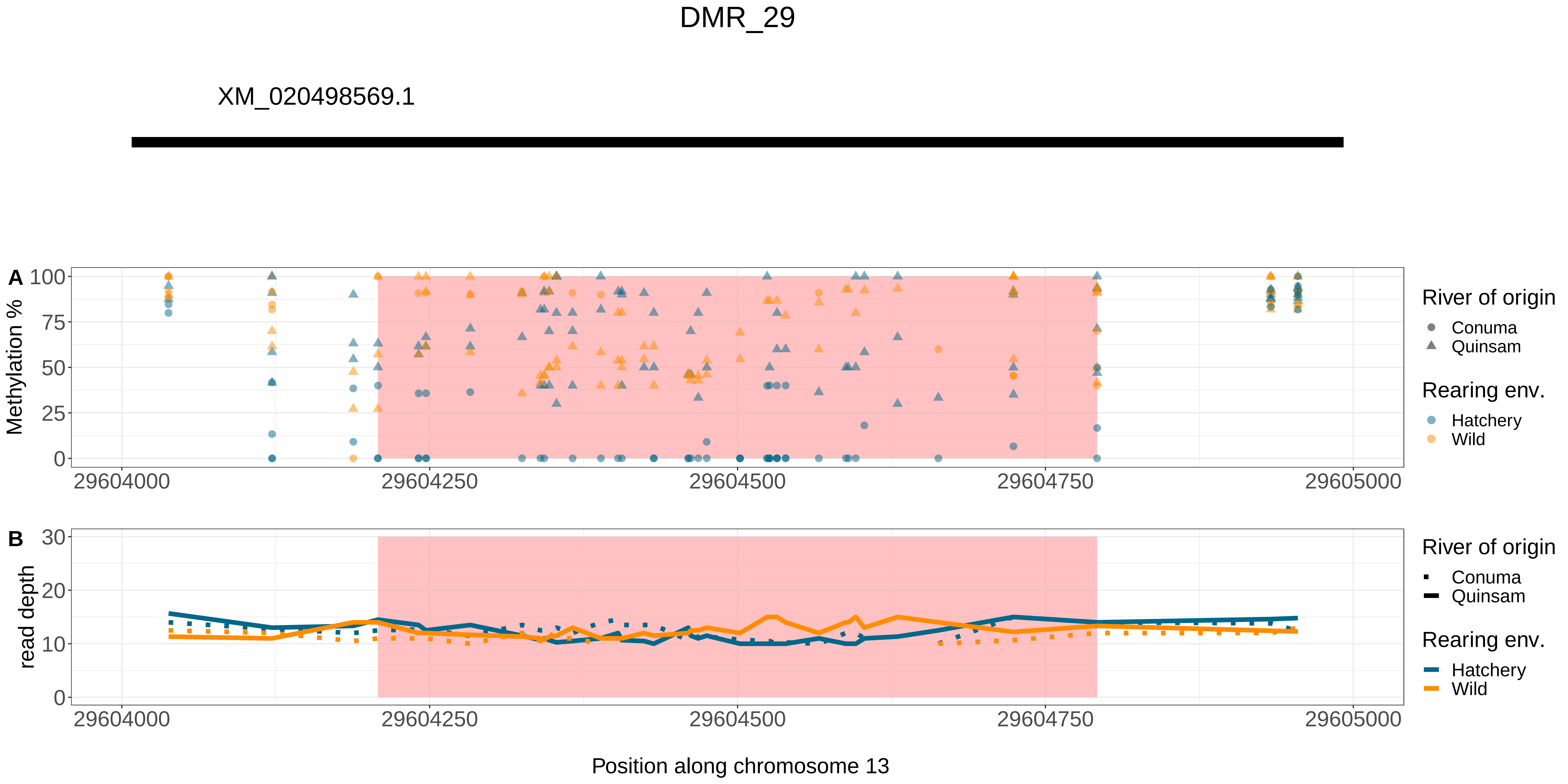

DMR_29
XM_020498569.1
A
Methylation %
100
75
50
25
0
29604000
29604250
29604500
29604750
29605000
River of origin
Conuma
Quinsam
Rearing env.
Hatchery
Wild
B
read depth
30
20
10
0
29604000
29604250
29604500
29604750
29605000
River of origin
Conuma
Quinsam
Rearing env.
Hatchery
Wild
Position along chromosome 13

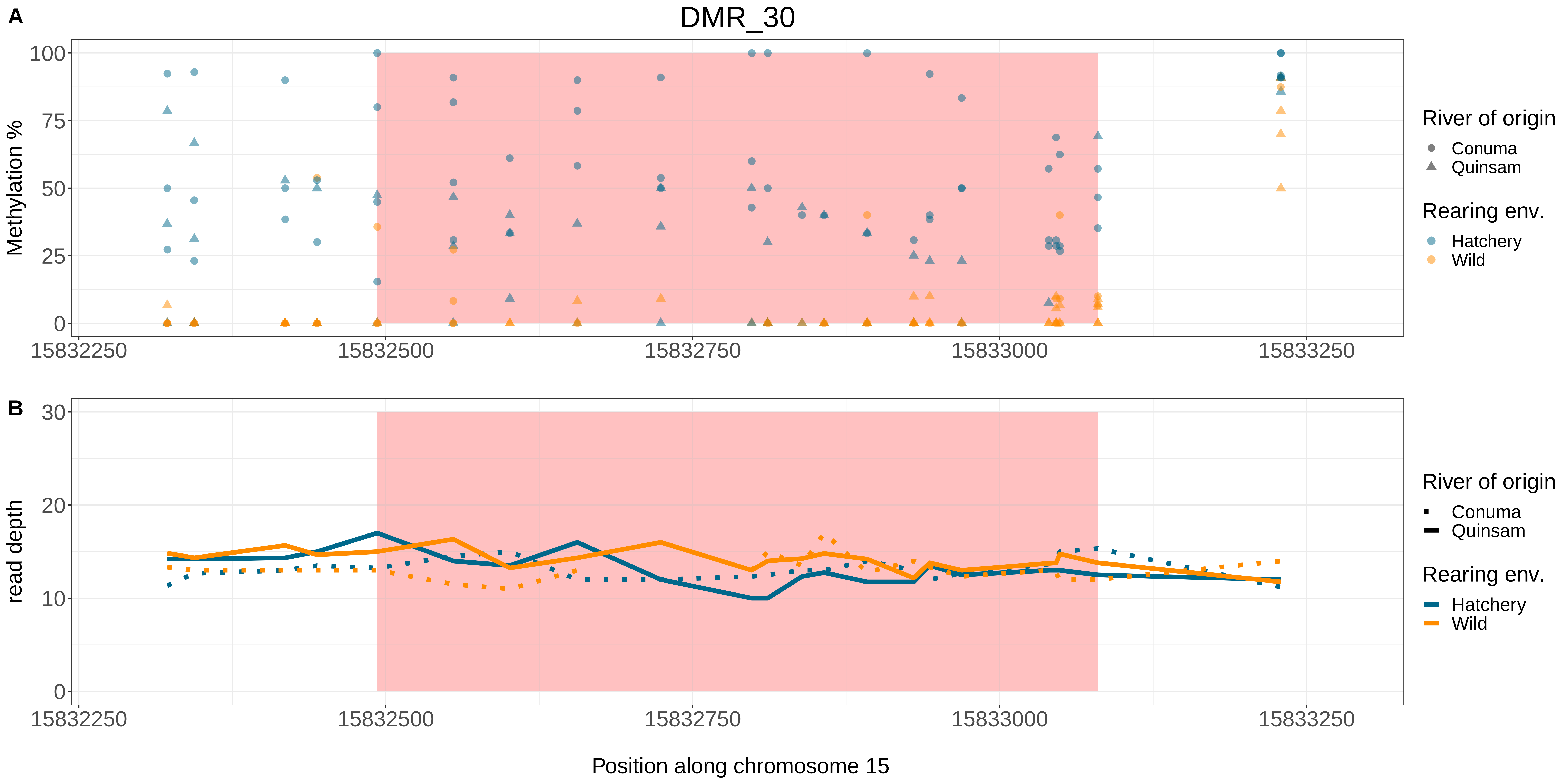
A
DMR_30
Methylation %
100
75
50
25
0
15832250
15832500
15832750
15833000
15833250
River of origin
Conuma
Quinsam
Rearing env.
Hatchery
Wild
B
read depth
30
20
10
0
15832250
15832500
15832750
15833000
15833250
River of origin
Conuma
Quinsam
Rearing env.
Hatchery
Wild
Position along chromosome 15

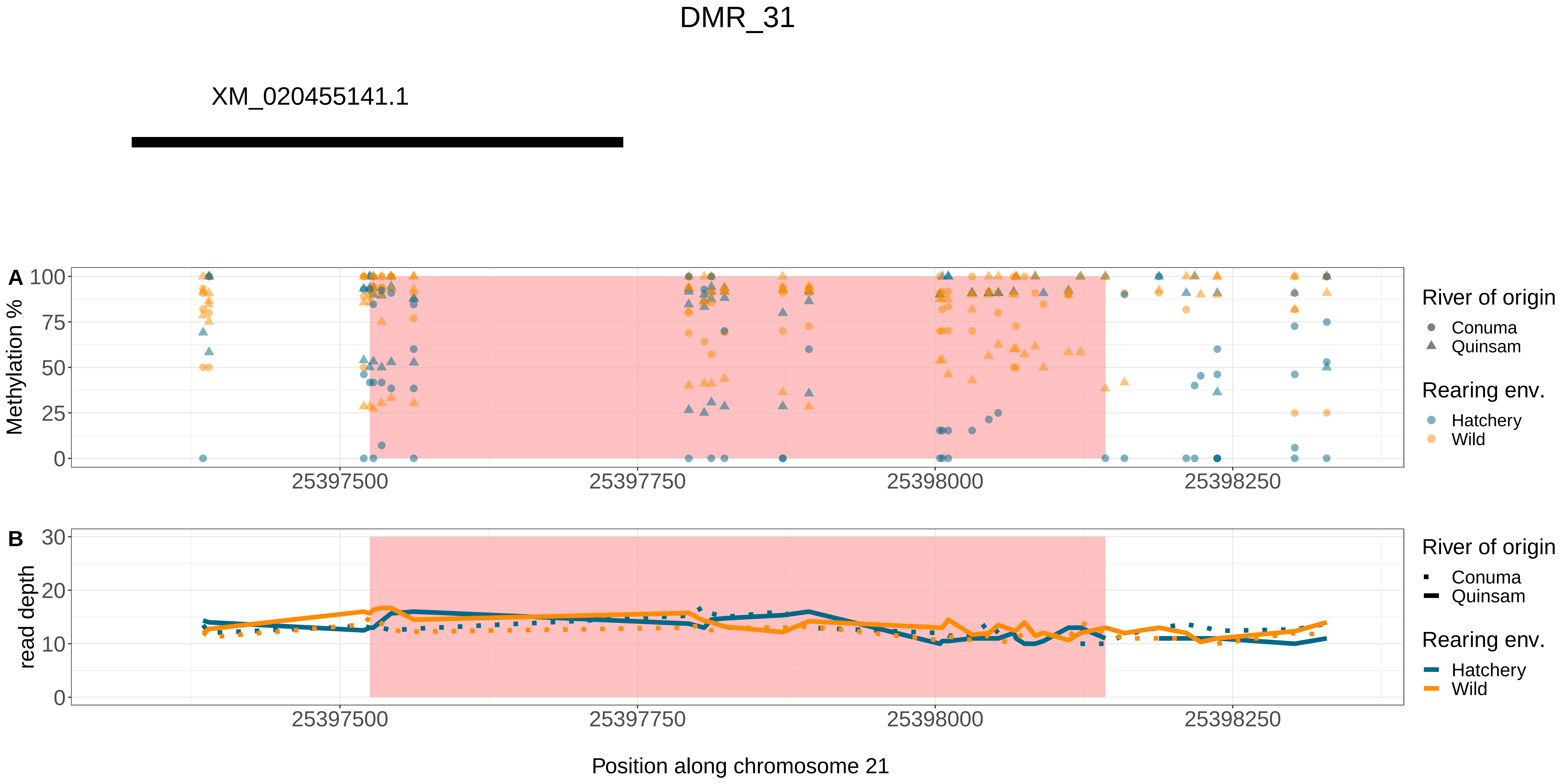

DMR_31
XM_020455141.1
A
B
Methylation %
read depth
100
75
50
25
0
30
20
10
0
25397500
25397750
25398000
25398250
Position along chromosome 21
River of origin
Conuma
Quinsam
Rearing env.
Hatchery
Wild

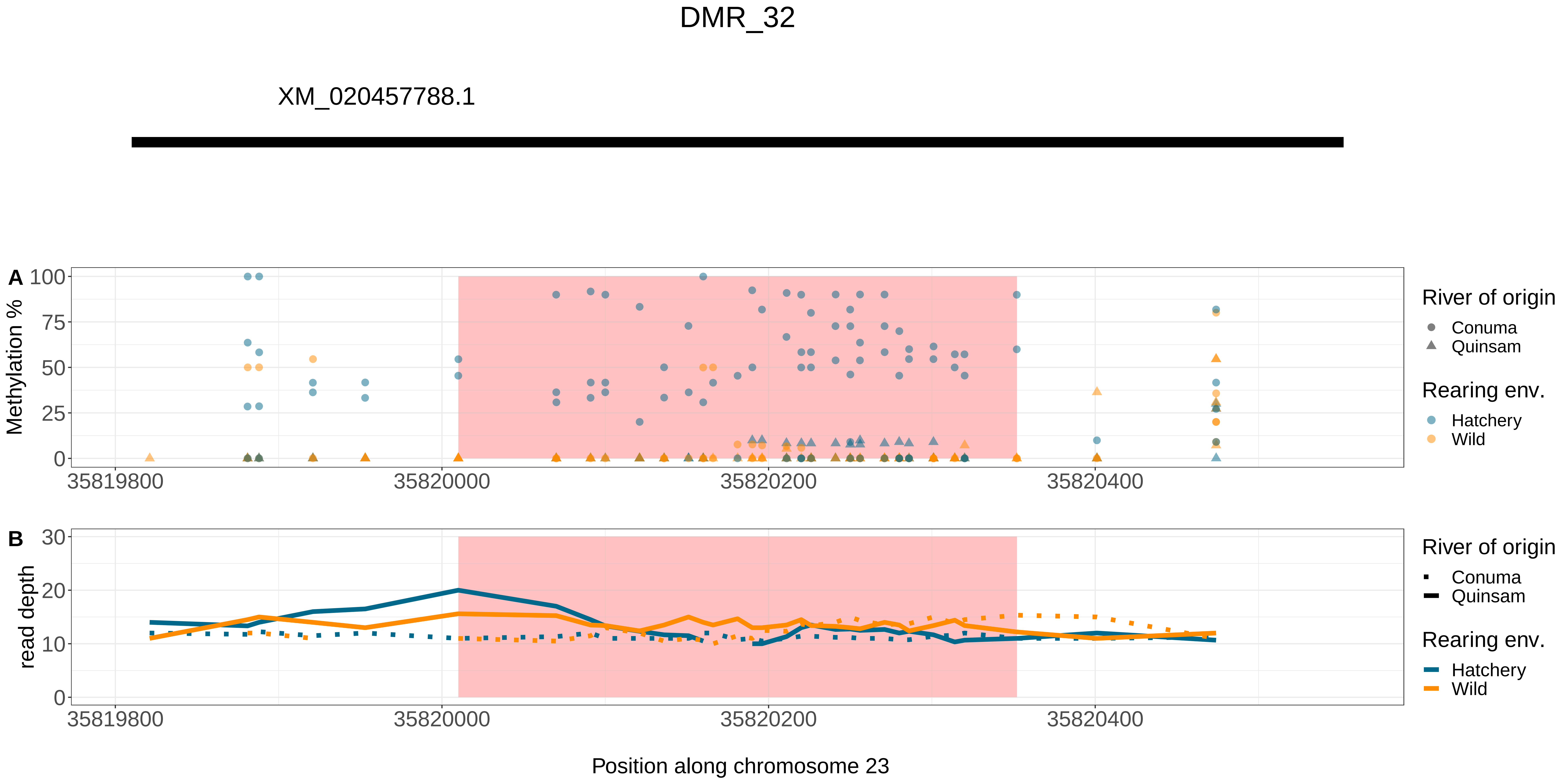

DMR_32
XM_020457788.1
A
Methylation %
100
75
50
25
0
35819800
35820000
35820200
35820400
River of origin
Conuma
Quinsam
Rearing env.
Hatchery
Wild
B
read depth
30
20
10
0
35819800
35820000
35820200
35820400
River of origin
Conuma
Quinsam
Rearing env.
Hatchery
Wild
Position along chromosome 23

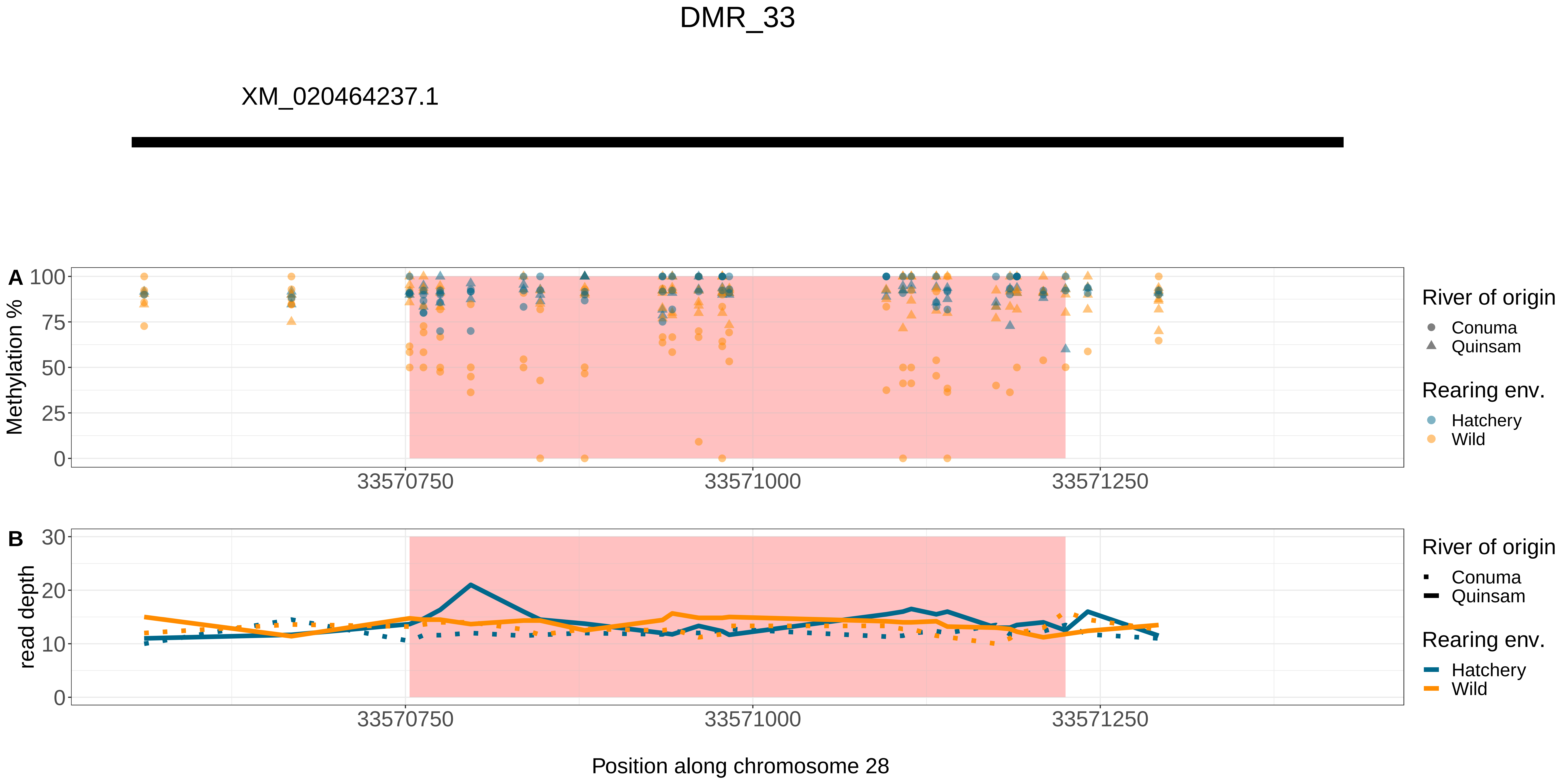
DMR_33
XM_020464237.1
A
Methylation %
100
75
50
25
0
33570750
33571000
33571250
River of origin
Conuma
Quinsam
Rearing env.
Hatchery
Wild
B
read depth
30
20
10
0
33570750
33571000
33571250
River of origin
Conuma
Quinsam
Rearing env.
Hatchery
Wild
Position along chromosome 28

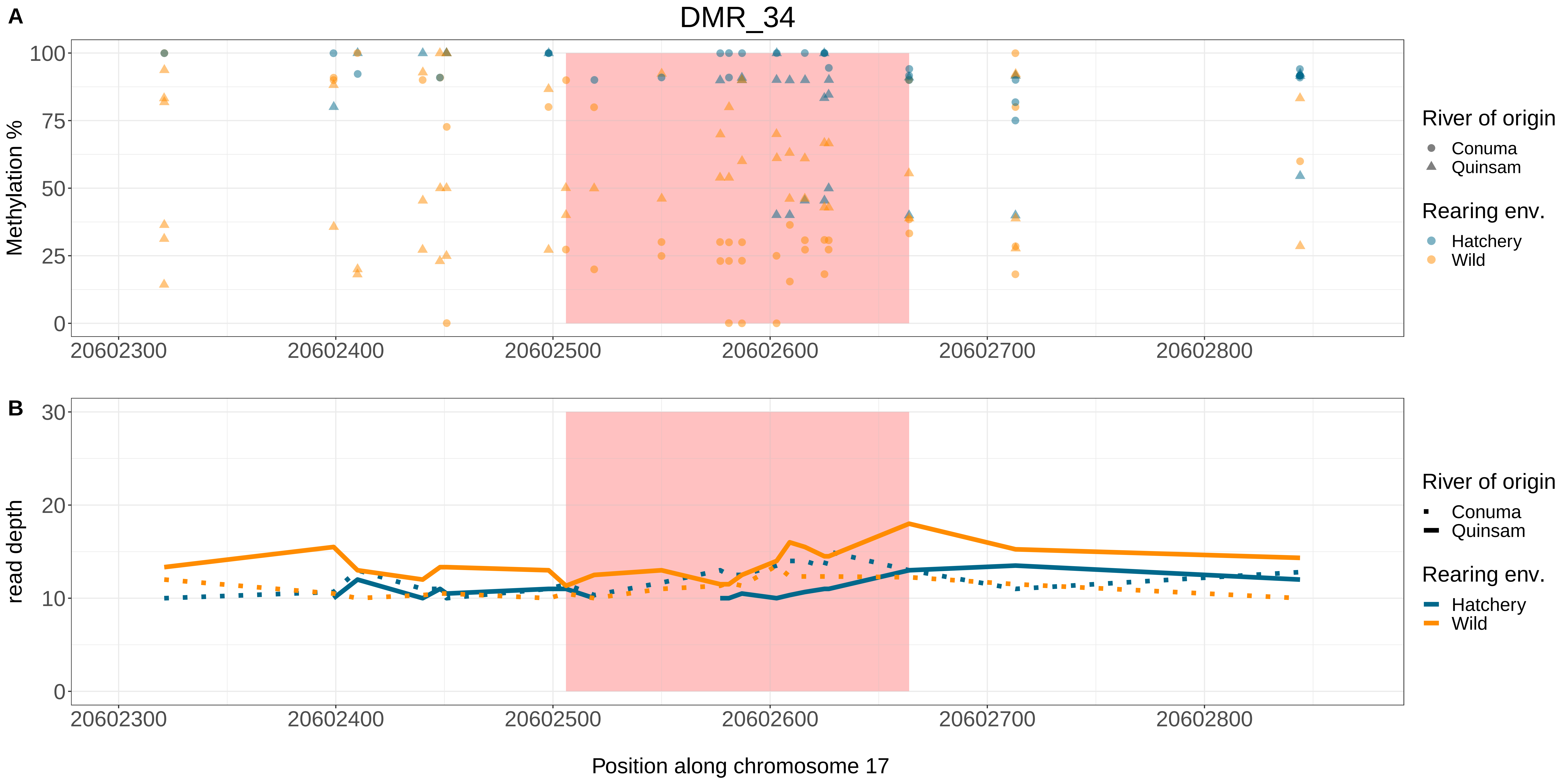

A
DMR_34
Methylation %
100
75
50
25
0
20602300
20602400
20602500
20602600
20602700
20602800
River of origin
Conuma
Quinsam
Rearing env.
Hatchery
Wild
B
read depth
30
20
10
0
20602300
20602400
20602500
20602600
20602700
20602800
River of origin
Conuma
Quinsam
Rearing env.
Hatchery
Wild
Position along chromosome 17

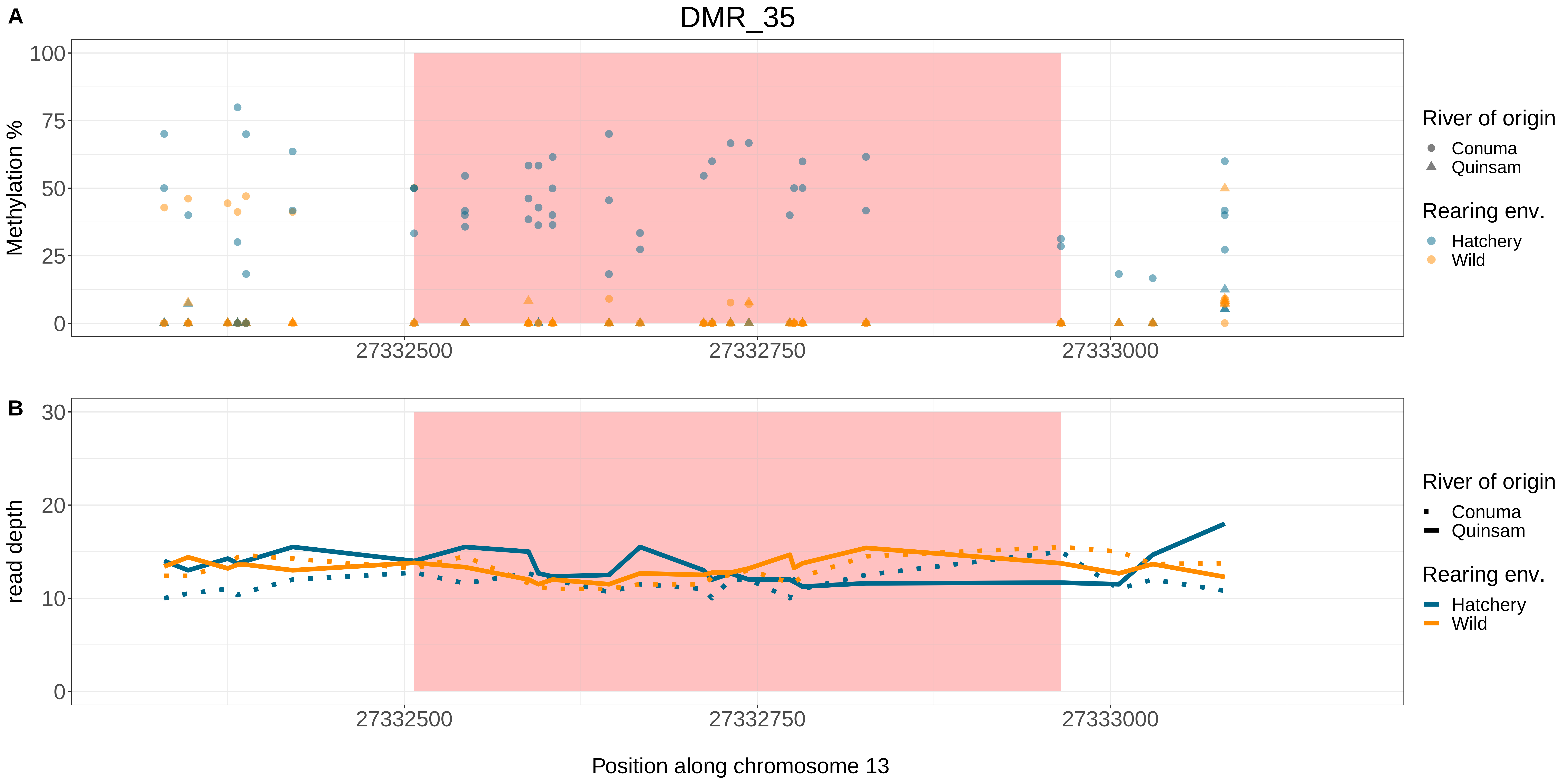

A
DMR_35
Methylation %
100
75
50
25
0
27332500
27332750
27333000
River of origin
Conuma
Quinsam
Rearing env.
Hatchery
Wild
B
read depth
30
20
10
0
27332500
27332750
27333000
River of origin
Conuma
Quinsam
Rearing env.
Hatchery
Wild
Position along chromosome 13

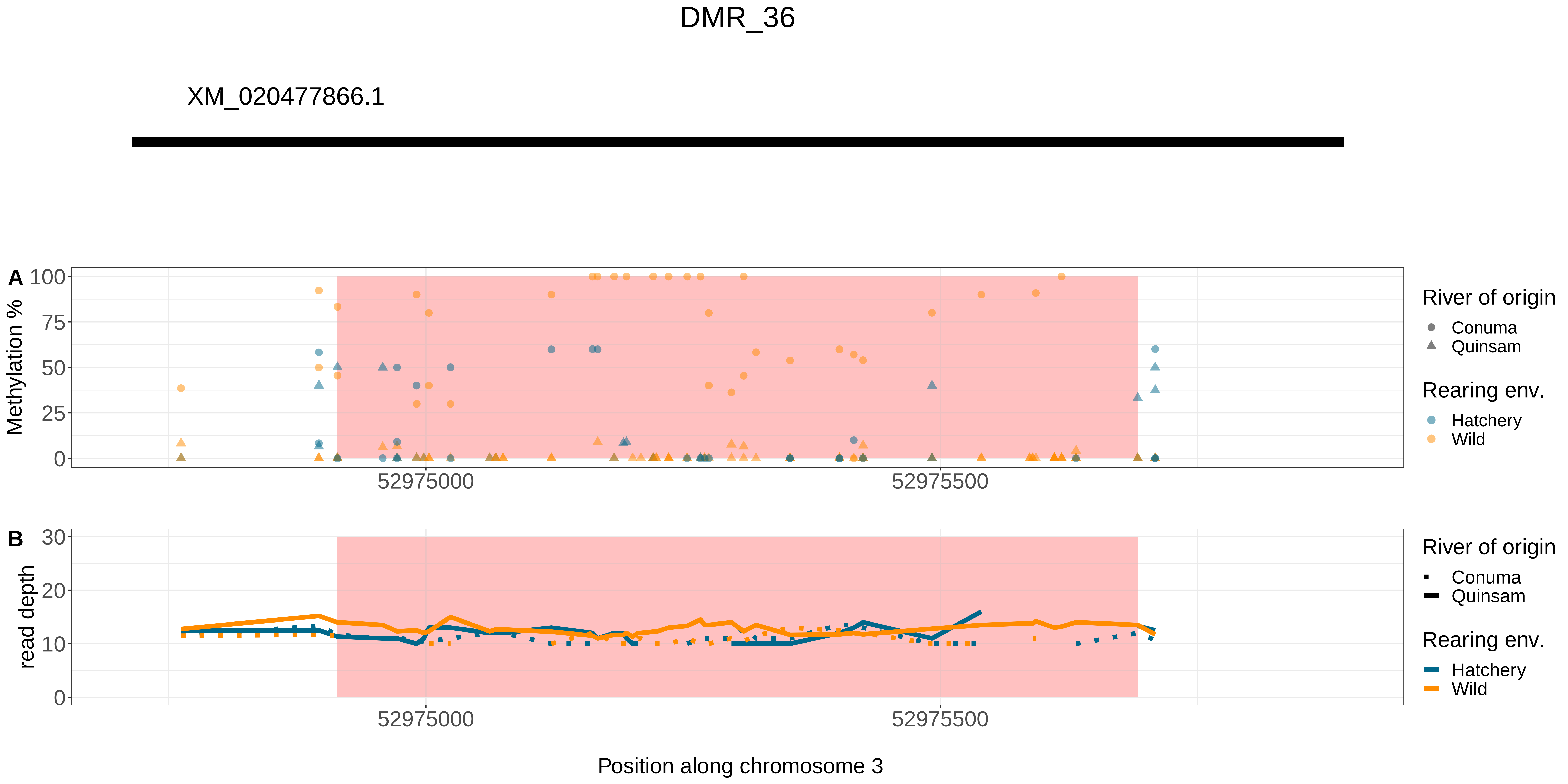

DMR_36
XM_020477866.1
A
B
Methylation %
100
75
50
25
0
52975000
52975500
read depth
30
20
10
0
River of origin
Conuma
Quinsam
Rearing env.
Hatchery
Wild
Position along chromosome 3

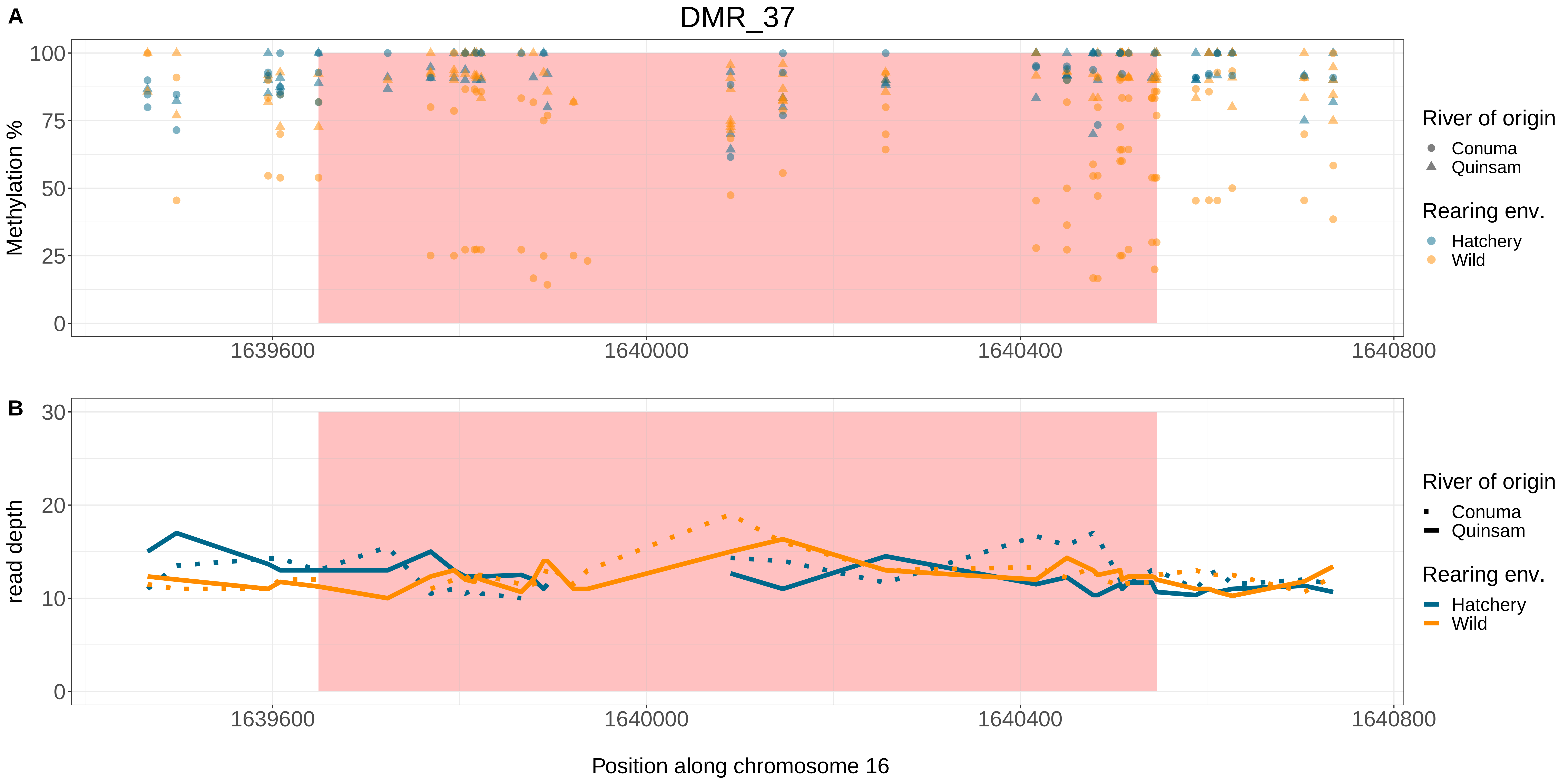
A
DMR_37
Methylation %
100
75
50
25
0
1639600
1640000
1640400
1640800
River of origin
Conuma
Quinsam
Rearing env.
Hatchery
Wild
B
read depth
30
20
10
0
1639600
1640000
1640400
1640800
River of origin
Conuma
Quinsam
Rearing env.
Hatchery
Wild
Position along chromosome 16

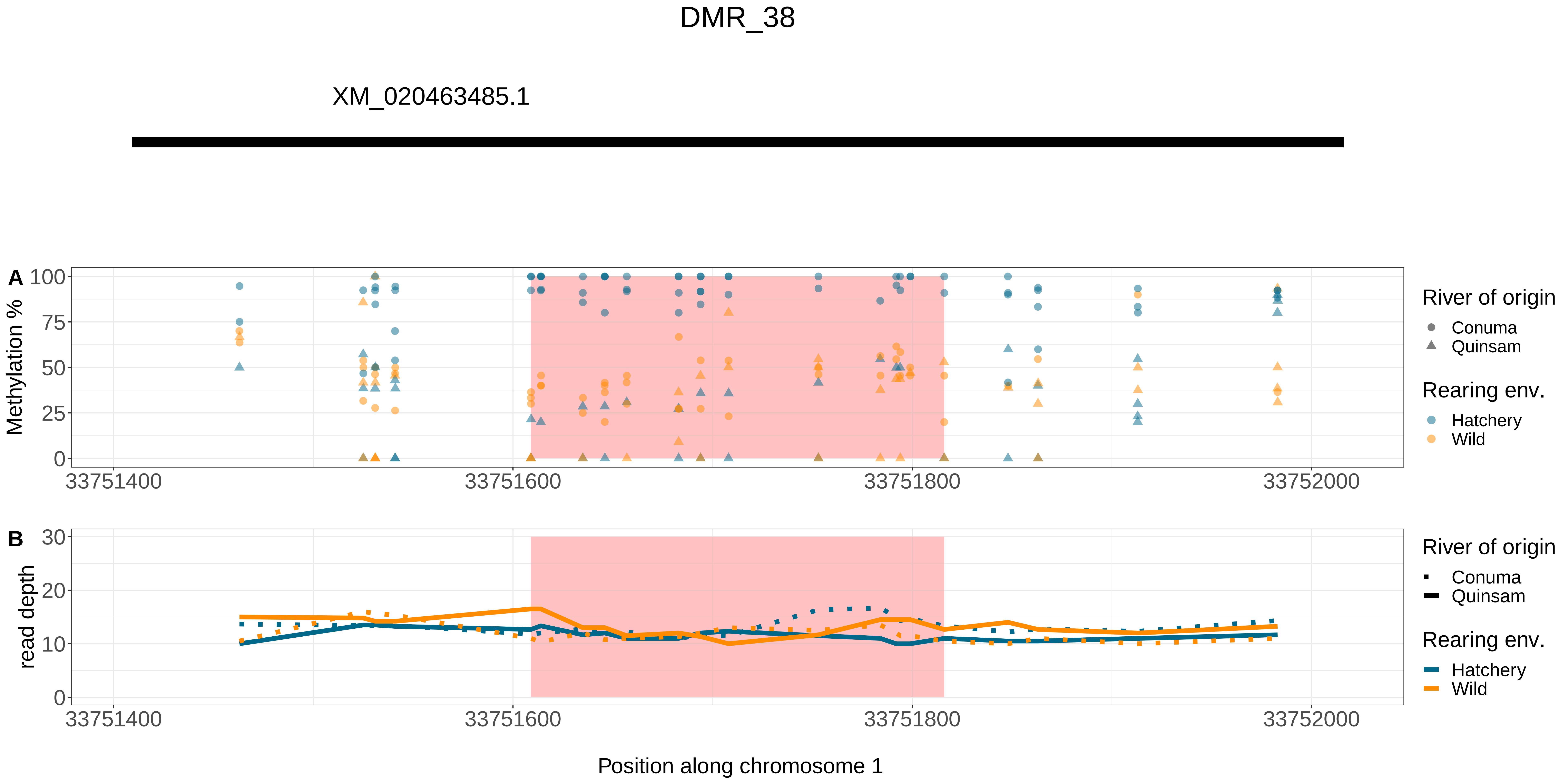

DMR_38
XM_020463485.1
A
Methylation %
100
75
50
25
0
33751400
33751600
33751800
33752000
River of origin
Conuma
Quinsam
Rearing env.
Hatchery
Wild
B
read depth
30
20
10
0
33751400
33751600
33751800
33752000
River of origin
Conuma
Quinsam
Rearing env.
Hatchery
Wild
Position along chromosome 1

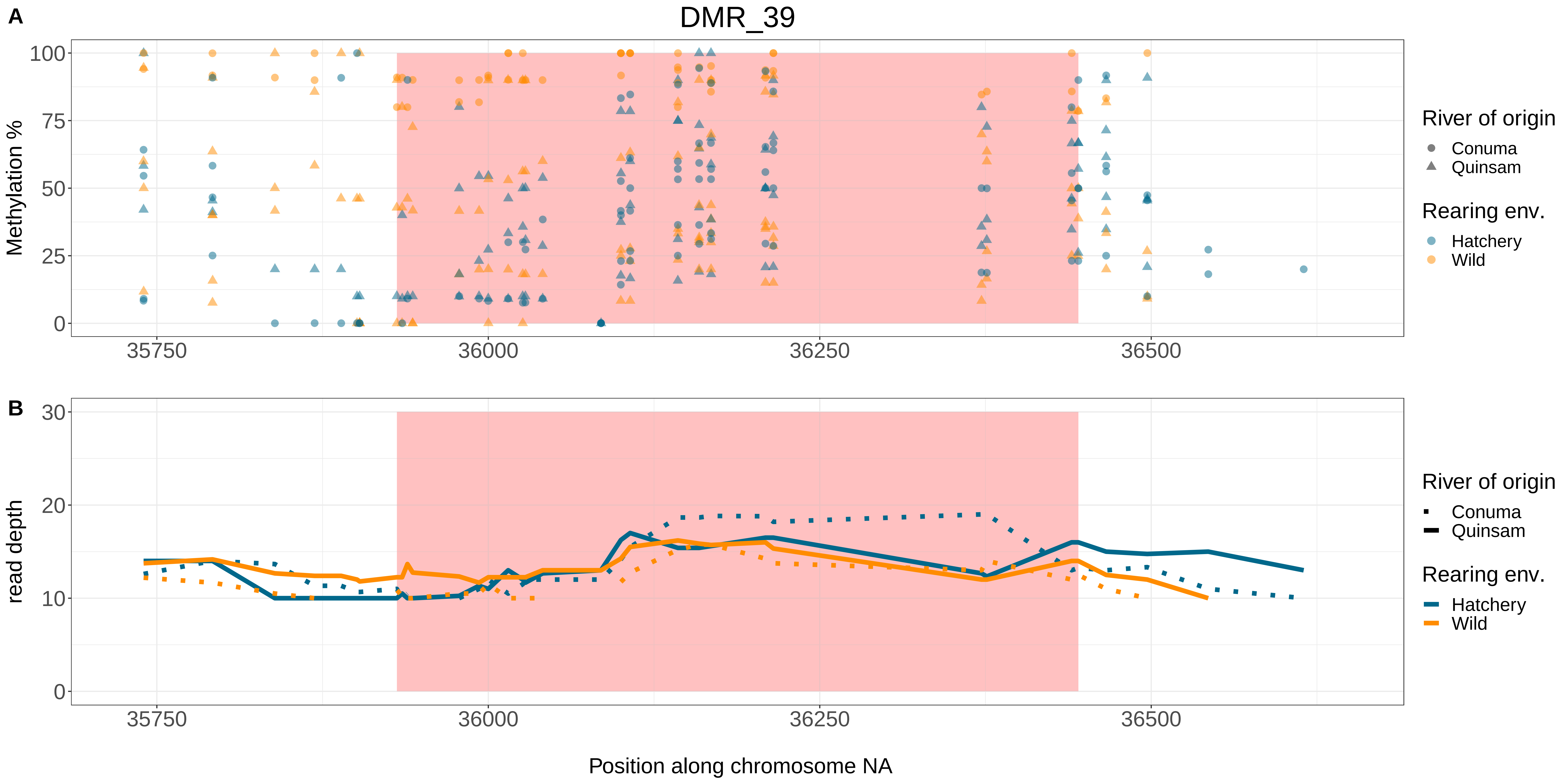

A
DMR_39
Methylation %
100
75
50
25
0
35750
36000
36250
36500
River of origin
Conuma
Quinsam
Rearing env.
Hatchery
Wild
B
read depth
30
20
10
0
35750
36000
36250
36500
River of origin
Conuma
Quinsam
Rearing env.
Hatchery
Wild
Position along chromosome NA

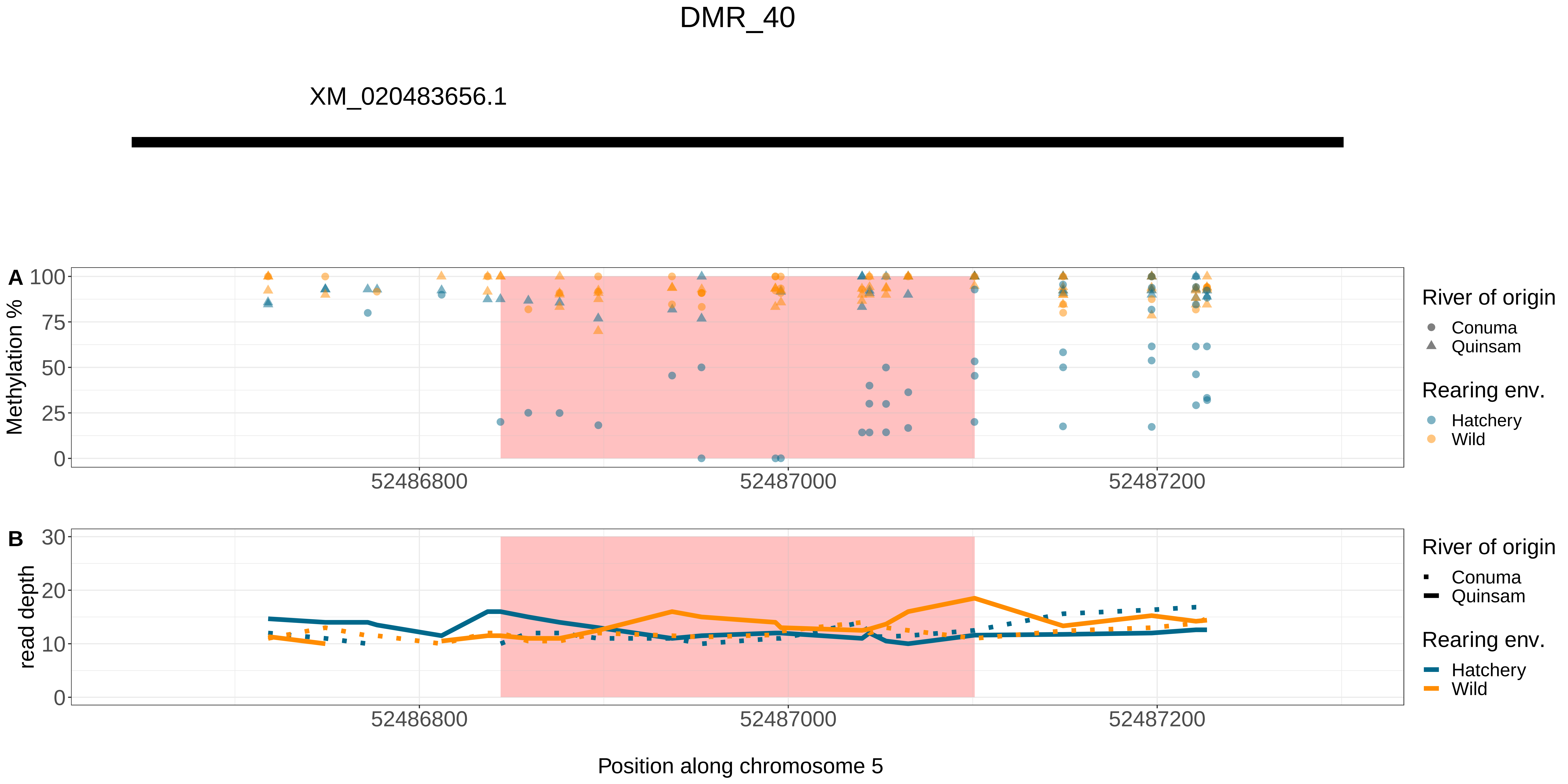

DMR_40
XM_020483656.1
A
Methylation %
100
75
50
25
0
52486800
52487000
52487200
River of origin
Conuma
Quinsam
Rearing env.
Hatchery
Wild
B
read depth
30
20
10
0
52486800
52487000
52487200
River of origin
Conuma
Quinsam
Rearing env.
Hatchery
Wild
Position along chromosome 5

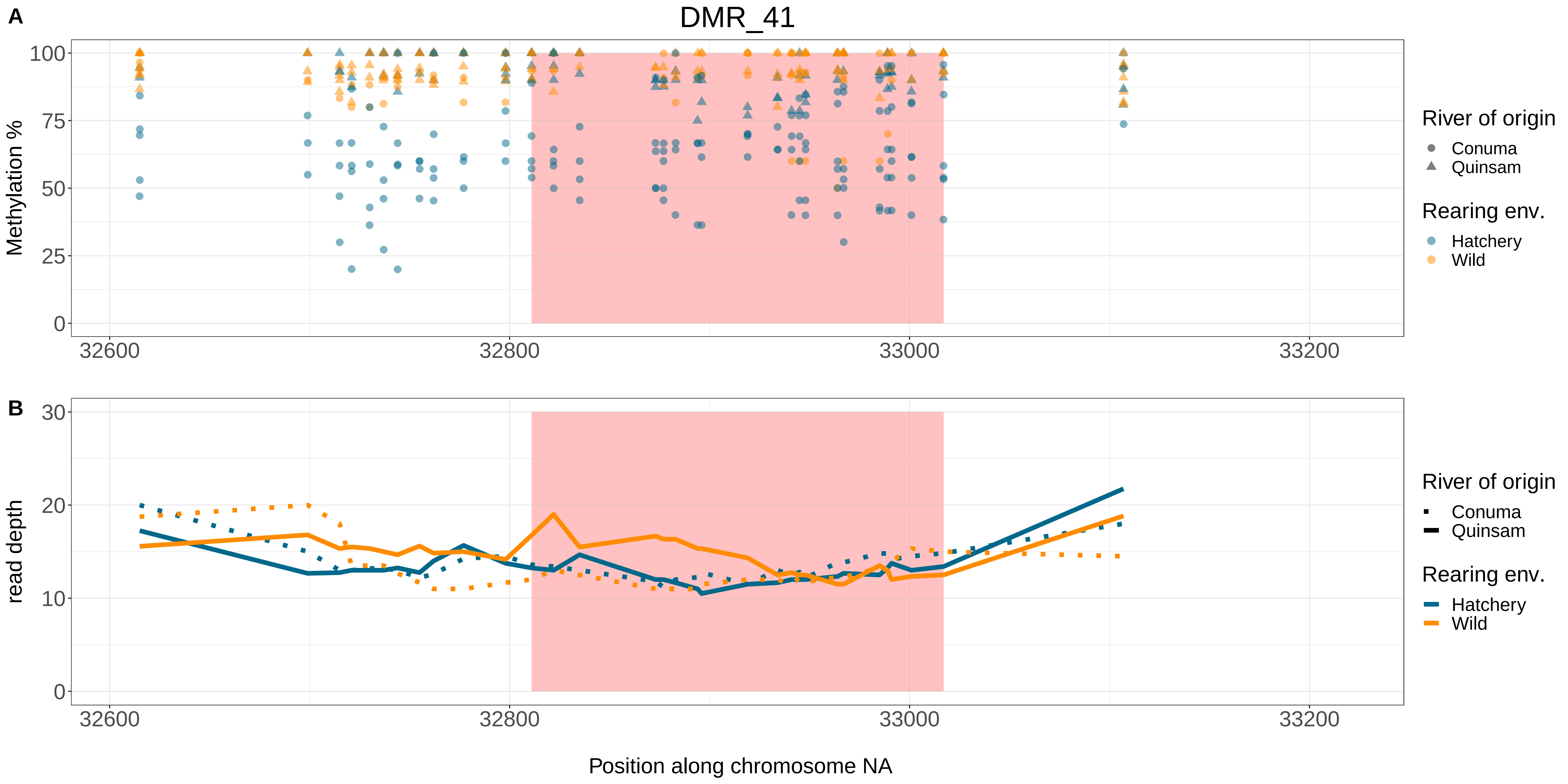

A
DMR_41
Methylation %
100
75
50
25
0
32600
32800
33000
33200
River of origin
Conuma
Quinsam
Rearing env.
Hatchery
Wild
B
read depth
30
20
10
0
32600
32800
33000
33200
River of origin
Conuma
Quinsam
Rearing env.
Hatchery
Wild
Position along chromosome NA

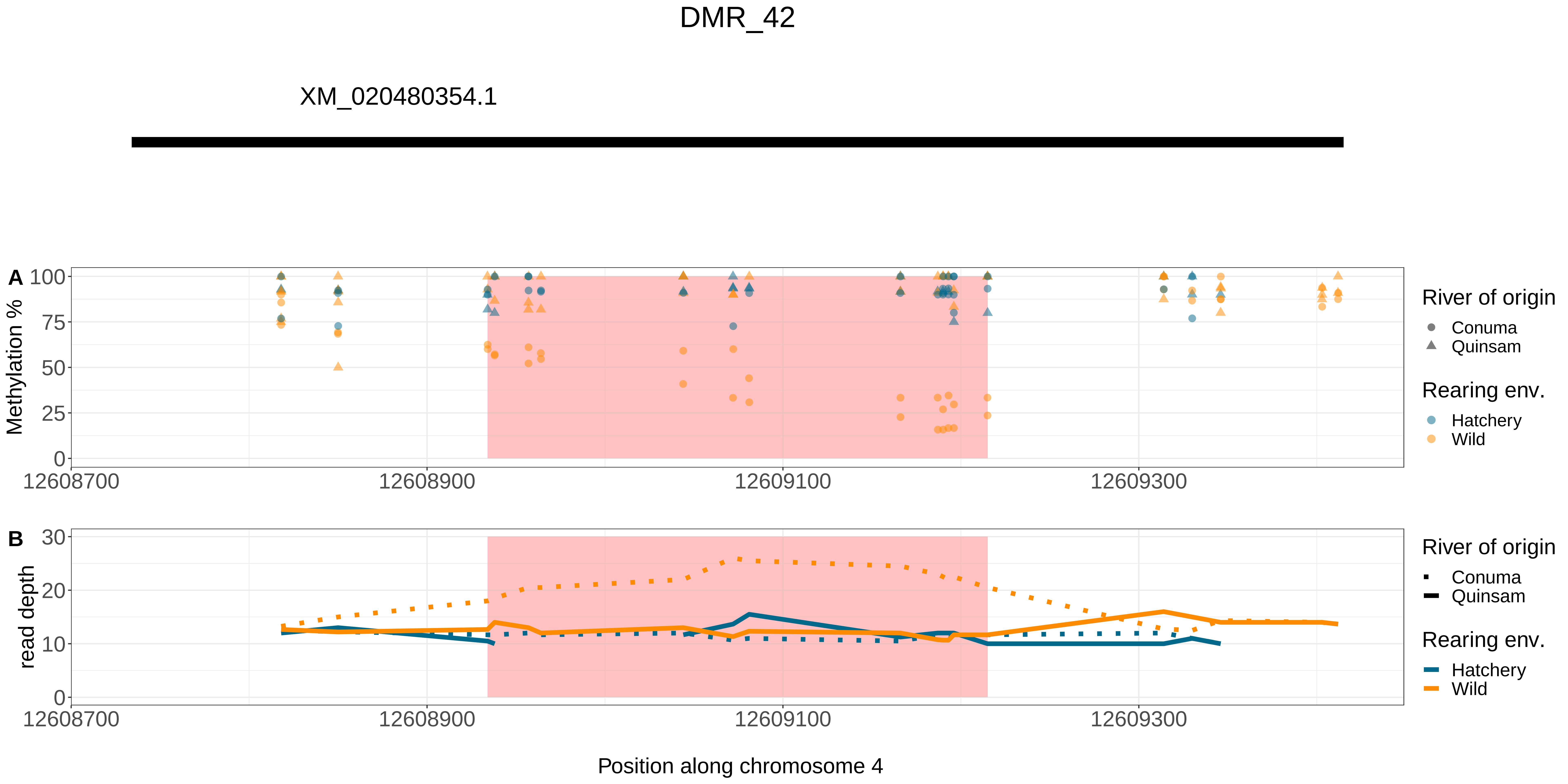
DMR_42
XM_020480354.1
A
Methylation %
100
75
50
25
0
12608700
12608900
12609100
12609300
River of origin
Conuma
Quinsam
Rearing env.
Hatchery
Wild
B
read depth
30
20
10
0
Position along chromosome 4

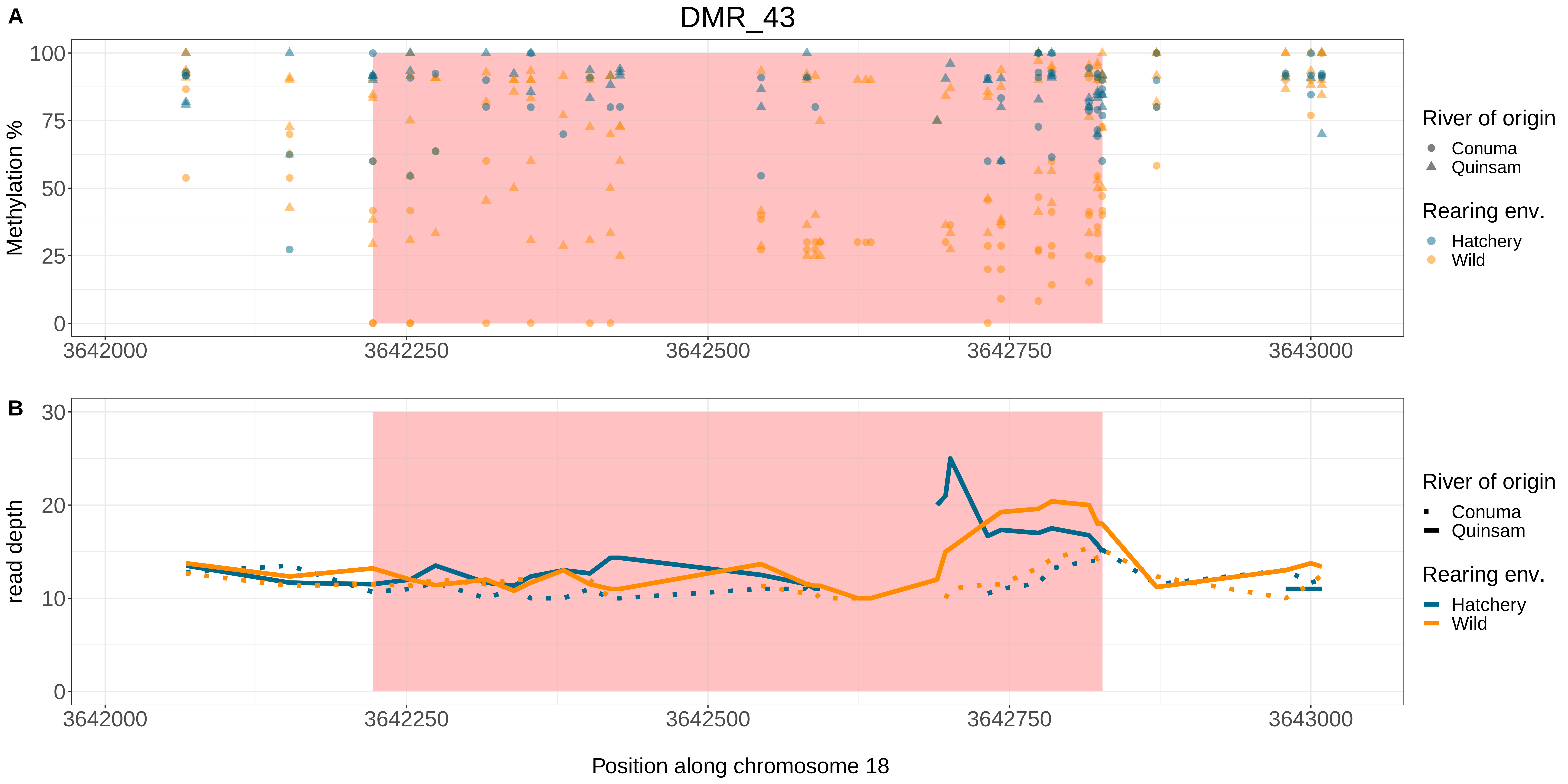

A
DMR_43
Methylation %
100
75
50
25
0
3642000
3642250
3642500
3642750
3643000
River of origin
Conuma
Quinsam
Rearing env.
Hatchery
Wild
B
read depth
30
20
10
0
3642000
3642250
3642500
3642750
3643000
River of origin
Conuma
Quinsam
Rearing env.
Hatchery
Wild
Position along chromosome 18

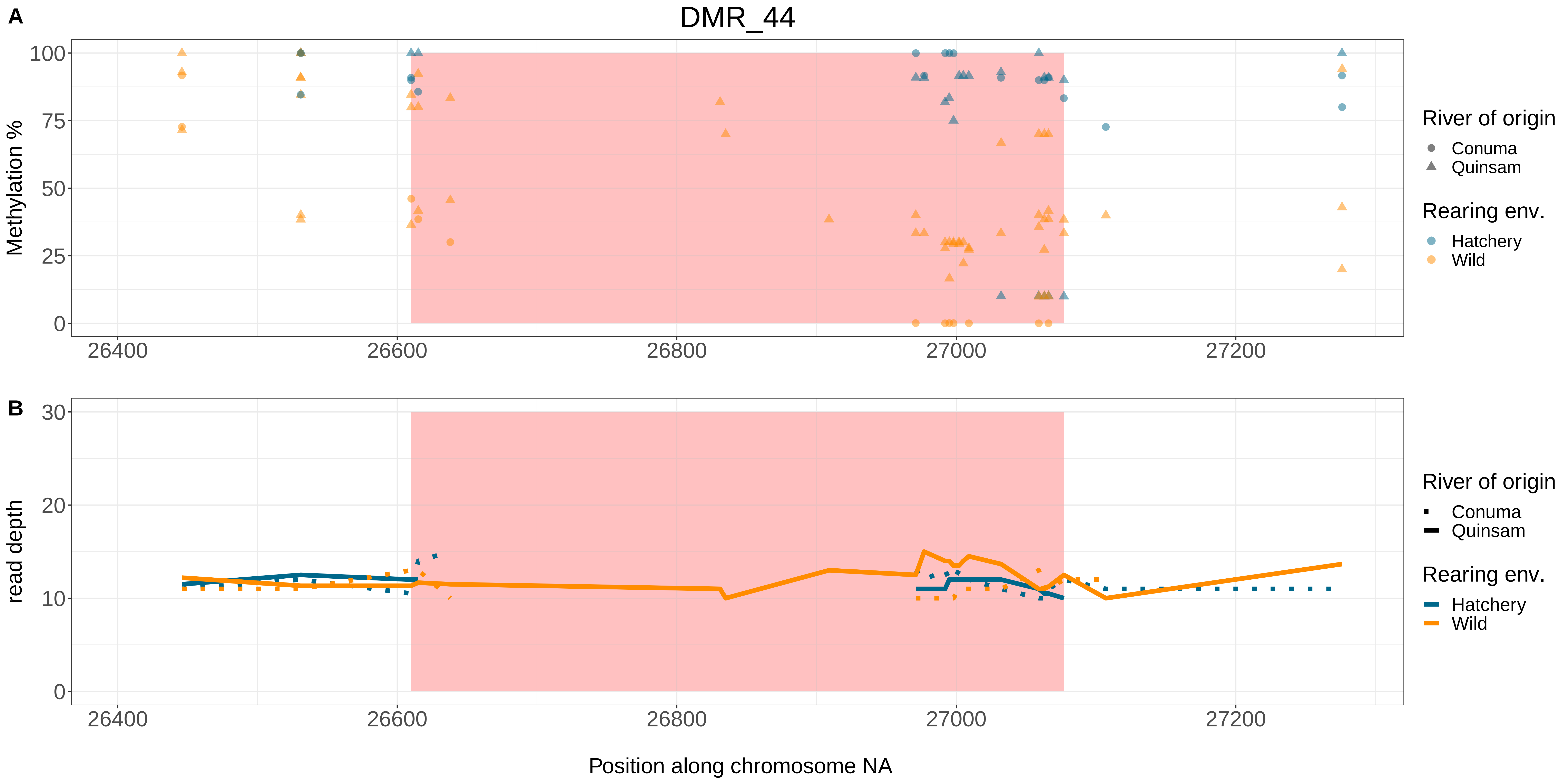

A
DMR_44
Methylation %
100
75
50
25
0
26400
26600
26800
27000
27200
River of origin
Conuma
Quinsam
Rearing env.
Hatchery
Wild
B
read depth
30
20
10
0
26400
26600
26800
27000
27200
River of origin
Conuma
Quinsam
Rearing env.
Hatchery
Wild
Position along chromosome NA

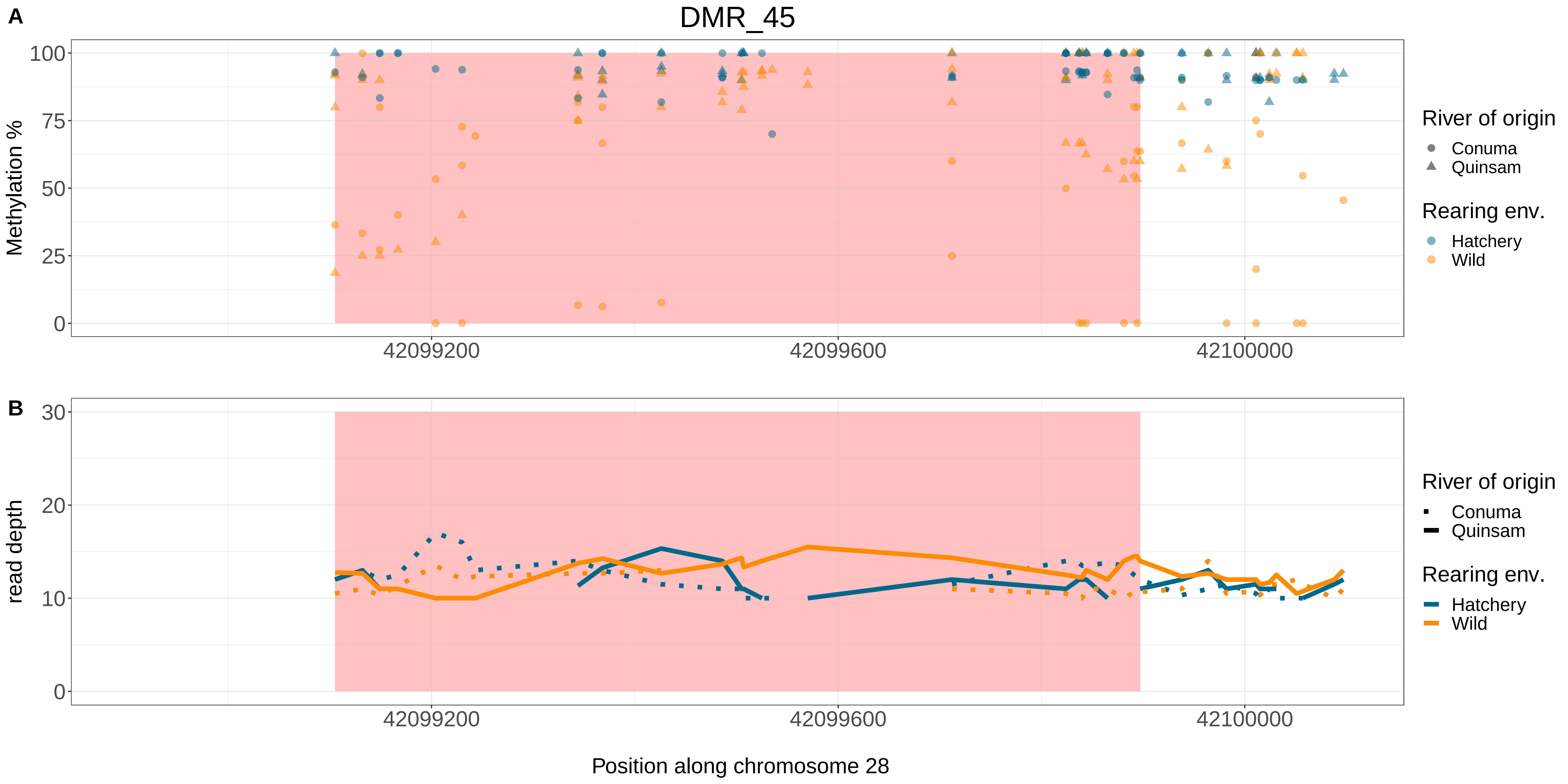
A
DMR_45
Methylation %
100
75
50
25
0
42099200
42099600
42100000
River of origin
Conuma
Quinsam
Rearing env.
Hatchery
Wild
B
read depth
30
20
10
0
42099200
42099600
42100000
River of origin
Conuma
Quinsam
Rearing env.
Hatchery
Wild
Position along chromosome 28

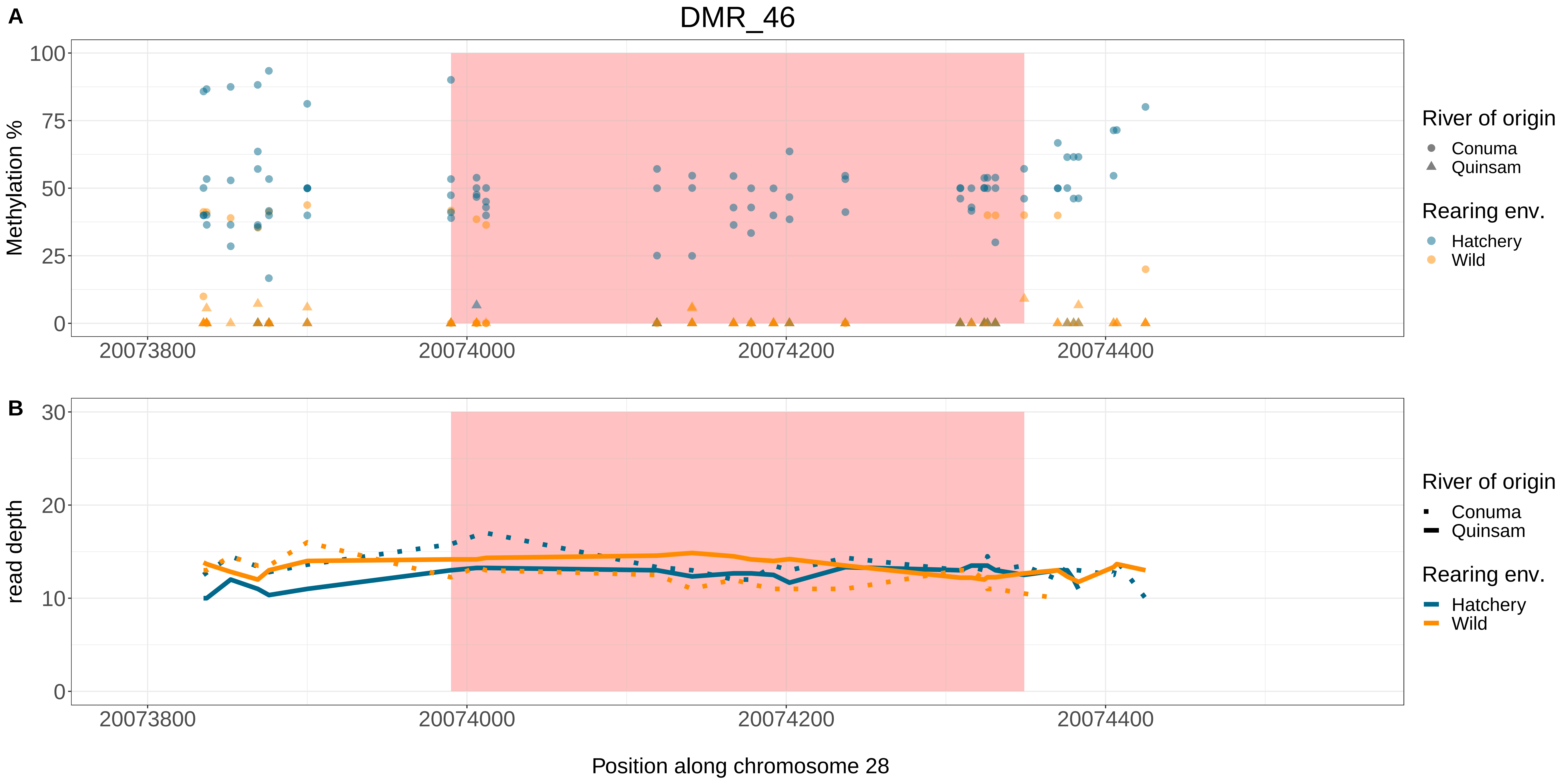

A
DMR_46
Methylation %
100
75
50
25
0
20073800
20074000
20074200
20074400
River of origin
Conuma
Quinsam
Rearing env.
Hatchery
Wild
B
read depth
30
20
10
0
20073800
20074000
20074200
20074400
River of origin
Conuma
Quinsam
Rearing env.
Hatchery
Wild
Position along chromosome 28

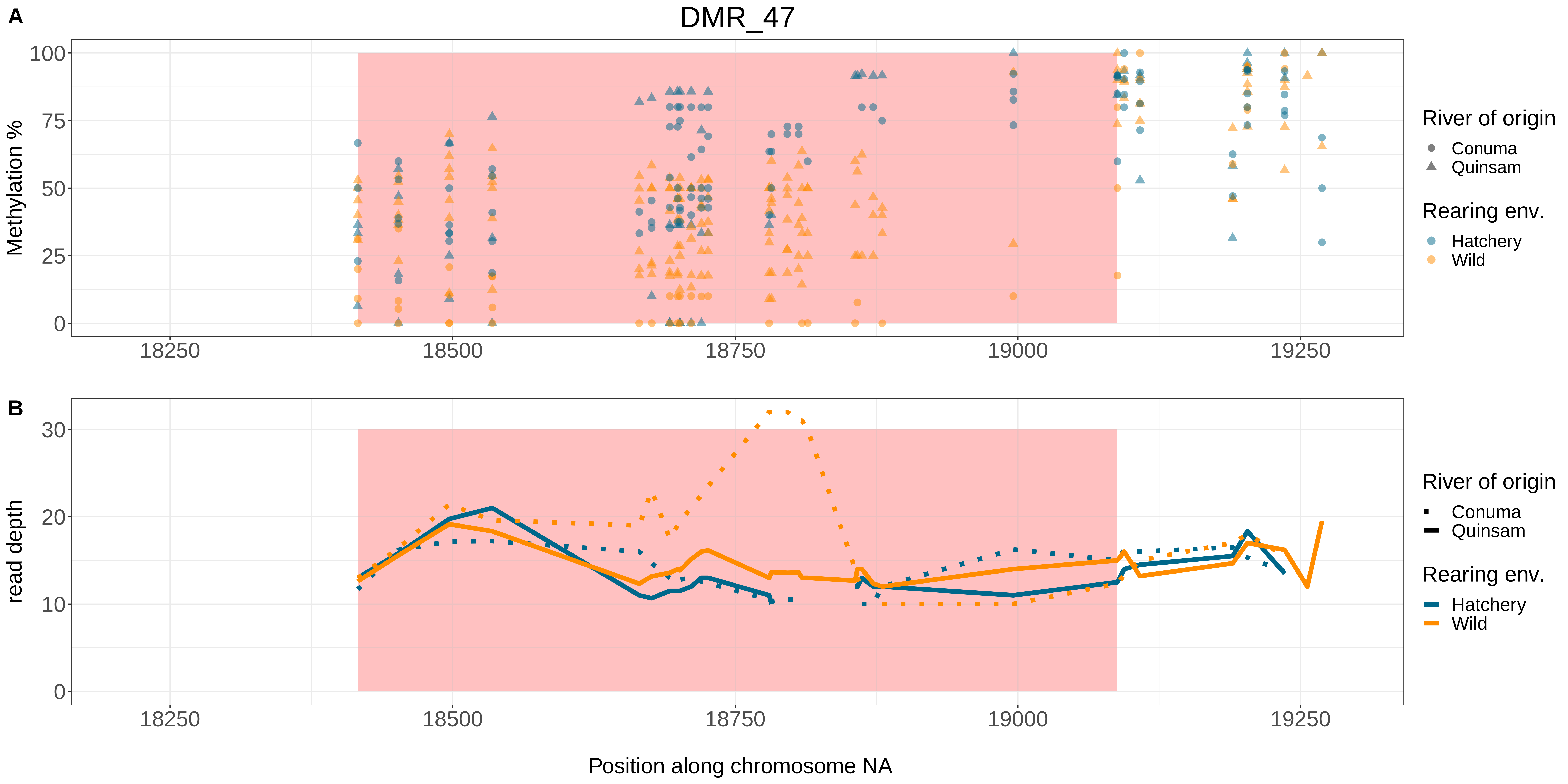

A
DMR_47
Methylation %
100
75
50
25
0
18250
18500
18750
19000
19250
River of origin
Conuma
Quinsam
Rearing env.
Hatchery
Wild
B
read depth
30
20
10
0
18250
18500
18750
19000
19250
River of origin
Conuma
Quinsam
Rearing env.
Hatchery
Wild
Position along chromosome NA

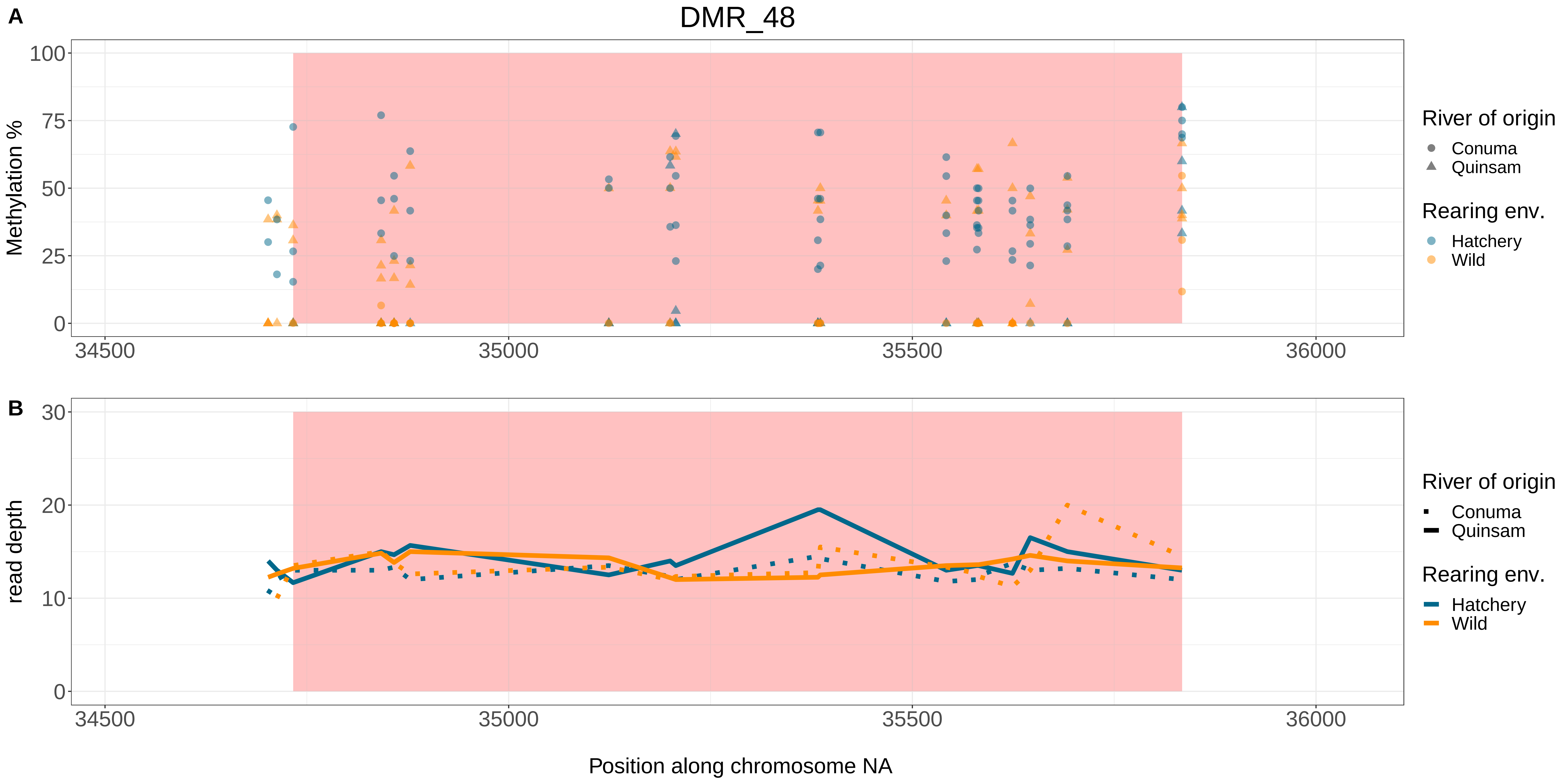

A
DMR_48
Methylation %
100
75
50
25
0
34500
35000
35500
36000
River of origin
Conuma
Quinsam
Rearing env.
Hatchery
Wild
B
read depth
30
20
10
0
34500
35000
35500
36000
River of origin
Conuma
Quinsam
Rearing env.
Hatchery
Wild
Position along chromosome NA

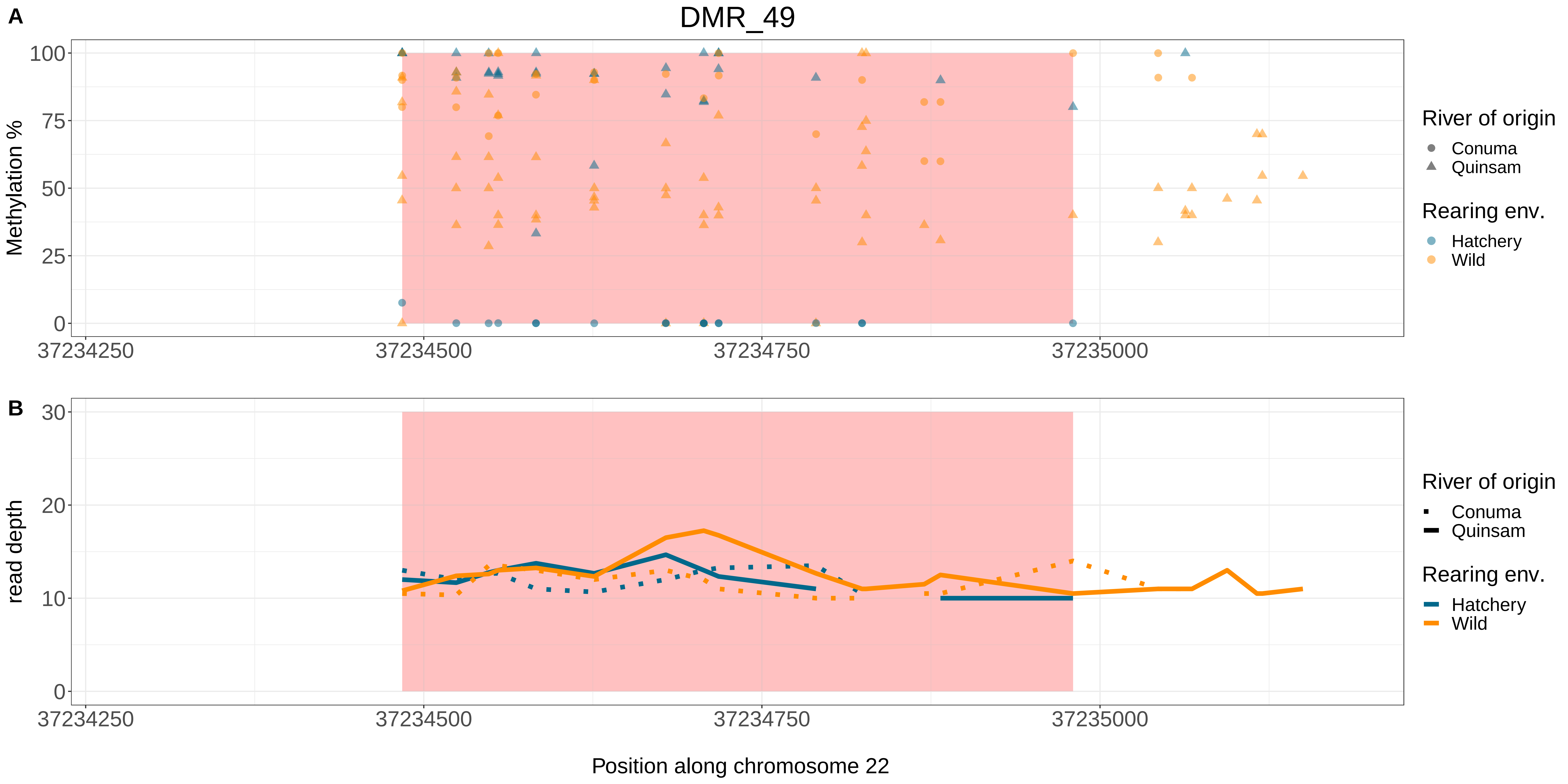

A
DMR_49
100
75
50
25
0
Methylation %
37234250
37234500
37234750
37235000
River of origin
Conuma
Quinsam
Rearing env.
Hatchery
Wild
B
30
20
10
0
read depth
37234250
37234500
37234750
37235000
River of origin
Conuma
Quinsam
Rearing env.
Hatchery
Wild
Position along chromosome 22

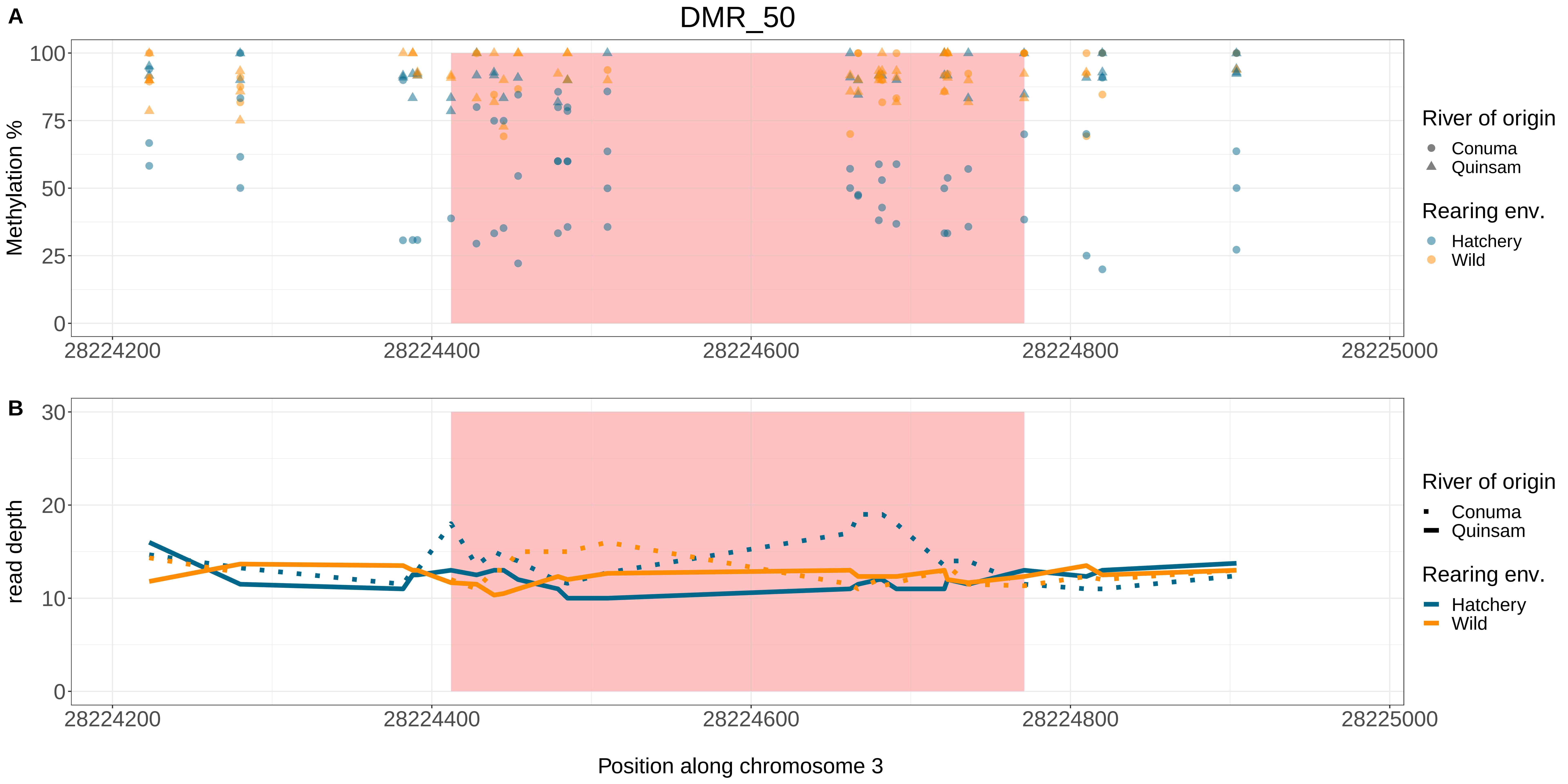

A
DMR_50
Methylation %
100
75
50
25
0
28224200
28224400
28224600
28224800
28225000
River of origin
Conuma
Quinsam
Rearing env.
Hatchery
Wild
B
read depth
30
20
10
0
28224200
28224400
28224600
28224800
28225000
River of origin
Conuma
Quinsam
Rearing env.
Hatchery
Wild
Position along chromosome 3

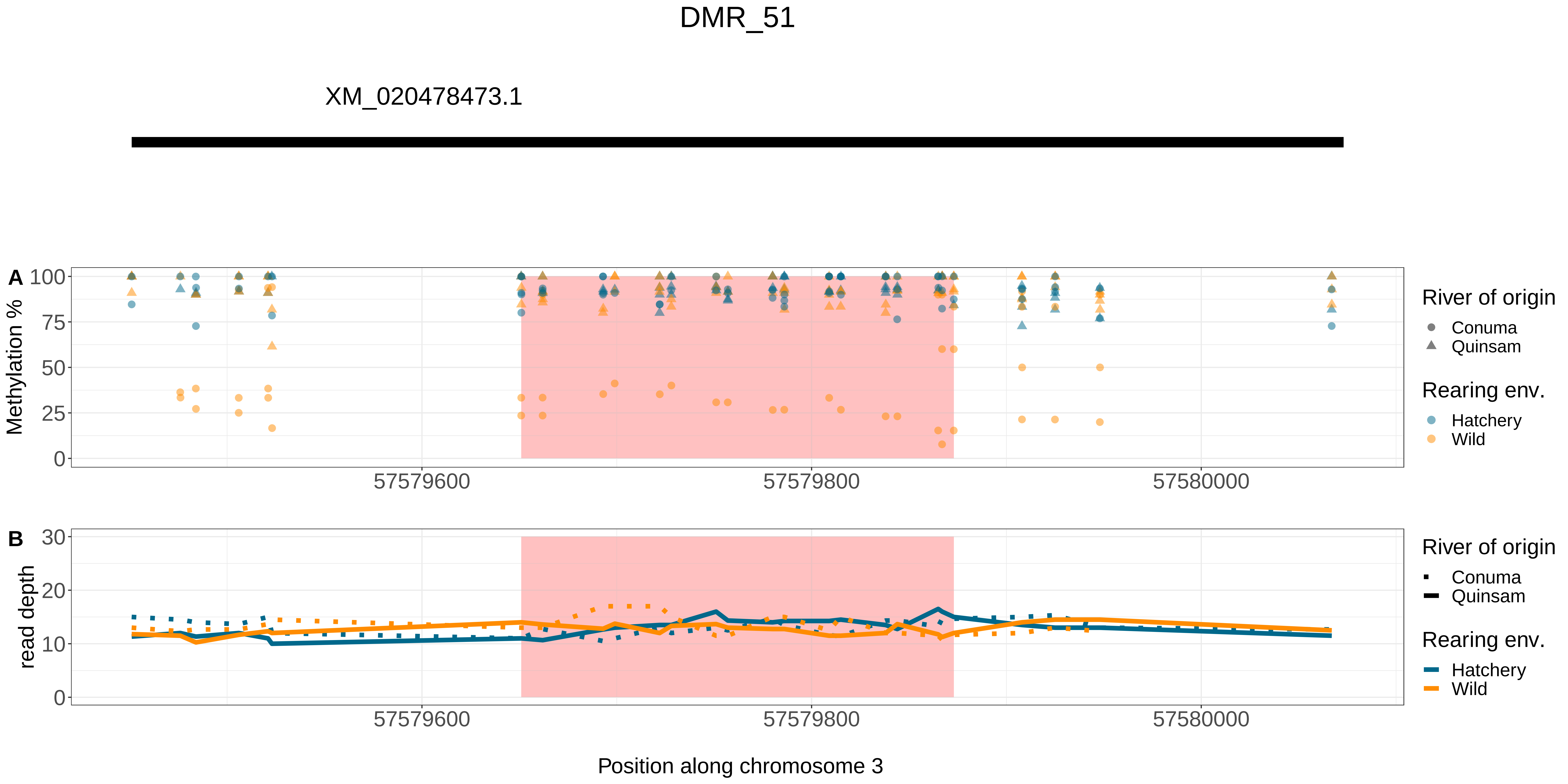
DMR_51
XM_020478473.1
A
Methylation %
100
75
50
25
0
57579600
57579800
57580000
River of origin
Conuma
Quinsam
Rearing env.
Hatchery
Wild
B
read depth
30
20
10
0
57579600
57579800
57580000
River of origin
Conuma
Quinsam
Rearing env.
Hatchery
Wild
Position along chromosome 3

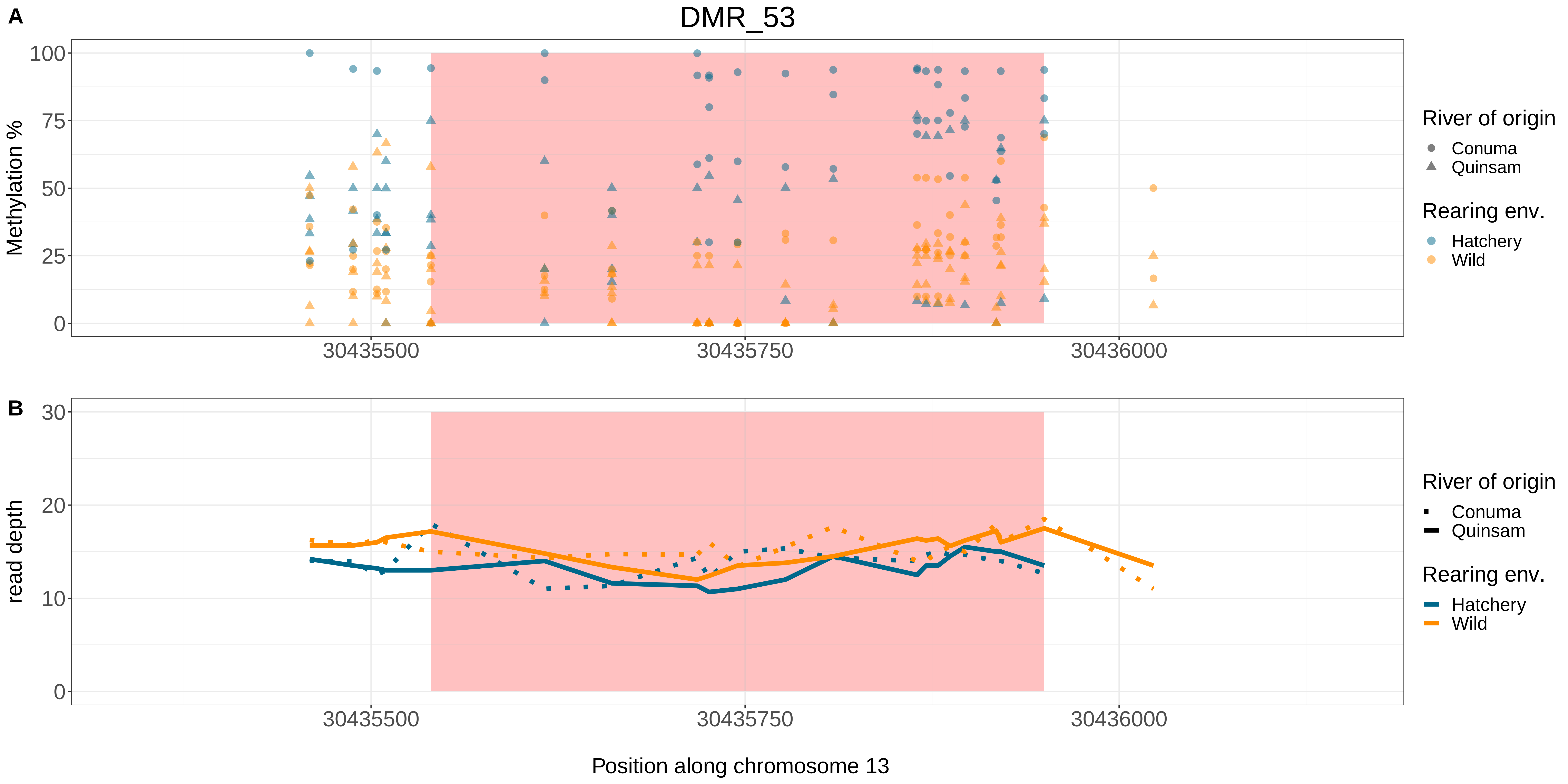

A
DMR_53
Methylation %
100
75
50
25
0
30435500
30435750
30436000
River of origin
Conuma
Quinsam
Rearing env.
Hatchery
Wild
B
read depth
30
20
10
0
30435500
30435750
30436000
River of origin
Conuma
Quinsam
Rearing env.
Hatchery
Wild
Position along chromosome 13

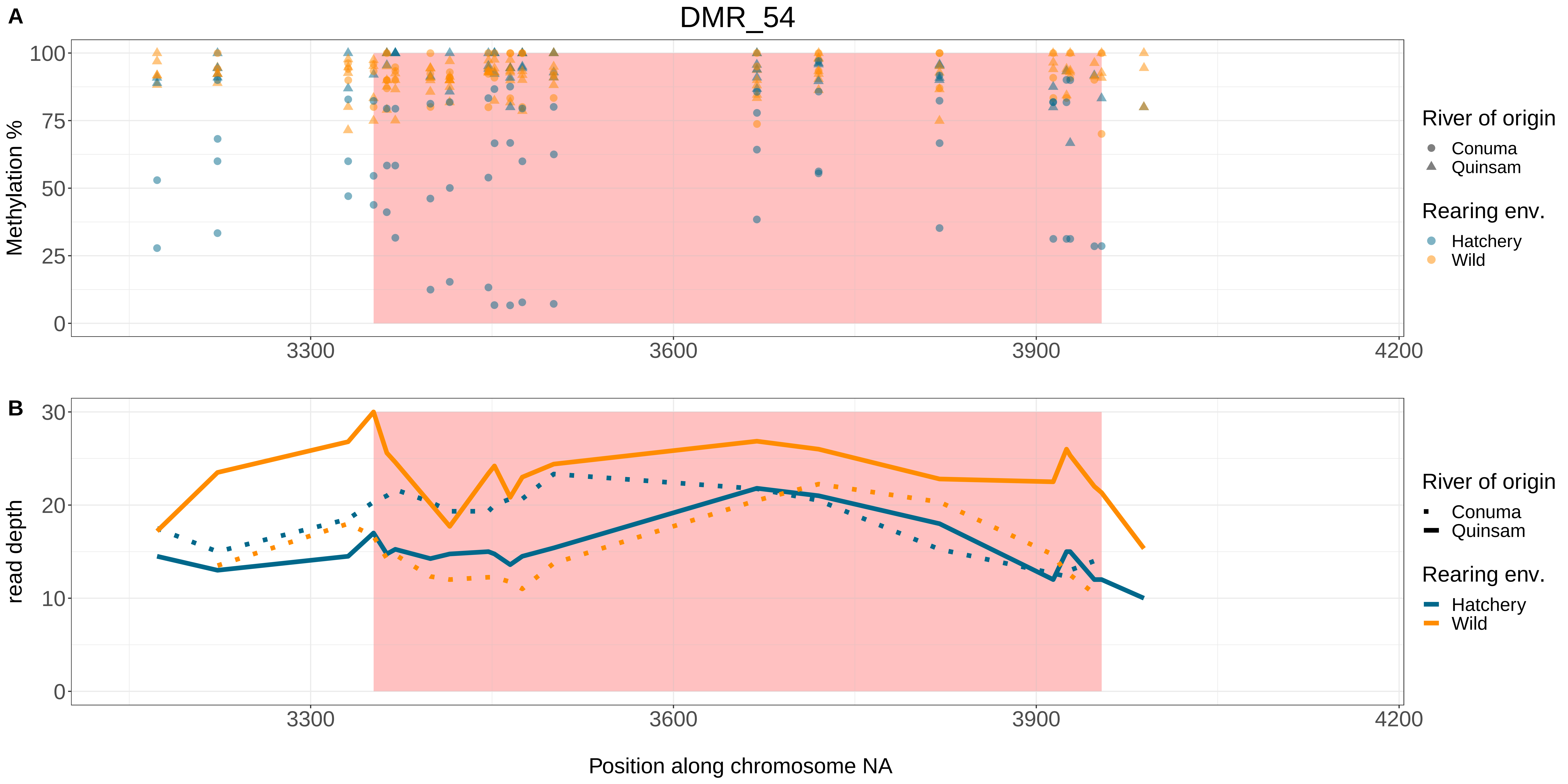

A
DMR_54
Methylation %
100
75
50
25
0
3300
3600
3900
4200
River of origin
Conuma
Quinsam
Rearing env.
Hatchery
Wild
B
read depth
30
20
10
0
3300
3600
3900
4200
River of origin
Conuma
Quinsam
Rearing env.
Hatchery
Wild
Position along chromosome NA

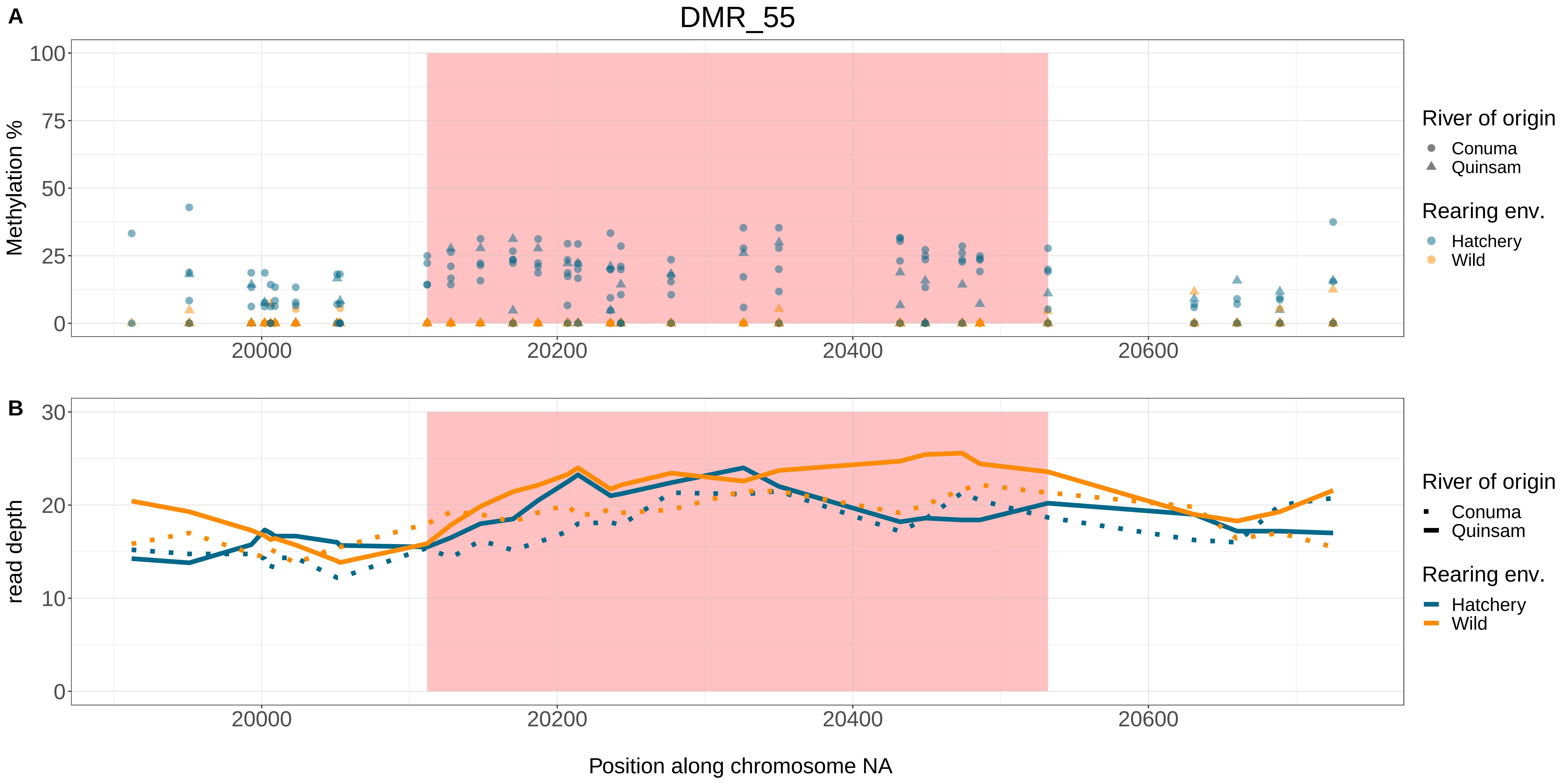

A
DMR_55
Methylation %
100
75
50
25
0
20000
20200
20400
20600
River of origin
Conuma
Quinsam
Rearing env.
Hatchery
Wild
B
read depth
30
20
10
0
20000
20200
20400
20600
River of origin
Conuma
Quinsam
Rearing env.
Hatchery
Wild
Position along chromosome NA

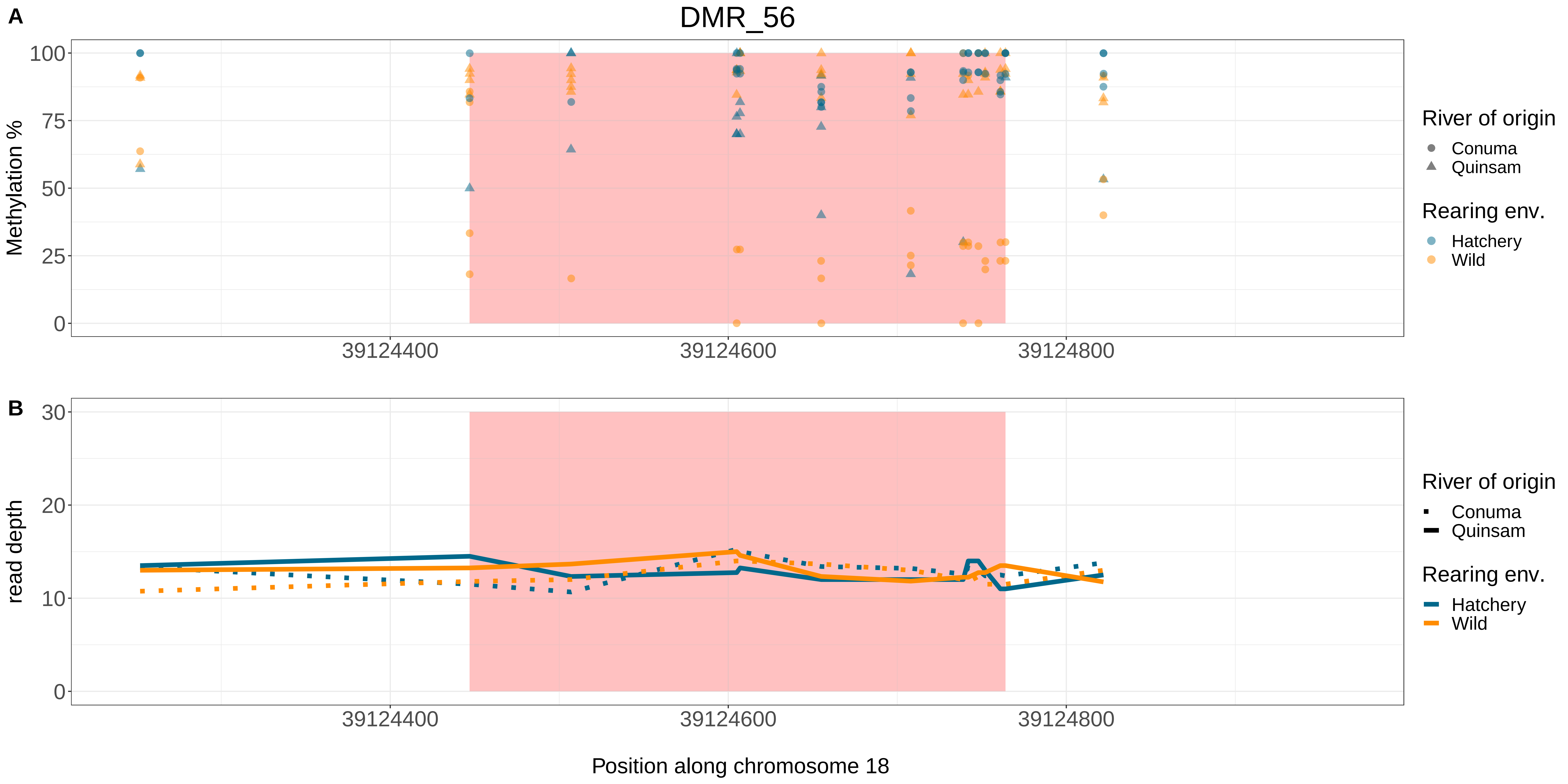
A
DMR_56
Methylation %
100
75
50
25
0
39124400
39124600
39124800
River of origin
Conuma
Quinsam
Rearing env.
Hatchery
Wild
B
read depth
30
20
10
0
39124400
39124600
39124800
River of origin
Conuma
Quinsam
Rearing env.
Hatchery
Wild
Position along chromosome 18

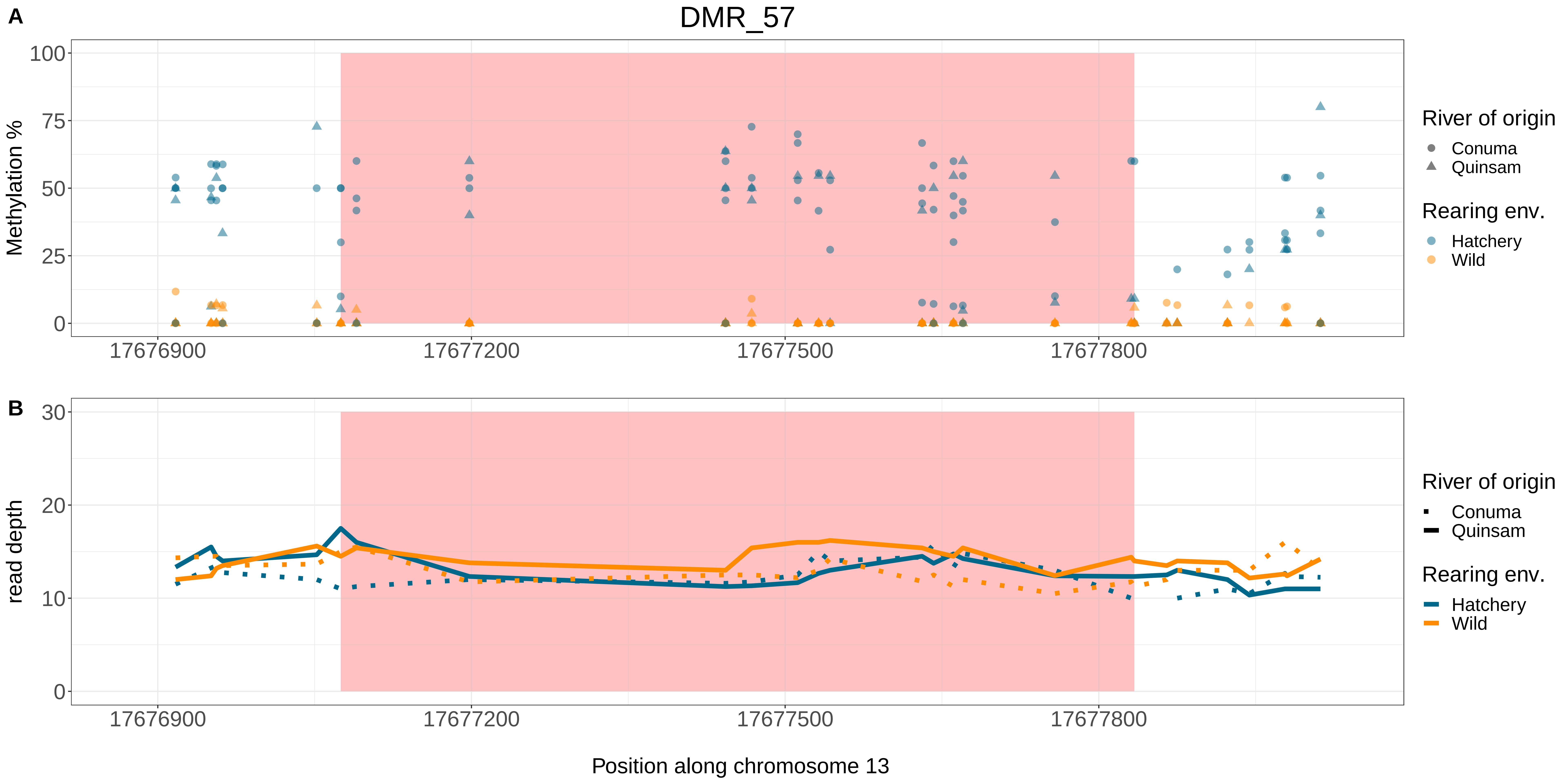
A
DMR_57
Methylation %
100
75
50
25
0
17676900
17677200
17677500
17677800
River of origin
Conuma
Quinsam
Rearing env.
Hatchery
Wild
B
read depth
30
20
10
0
17676900
17677200
17677500
17677800
River of origin
Conuma
Quinsam
Rearing env.
Hatchery
Wild
Position along chromosome 13

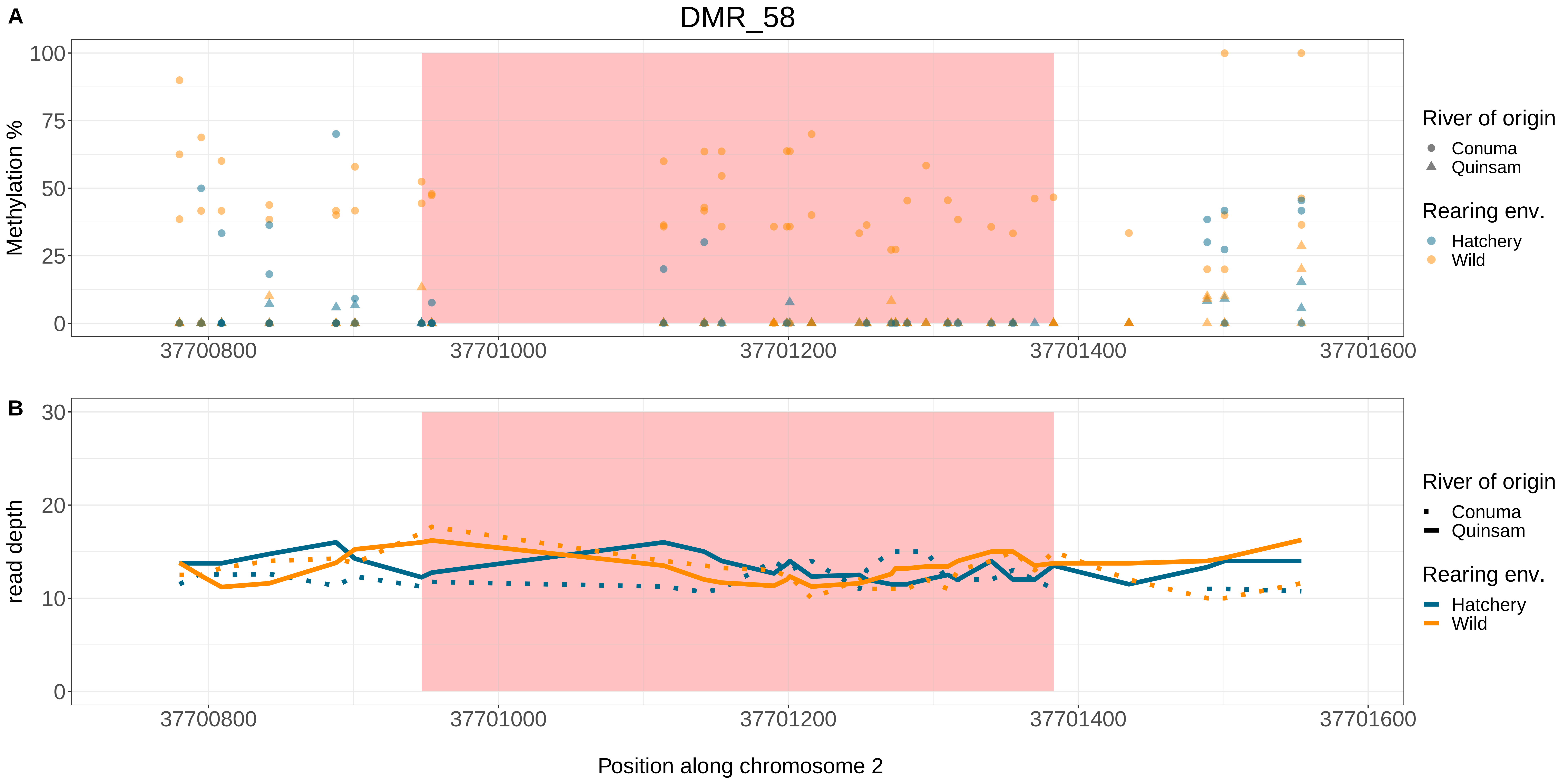
A
DMR_58
Methylation %
100
75
50
25
0
37700800
37701000
37701200
37701400
37701600
River of origin
Conuma
Quinsam
Rearing env.
Hatchery
Wild
B
read depth
30
20
10
0
37700800
37701000
37701200
37701400
37701600
River of origin
Conuma
Quinsam
Rearing env.
Hatchery
Wild
Position along chromosome 2

DMR_59

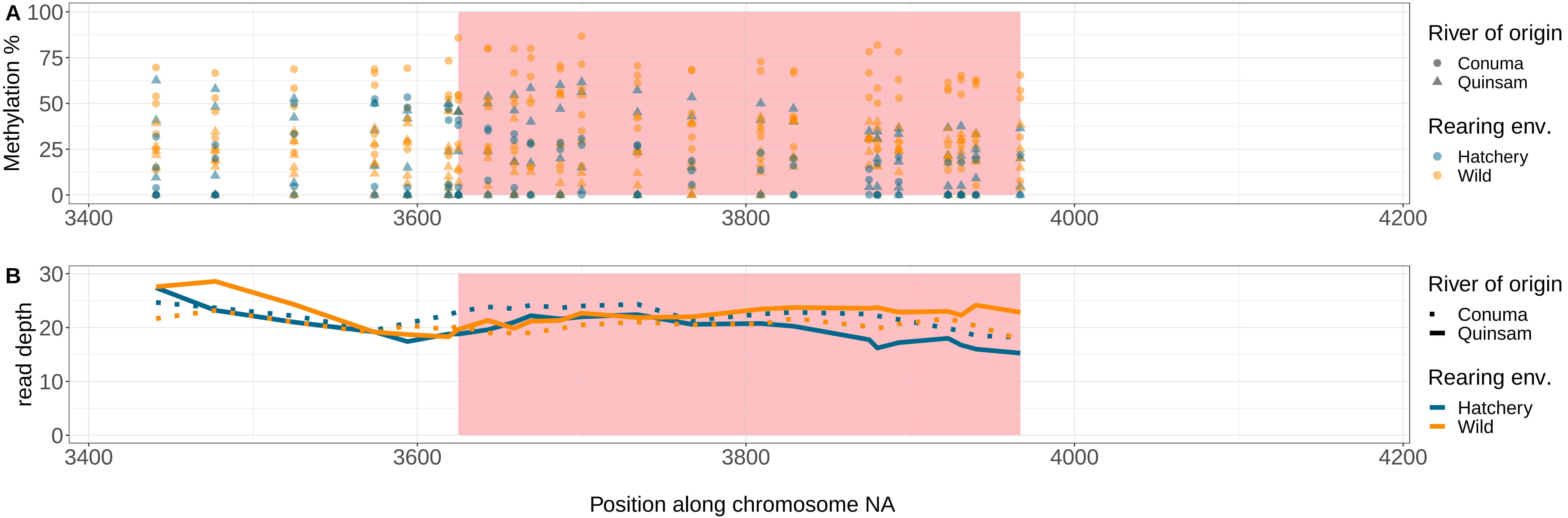
A
Methylation %
100
75
50
25
0
3400
3600
3800
4000
4200
River of origin
Conuma
Quinsam
Rearing env.
Hatchery
Wild
B
read depth
30
20
10
0
Position along chromosome NA

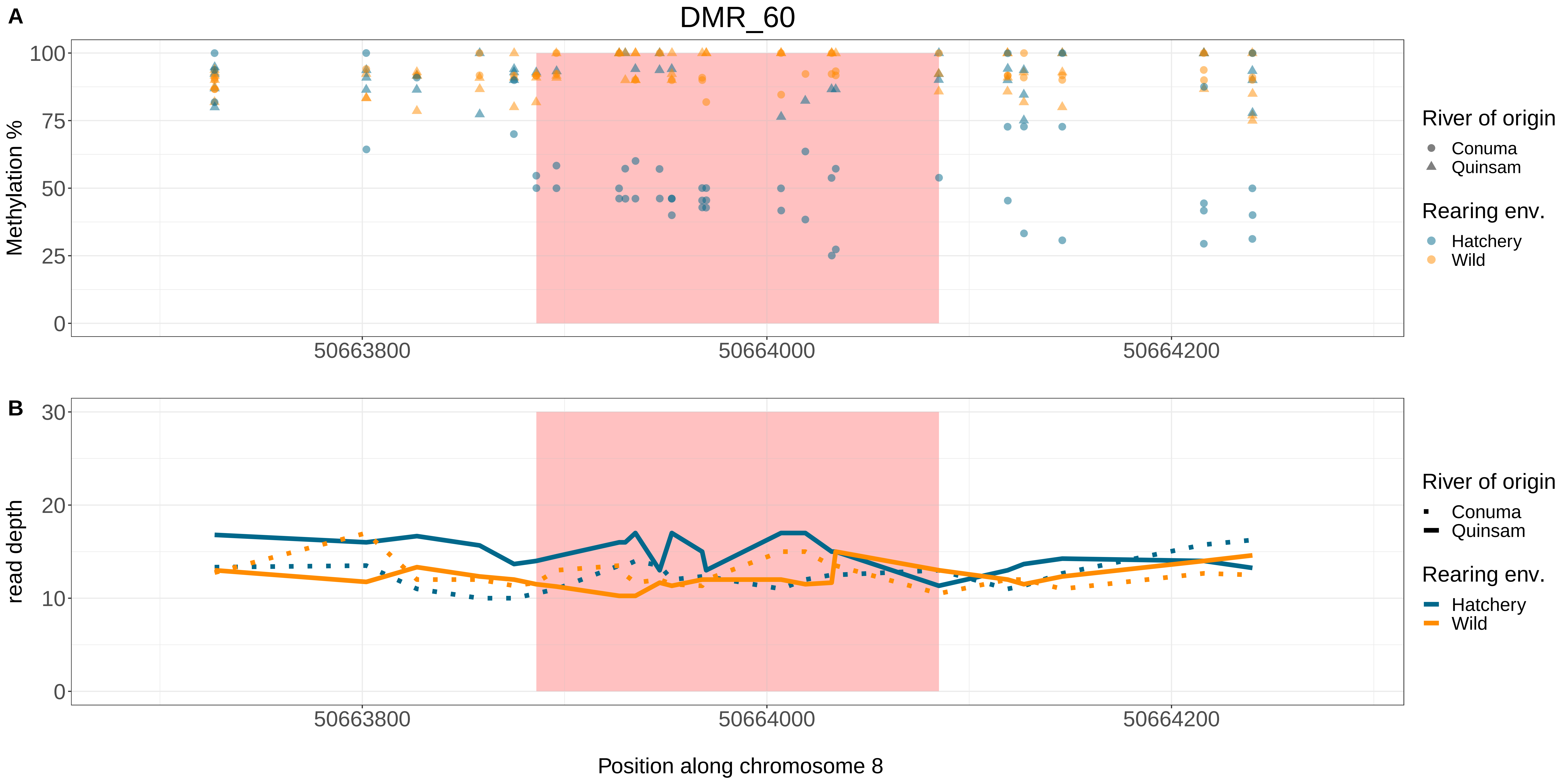

A
DMR_60
100
75
50
25
0
Methylation %
50663800
50664000
50664200
River of origin
Conuma
Quinsam
Rearing env.
Hatchery
Wild
B
30
20
10
0
read depth
50663800
50664000
50664200
River of origin
Conuma
Quinsam
Rearing env.
Hatchery
Wild
Position along chromosome 8

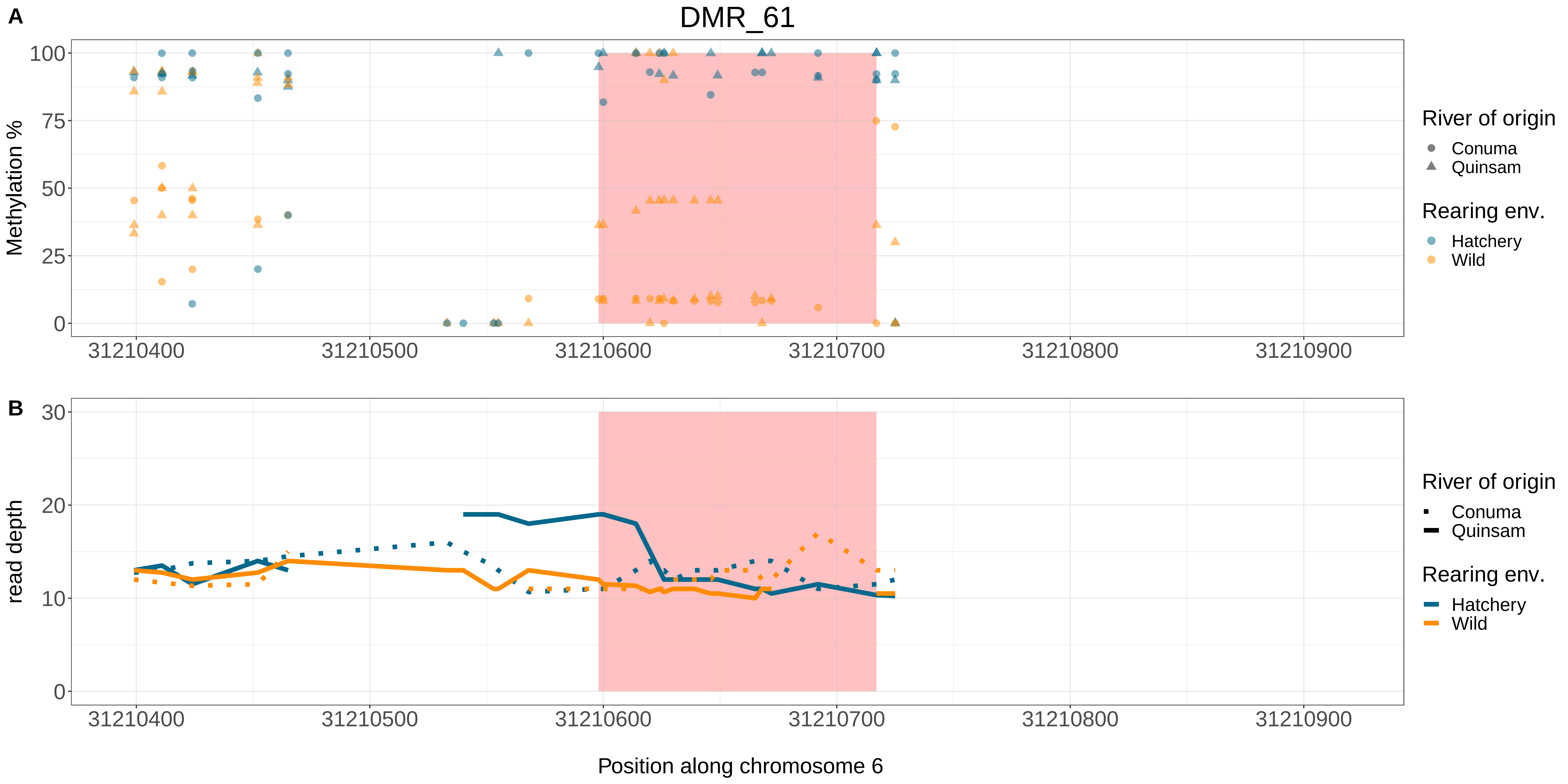
A
DMR_61
Methylation %
100
75
50
25
0
31210400
31210500
31210600
31210700
31210800
31210900
River of origin
Conuma
Quinsam
Rearing env.
Hatchery
Wild
B
read depth
30
20
10
0
31210400
31210500
31210600
31210700
31210800
31210900
River of origin
Conuma
Quinsam
Rearing env.
Hatchery
Wild
Position along chromosome 6

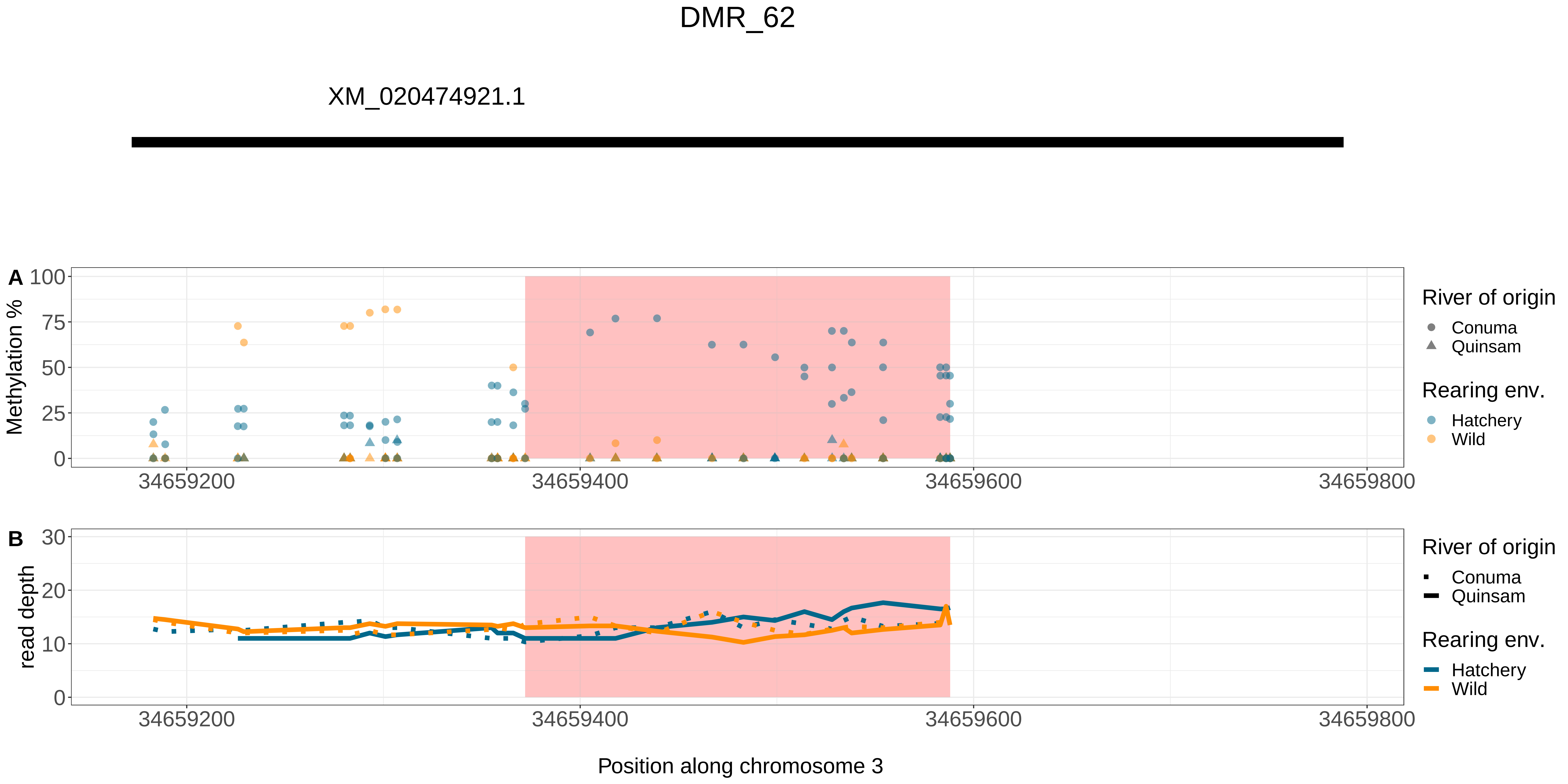

DMR_62
XM_020474921.1
A
B
Methylation %
read depth
0
25
50
75
100
10
20
30
34659200
34659400
34659600
34659800
River of origin
Conuma
Quinsam
Rearing env.
Hatchery
Wild
Position along chromosome 3

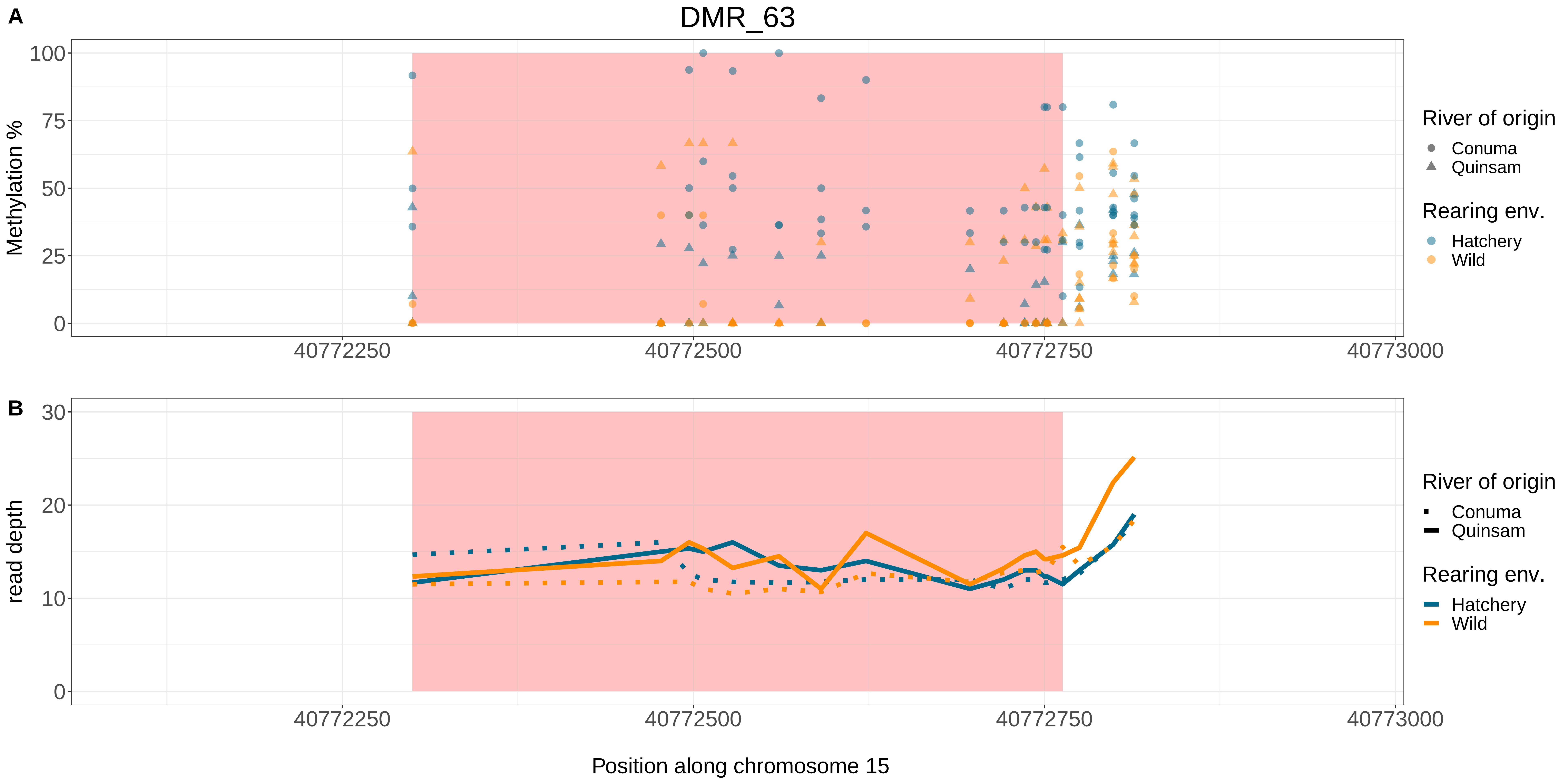
A
DMR_63
Methylation %
100
75
50
25
0
40772250
40772500
40772750
40773000
River of origin
Conuma
Quinsam
Rearing env.
Hatchery
Wild
B
read depth
30
20
10
0
Position along chromosome 15

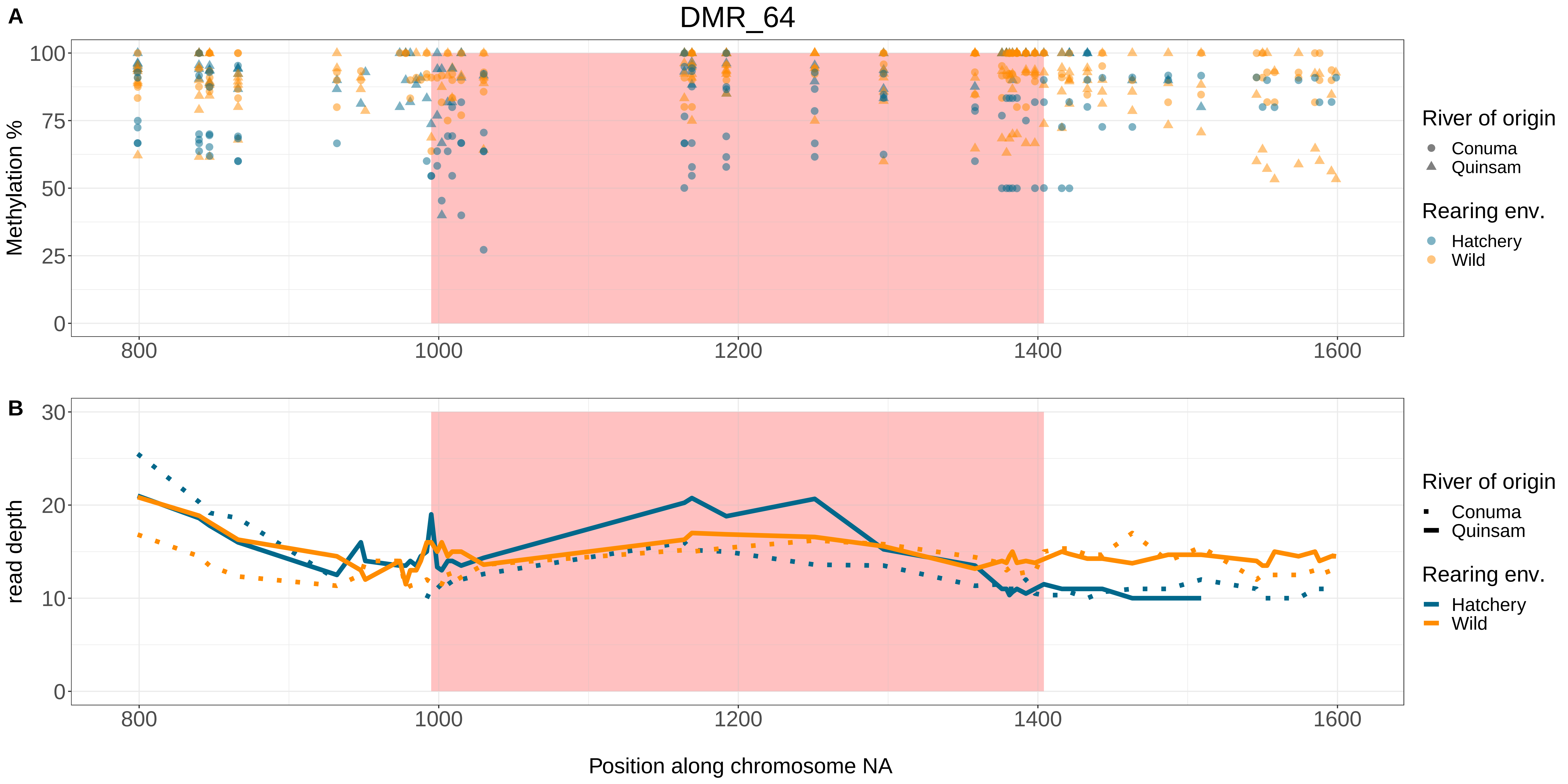

A
DMR_64
Methylation %
100
75
50
25
0
800
1000
1200
1400
1600
River of origin
Conuma
Quinsam
Rearing env.
Hatchery
Wild
B
read depth
30
20
10
0
800
1000
1200
1400
1600
River of origin
Conuma
Quinsam
Rearing env.
Hatchery
Wild
Position along chromosome NA

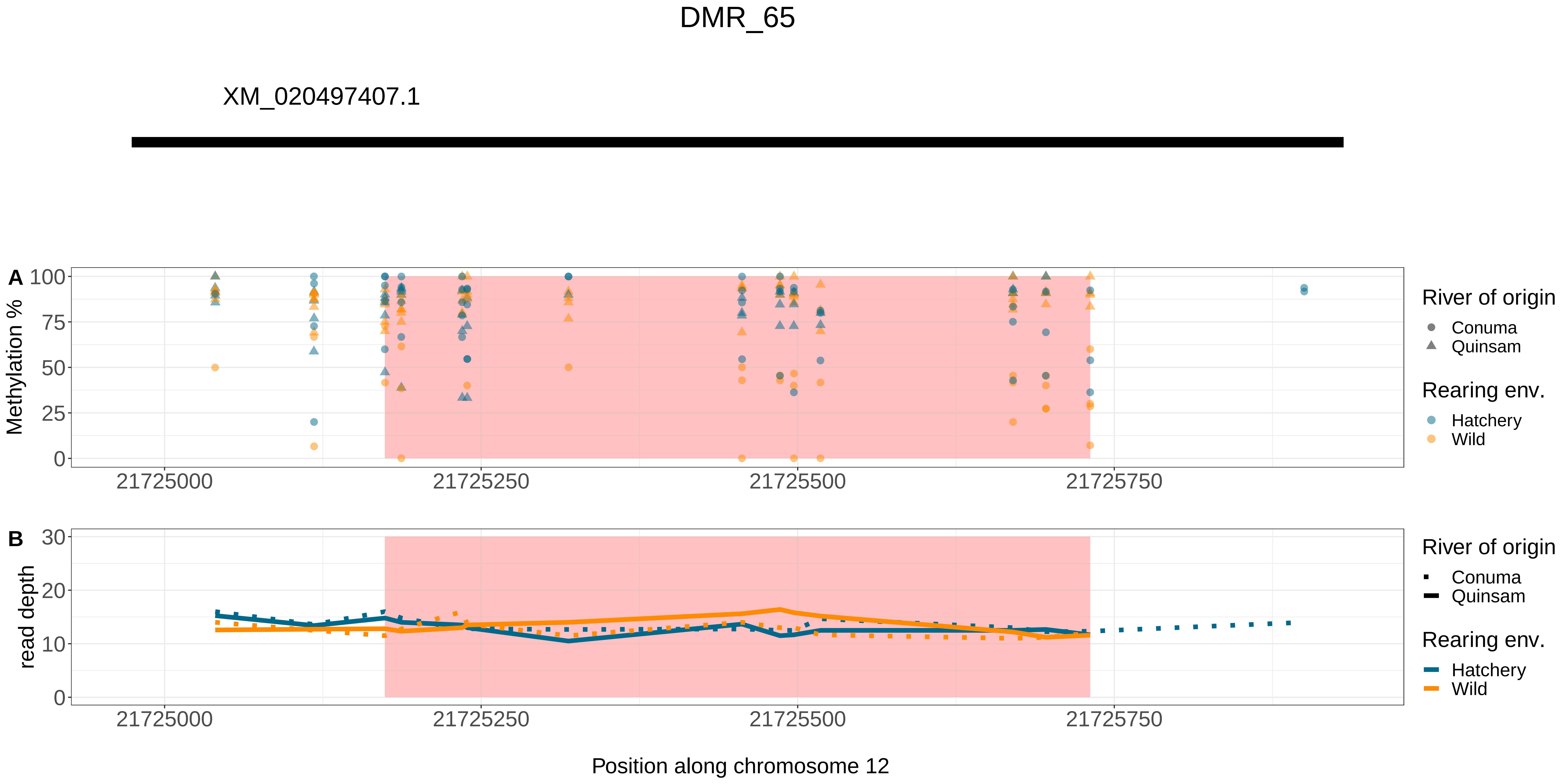

DMR_65
XM_020497407.1
A
B
Methylation %
100
75
50
25
0
read depth
30
20
10
0
21725000
21725250
21725500
21725750
Position along chromosome 12
River of origin
Conuma
Quinsam
Rearing env.
Hatchery
Wild

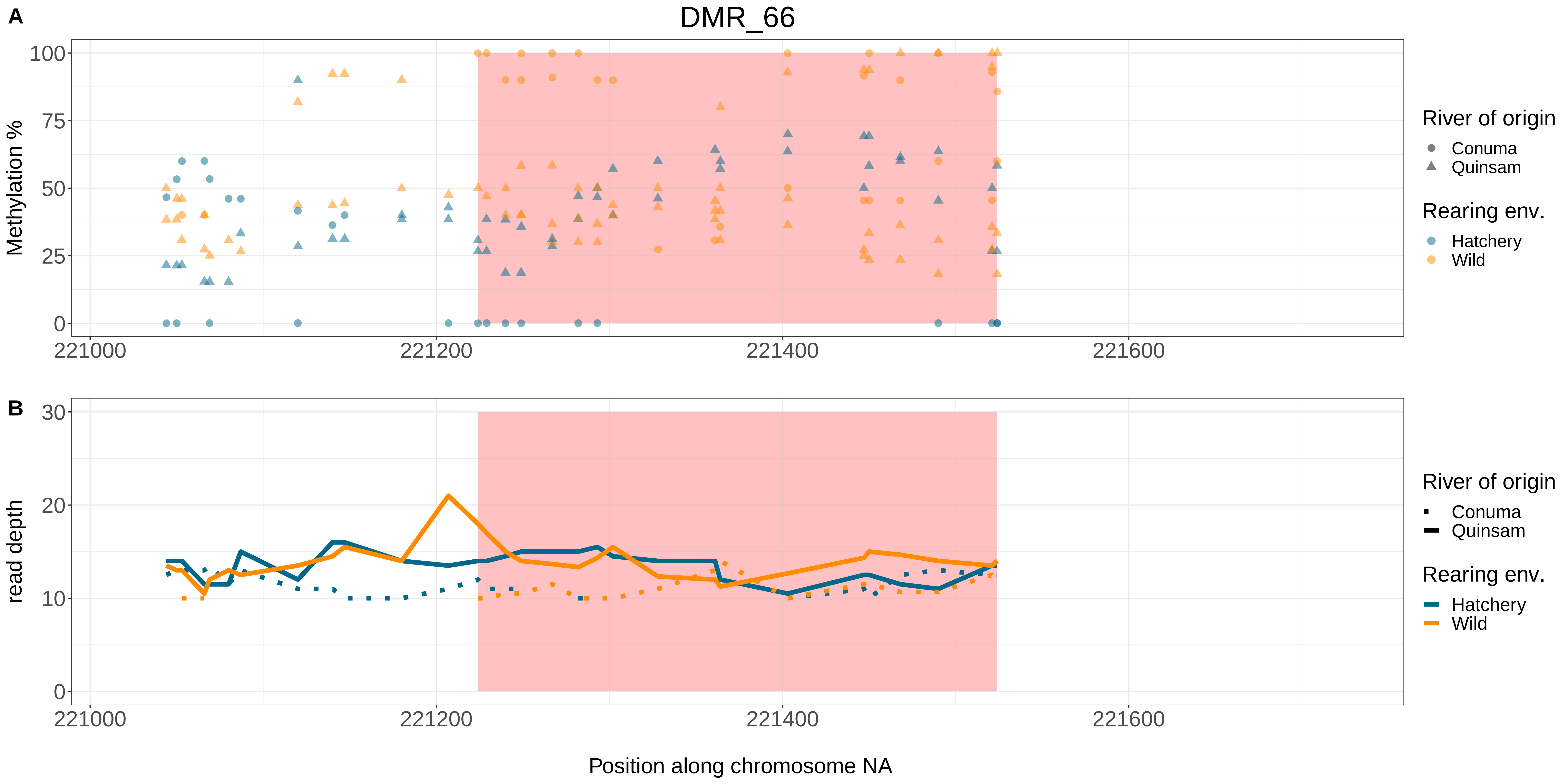
A
DMR_66
Methylation %
100
75
50
25
0
221000
221200
221400
221600
River of origin
Conuma
Quinsam
Rearing env.
Hatchery
Wild
B
read depth
30
20
10
0
221000
221200
221400
221600
River of origin
Conuma
Quinsam
Rearing env.
Hatchery
Wild
Position along chromosome NA

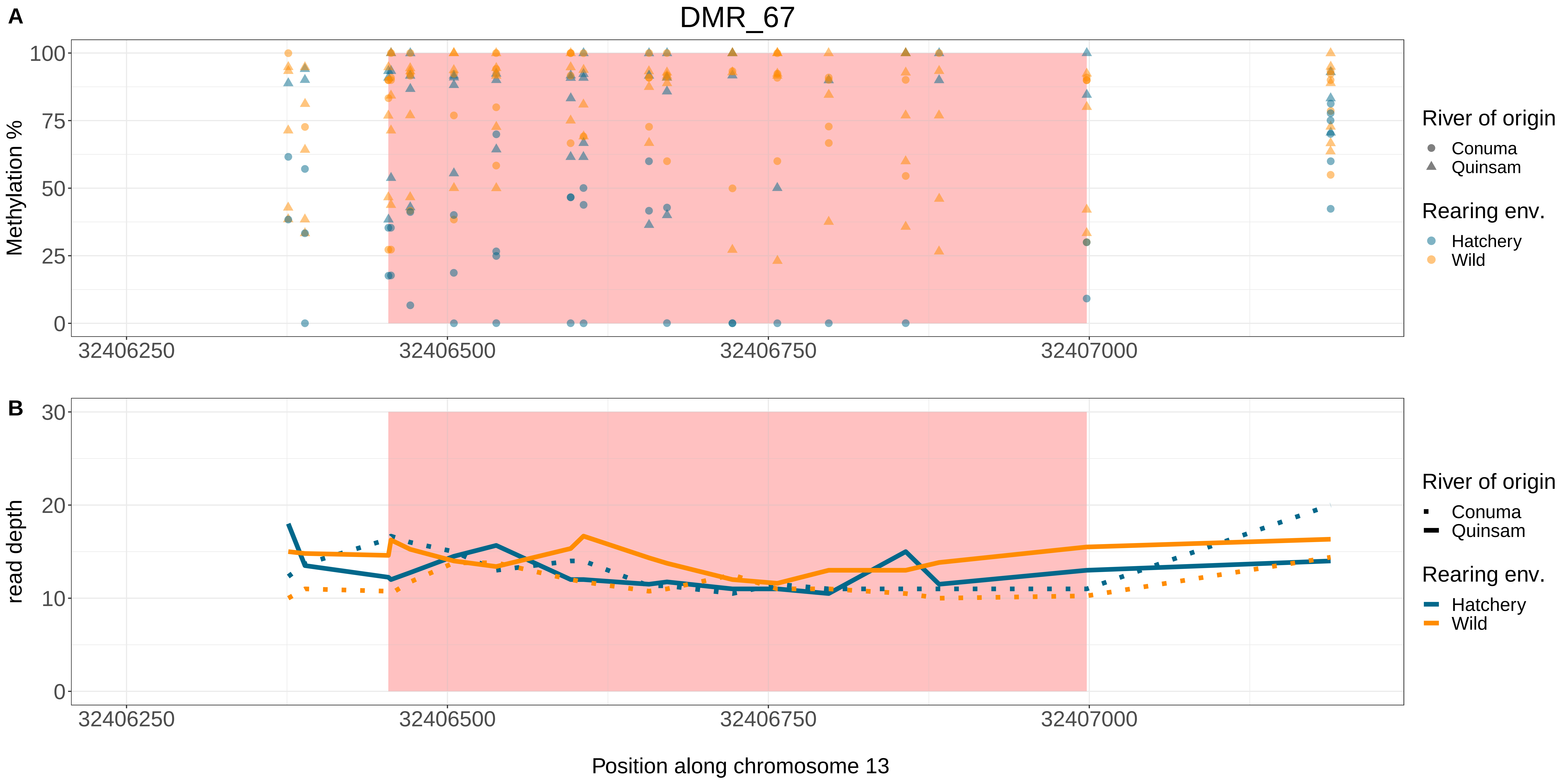
A
DMR_67
Methylation %
100
75
50
25
0
32406250
32406500
32406750
32407000
River of origin
Conuma
Quinsam
Rearing env.
Hatchery
Wild
B
read depth
30
20
10
0
32406250
32406500
32406750
32407000
River of origin
Conuma
Quinsam
Rearing env.
Hatchery
Wild
Position along chromosome 13

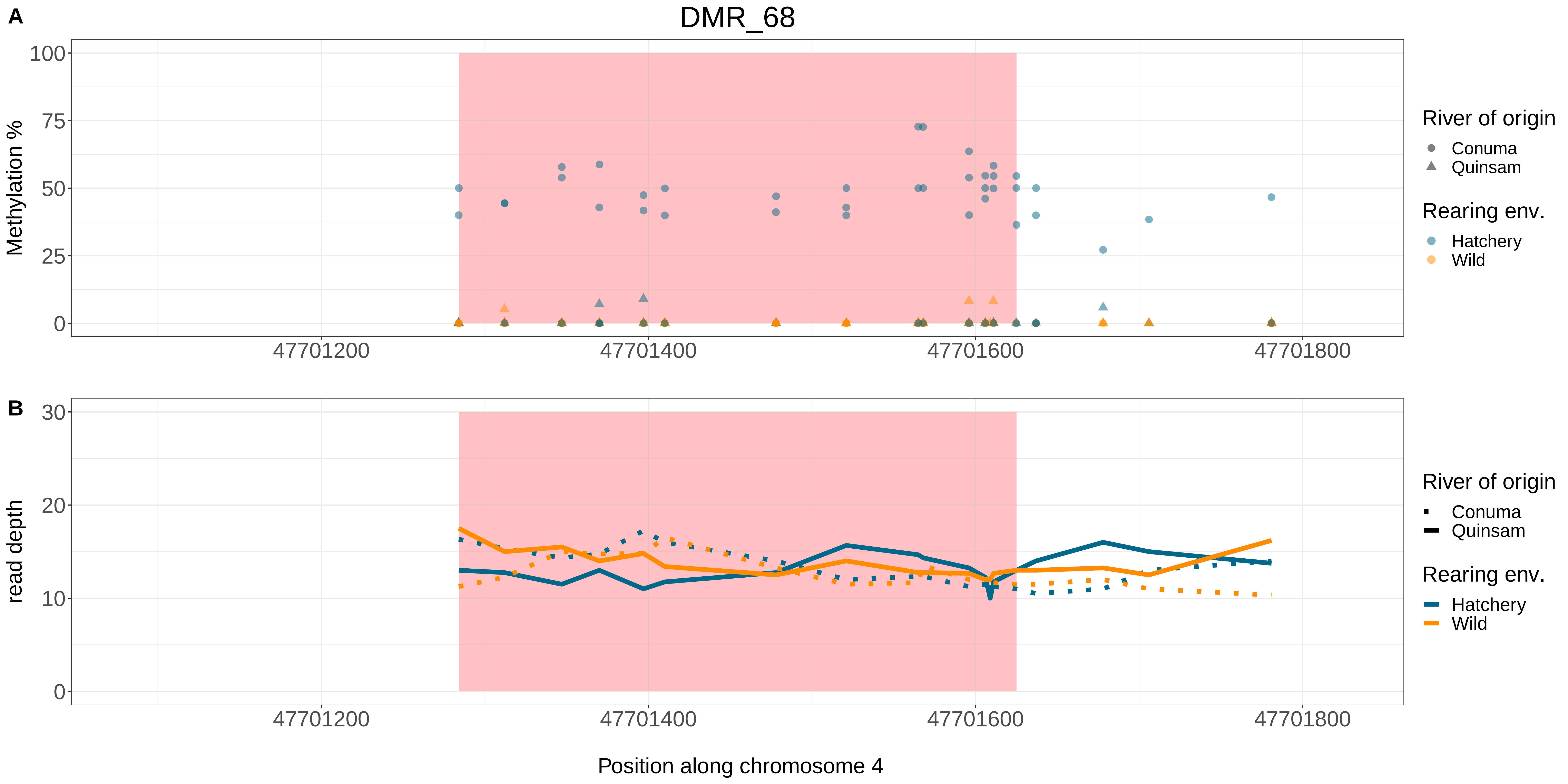

A
DMR_68
Methylation %
100
75
50
25
0
47701200
47701400
47701600
47701800
River of origin
Conuma
Quinsam
Rearing env.
Hatchery
Wild
B
read depth
30
20
10
0
47701200
47701400
47701600
47701800
Position along chromosome 4
River of origin
Conuma
Quinsam
Rearing env.
Hatchery
Wild

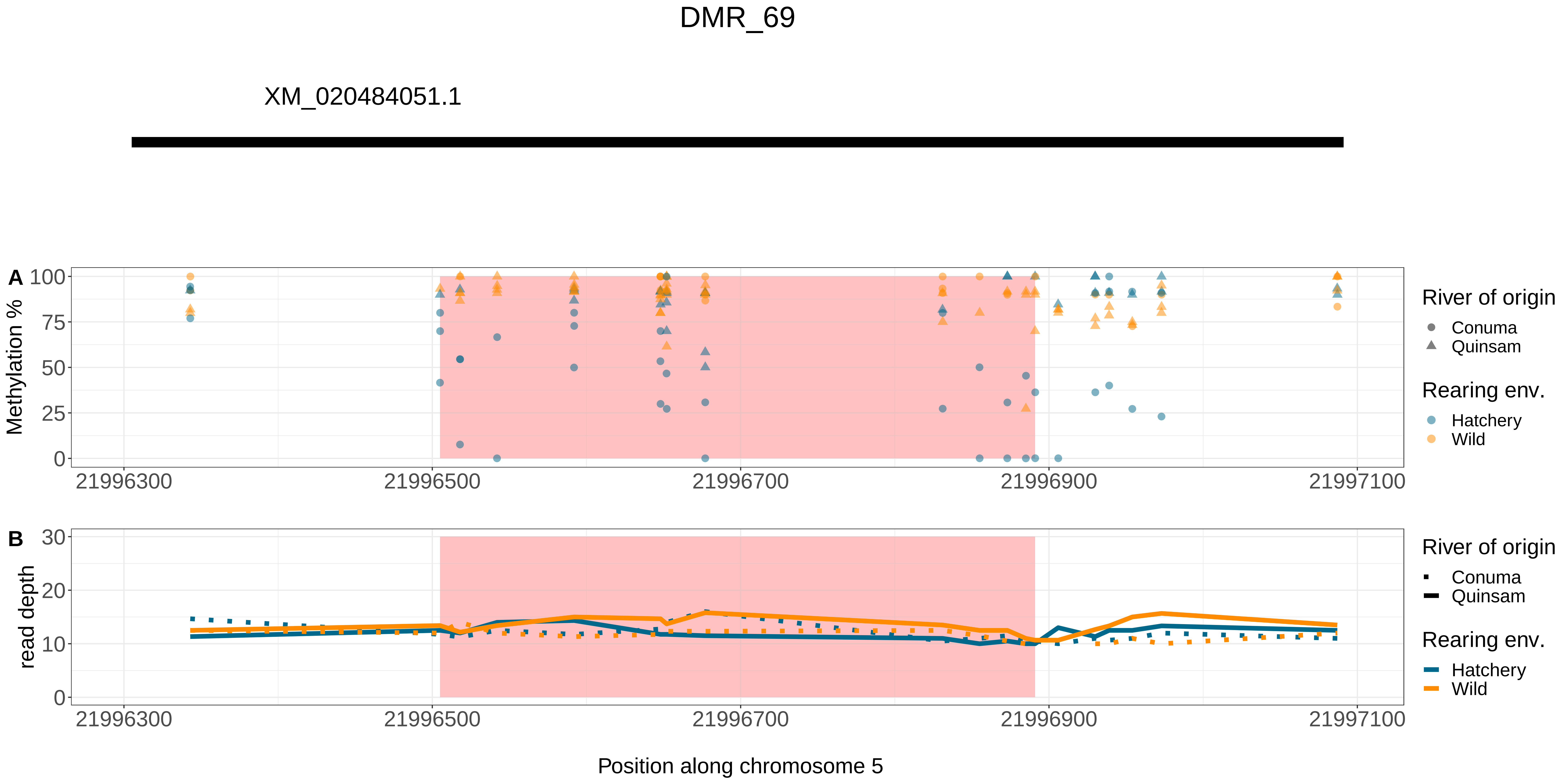

DMR_69
XM_020484051.1
A
Methylation %
100
75
50
25
0
21996300
21996500
21996700
21996900
21997100
River of origin
Conuma
Quinsam
Rearing env.
Hatchery
Wild
B
read depth
30
20
10
0
21996300
21996500
21996700
21996900
21997100
River of origin
Conuma
Quinsam
Rearing env.
Hatchery
Wild
Position along chromosome 5

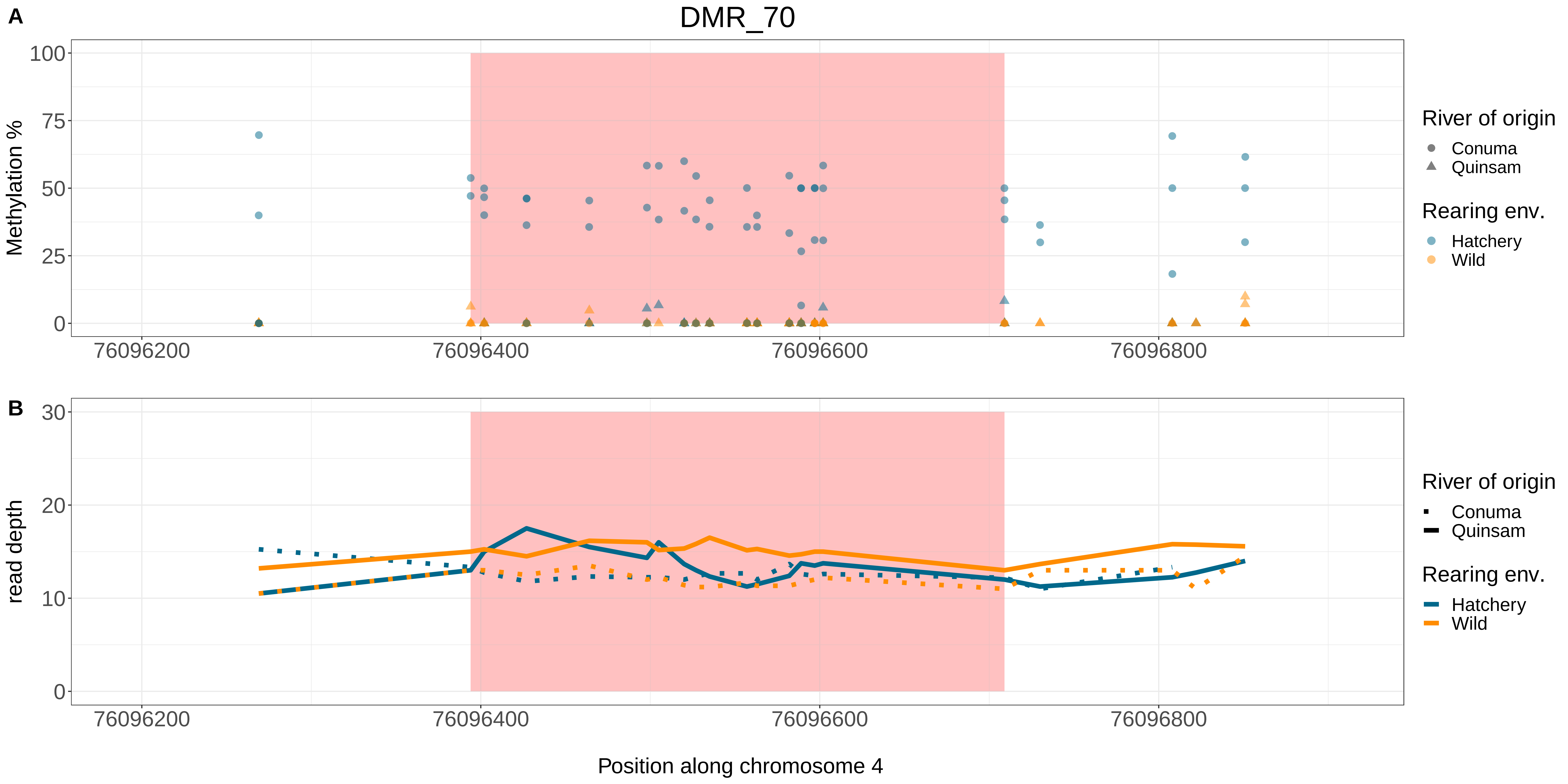

A
DMR_70
Methylation %
100
75
50
25
0
76096200
76096400
76096600
76096800
River of origin
Conuma
Quinsam
Rearing env.
Hatchery
Wild
B
read depth
30
20
10
0
76096200
76096400
76096600
76096800
River of origin
Conuma
Quinsam
Rearing env.
Hatchery
Wild
Position along chromosome 4

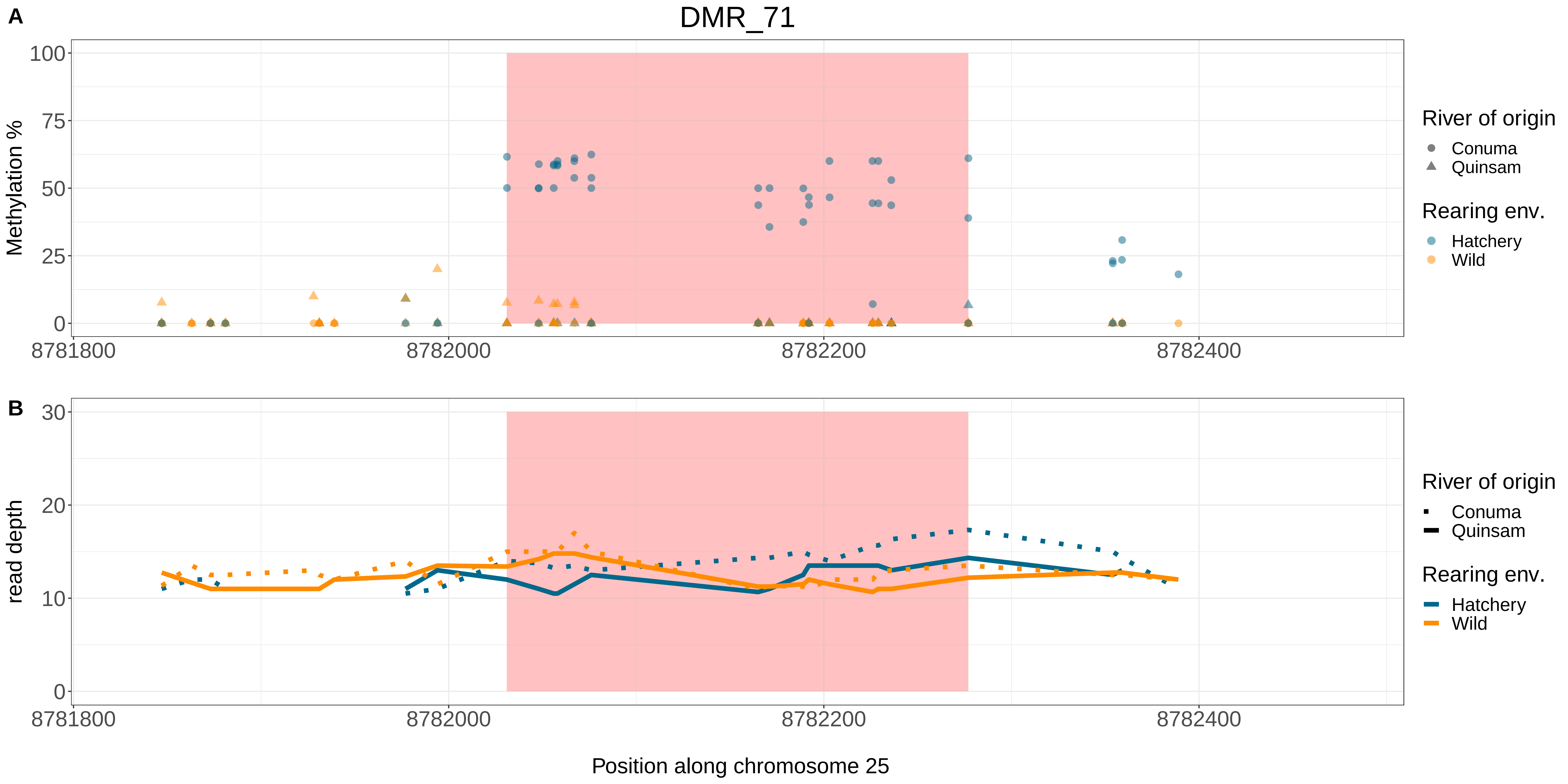

A
DMR_71
Methylation %
100
75
50
25
0
8781800
8782000
8782200
8782400
River of origin
Conuma
Quinsam
Rearing env.
Hatchery
Wild
B
read depth
30
20
10
0
8781800
8782000
8782200
8782400
River of origin
Conuma
Quinsam
Rearing env.
Hatchery
Wild
Position along chromosome 25

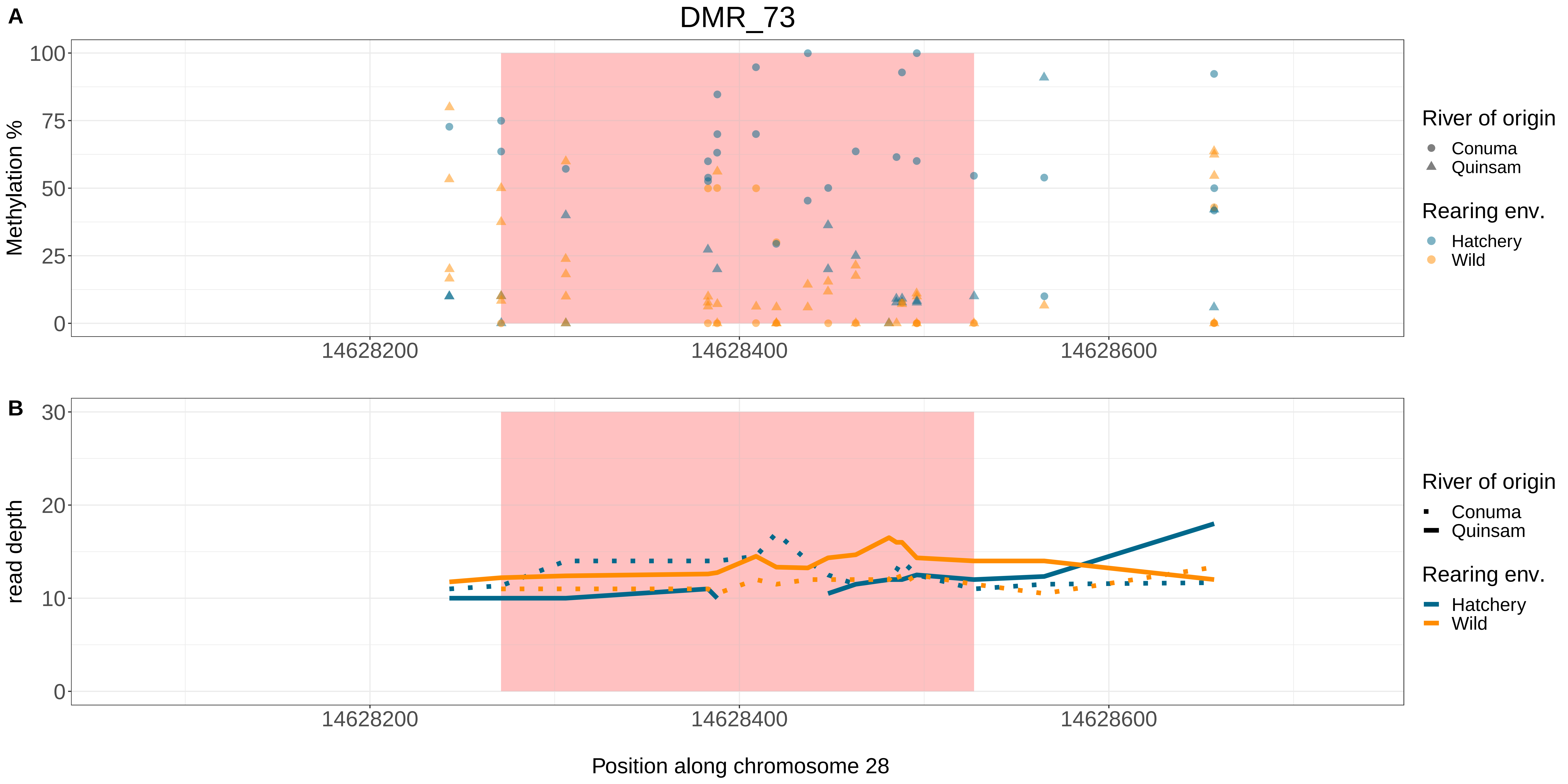

A
DMR_73
Methylation %
100
75
50
25
0
14628200
14628400
14628600
River of origin
Conuma
Quinsam
Rearing env.
Hatchery
Wild
B
read depth
30
20
10
0
14628200
14628400
14628600
River of origin
Conuma
Quinsam
Rearing env.
Hatchery
Wild
Position along chromosome 28

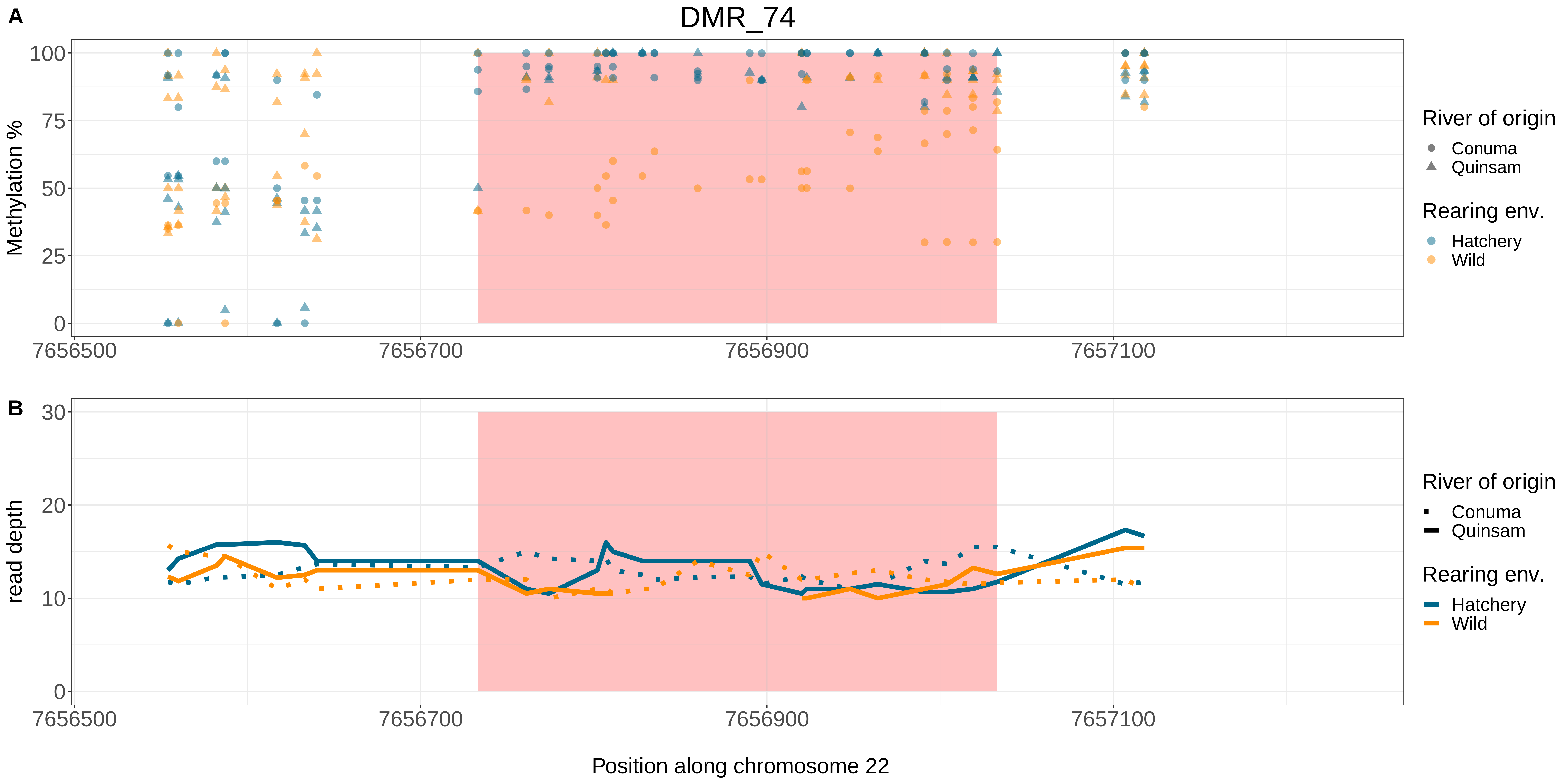
A
DMR_74
Methylation %
100
75
50
25
0
7656500
7656700
7656900
7657100
River of origin
Conuma
Quinsam
Rearing env.
Hatchery
Wild
B
read depth
30
20
10
0
7656500
7656700
7656900
7657100
River of origin
Conuma
Quinsam
Rearing env.
Hatchery
Wild
Position along chromosome 22

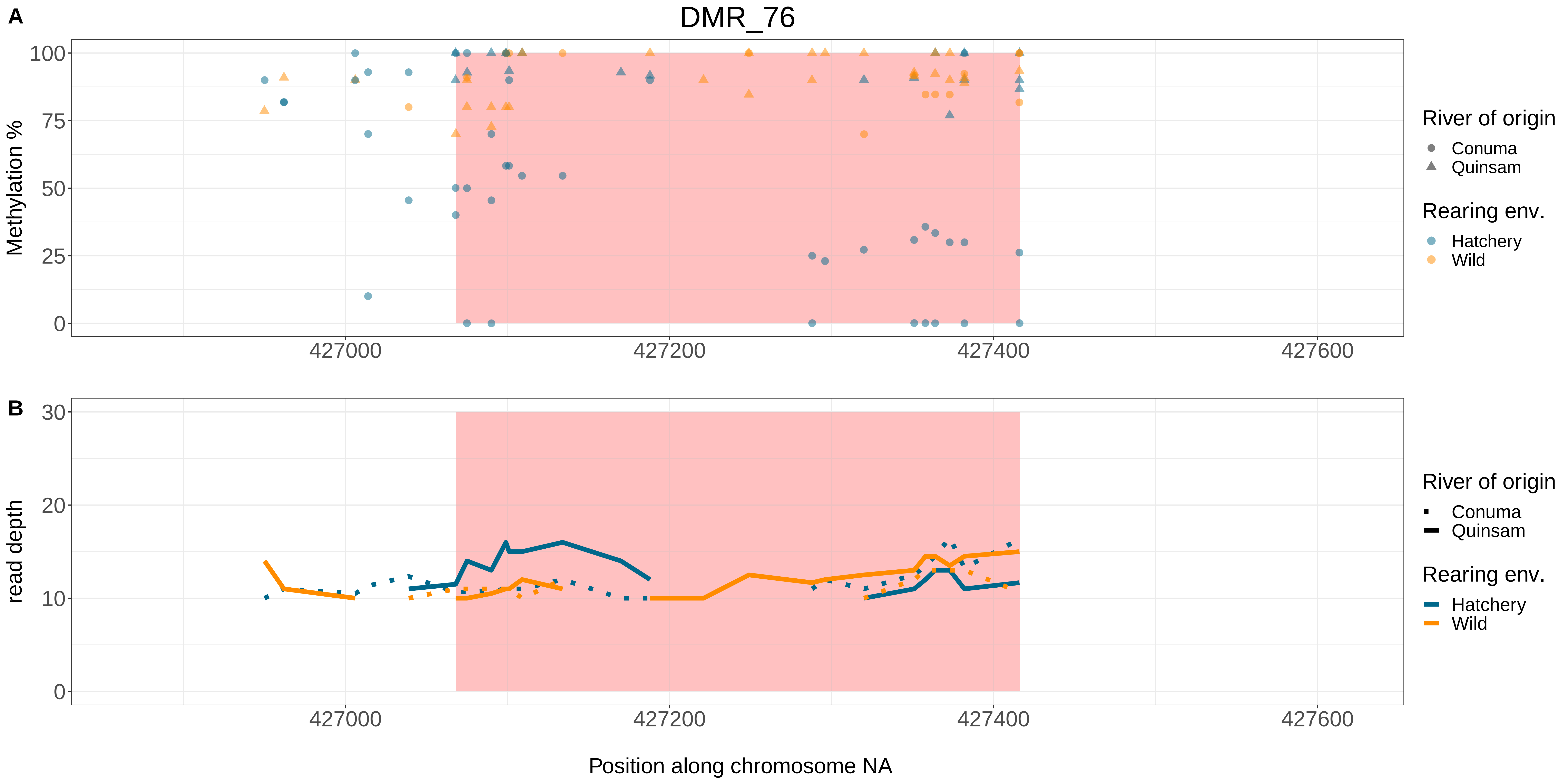

A
DMR_76
Methylation %
100
75
50
25
0
427000
427200
427400
427600
River of origin
Conuma
Quinsam
Rearing env.
Hatchery
Wild
B
read depth
30
20
10
0
427000
427200
427400
427600
River of origin
Conuma
Quinsam
Rearing env.
Hatchery
Wild
Position along chromosome NA

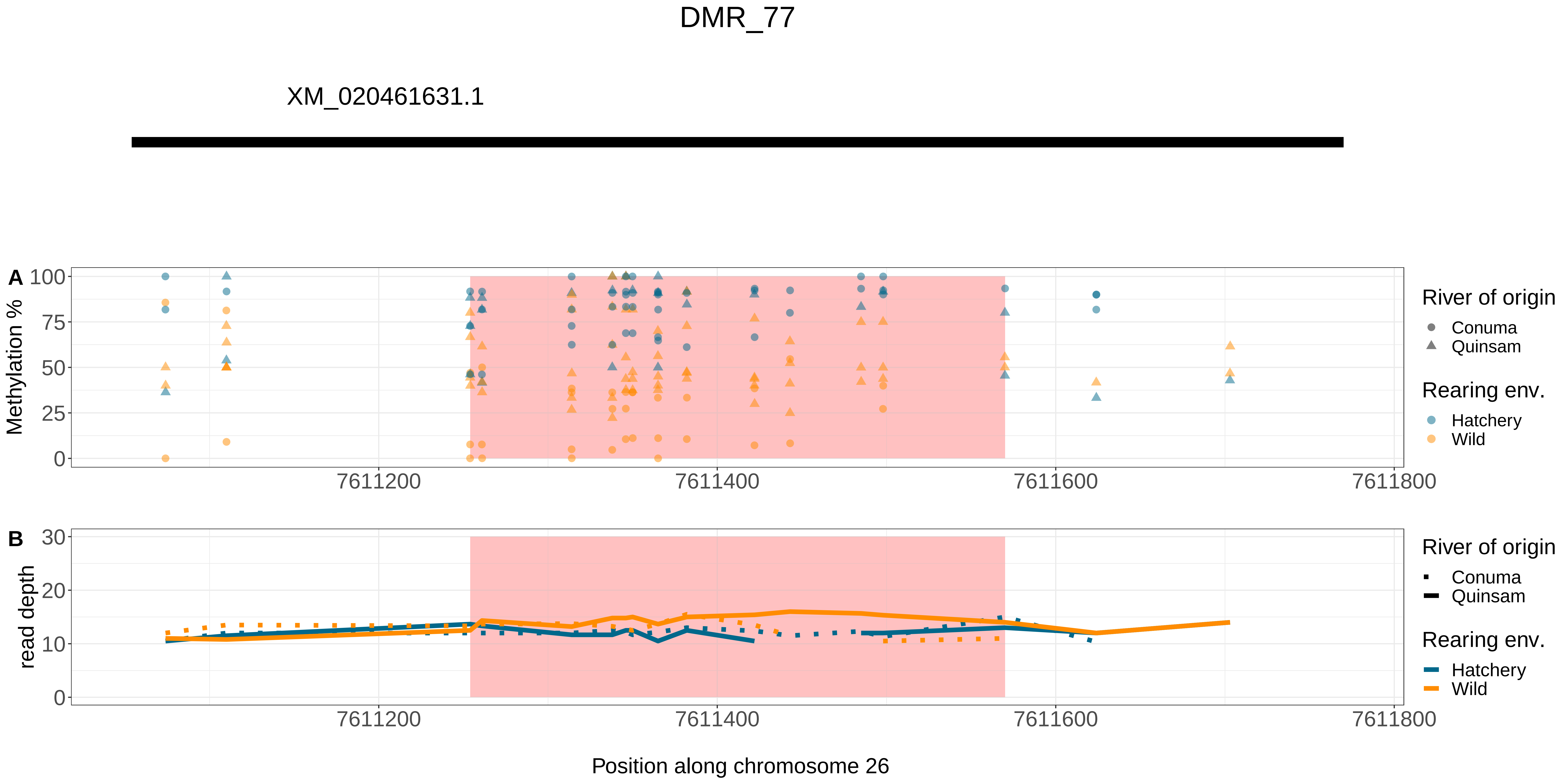
DMR_77
XM_020461631.1
A
Methylation %
100
75
50
25
0
7611200
7611400
7611600
7611800
River of origin
Conuma
Quinsam
Rearing env.
Hatchery
Wild
B
read depth
30
20
10
0
7611200
7611400
7611600
7611800
River of origin
Conuma
Quinsam
Rearing env.
Hatchery
Wild
Position along chromosome 26

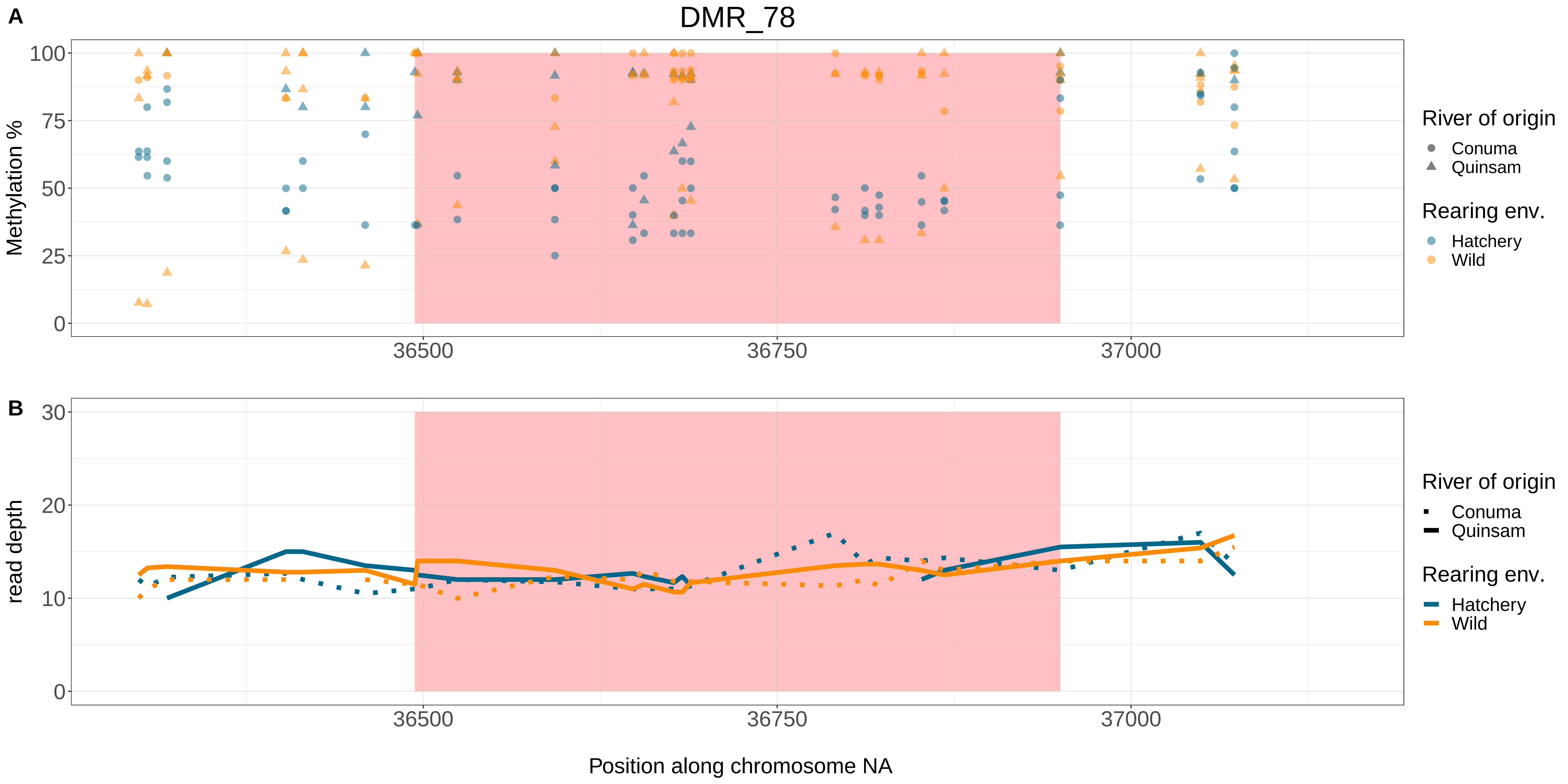

A
DMR_78
Methylation %
100
75
50
25
0
36500
36750
37000
River of origin
Conuma
Quinsam
Rearing env.
Hatchery
Wild
B
read depth
30
20
10
0
36500
36750
37000
River of origin
Conuma
Quinsam
Rearing env.
Hatchery
Wild
Position along chromosome NA

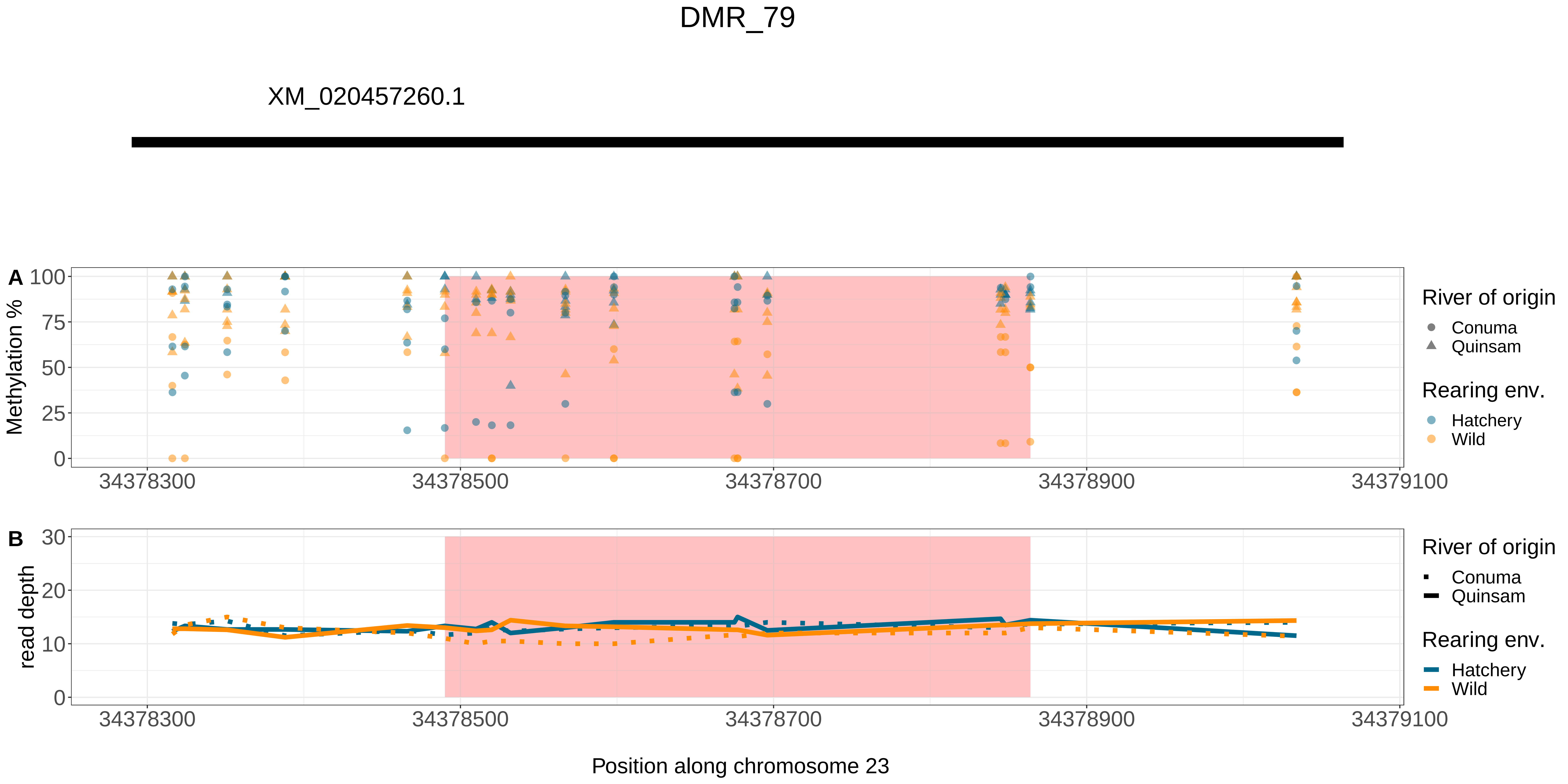

DMR_79
XM_020457260.1
A
B
Methylation %
read depth
100
75
50
25
0
30
20
10
0
34378300
34378500
34378700
34378900
34379100
Position along chromosome 23
River of origin
Conuma
Quinsam
Rearing env.
Hatchery
Wild

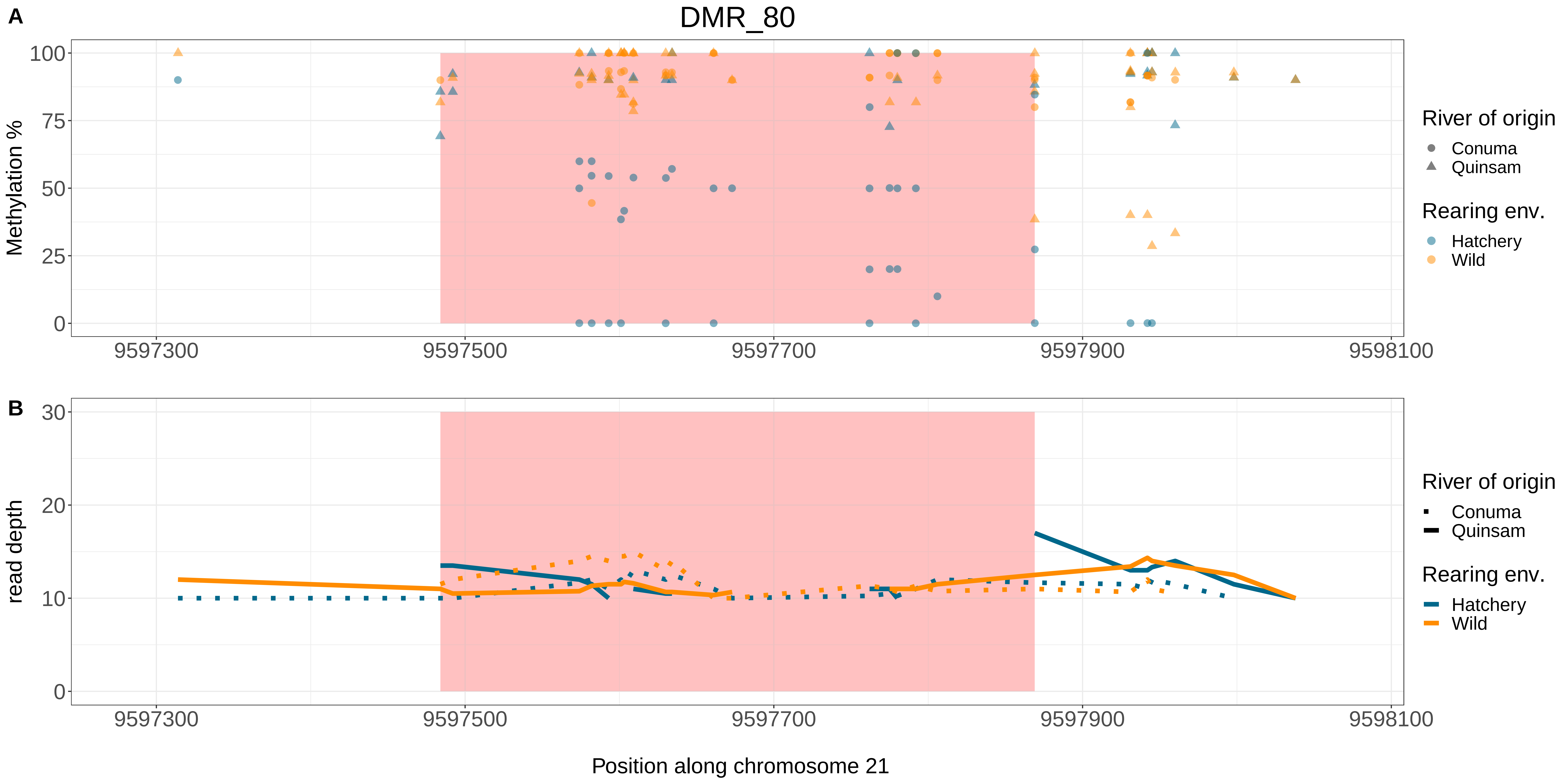
A
DMR_80
Methylation %
100
75
50
25
0
9597300
9597500
9597700
9597900
9598100
River of origin
Conuma
Quinsam
Rearing env.
Hatchery
Wild
B
read depth
30
20
10
0
9597300
9597500
9597700
9597900
9598100
River of origin
Conuma
Quinsam
Rearing env.
Hatchery
Wild
Position along chromosome 21

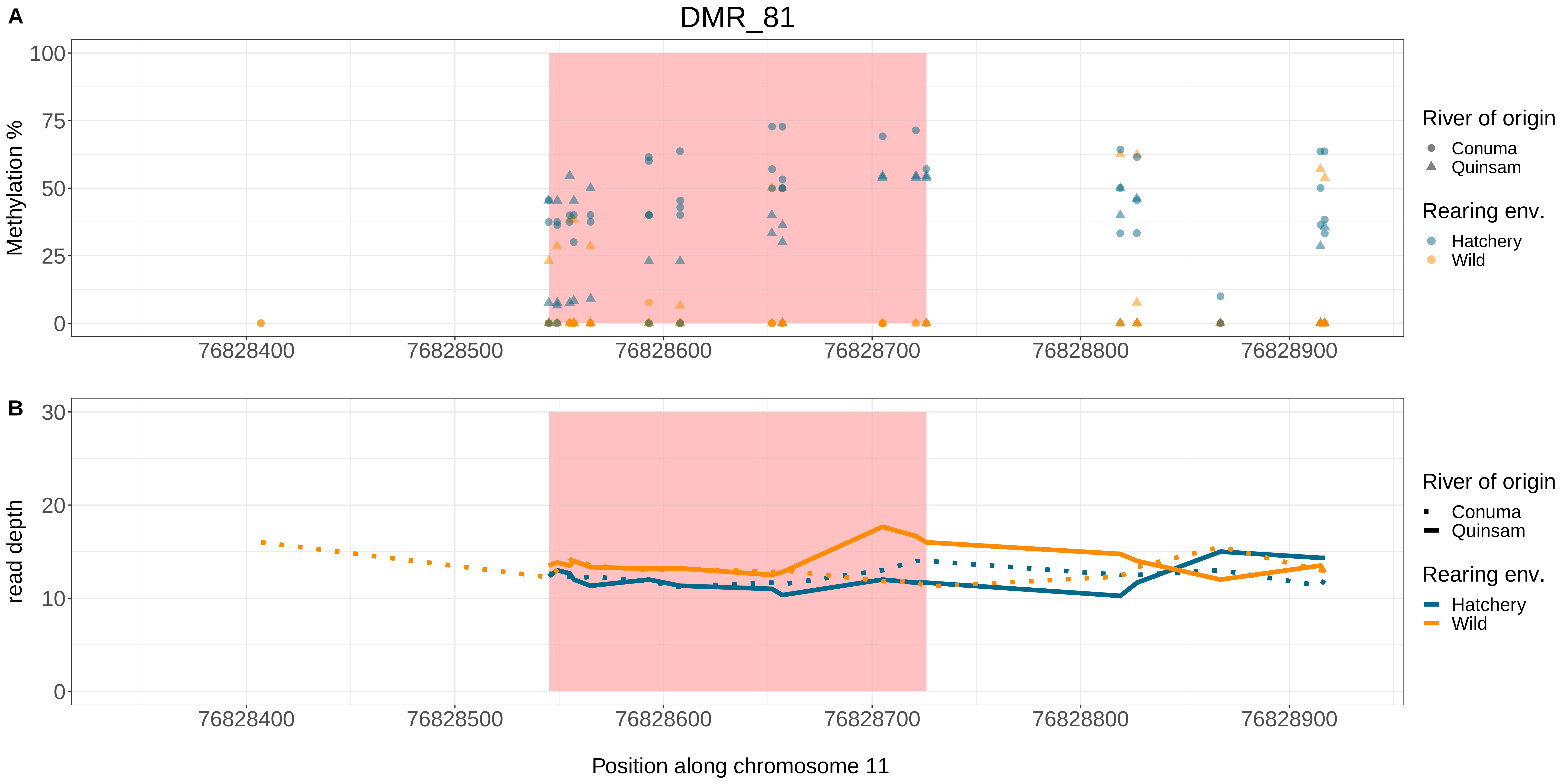
A
DMR_81
Methylation %
100
75
50
25
0
76828400
76828500
76828600
76828700
76828800
76828900
River of origin
Conuma
Quinsam
Rearing env.
Hatchery
Wild
B
read depth
30
20
10
0
76828400
76828500
76828600
76828700
76828800
76828900
River of origin
Conuma
Quinsam
Rearing env.
Hatchery
Wild
Position along chromosome 11

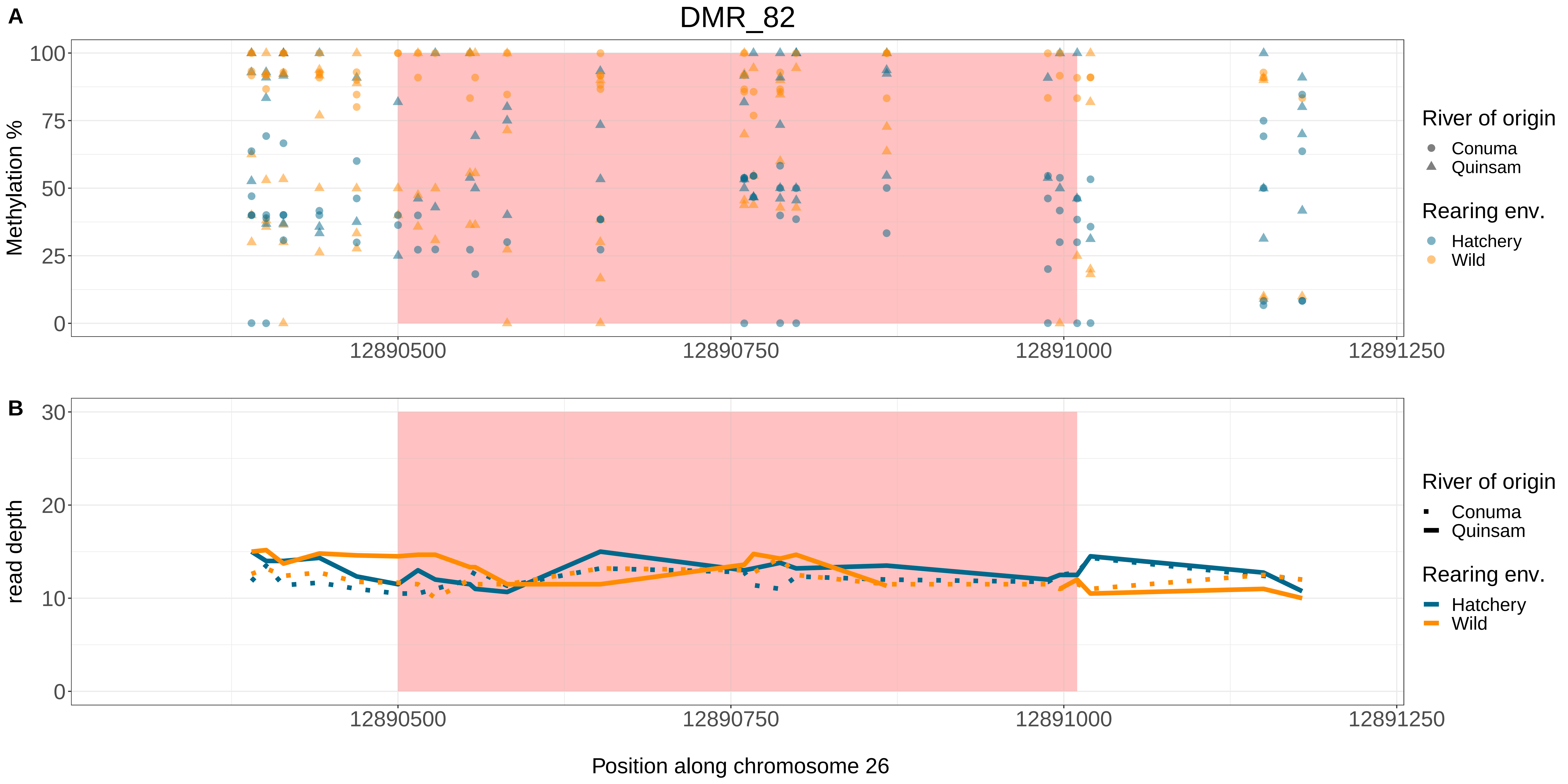

A
DMR_82
Methylation %
100
75
50
25
0
12890500
12890750
12891000
12891250
River of origin
Conuma
Quinsam
Rearing env.
Hatchery
Wild
B
read depth
30
20
10
0
12890500
12890750
12891000
12891250
River of origin
Conuma
Quinsam
Rearing env.
Hatchery
Wild
Position along chromosome 26

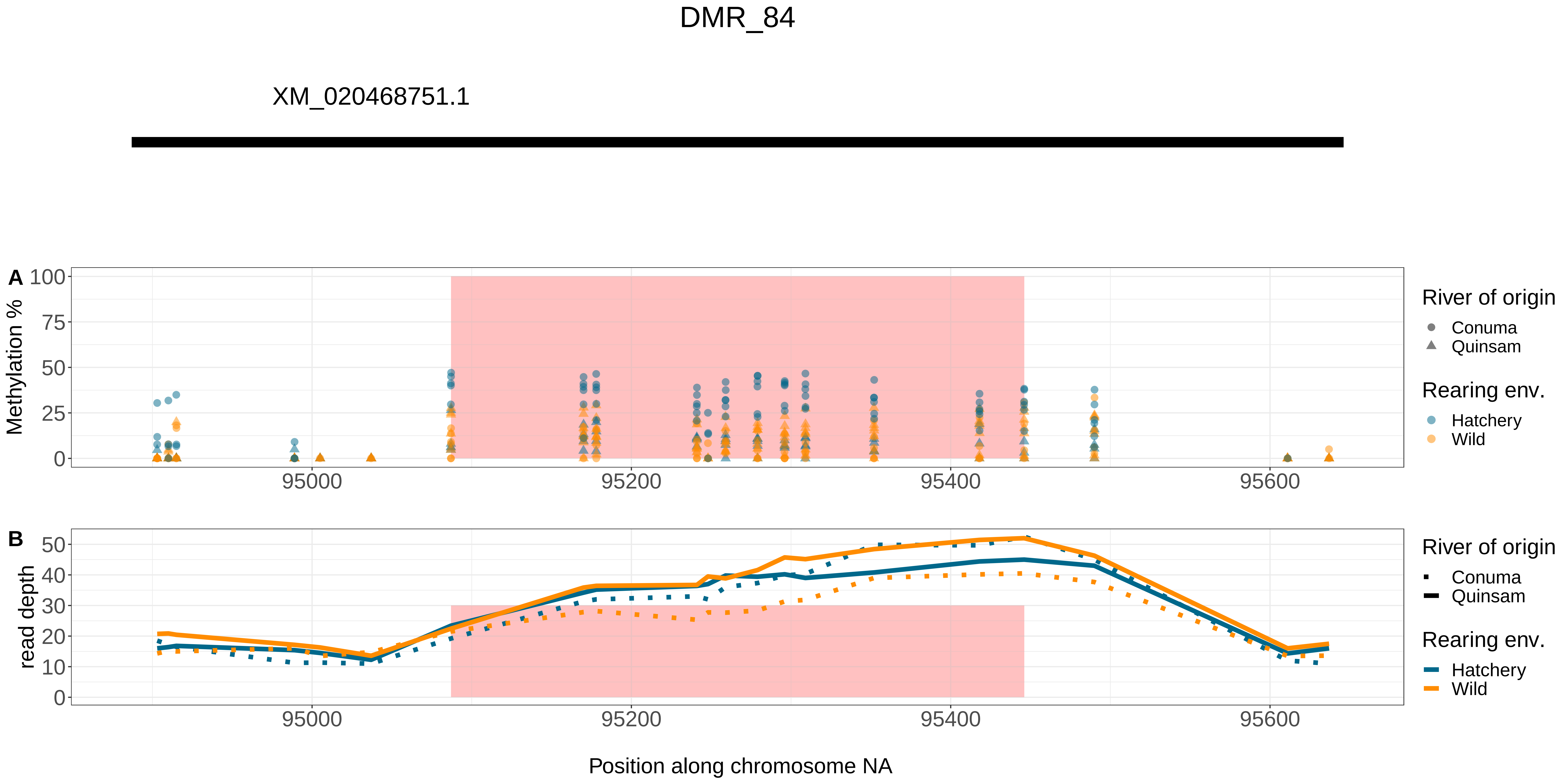

DMR_84
XM_020468751.1
A
Methylation %
100
75
50
25
0
95000
95200
95400
95600
River of origin
Conuma
Quinsam
Rearing env.
Hatchery
Wild
B
read depth
50
40
30
20
10
0
95000
95200
95400
95600
River of origin
Conuma
Quinsam
Rearing env.
Hatchery
Wild
Position along chromosome NA

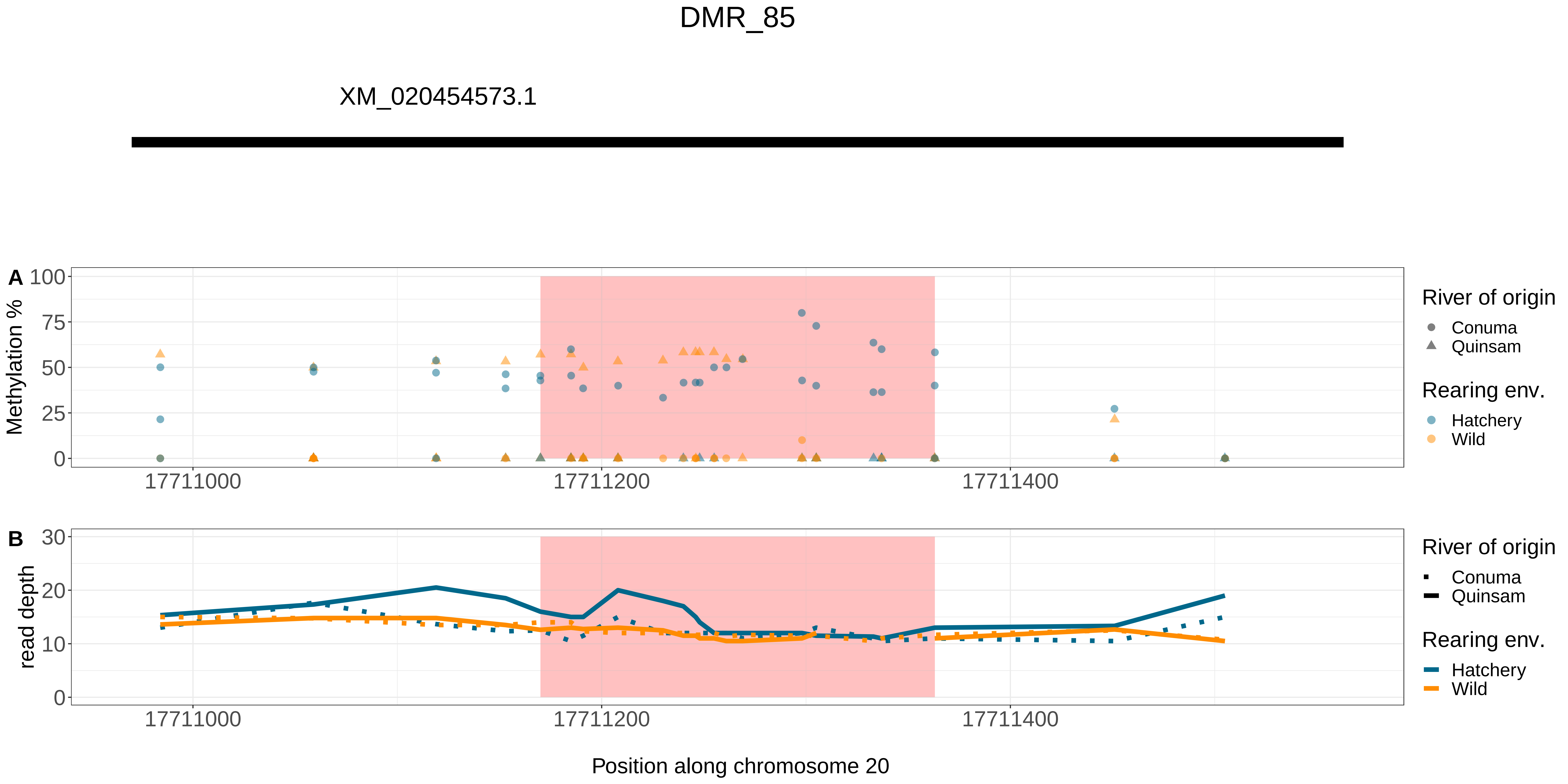

DMR_85
XM_020454573.1
A
Methylation %
100
75
50
25
0
17711000
17711200
17711400
River of origin
Conuma
Quinsam
Rearing env.
Hatchery
Wild
B
read depth
30
20
10
0
17711000
17711200
17711400
River of origin
Conuma
Quinsam
Rearing env.
Hatchery
Wild
Position along chromosome 20

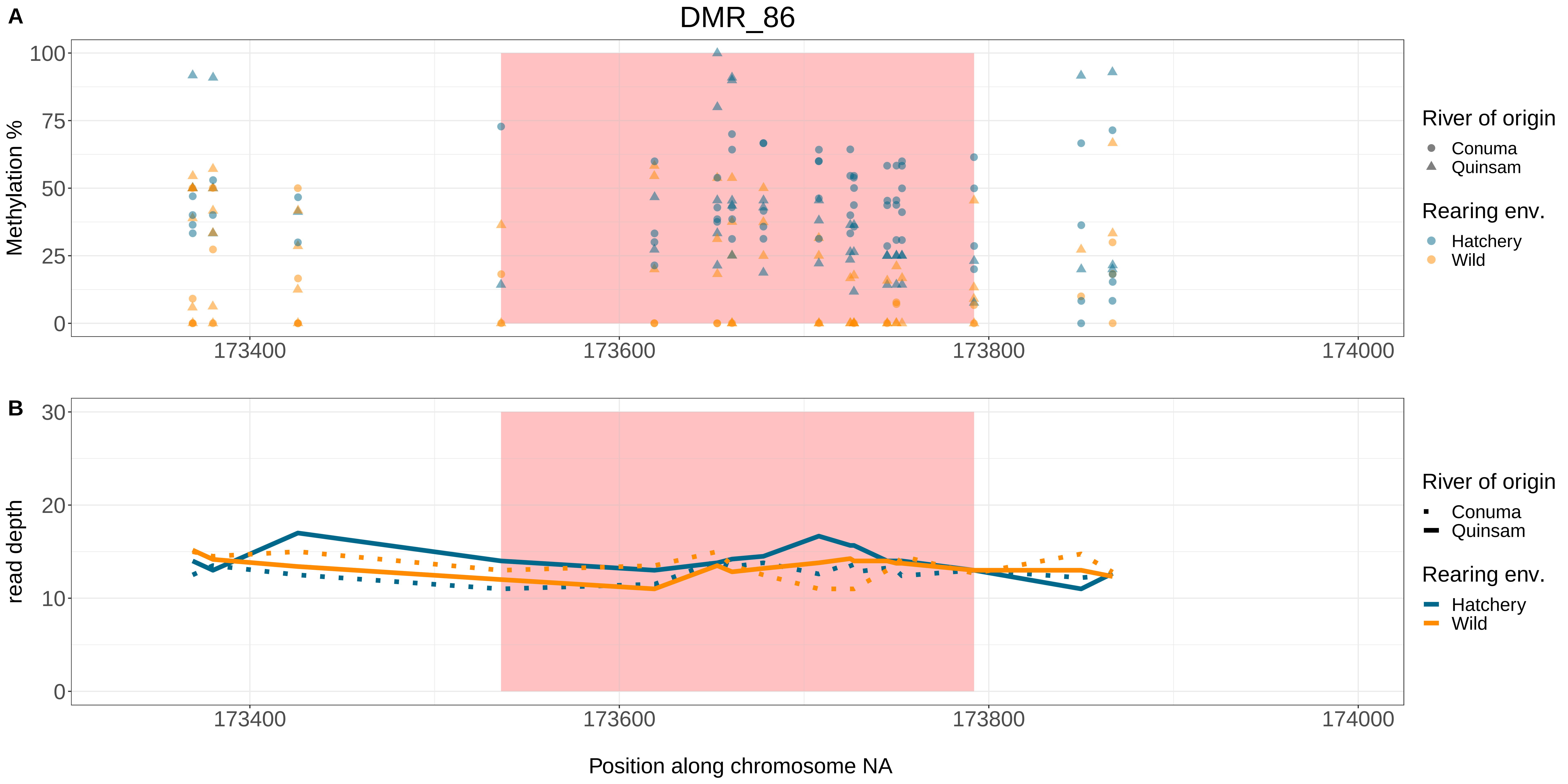

A
DMR_86
Methylation %
100
75
50
25
0
173400
173600
173800
174000
River of origin
Conuma
Quinsam
Rearing env.
Hatchery
Wild
B
read depth
30
20
10
0
173400
173600
173800
174000
River of origin
Conuma
Quinsam
Rearing env.
Hatchery
Wild
Position along chromosome NA

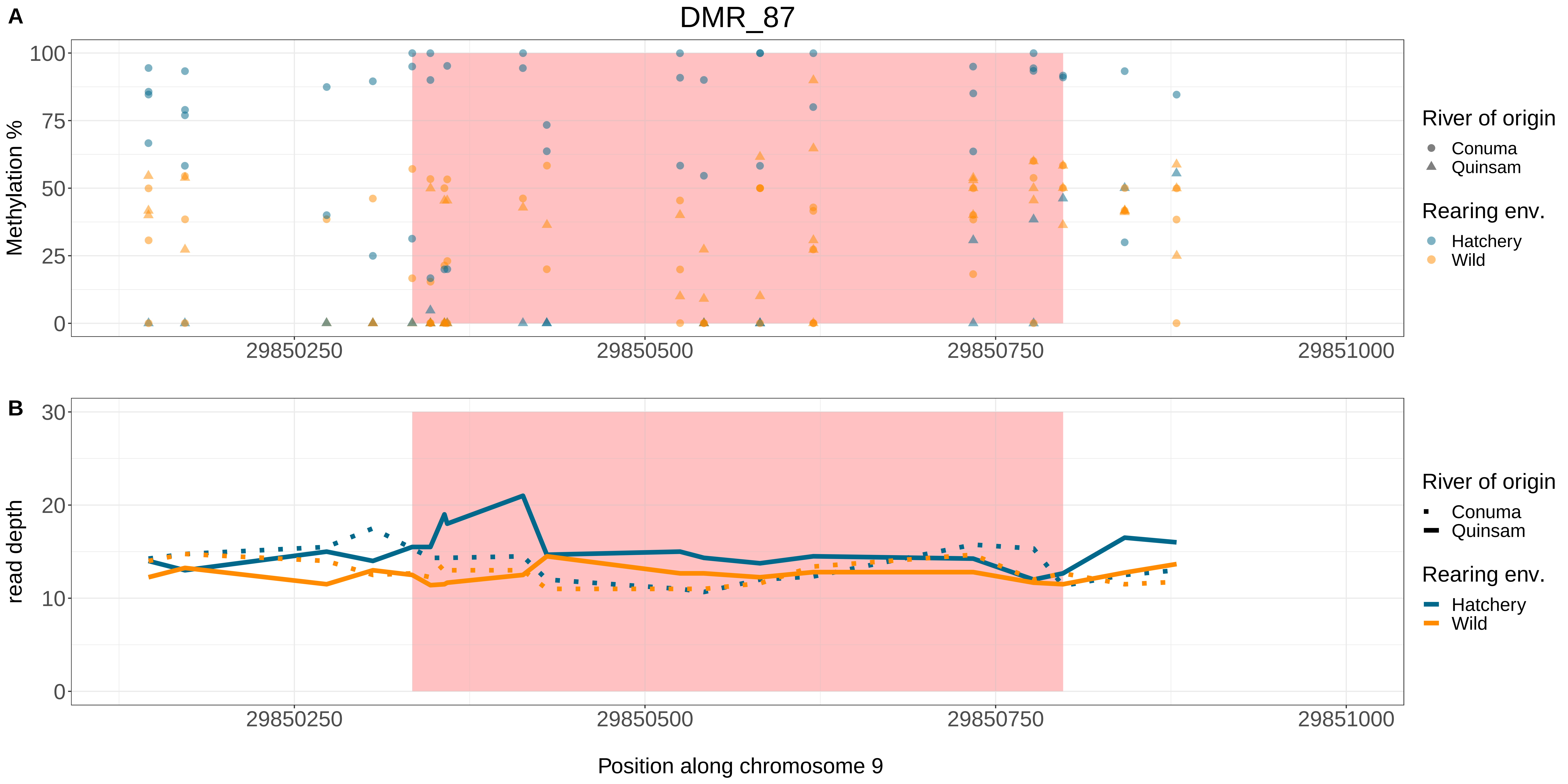
A
DMR_87
Methylation %
100
75
50
25
0
29850250
29850500
29850750
29851000
River of origin
Conuma
Quinsam
Rearing env.
Hatchery
Wild
B
read depth
30
20
10
0
29850250
29850500
29850750
29851000
River of origin
Conuma
Quinsam
Rearing env.
Hatchery
Wild
Position along chromosome 9

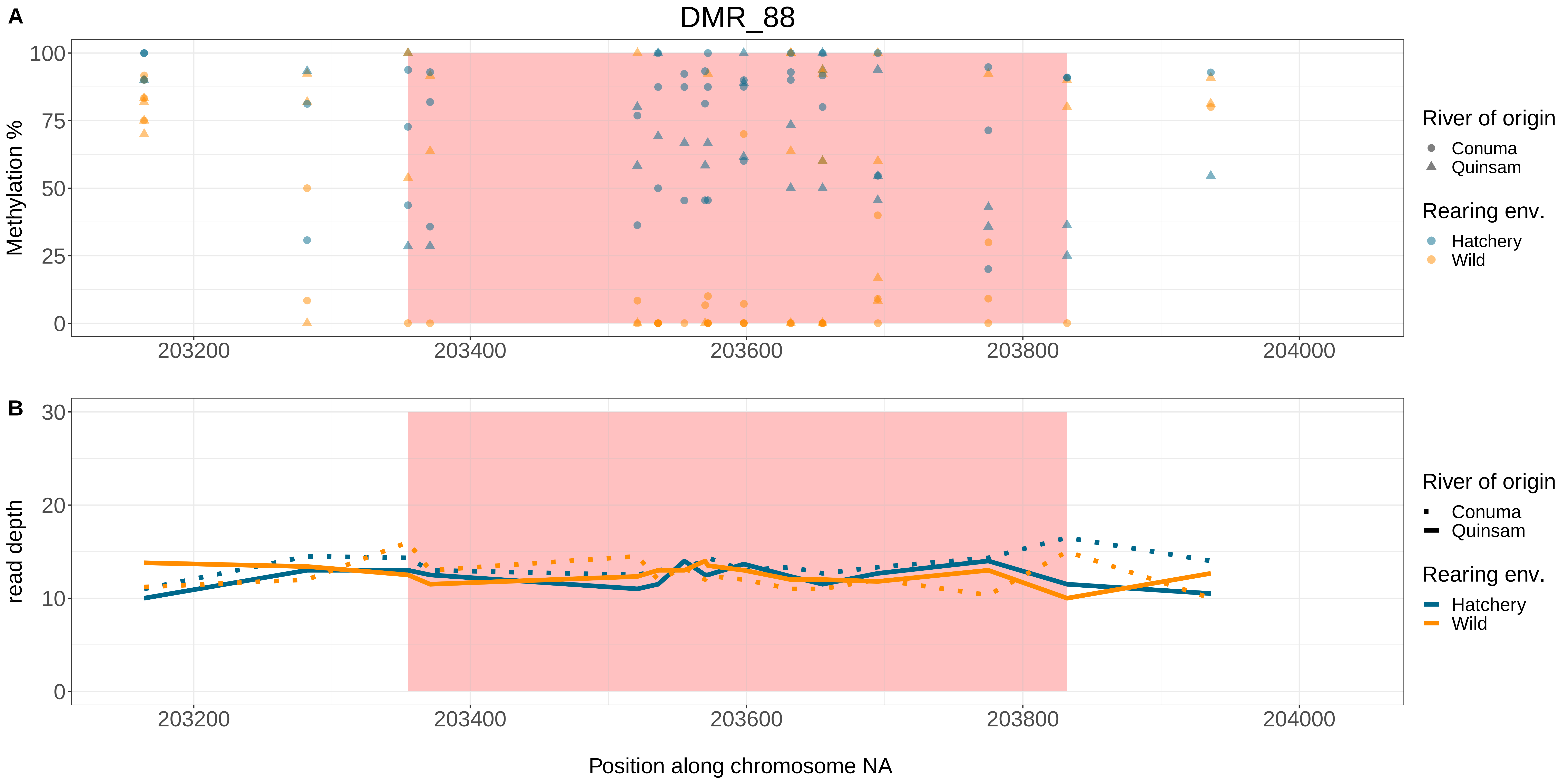

A
DMR_88
Methylation %
100
75
50
25
0
203200
203400
203600
203800
204000
River of origin
Conuma
Quinsam
Rearing env.
Hatchery
Wild
B
read depth
30
20
10
0
203200
203400
203600
203800
204000
River of origin
Conuma
Quinsam
Rearing env.
Hatchery
Wild
Position along chromosome NA

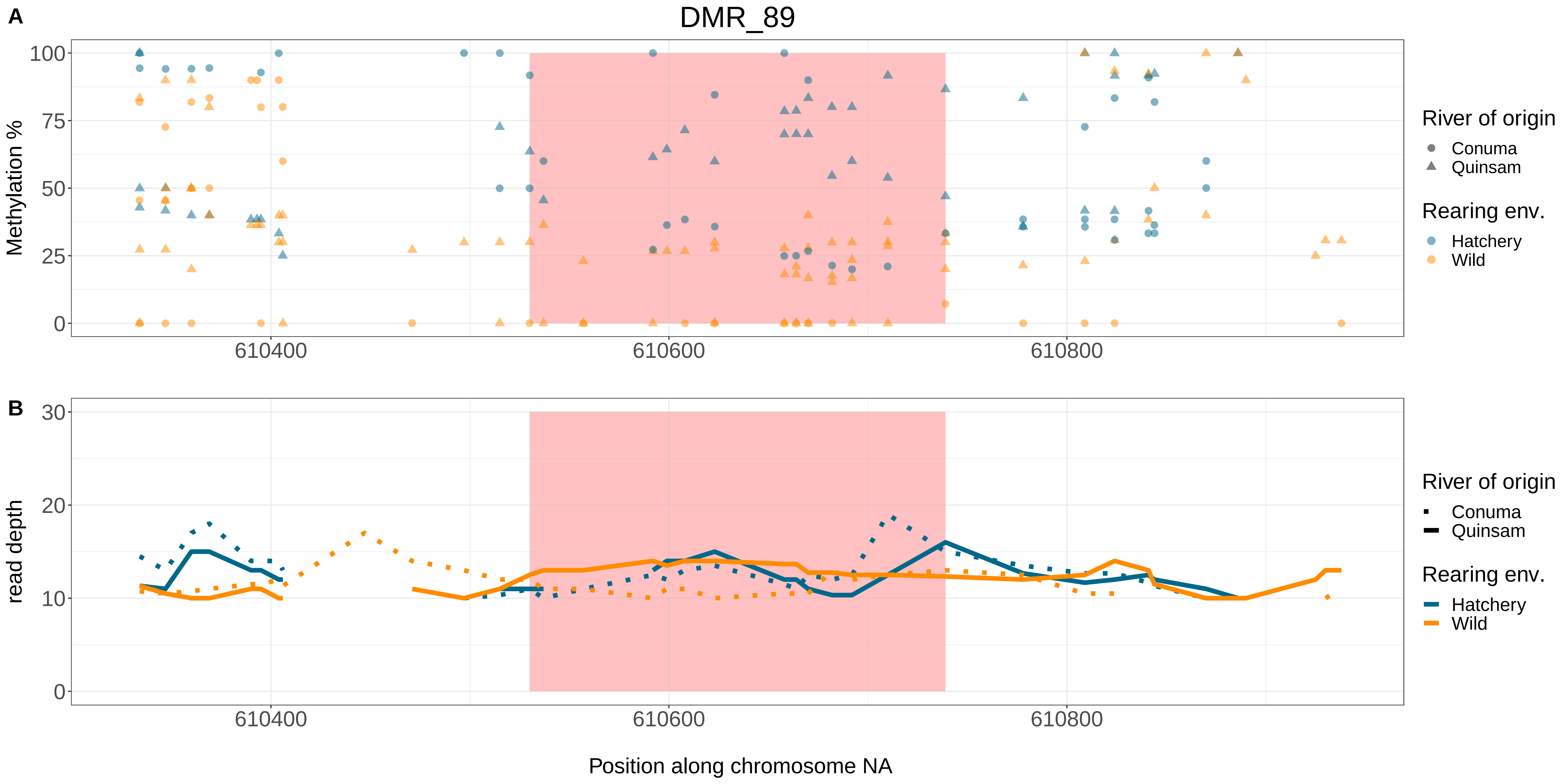

A
DMR_89
Methylation %
100
75
50
25
0
610400
610600
610800
River of origin
Conuma
Quinsam
Rearing env.
Hatchery
Wild
B
read depth
30
20
10
0
610400
610600
610800
River of origin
Conuma
Quinsam
Rearing env.
Hatchery
Wild
Position along chromosome NA

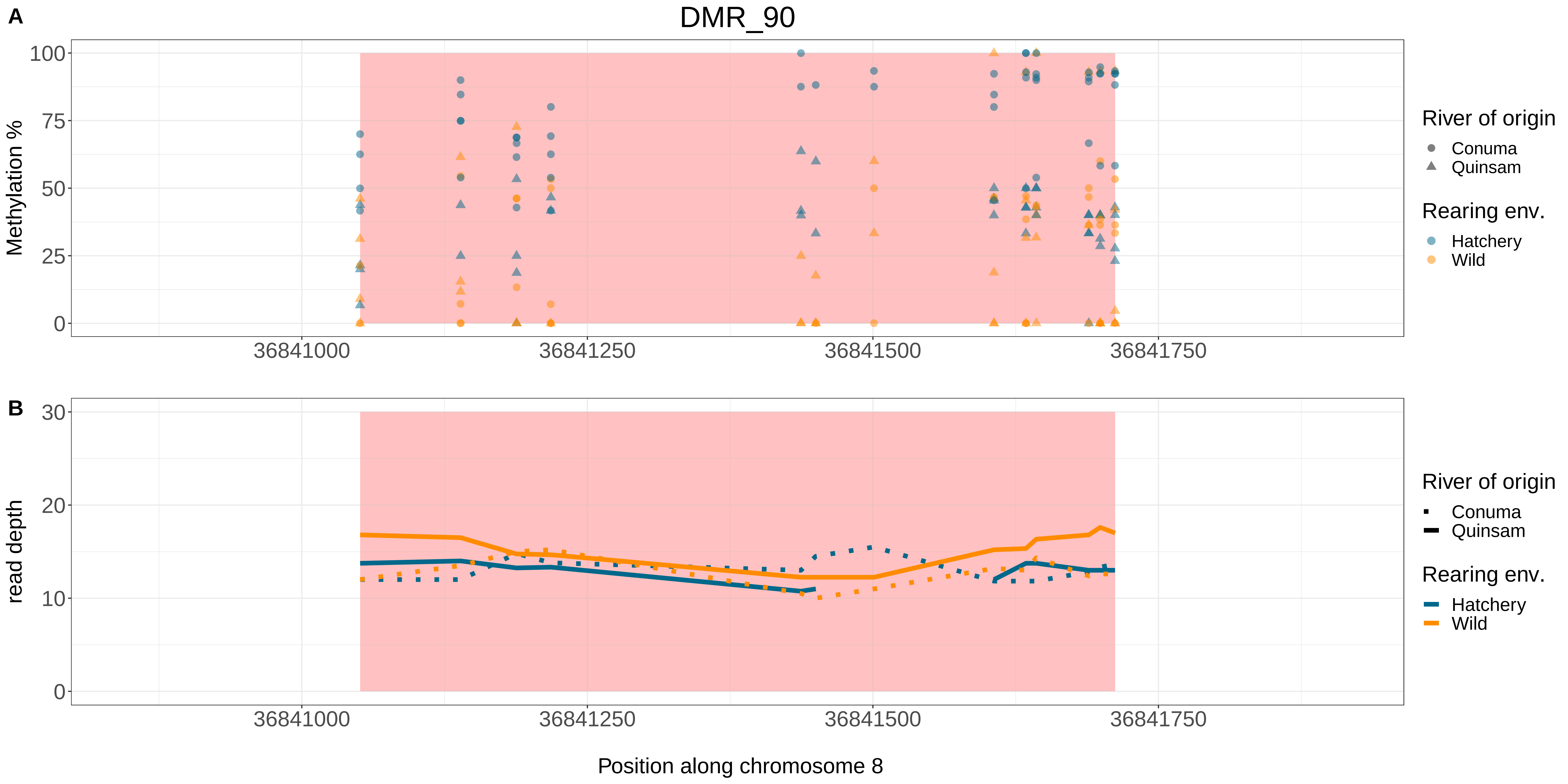

A
DMR_90
Methylation %
100
75
50
25
0
36841000
36841250
36841500
36841750
River of origin
Conuma
Quinsam
Rearing env.
Hatchery
Wild
B
read depth
30
20
10
0
36841000
36841250
36841500
36841750
River of origin
Conuma
Quinsam
Rearing env.
Hatchery
Wild
Position along chromosome 8

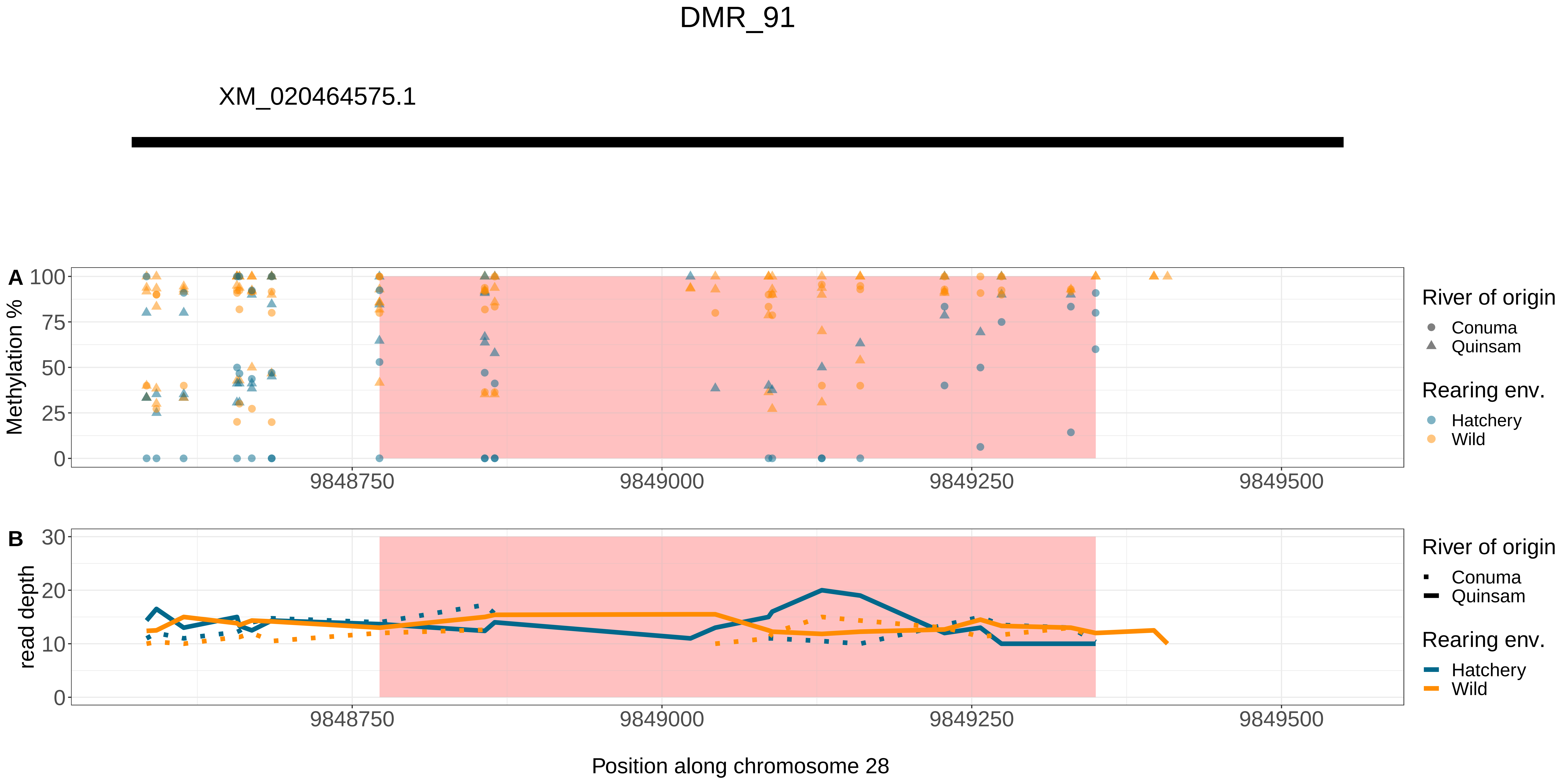

DMR_91
XM_020464575.1
A
Methylation %
100
75
50
25
0
9848750
9849000
9849250
9849500
River of origin
Conuma
Quinsam
Rearing env.
Hatchery
Wild
B
read depth
30
20
10
0
9848750
9849000
9849250
9849500
River of origin
Conuma
Quinsam
Rearing env.
Hatchery
Wild
Position along chromosome 28

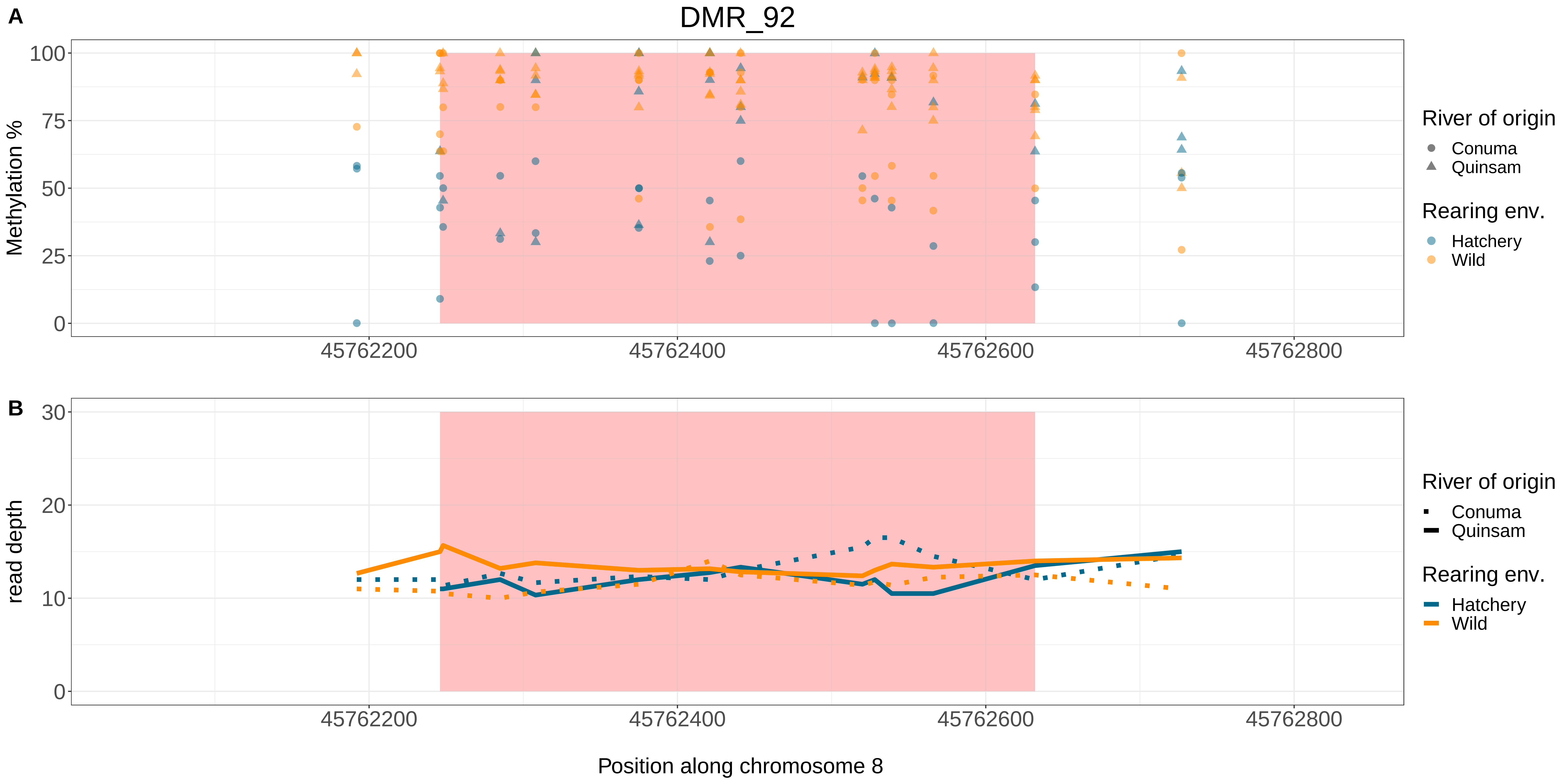

A
DMR_92
100
75
50
25
0
Methylation %
45762200
45762400
45762600
45762800
River of origin
Conuma
Quinsam
Rearing env.
Hatchery
Wild
B
30
20
10
0
read depth
45762200
45762400
45762600
45762800
River of origin
Conuma
Quinsam
Rearing env.
Hatchery
Wild
Position along chromosome 8

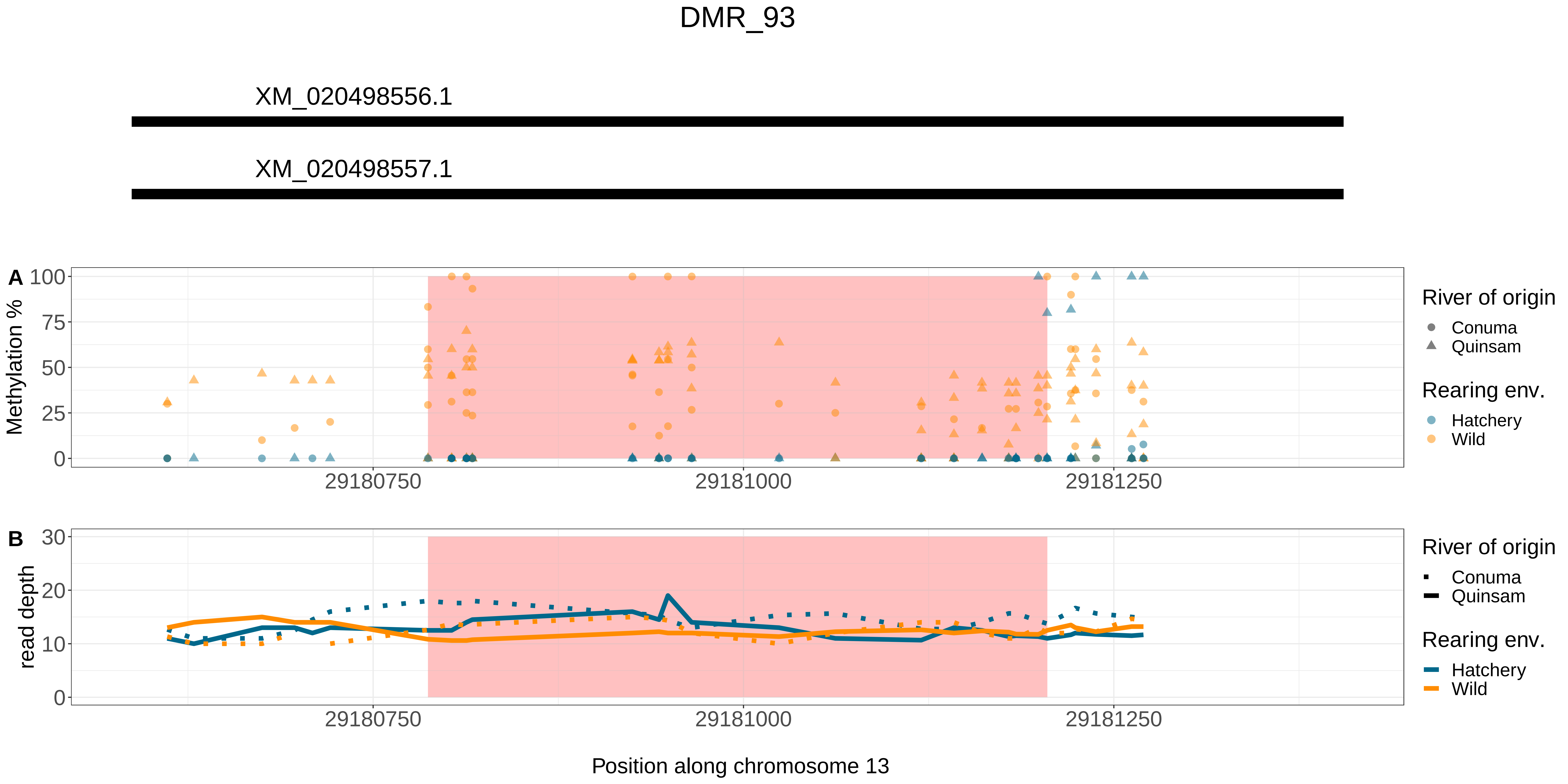

DMR_93
XM_020498556.1
XM_020498557.1
A
Methylation %
100
75
50
25
0
29180750
29181000
29181250
River of origin
Conuma
Quinsam
Rearing env.
Hatchery
Wild
B
read depth
30
20
10
0
29180750
29181000
29181250
River of origin
Conuma
Quinsam
Rearing env.
Hatchery
Wild
Position along chromosome 13

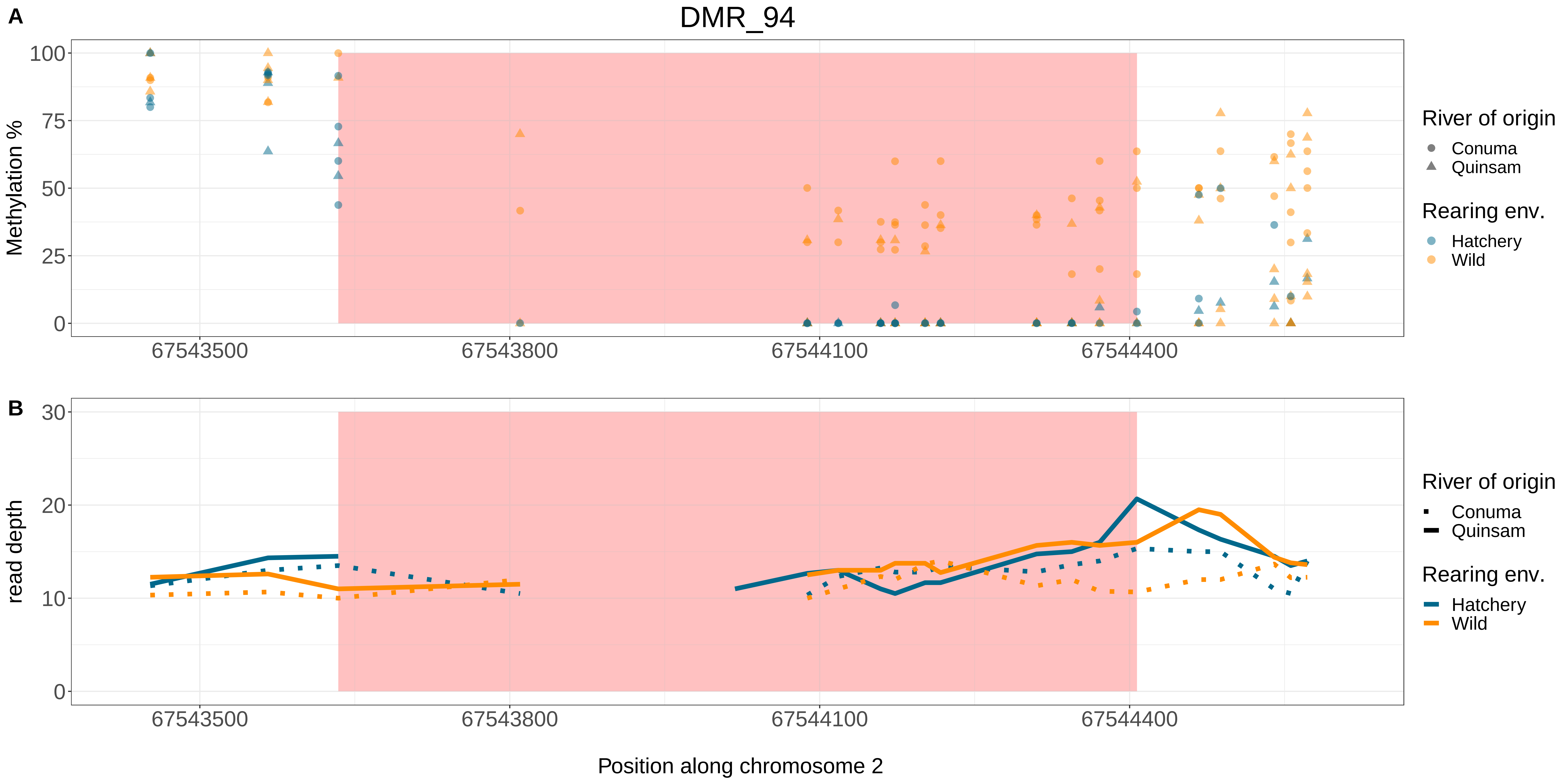

A
DMR_94
Methylation %
100
75
50
25
0
67543500
67543800
67544100
67544400
River of origin
Conuma
Quinsam
Rearing env.
Hatchery
Wild
B
read depth
30
20
10
0
67543500
67543800
67544100
67544400
River of origin
Conuma
Quinsam
Rearing env.
Hatchery
Wild
Position along chromosome 2

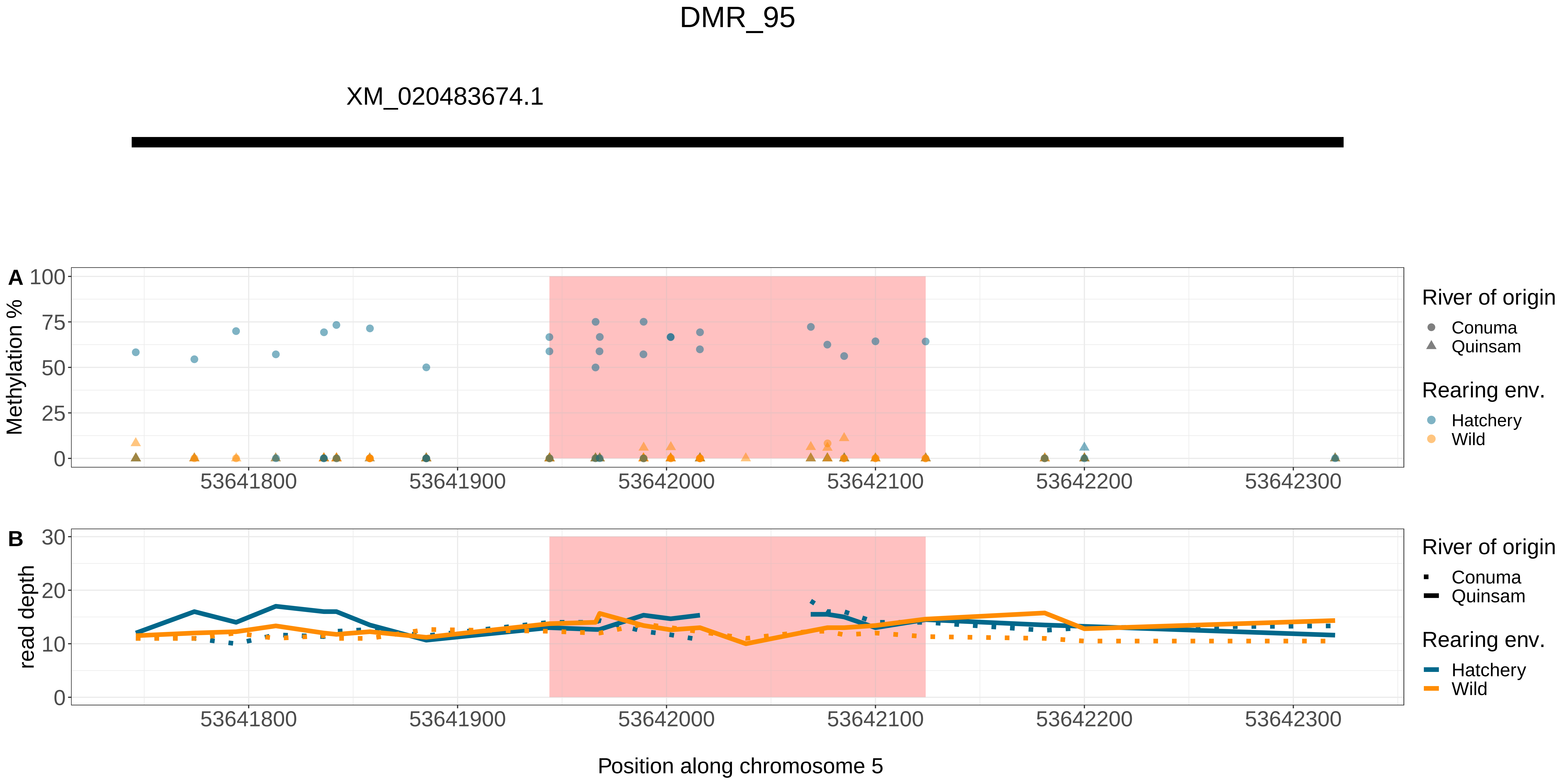

DMR_95
XM_020483674.1
A
Methylation %
100
75
50
25
0
53641800
53641900
53642000
53642100
53642200
53642300
River of origin
Conuma
Quinsam
Rearing env.
Hatchery
Wild
B
read depth
30
20
10
0
53641800
53641900
53642000
53642100
53642200
53642300
River of origin
Conuma
Quinsam
Rearing env.
Hatchery
Wild
Position along chromosome 5

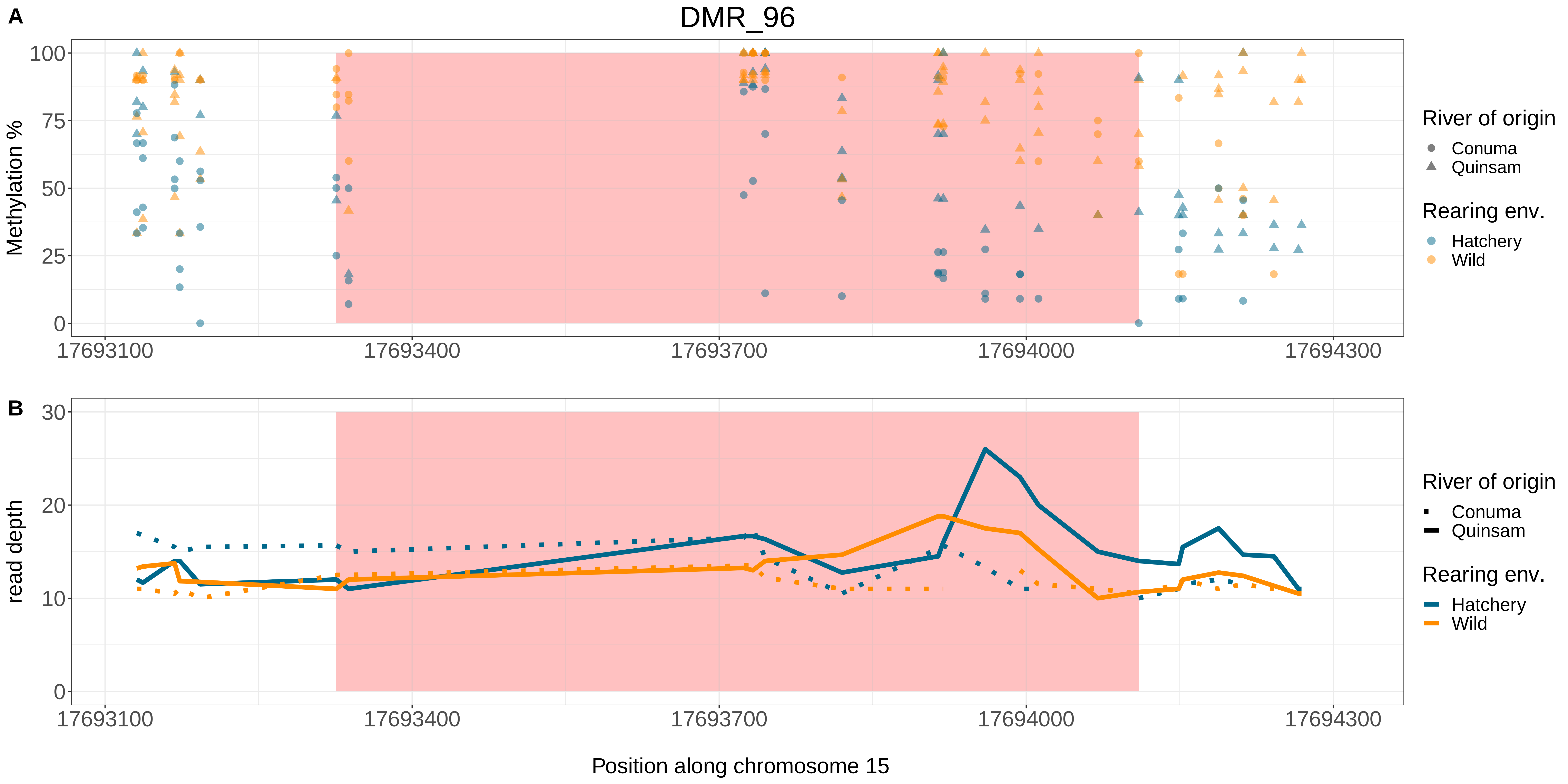

A
DMR_96
Methylation %
100
75
50
25
0
17693100
17693400
17693700
17694000
17694300
River of origin
Conuma
Quinsam
Rearing env.
Hatchery
Wild
B
read depth
30
20
10
0
17693100
17693400
17693700
17694000
17694300
River of origin
Conuma
Quinsam
Rearing env.
Hatchery
Wild
Position along chromosome 15

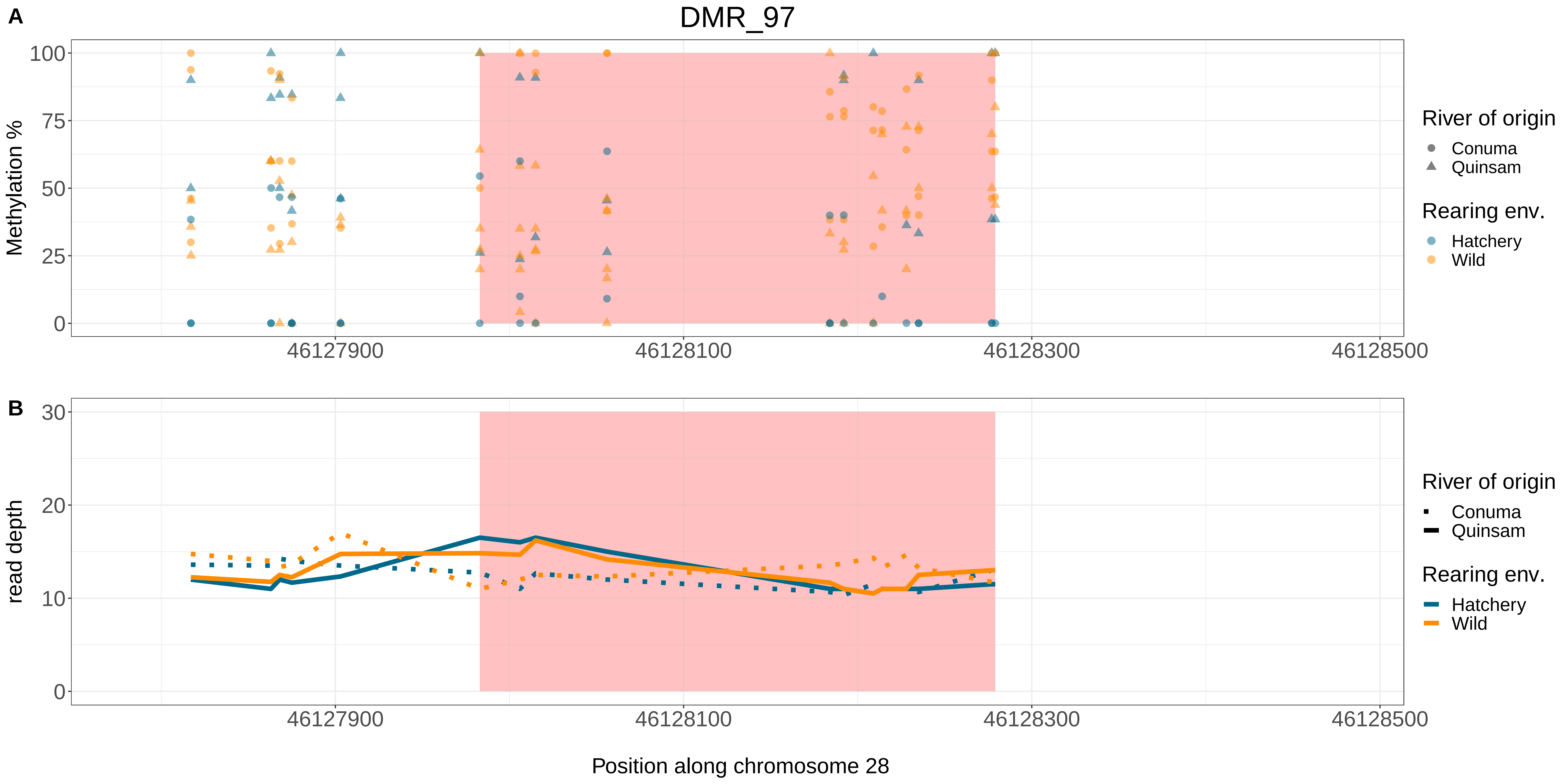

A
DMR_97
Methylation %
100
75
50
25
0
46127900
46128100
46128300
46128500
River of origin
Conuma
Quinsam
Rearing env.
Hatchery
Wild
B
read depth
30
20
10
0
46127900
46128100
46128300
46128500
River of origin
Conuma
Quinsam
Rearing env.
Hatchery
Wild
Position along chromosome 28

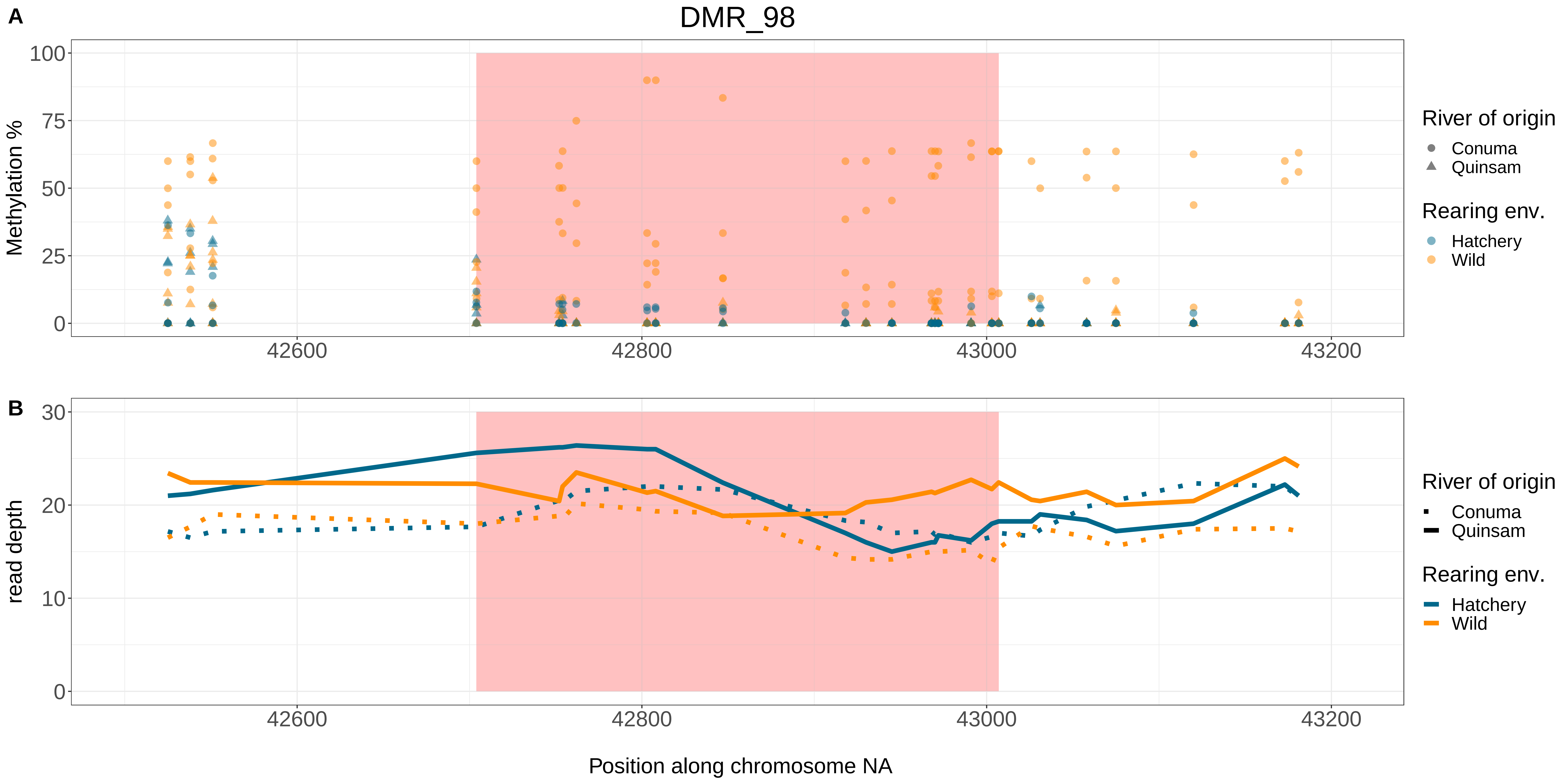
A
DMR_98
Methylation %
100
75
50
25
0
42600
42800
43000
43200
River of origin
Conuma
Quinsam
Rearing env.
Hatchery
Wild
B
read depth
30
20
10
0
42600
42800
43000
43200
River of origin
Conuma
Quinsam
Rearing env.
Hatchery
Wild
Position along chromosome NA

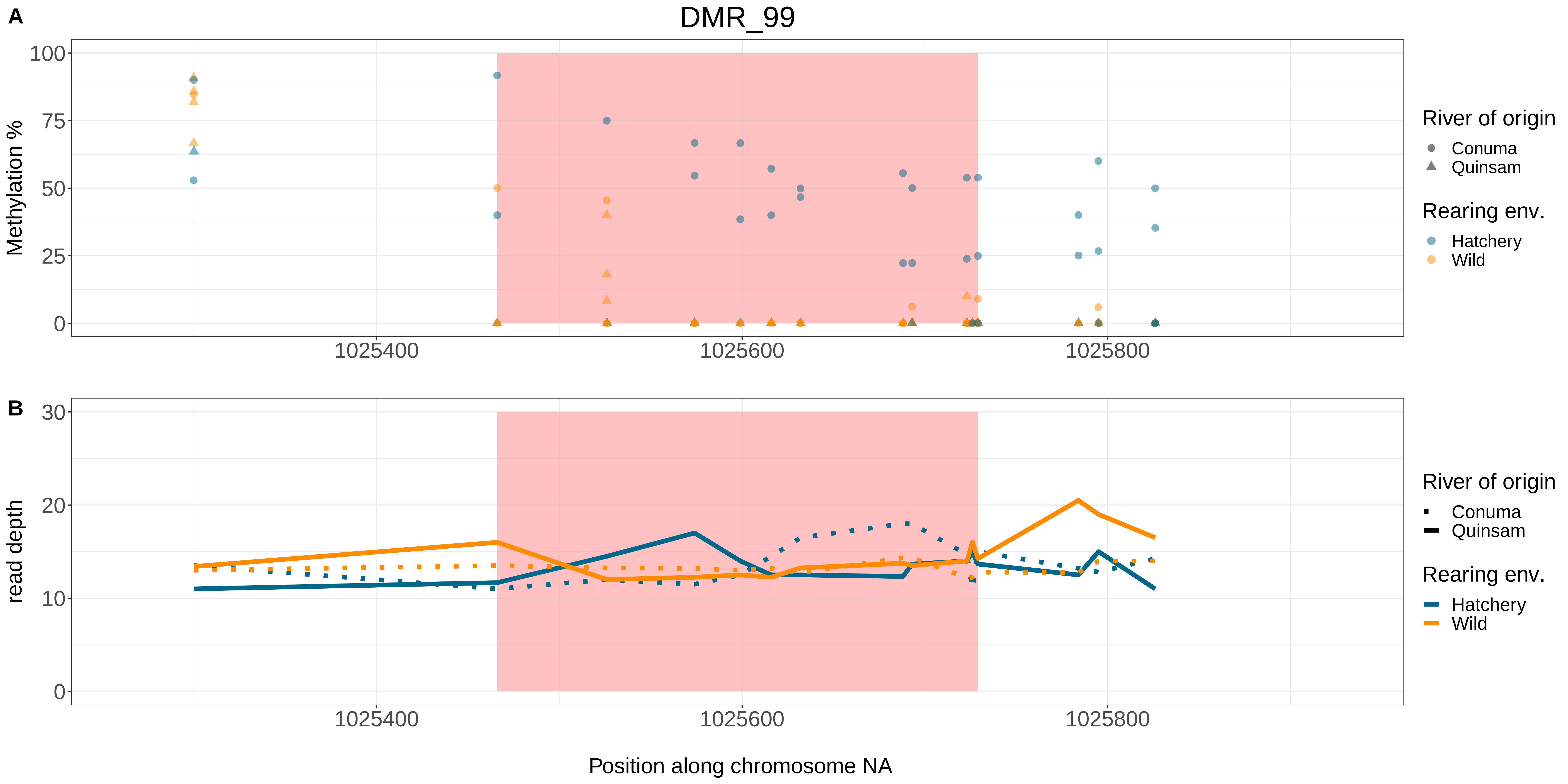
A
DMR_99
Methylation %
100
75
50
25
0
1025400
1025600
1025800
River of origin
Conuma
Quinsam
Rearing env.
Hatchery
Wild
B
read depth
30
20
10
0
1025400
1025600
1025800
River of origin
Conuma
Quinsam
Rearing env.
Hatchery
Wild
Position along chromosome NA

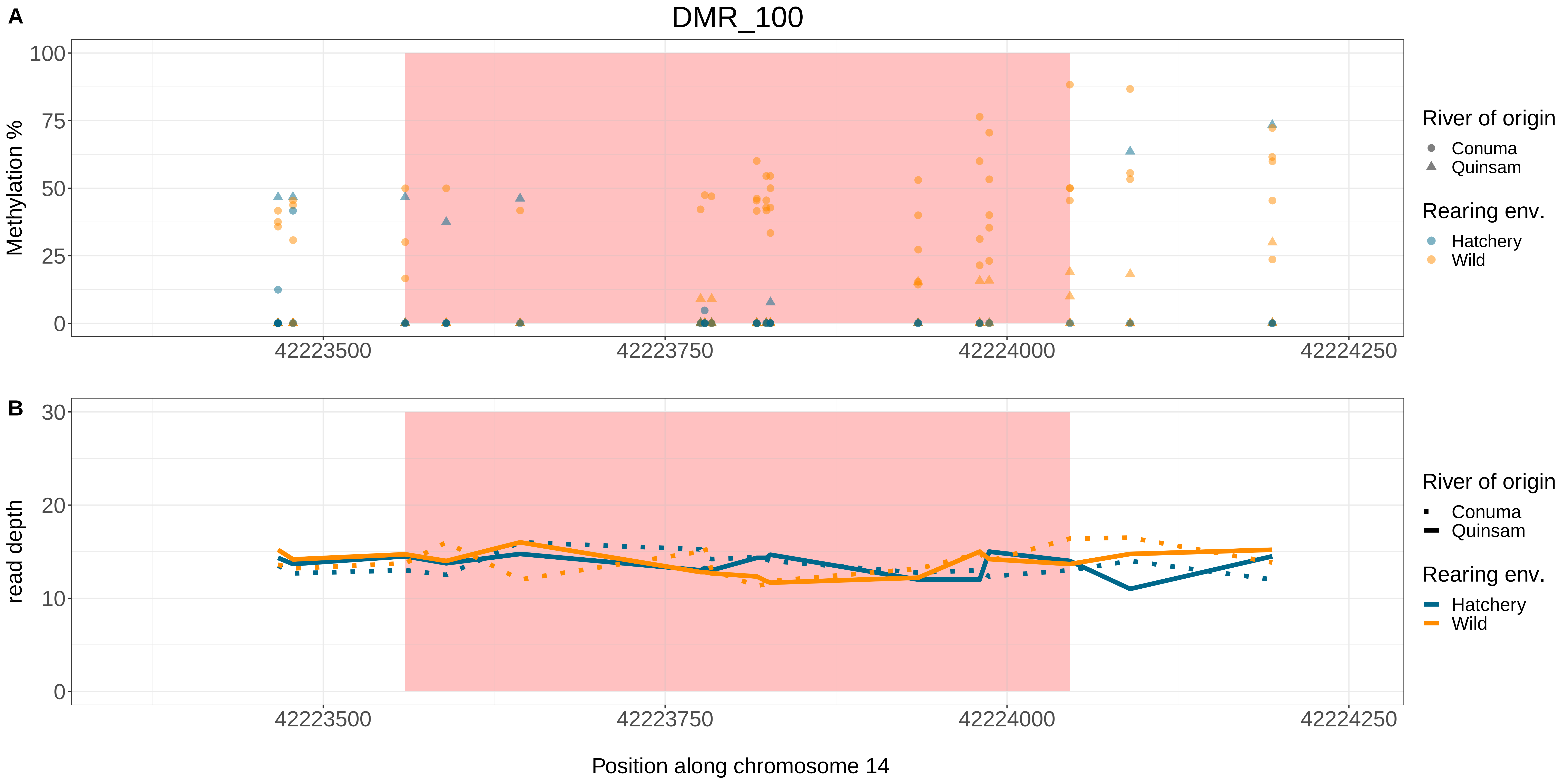
A
DMR_100
Methylation %
100
75
50
25
0
42223500
42223750
42224000
42224250
River of origin
Conuma
Quinsam
Rearing env.
Hatchery
Wild
B
read depth
30
20
10
0
Position along chromosome 14

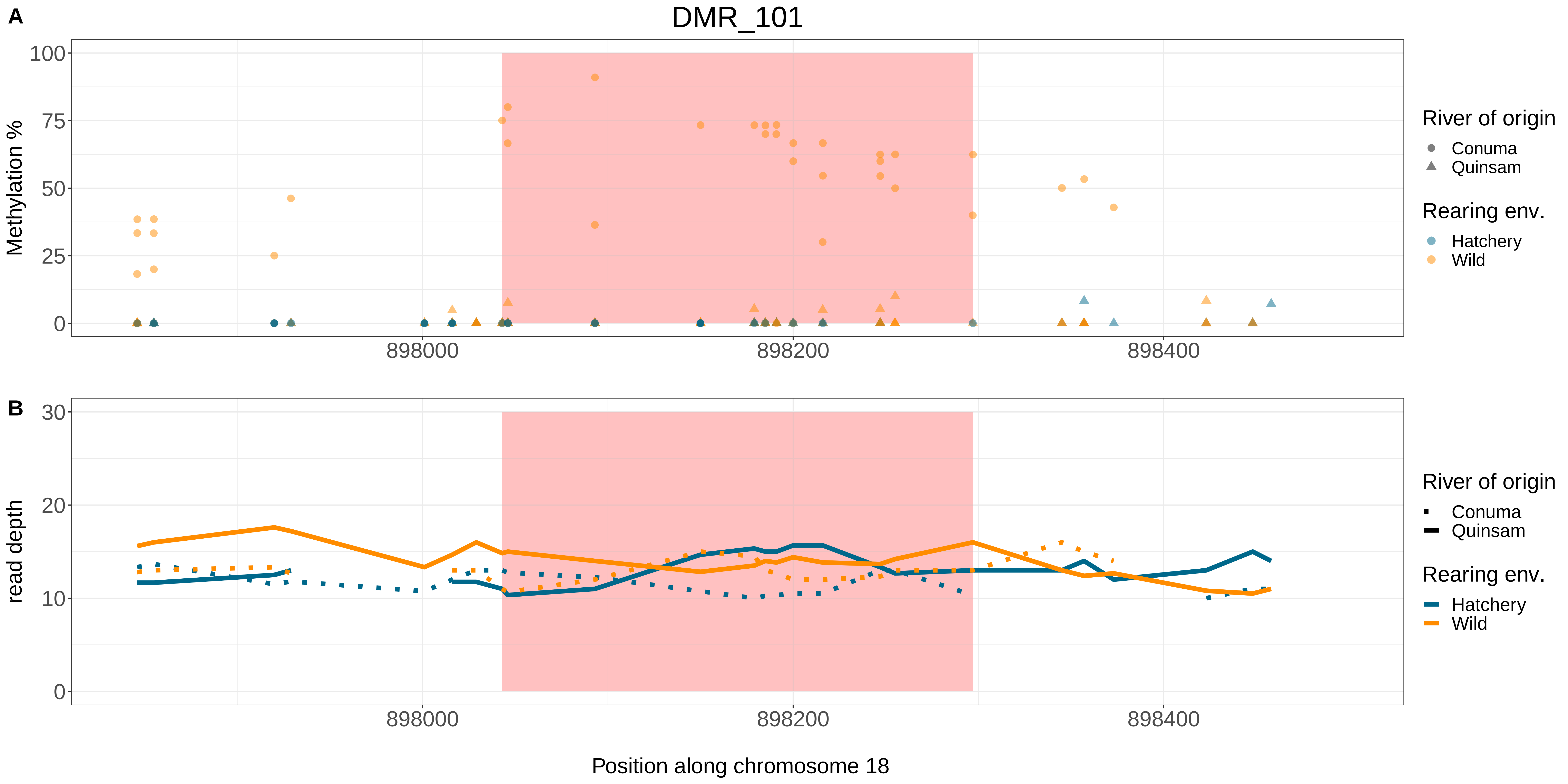

A
DMR_101
Methylation %
100
75
50
25
0
898000
898200
898400
River of origin
Conuma
Quinsam
Rearing env.
Hatchery
Wild
B
read depth
30
20
10
0
898000
898200
898400
River of origin
Conuma
Quinsam
Rearing env.
Hatchery
Wild
Position along chromosome 18

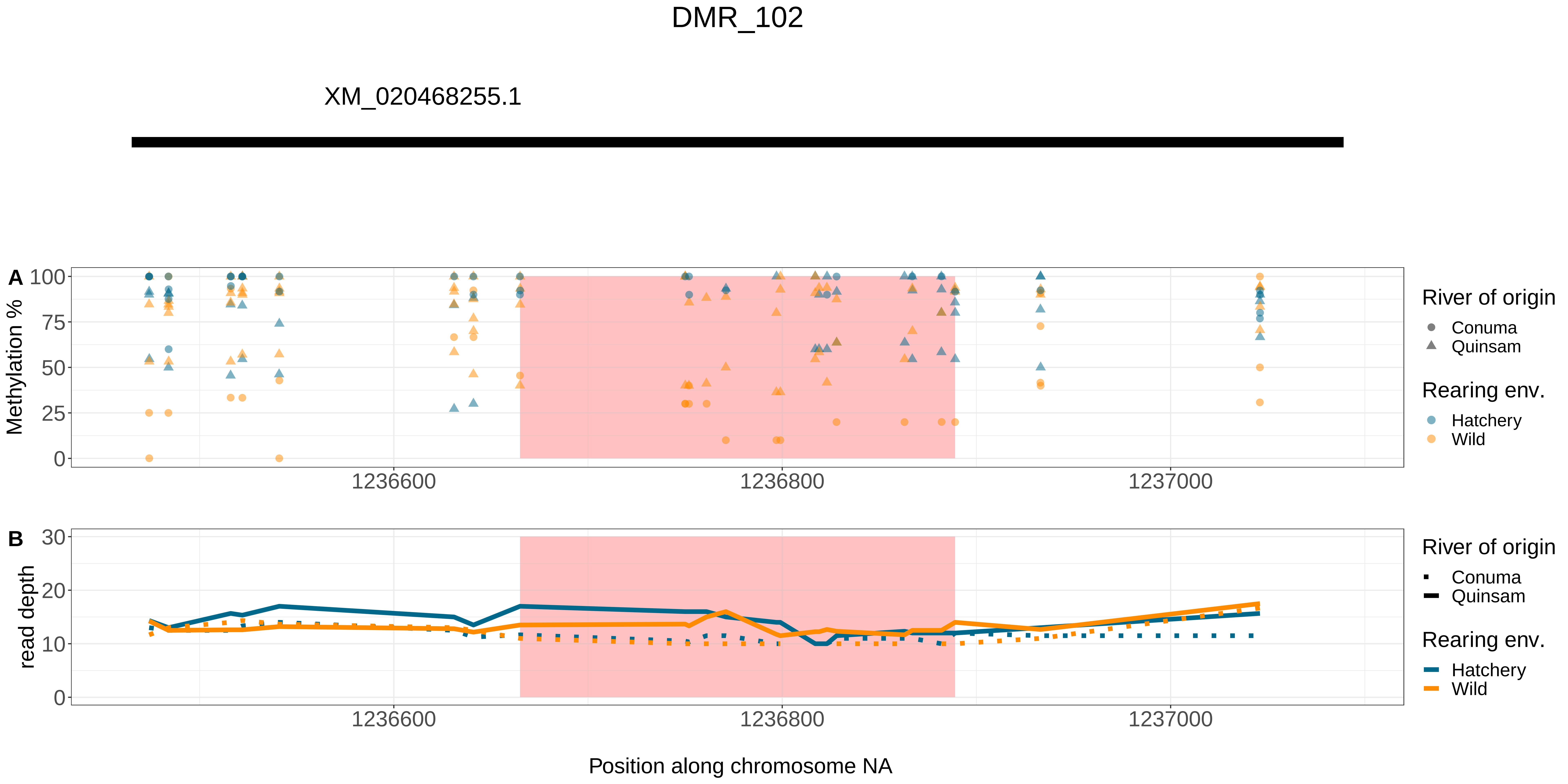

DMR_102
XM_020468255.1
A
Methylation %
100
75
50
25
0
1236600
1236800
1237000
River of origin
Conuma
Quinsam
Rearing env.
Hatchery
Wild
B
read depth
30
20
10
0
1236600
1236800
1237000
River of origin
Conuma
Quinsam
Rearing env.
Hatchery
Wild
Position along chromosome NA

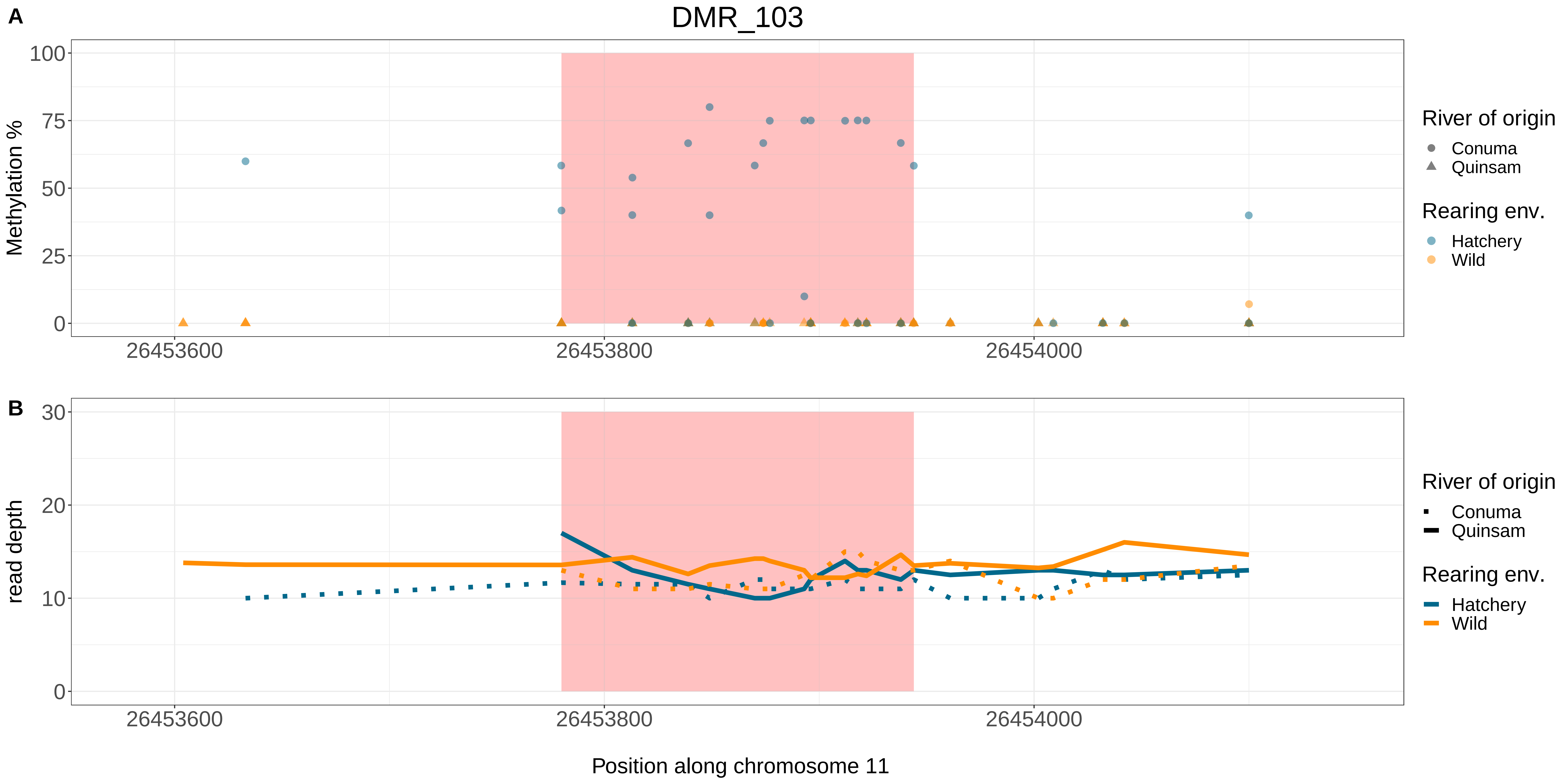
A
DMR_103
Methylation %
100
75
50
25
0
26453600
26453800
26454000
River of origin
Conuma
Quinsam
Rearing env.
Hatchery
Wild
B
read depth
30
20
10
0
26453600
26453800
26454000
River of origin
Conuma
Quinsam
Rearing env.
Hatchery
Wild
Position along chromosome 11

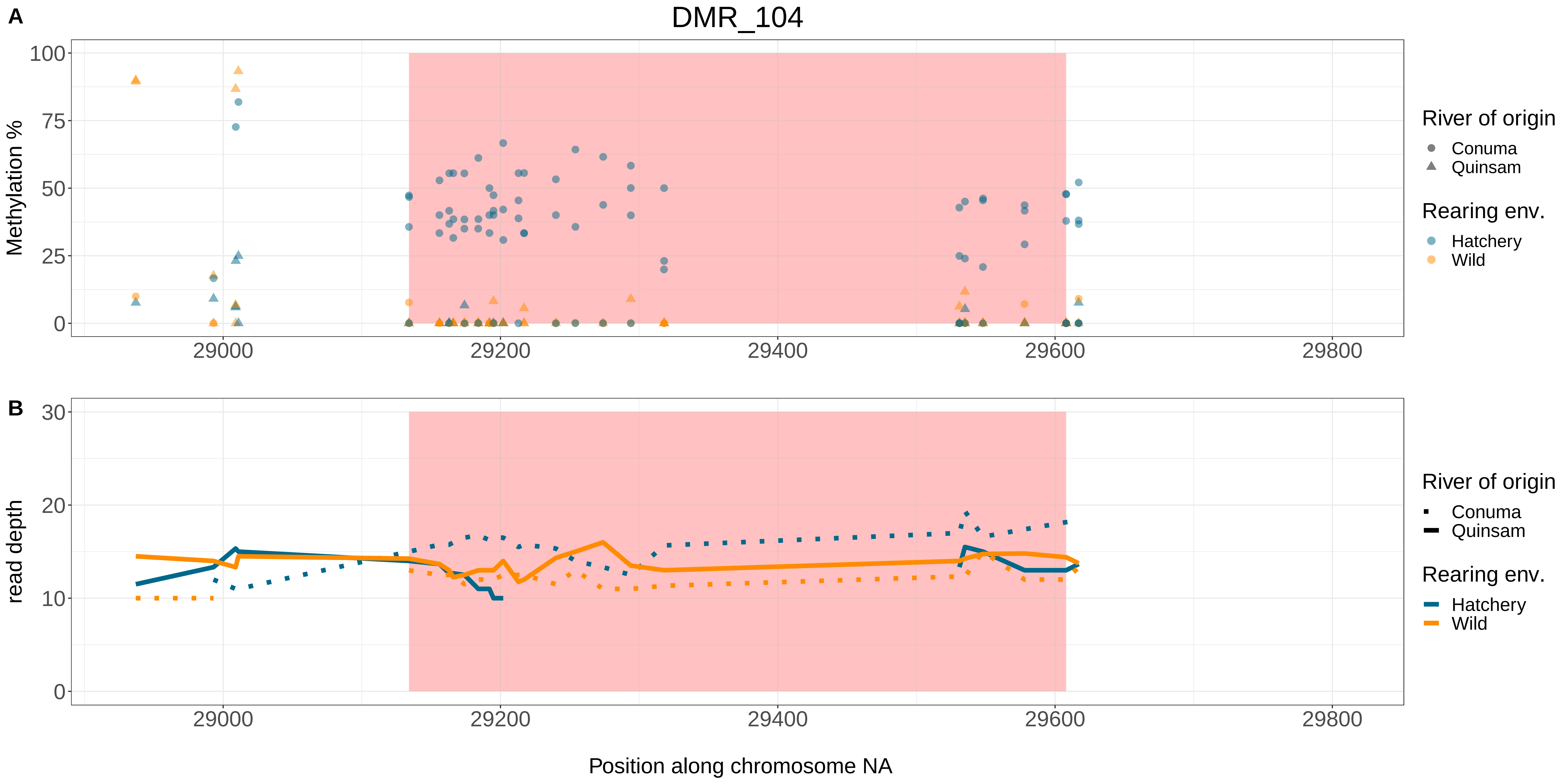

A
DMR_104
Methylation %
100
75
50
25
0
29000
29200
29400
29600
29800
River of origin
Conuma
Quinsam
Rearing env.
Hatchery
Wild
B
read depth
30
20
10
0
Position along chromosome NA

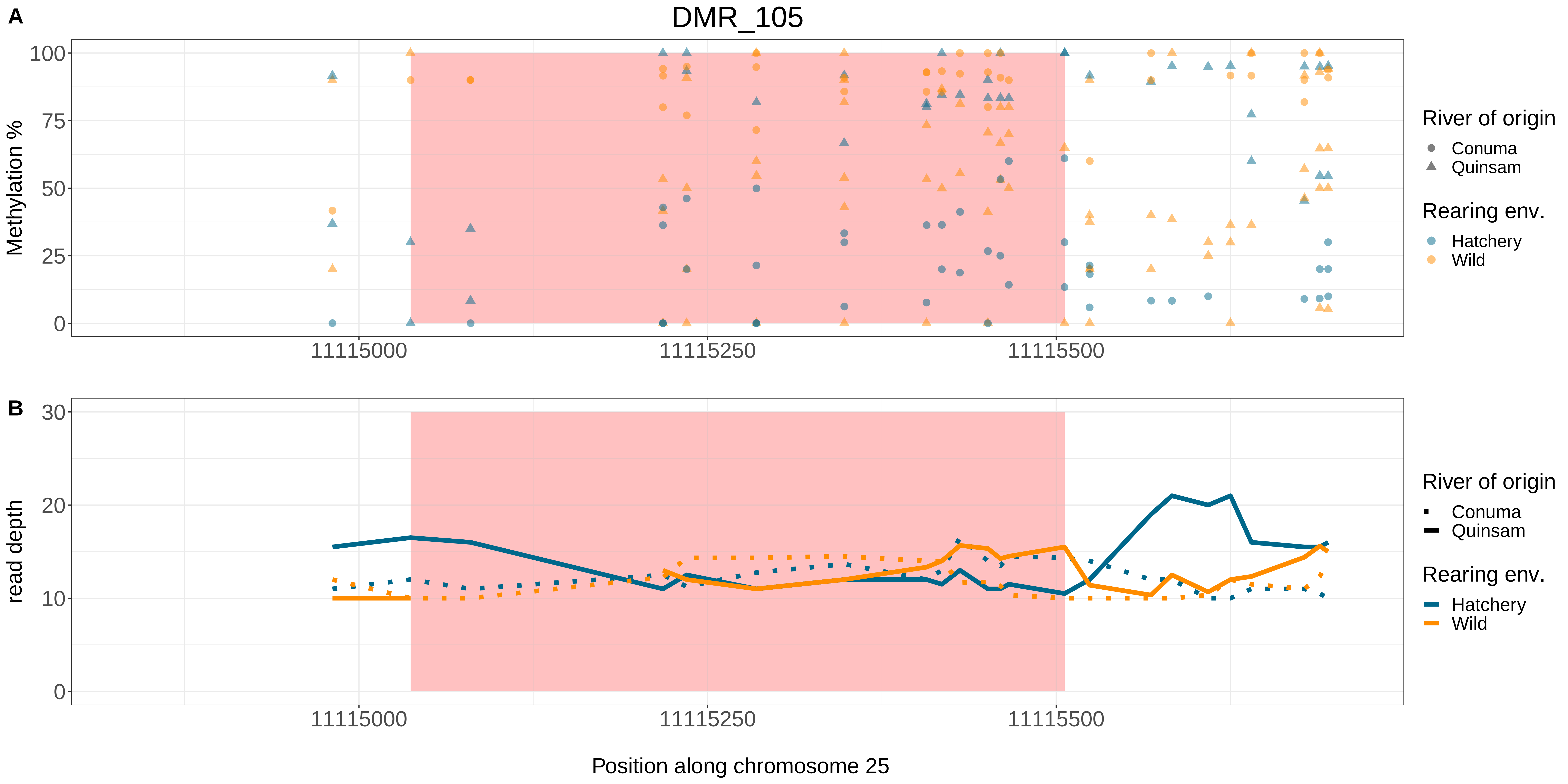
A
DMR_105
Methylation %
100
75
50
25
0
11115000
11115250
11115500
River of origin
Conuma
Quinsam
Rearing env.
Hatchery
Wild
B
read depth
30
20
10
0
11115000
11115250
11115500
River of origin
Conuma
Quinsam
Rearing env.
Hatchery
Wild
Position along chromosome 25

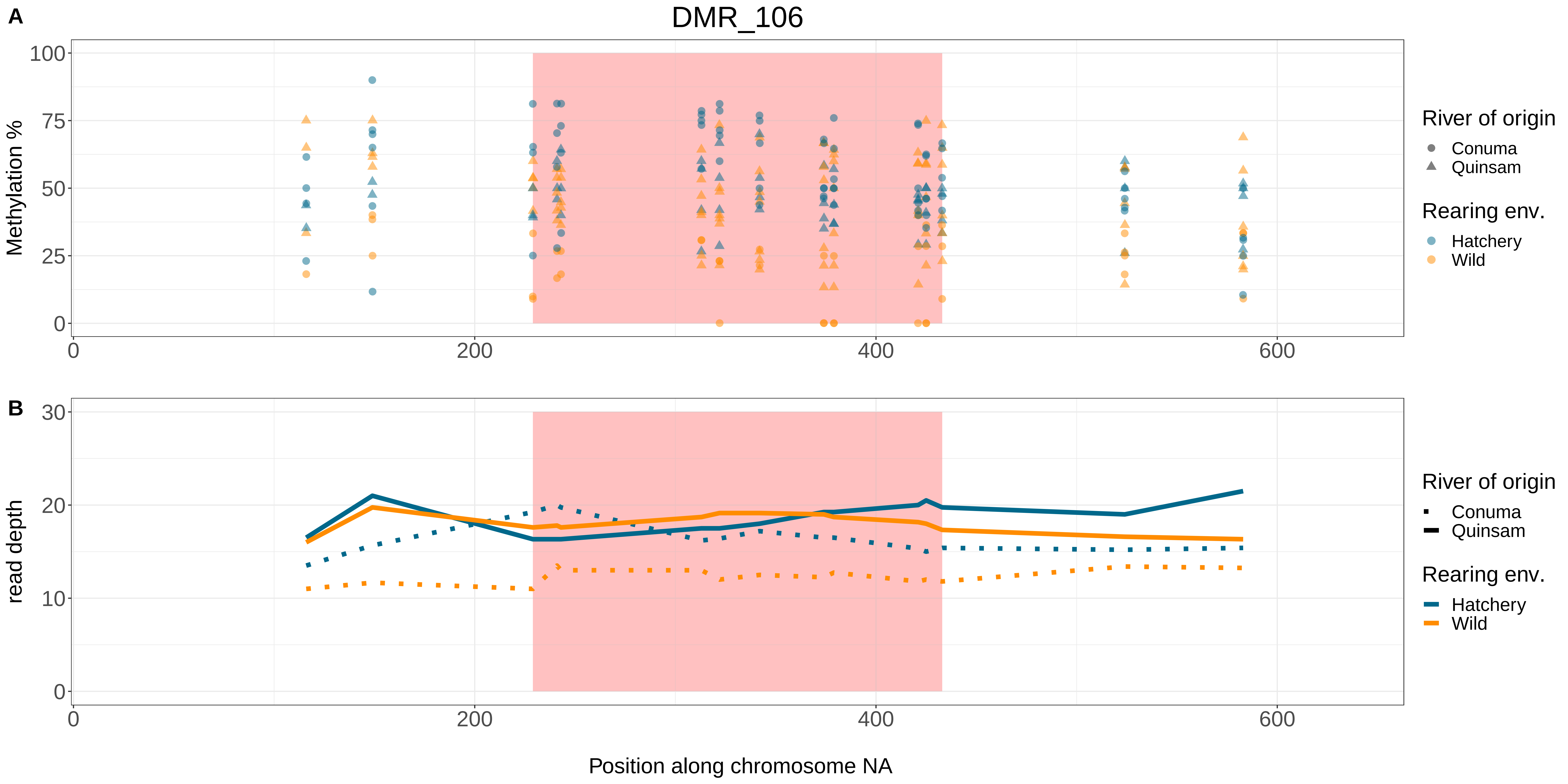

A
DMR_106
Methylation %
100
75
50
25
0
0
200
400
600
River of origin
Conuma
Quinsam
Rearing env.
Hatchery
Wild
B
read depth
30
20
10
0
0
200
400
600
River of origin
Conuma
Quinsam
Rearing env.
Hatchery
Wild
Position along chromosome NA

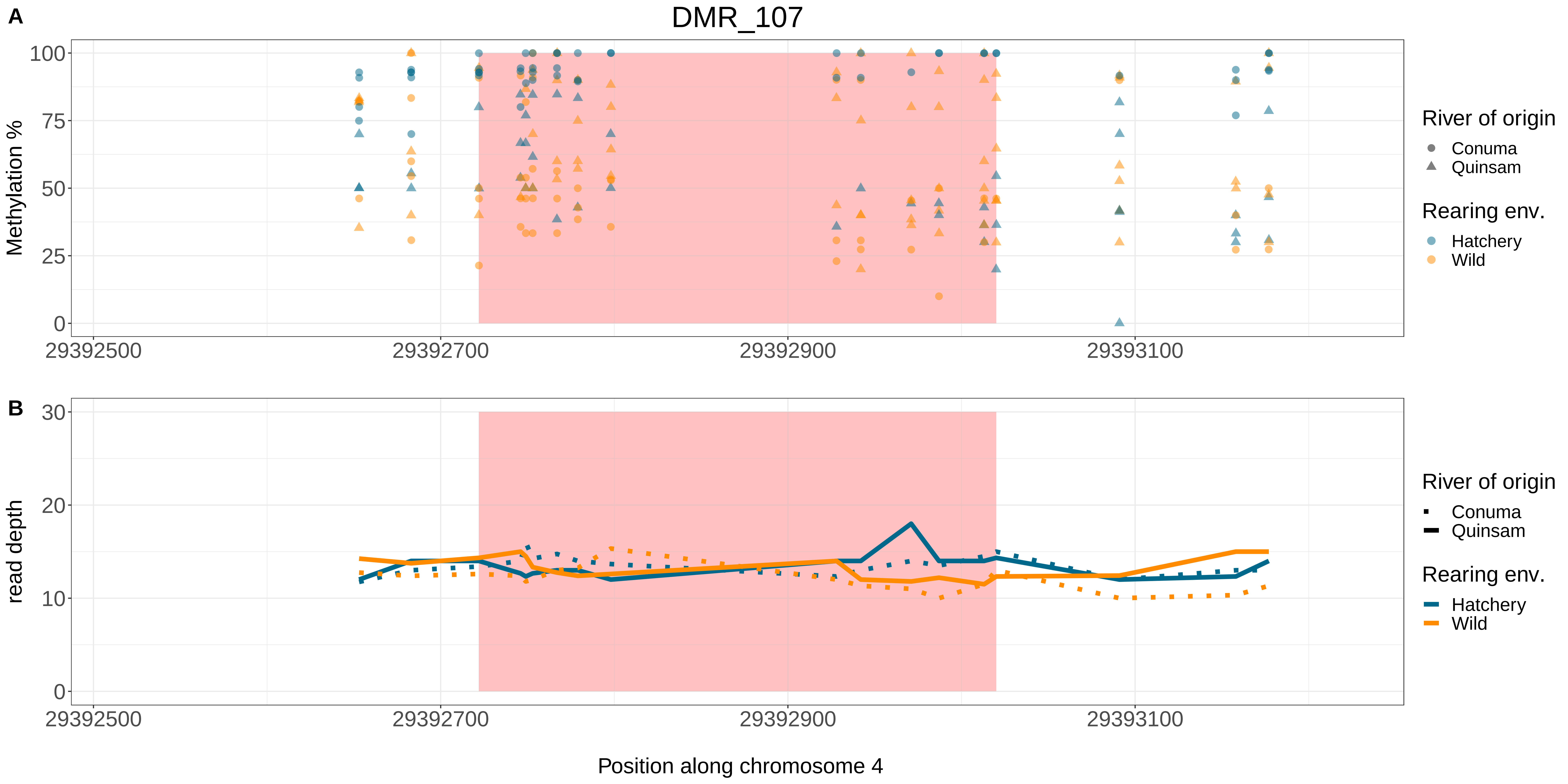
A
DMR_107
Methylation %
100
75
50
25
0
29392500
29392700
29392900
29393100
River of origin
Conuma
Quinsam
Rearing env.
Hatchery
Wild
B
read depth
30
20
10
0
29392500
29392700
29392900
29393100
River of origin
Conuma
Quinsam
Rearing env.
Hatchery
Wild
Position along chromosome 4

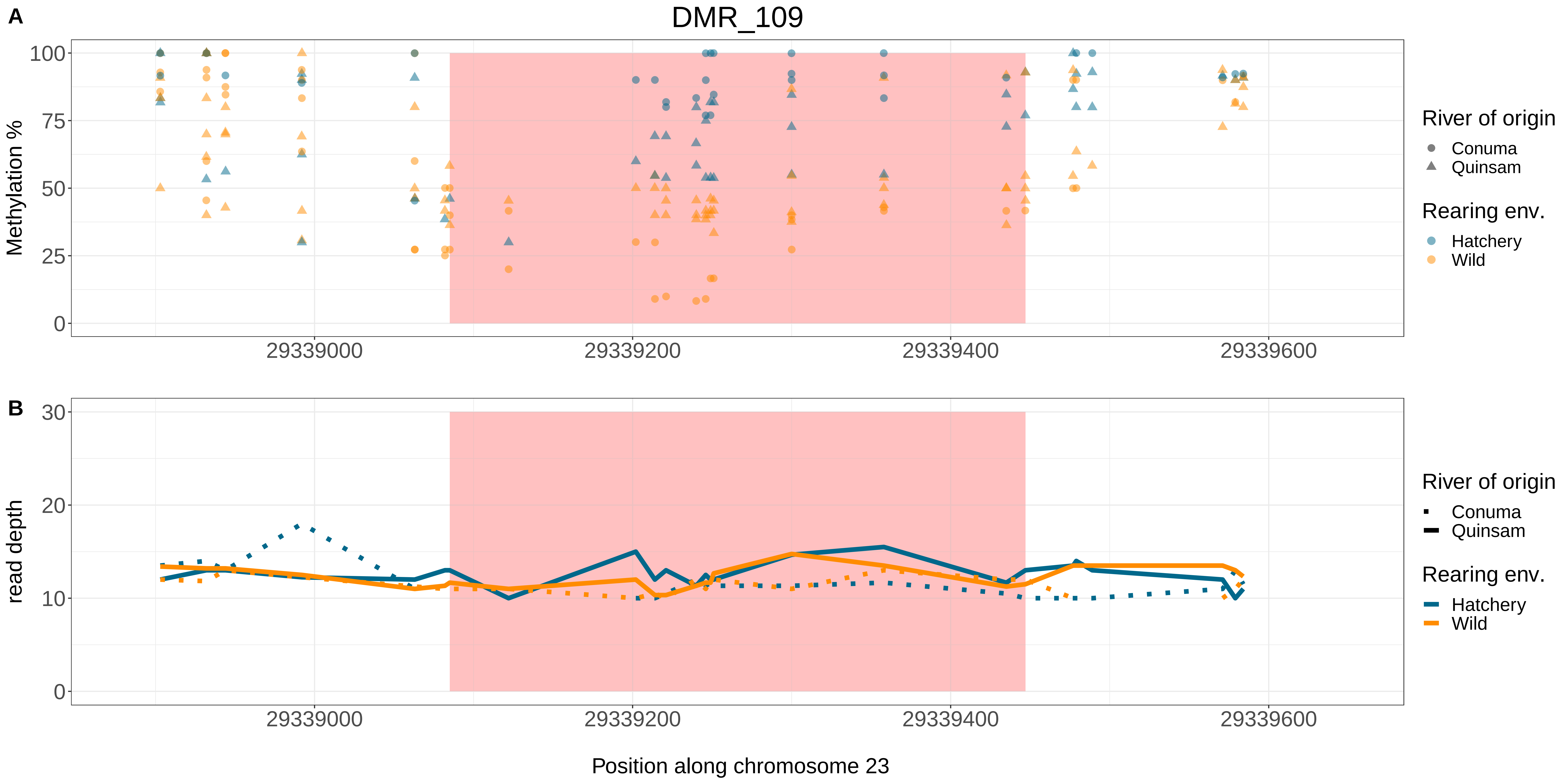

A
DMR_109
Methylation %
100
75
50
25
0
29339000
29339200
29339400
29339600
River of origin
Conuma
Quinsam
Rearing env.
Hatchery
Wild
B
read depth
30
20
10
0
29339000
29339200
29339400
29339600
River of origin
Conuma
Quinsam
Rearing env.
Hatchery
Wild
Position along chromosome 23

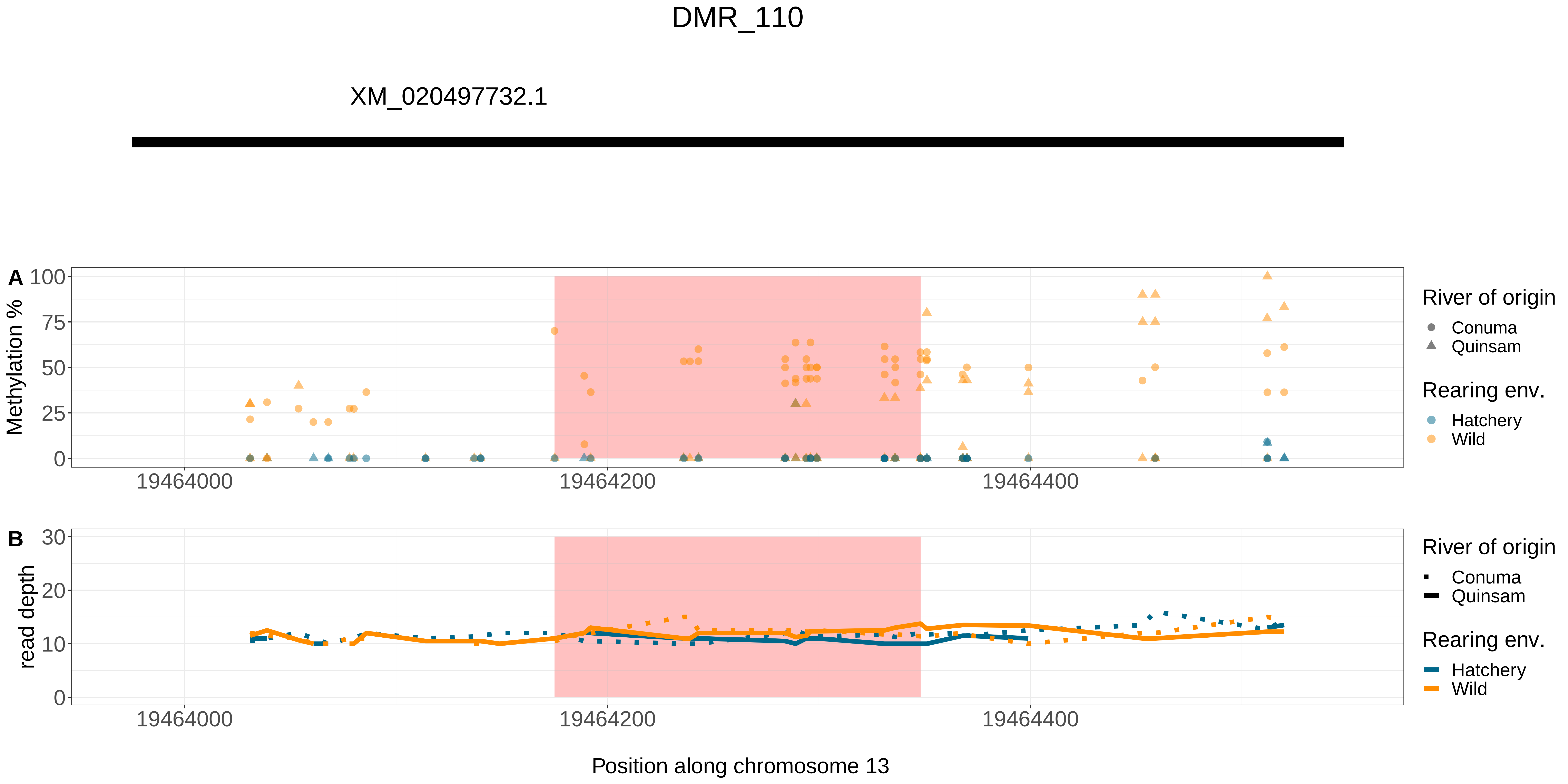
DMR_110
XM_020497732.1
A
B
Methylation %
read depth
100
75
50
25
0
30
20
10
0
19464000
19464200
19464400
River of origin
Conuma
Quinsam
Rearing env.
Hatchery
Wild
Position along chromosome 13

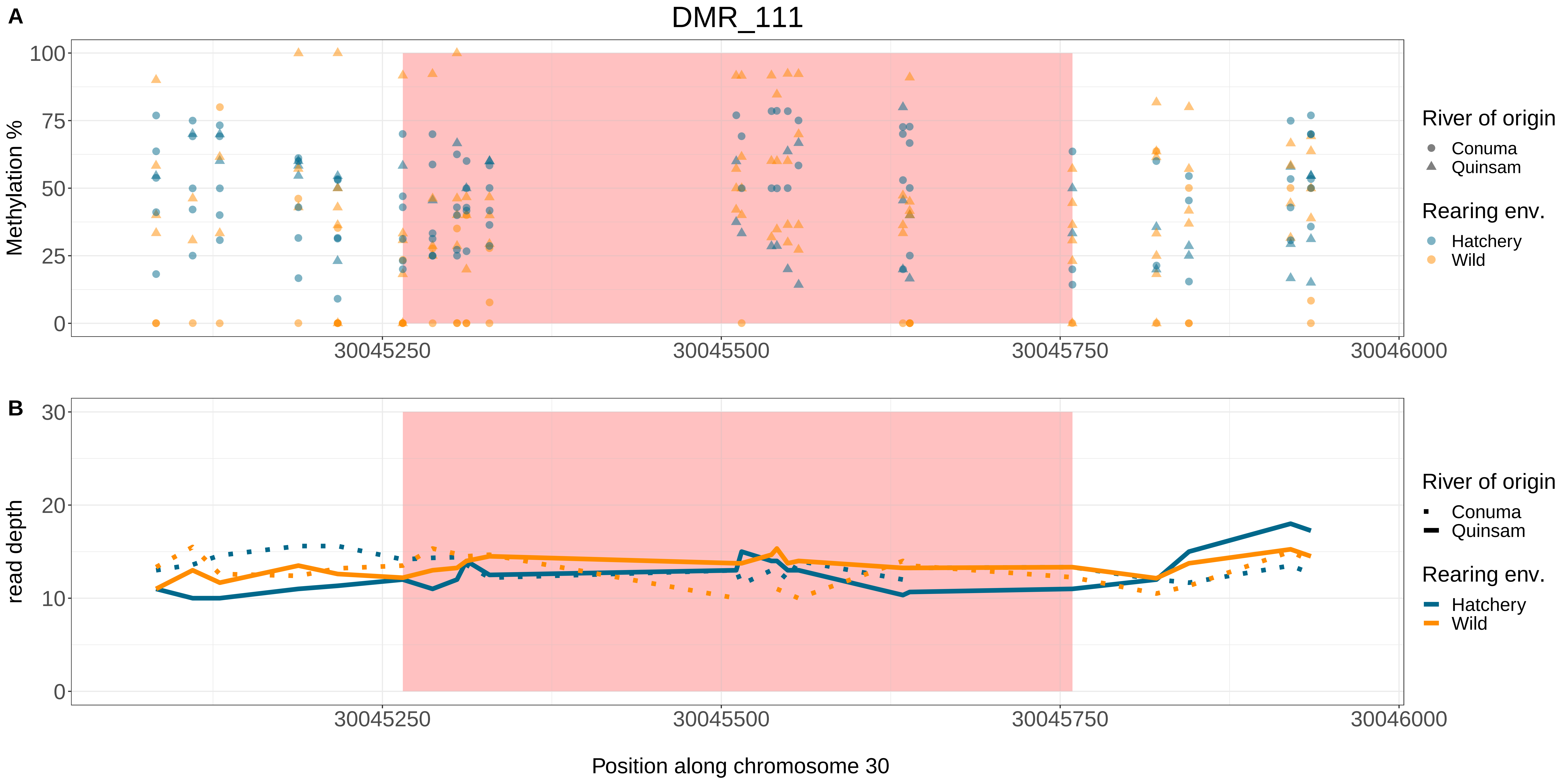

A
DMR_111
Methylation %
100
75
50
25
0
30045250
30045500
30045750
30046000
River of origin
Conuma
Quinsam
Rearing env.
Hatchery
Wild
B
read depth
30
20
10
0
Position along chromosome 30

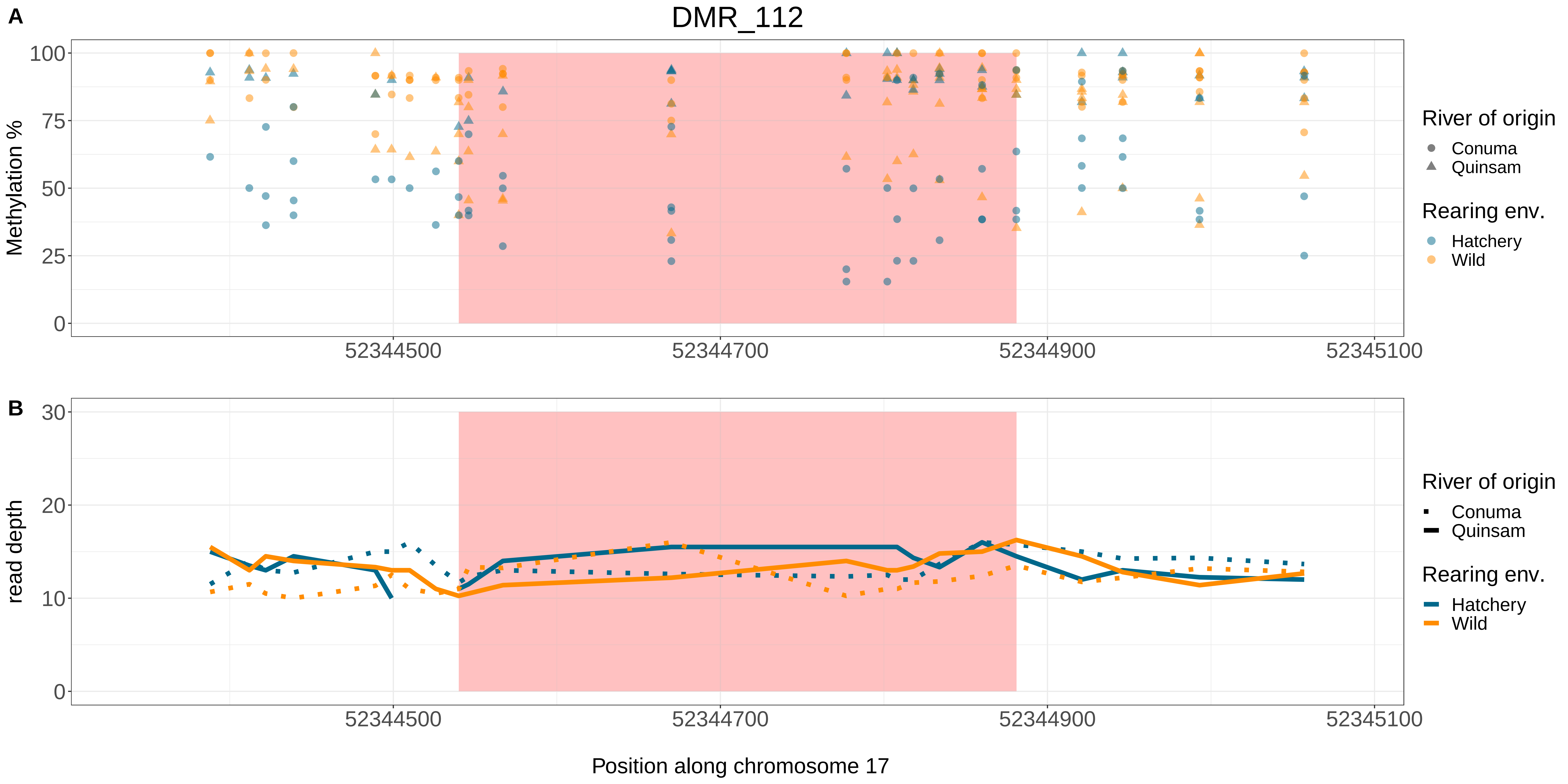
DMR_112
A
Methylation %
100
75
50
25
0
52344500
52344700
52344900
52345100
River of origin
Conuma
Quinsam
Rearing env.
Hatchery
Wild
B
read depth
30
20
10
0
52344500
52344700
52344900
52345100
River of origin
Conuma
Quinsam
Rearing env.
Hatchery
Wild
Position along chromosome 17

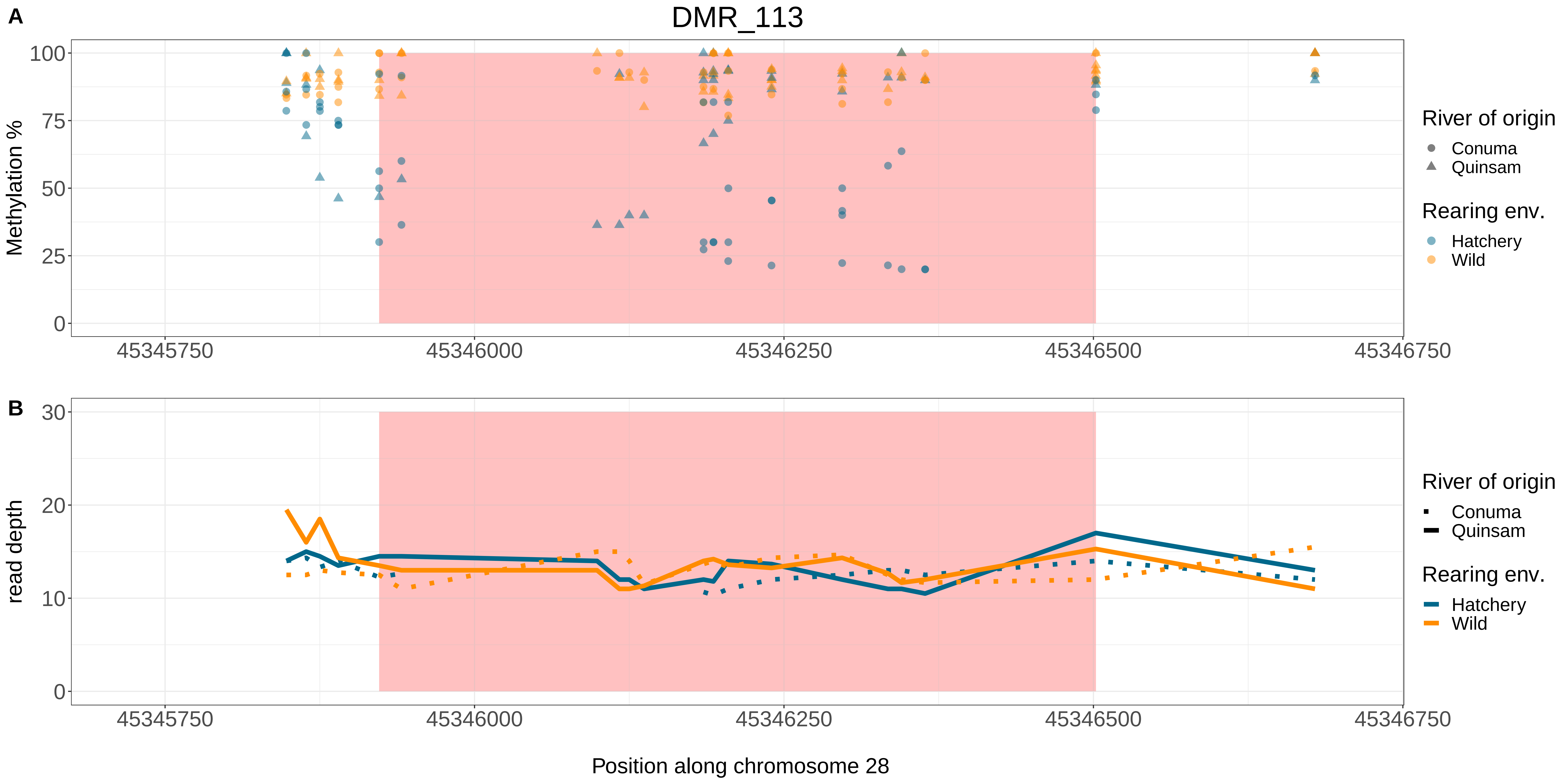

A
DMR_113
Methylation %
100
75
50
25
0
45345750
45346000
45346250
45346500
45346750
River of origin
Conuma
Quinsam
Rearing env.
Hatchery
Wild
B
read depth
30
20
10
0
45345750
45346000
45346250
45346500
45346750
River of origin
Conuma
Quinsam
Rearing env.
Hatchery
Wild
Position along chromosome 28

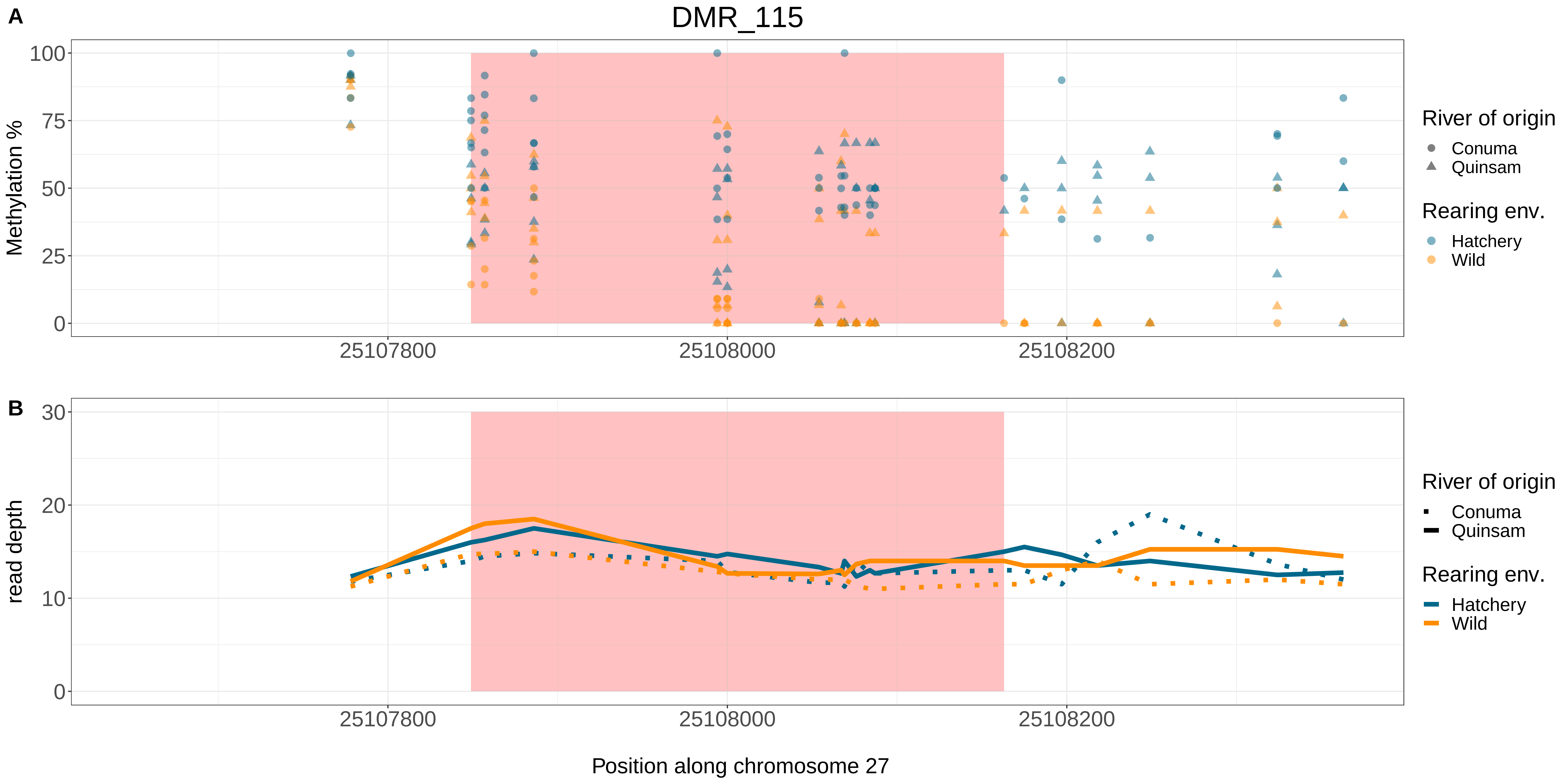
A
DMR_115
Methylation %
100
75
50
25
0
25107800
25108000
25108200
River of origin
Conuma
Quinsam
Rearing env.
Hatchery
Wild
B
read depth
30
20
10
0
25107800
25108000
25108200
River of origin
Conuma
Quinsam
Rearing env.
Hatchery
Wild
Position along chromosome 27

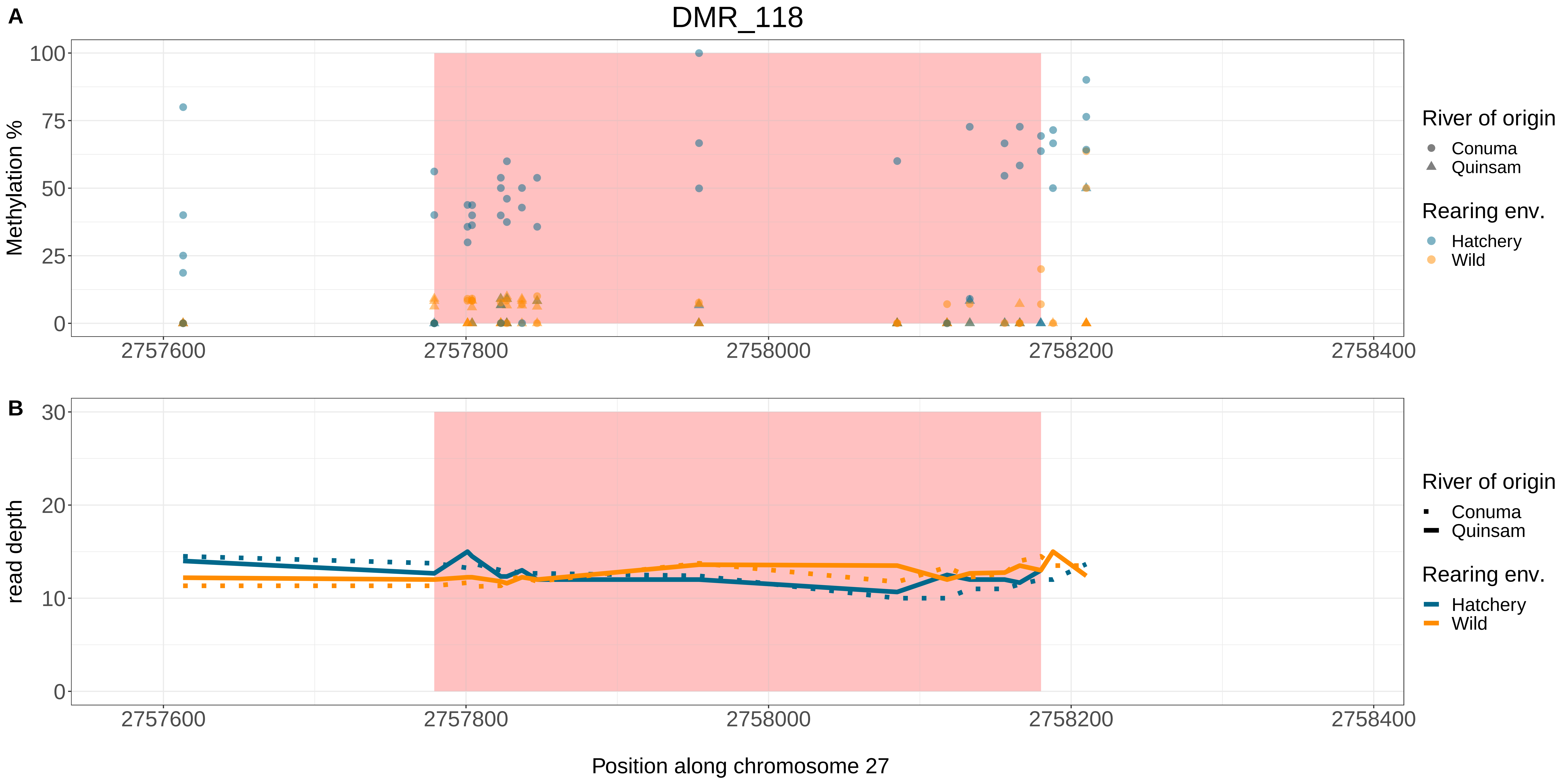
A
DMR_118
Methylation %
100
75
50
25
0
2757600
2757800
2758000
2758200
2758400
River of origin
Conuma
Quinsam
Rearing env.
Hatchery
Wild
B
read depth
30
20
10
0
2757600
2757800
2758000
2758200
2758400
River of origin
Conuma
Quinsam
Rearing env.
Hatchery
Wild
Position along chromosome 27

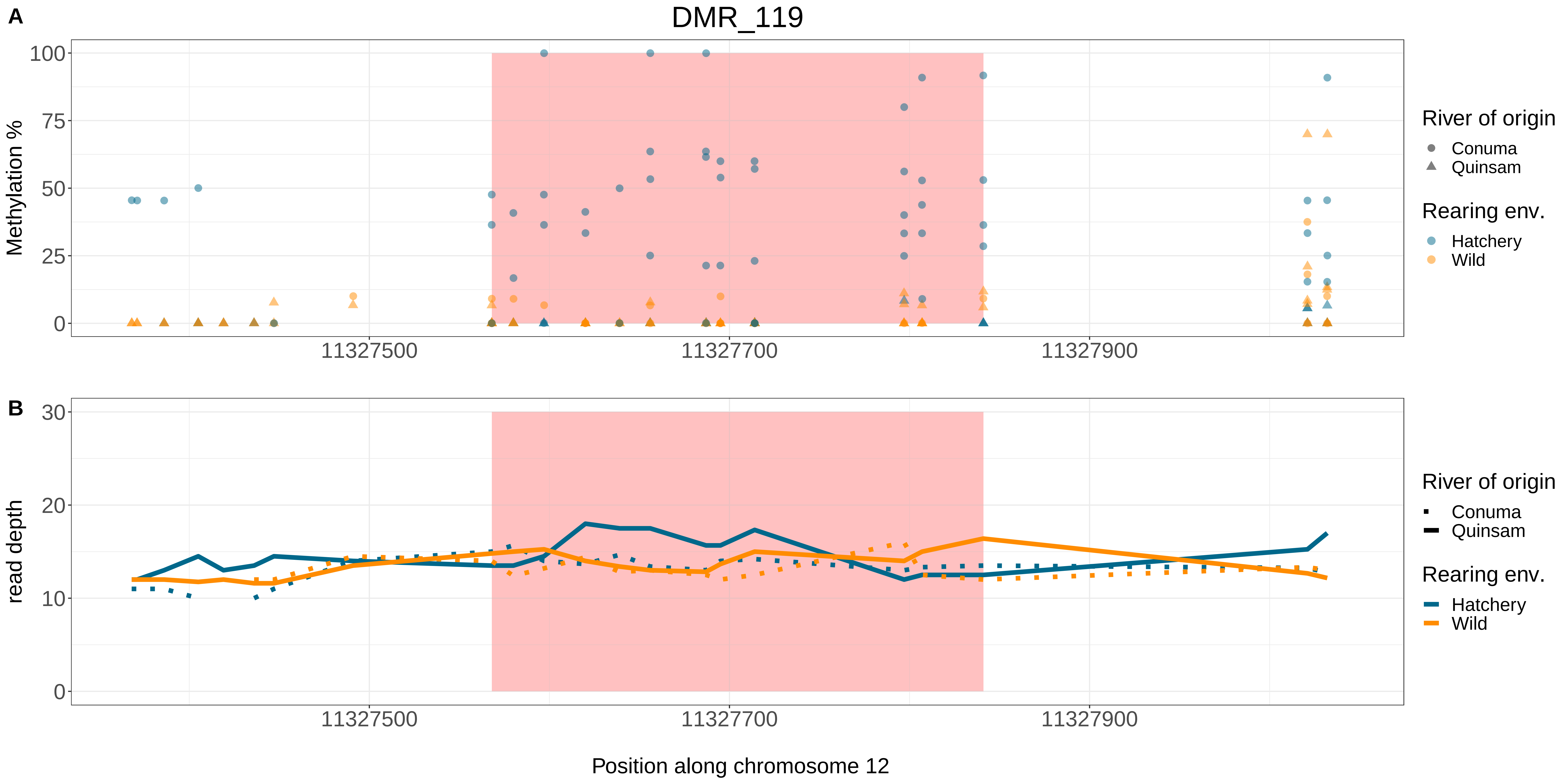

A
DMR_119
Methylation %
100
75
50
25
0
11327500
11327700
11327900
River of origin
Conuma
Quinsam
Rearing env.
Hatchery
Wild
B
read depth
30
20
10
0
11327500
11327700
11327900
River of origin
Conuma
Quinsam
Rearing env.
Hatchery
Wild
Position along chromosome 12

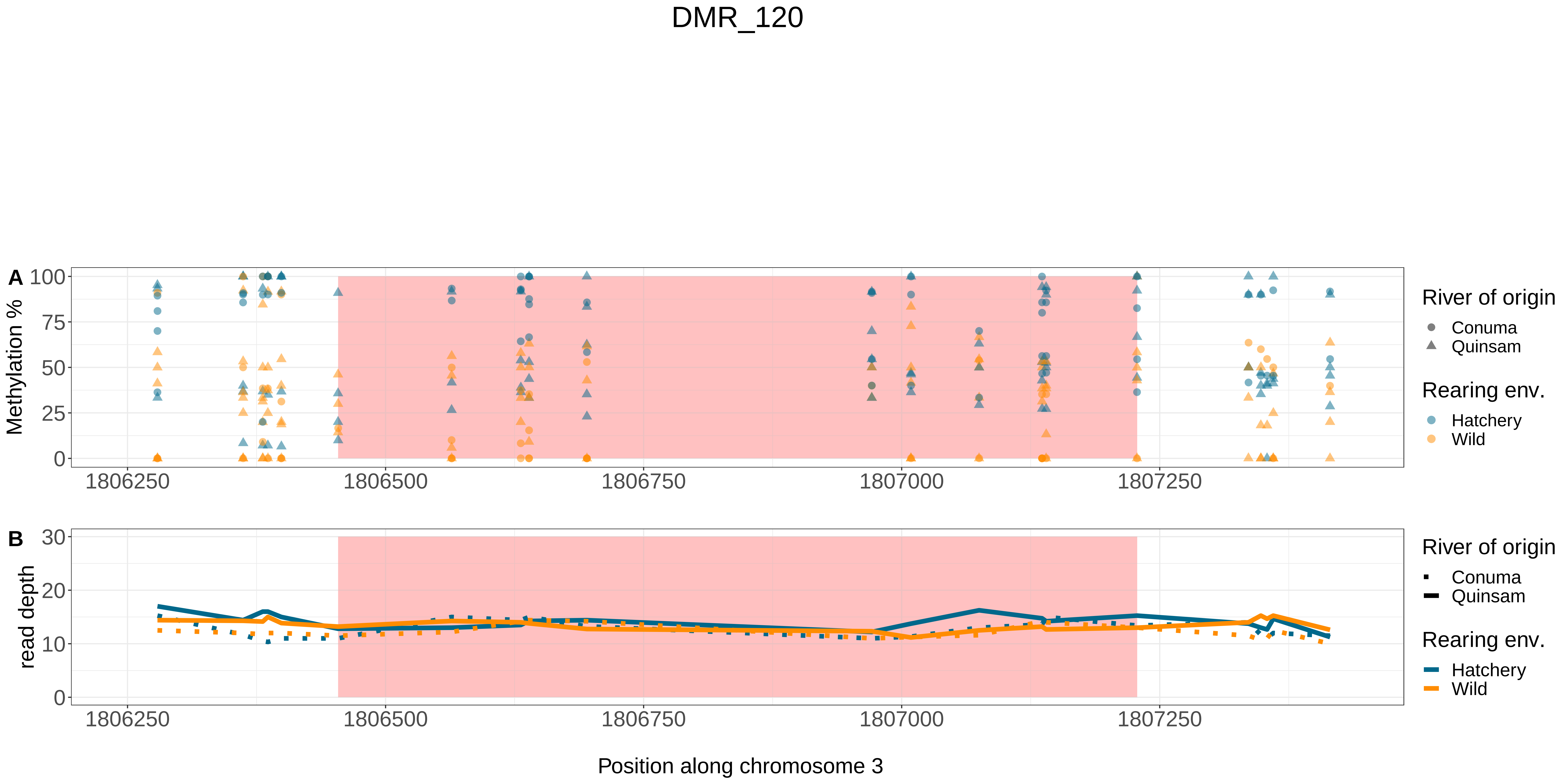
DMR_120
A
Methylation %
100
75
50
25
0
1806250
1806500
1806750
1807000
1807250
River of origin
Conuma
Quinsam
Rearing env.
Hatchery
Wild
B
read depth
30
20
10
0
1806250
1806500
1806750
1807000
1807250
River of origin
Conuma
Quinsam
Rearing env.
Hatchery
Wild
Position along chromosome 3

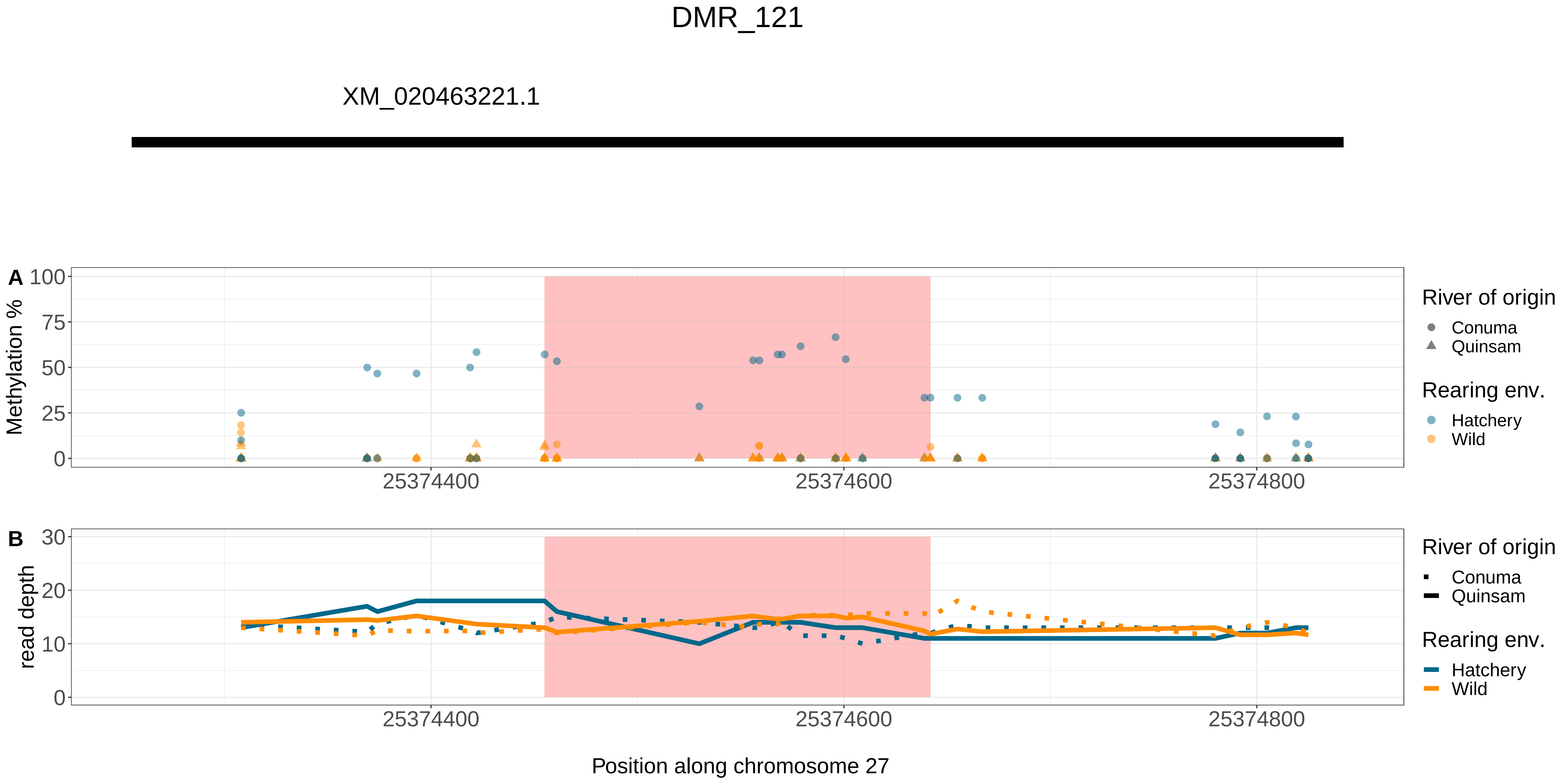
DMR_121
XM_020463221.1
A
Methylation %
100
75
50
25
0
25374400
25374600
25374800
River of origin
Conuma
Quinsam
Rearing env.
Hatchery
Wild
B
read depth
30
20
10
0
25374400
25374600
25374800
River of origin
Conuma
Quinsam
Rearing env.
Hatchery
Wild
Position along chromosome 27

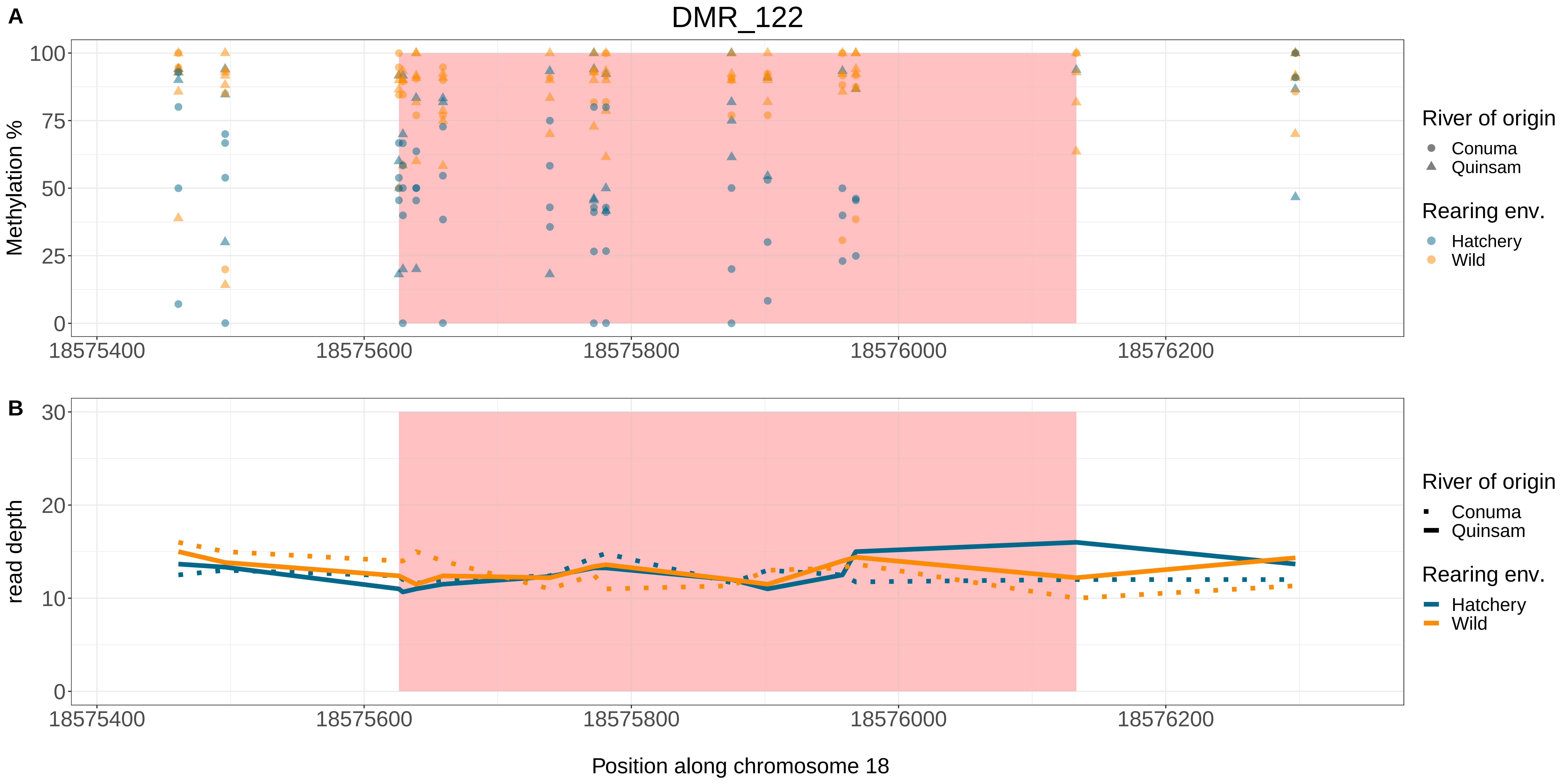

A
DMR_122
Methylation %
100
75
50
25
0
18575400
18575600
18575800
18576000
18576200
River of origin
Conuma
Quinsam
Rearing env.
Hatchery
Wild
B
read depth
30
20
10
0
18575400
18575600
18575800
18576000
18576200
River of origin
Conuma
Quinsam
Rearing env.
Hatchery
Wild
Position along chromosome 18

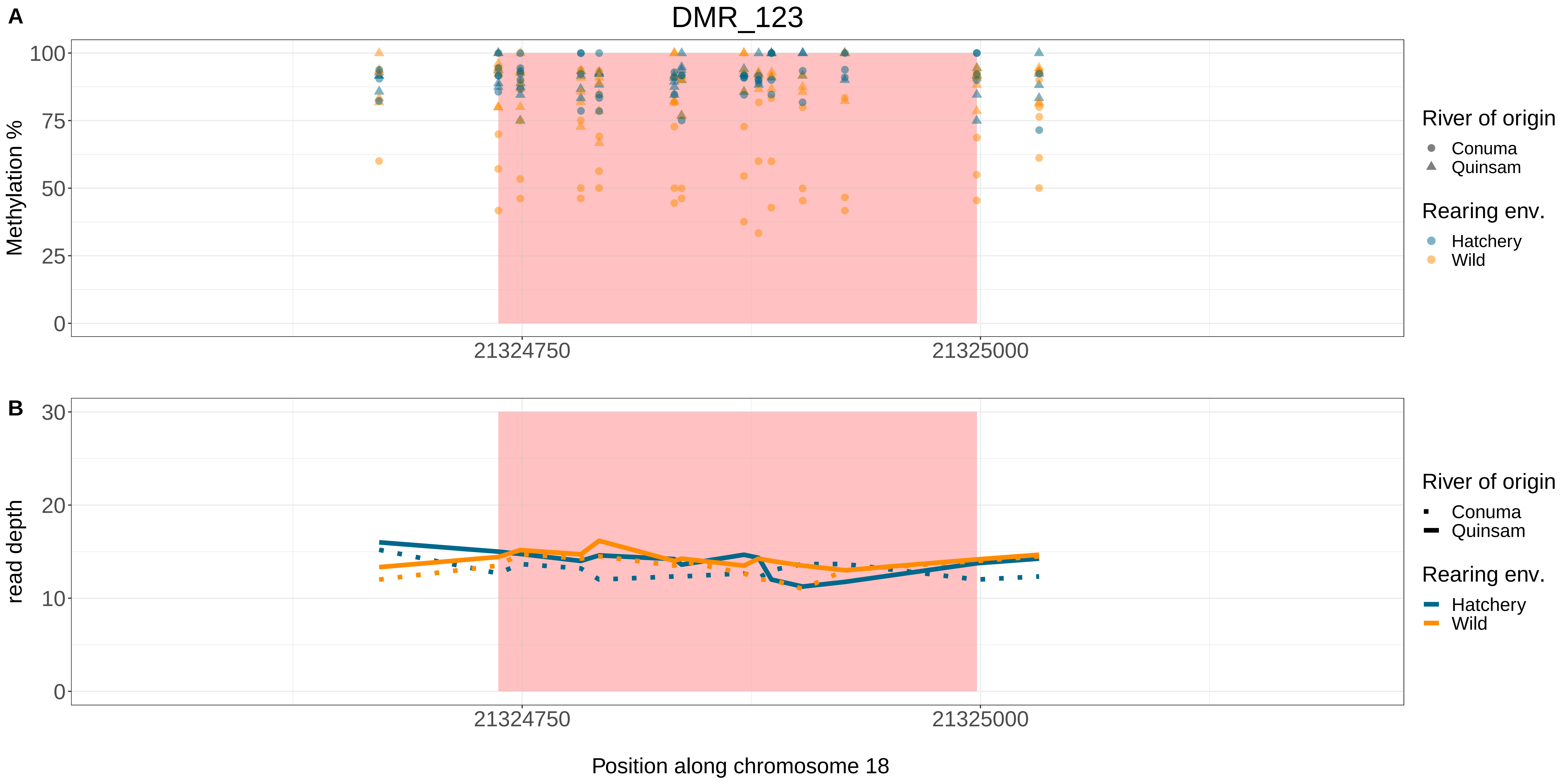
A
DMR_123
Methylation %
100
75
50
25
0
21324750
21325000
River of origin
Conuma
Quinsam
Rearing env.
Hatchery
Wild
B
read depth
30
20
10
0
21324750
21325000
River of origin
Conuma
Quinsam
Rearing env.
Hatchery
Wild
Position along chromosome 18

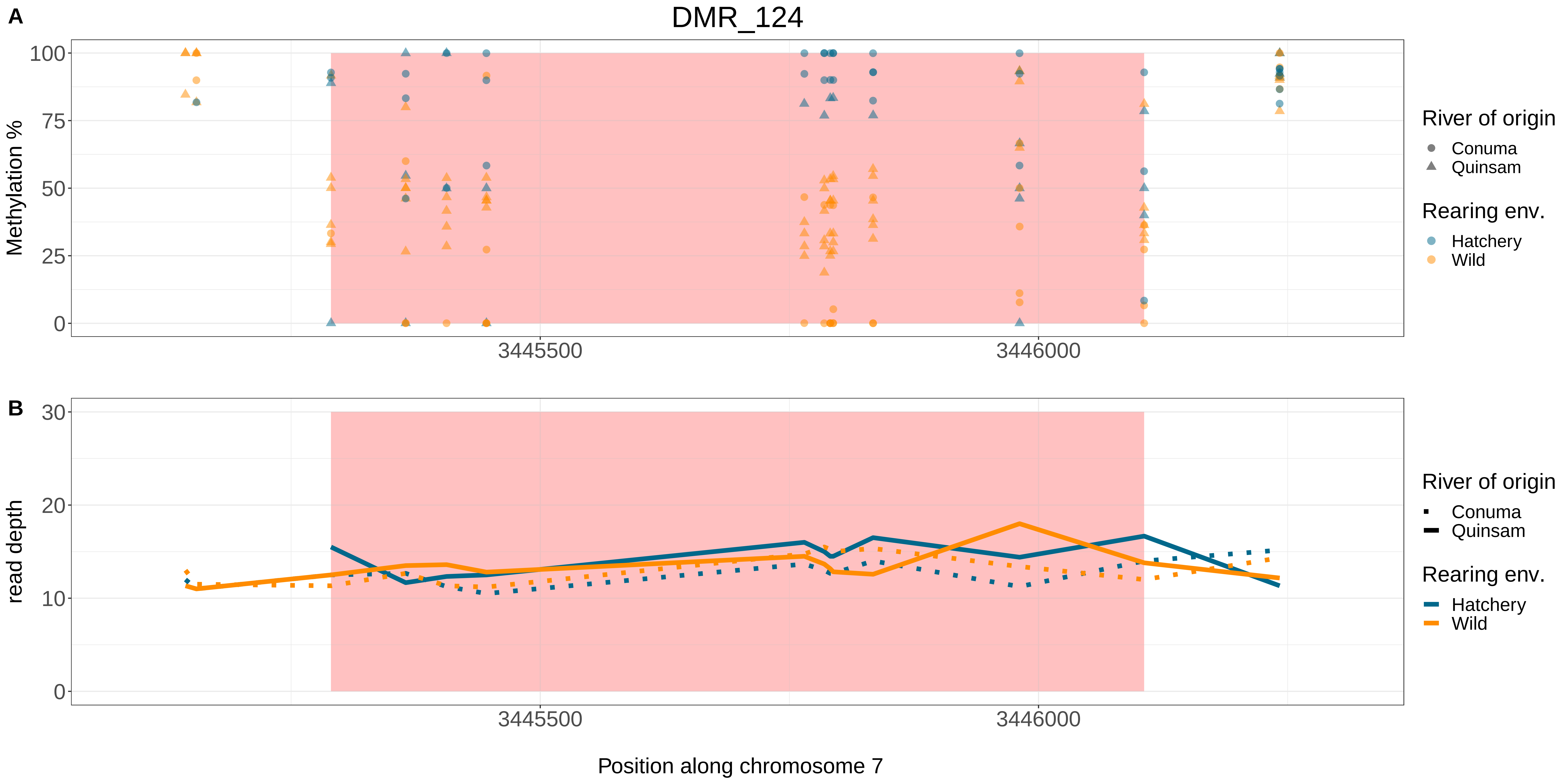

A
DMR_124
Methylation %
100
75
50
25
0
3445500
3446000
River of origin
Conuma
Quinsam
Rearing env.
Hatchery
Wild
B
read depth
30
20
10
0
3445500
3446000
River of origin
Conuma
Quinsam
Rearing env.
Hatchery
Wild
Position along chromosome 7

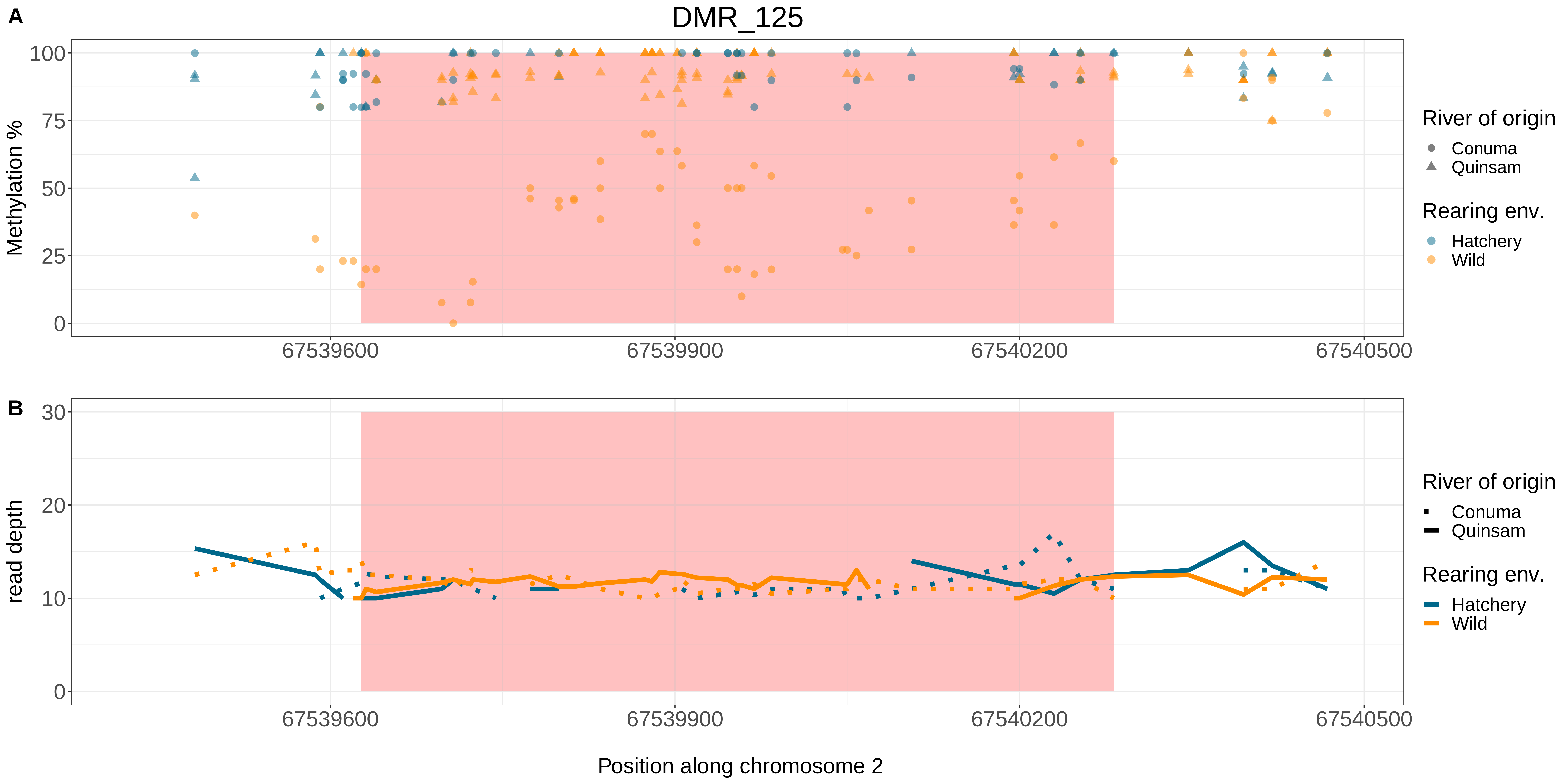

A
DMR_125
Methylation %
100
75
50
25
0
67539600
67539900
67540200
67540500
River of origin
Conuma
Quinsam
Rearing env.
Hatchery
Wild
B
read depth
30
20
10
0
67539600
67539900
67540200
67540500
River of origin
Conuma
Quinsam
Rearing env.
Hatchery
Wild
Position along chromosome 2

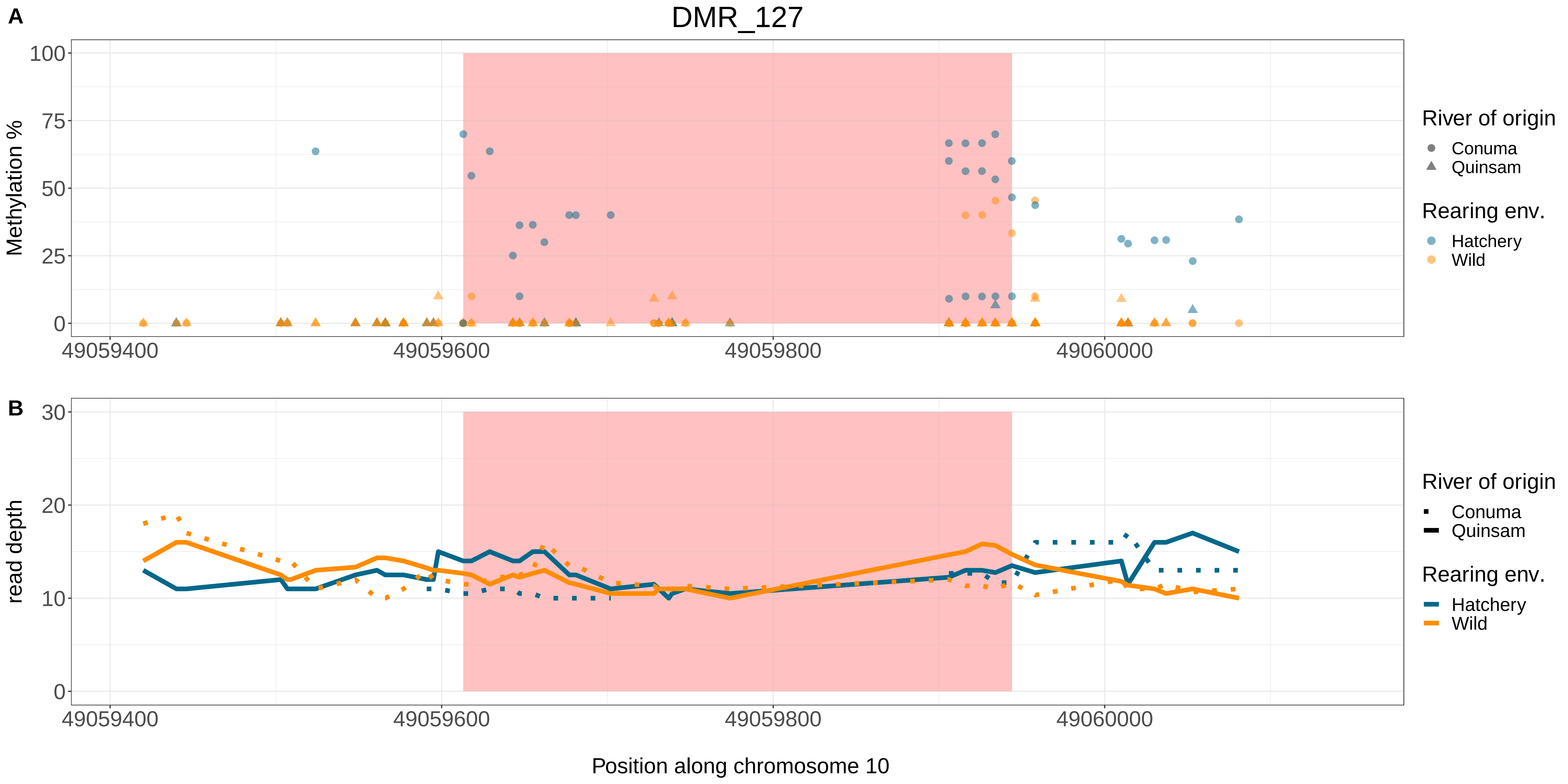
A
DMR_127
Methylation %
100
75
50
25
0
49059400
49059600
49059800
49060000
River of origin
Conuma
Quinsam
Rearing env.
Hatchery
Wild
B
read depth
30
20
10
0
49059400
49059600
49059800
49060000
River of origin
Conuma
Quinsam
Rearing env.
Hatchery
Wild
Position along chromosome 10

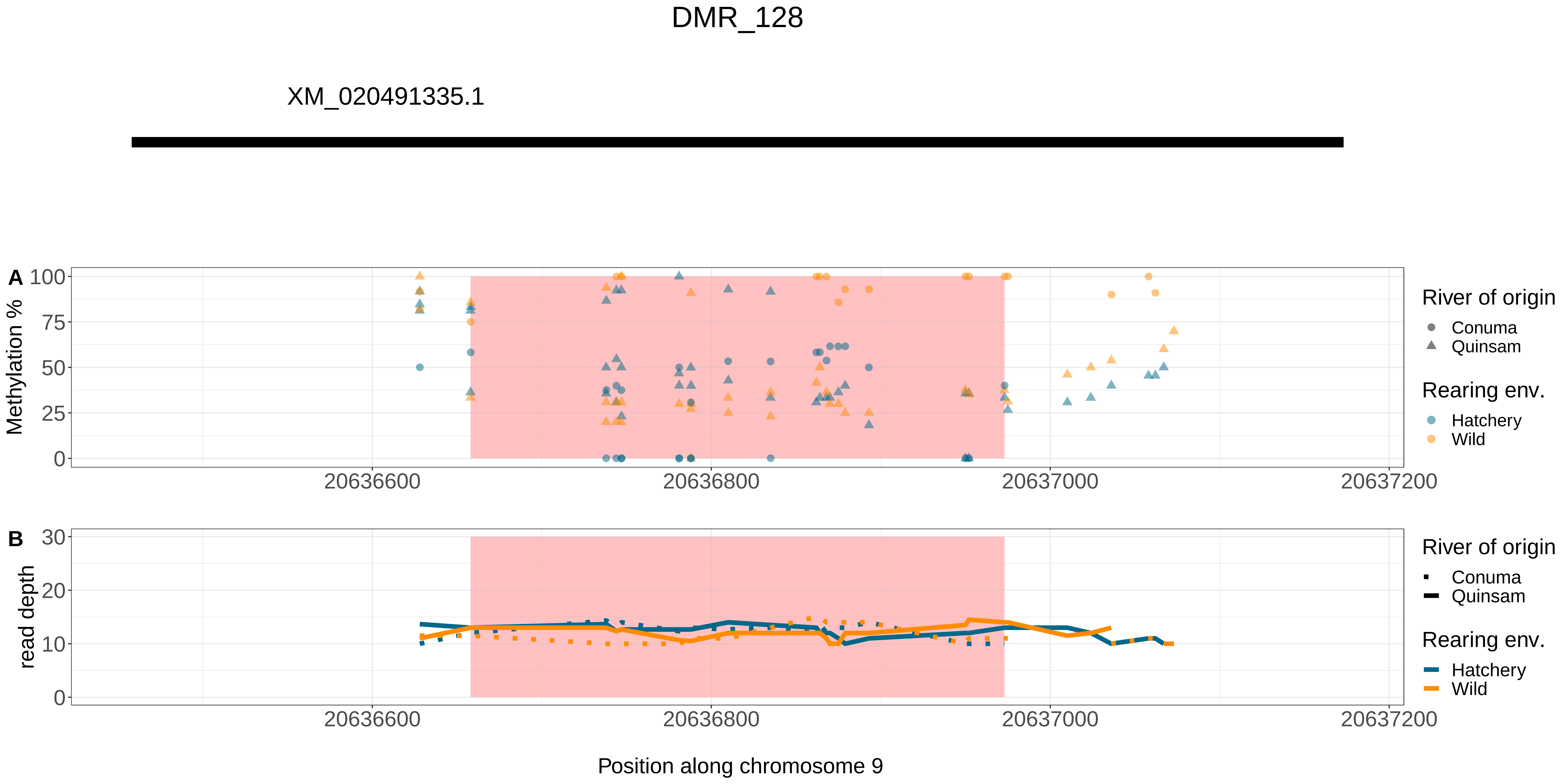
DMR_128
XM_020491335.1
A
Methylation %
100
75
50
25
0
20636600
20636800
20637000
20637200
River of origin
Conuma
Quinsam
Rearing env.
Hatchery
Wild
B
read depth
30
20
10
0
20636600
20636800
20637000
20637200
River of origin
Conuma
Quinsam
Rearing env.
Hatchery
Wild
Position along chromosome 9

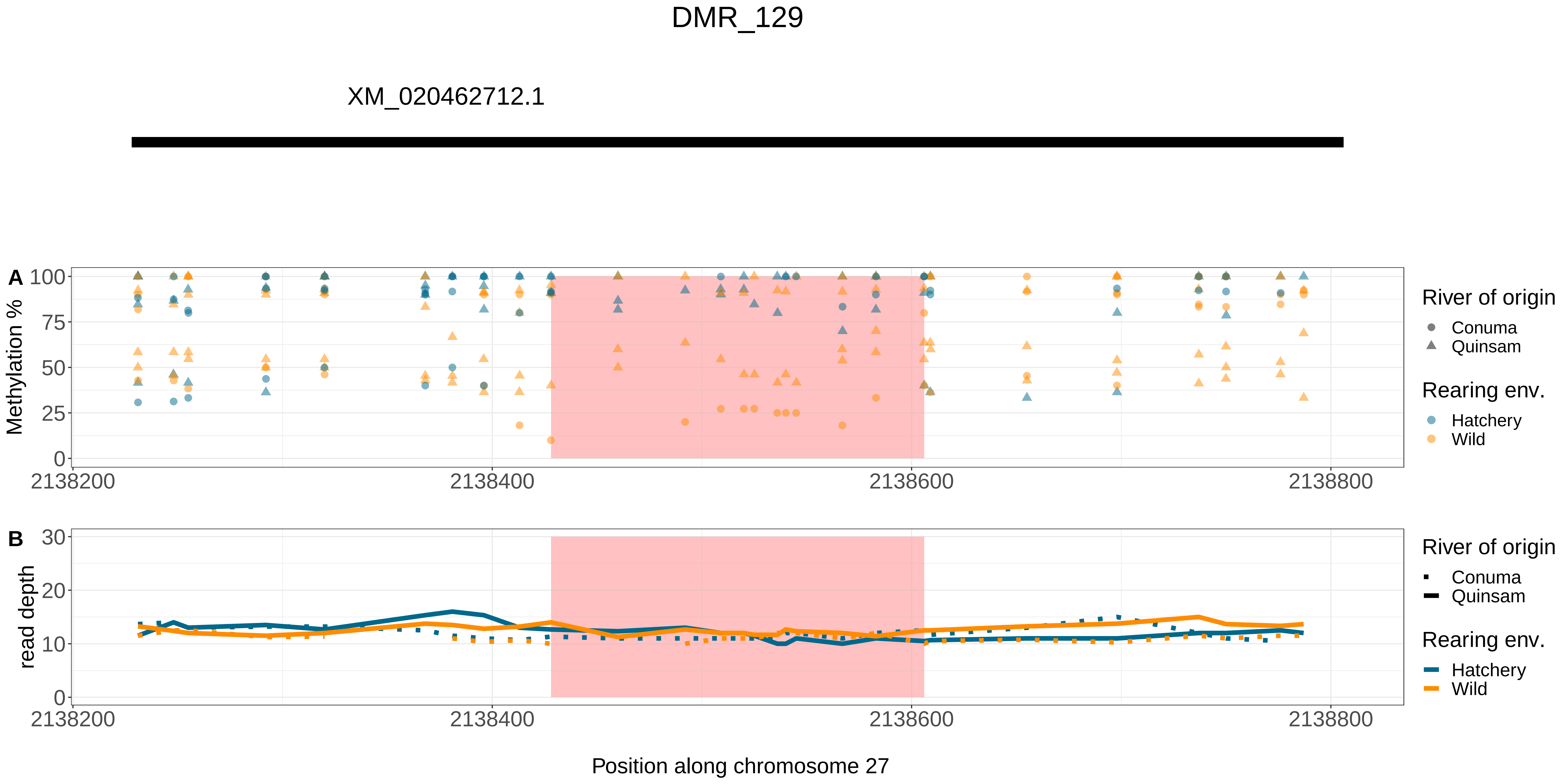
DMR_129
XM_020462712.1
A
Methylation %
100
75
50
25
0
2138200
2138400
2138600
2138800
River of origin
Conuma
Quinsam
Rearing env.
Hatchery
Wild
B
read depth
30
20
10
0
2138200
2138400
2138600
2138800
River of origin
Conuma
Quinsam
Rearing env.
Hatchery
Wild
Position along chromosome 27

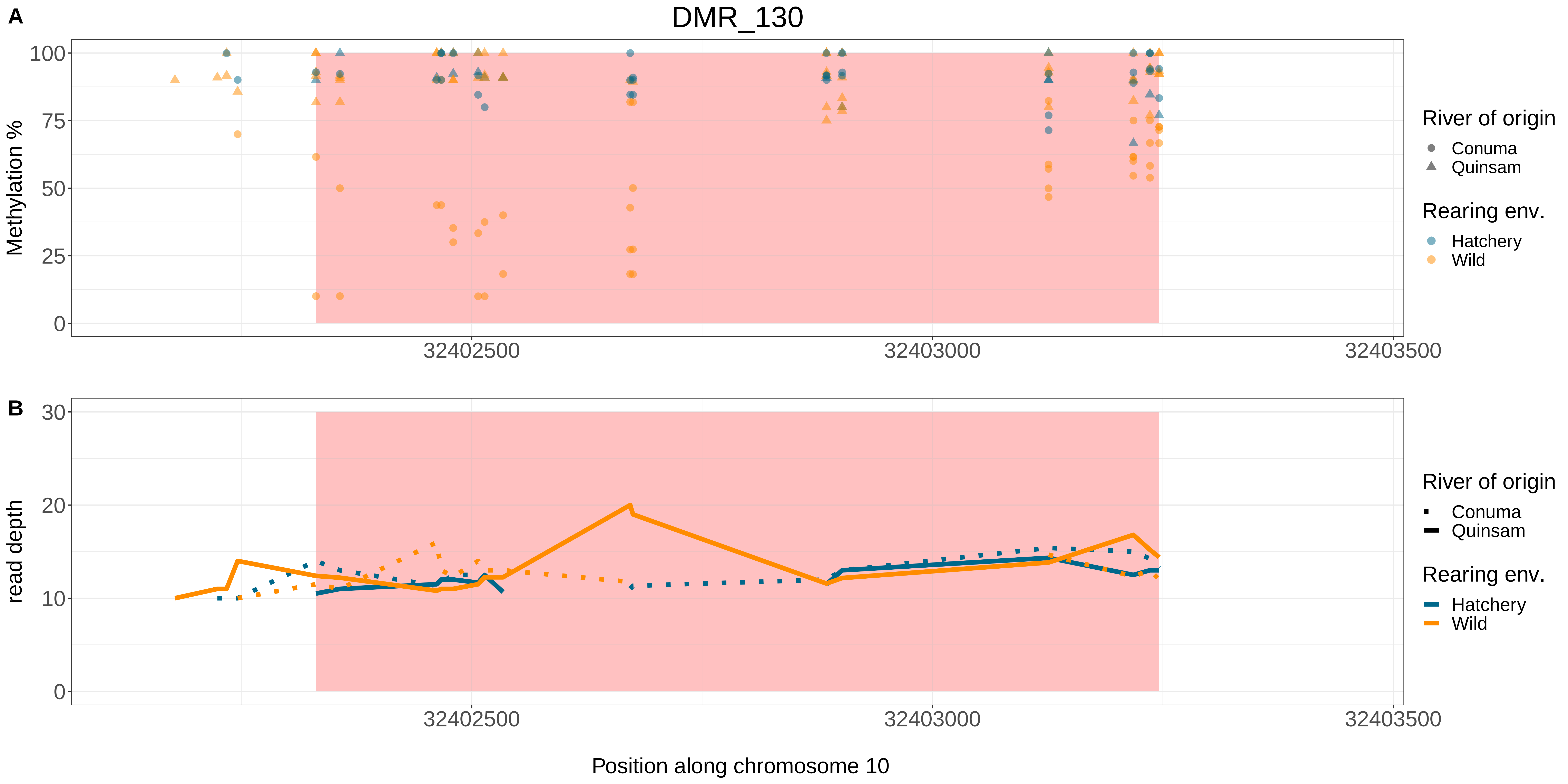
A
DMR_130
Methylation %
100
75
50
25
0
32402500
32403000
32403500
River of origin
Conuma
Quinsam
Rearing env.
Hatchery
Wild
B
read depth
30
20
10
0
32402500
32403000
32403500
River of origin
Conuma
Quinsam
Rearing env.
Hatchery
Wild
Position along chromosome 10

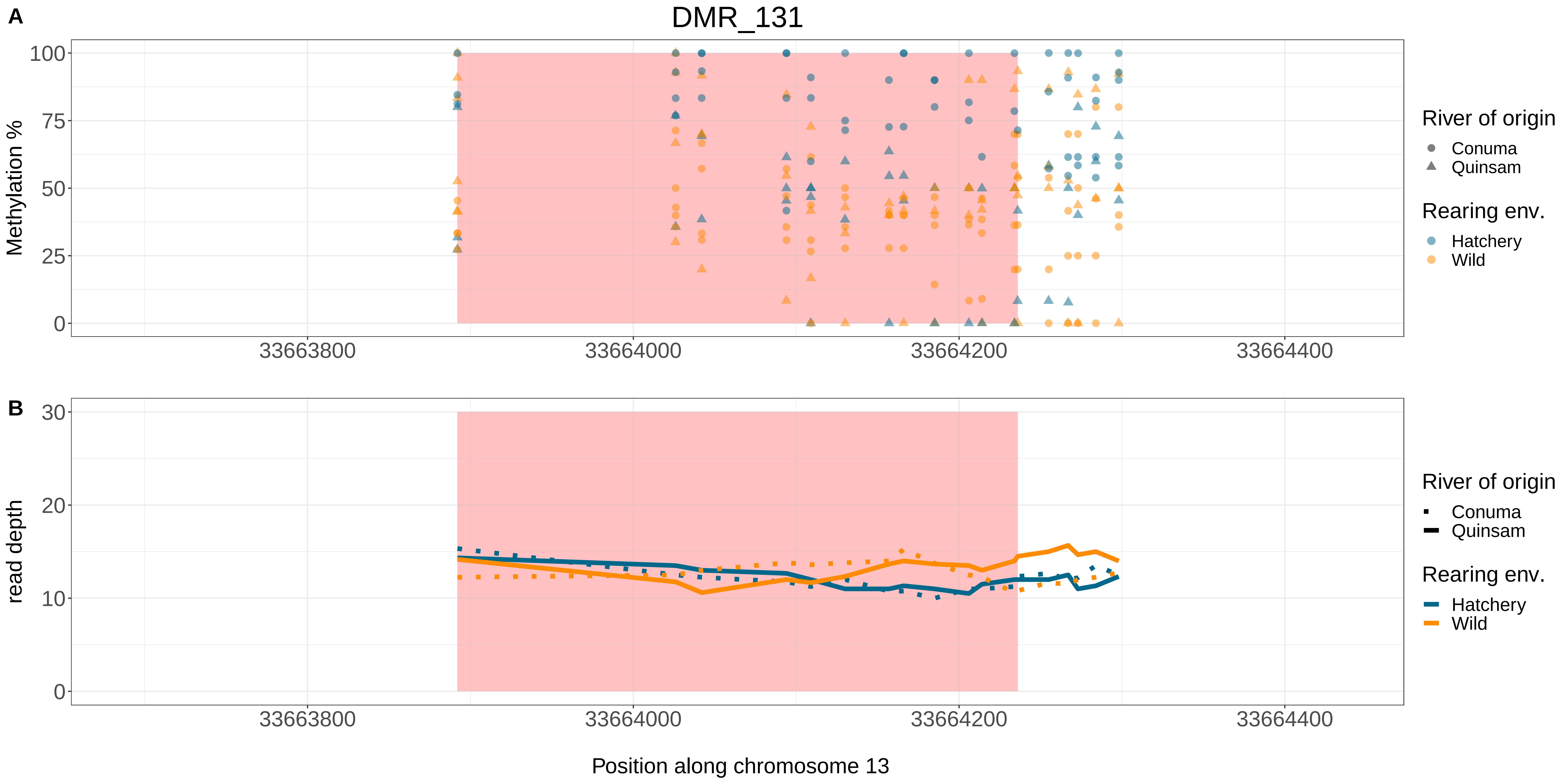
A
DMR_131
Methylation %
100
75
50
25
0
33663800
33664000
33664200
33664400
River of origin
Conuma
Quinsam
Rearing env.
Hatchery
Wild
B
read depth
30
20
10
0
33663800
33664000
33664200
33664400
River of origin
Conuma
Quinsam
Rearing env.
Hatchery
Wild
Position along chromosome 13

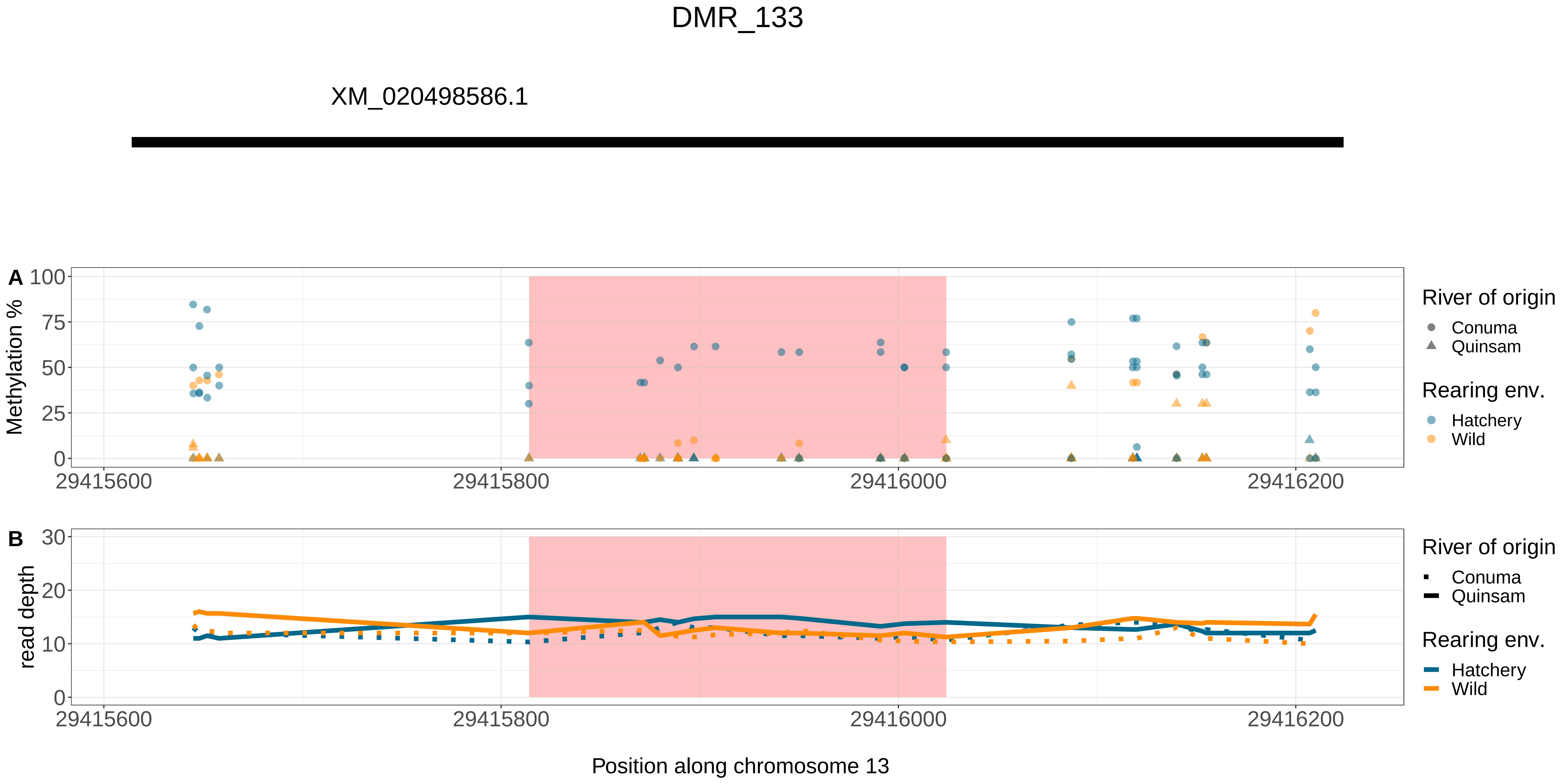
DMR_133
XM_020498586.1
A
Methylation %
100
75
50
25
0
29415600
29415800
29416000
29416200
River of origin
Conuma
Quinsam
Rearing env.
Hatchery
Wild
B
read depth
30
20
10
0
29415600
29415800
29416000
29416200
River of origin
Conuma
Quinsam
Rearing env.
Hatchery
Wild
Position along chromosome 13

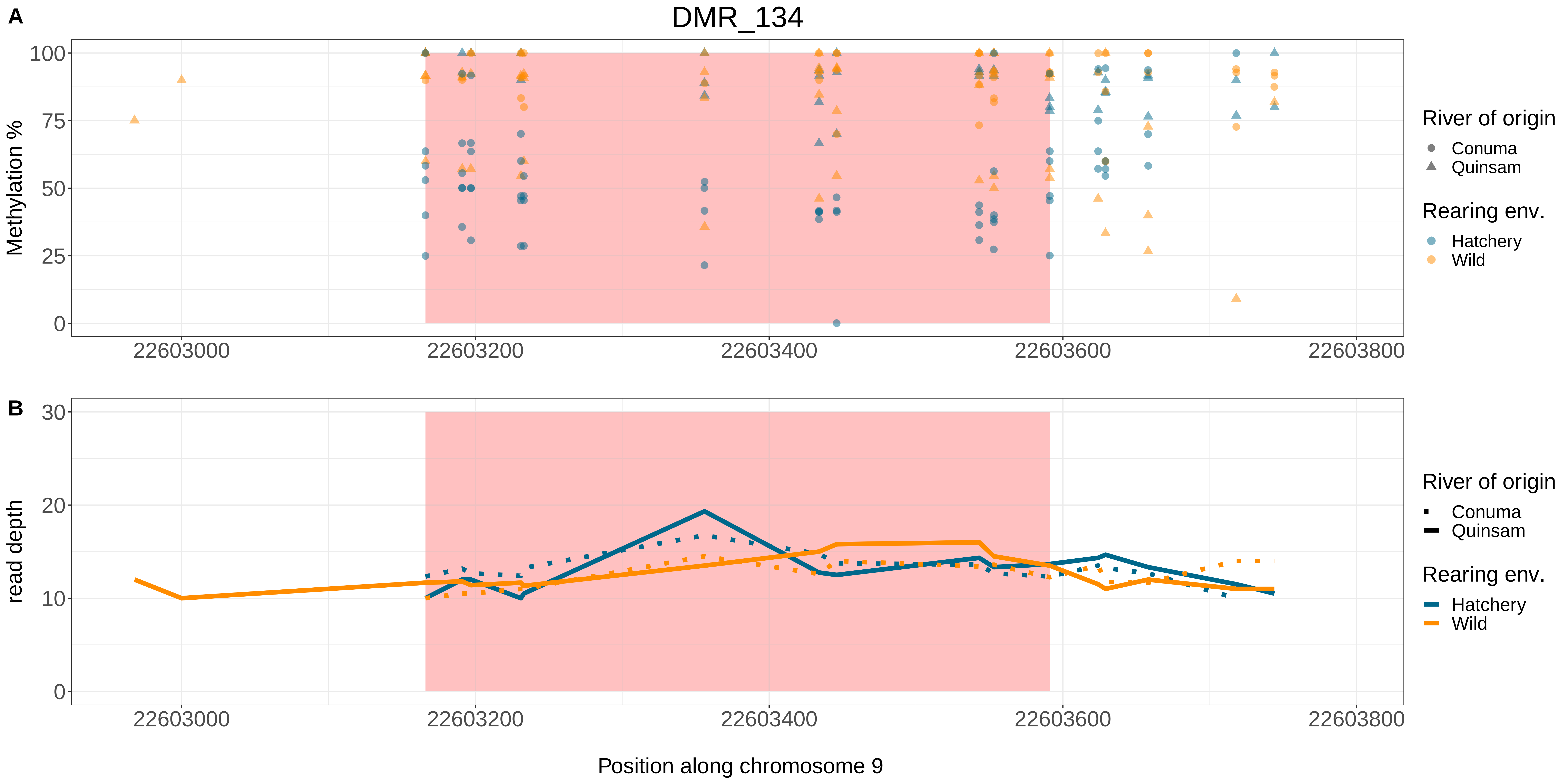
A
DMR_134
Methylation %
100
75
50
25
0
22603000
22603200
22603400
22603600
22603800
River of origin
Conuma
Quinsam
Rearing env.
Hatchery
Wild
B
read depth
30
20
10
0
22603000
22603200
22603400
22603600
22603800
River of origin
Conuma
Quinsam
Rearing env.
Hatchery
Wild
Position along chromosome 9

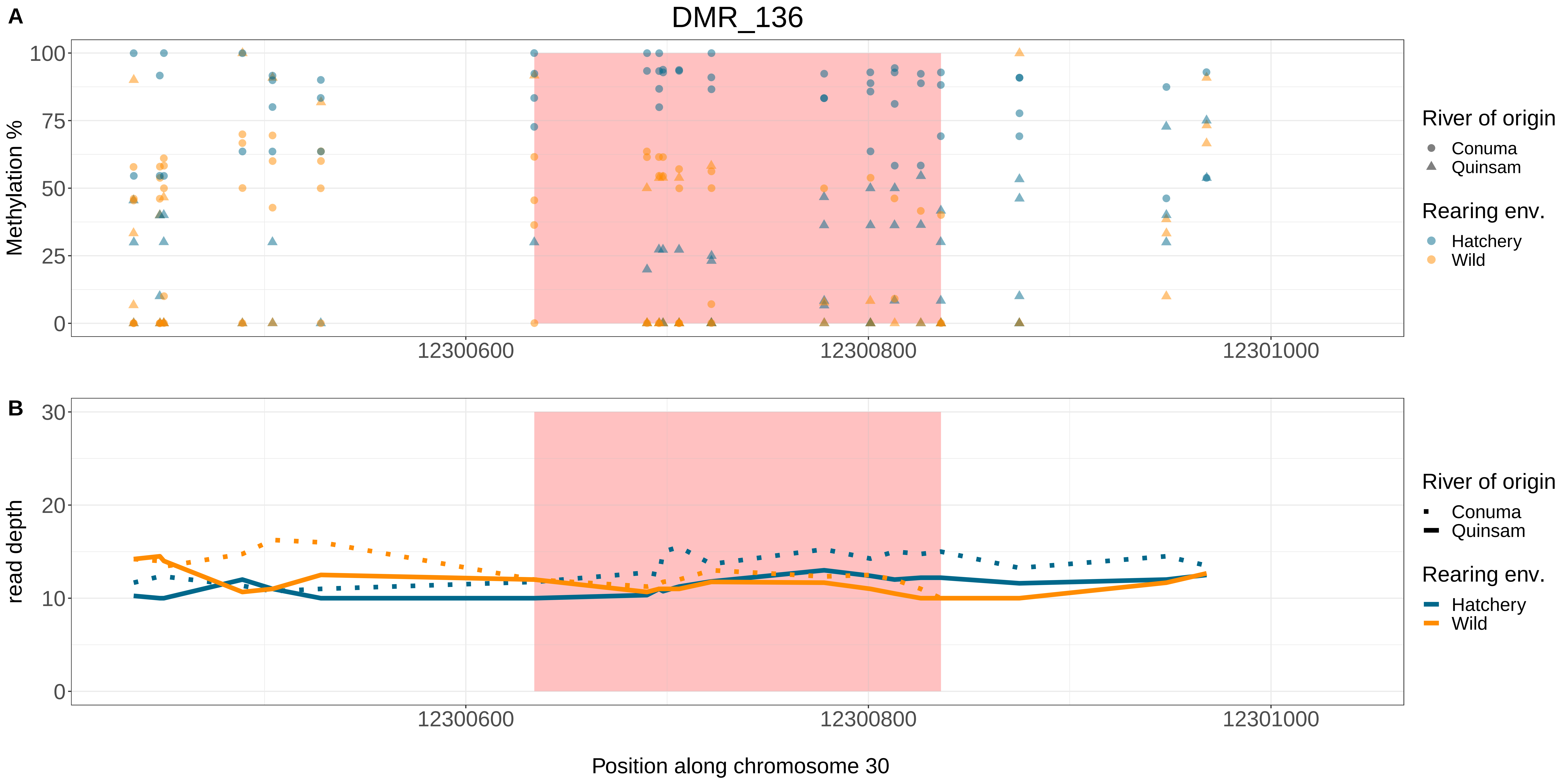

A
DMR_136
Methylation %
100
75
50
25
0
12300600
12300800
12301000
River of origin
Conuma
Quinsam
Rearing env.
Hatchery
Wild
B
read depth
30
20
10
0
12300600
12300800
12301000
River of origin
Conuma
Quinsam
Rearing env.
Hatchery
Wild
Position along chromosome 30

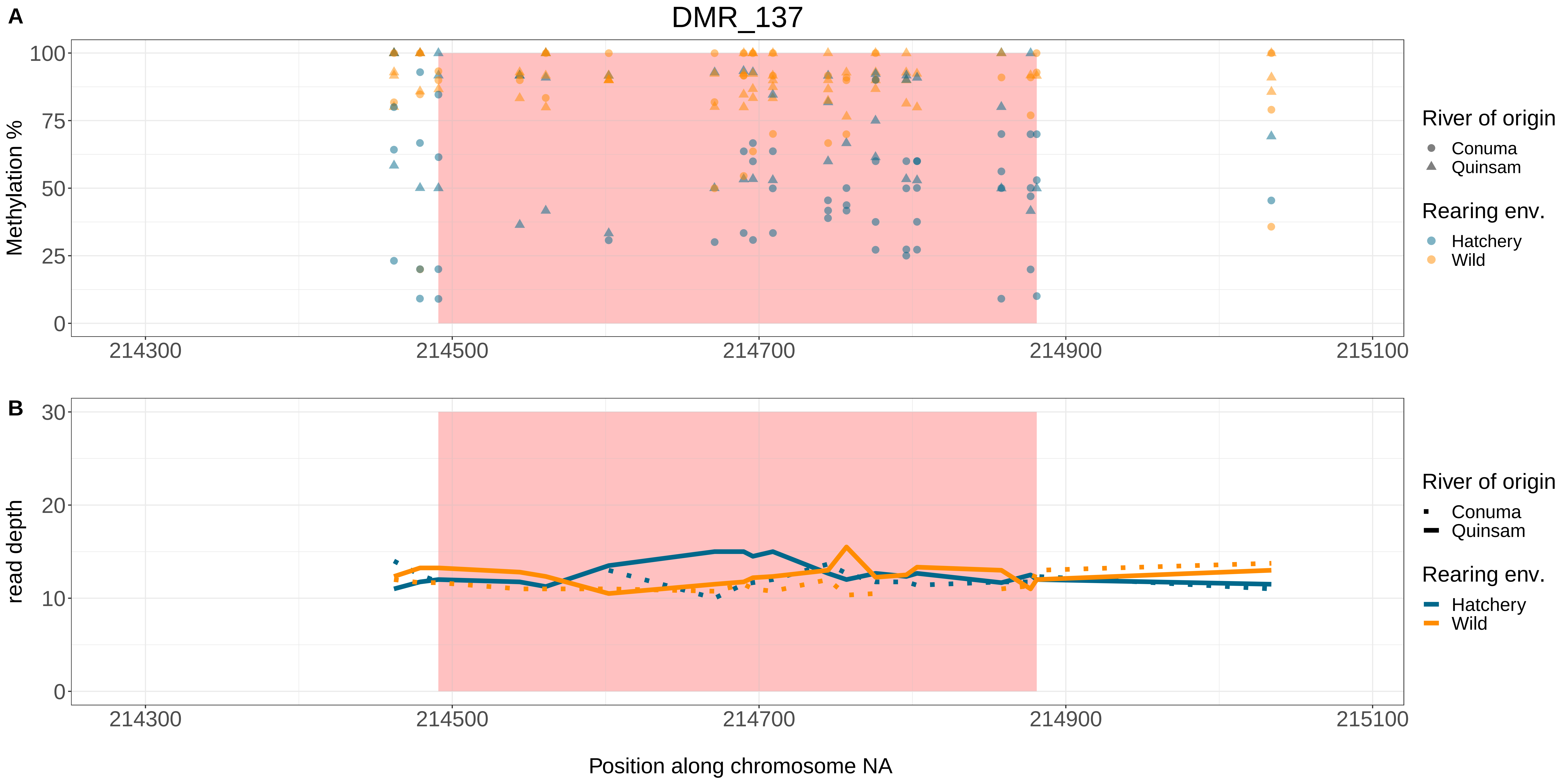
A
DMR_137
Methylation %
100
75
50
25
0
214300
214500
214700
214900
215100
River of origin
Conuma
Quinsam
Rearing env.
Hatchery
Wild
B
read depth
30
20
10
0
214300
214500
214700
214900
215100
River of origin
Conuma
Quinsam
Rearing env.
Hatchery
Wild
Position along chromosome NA

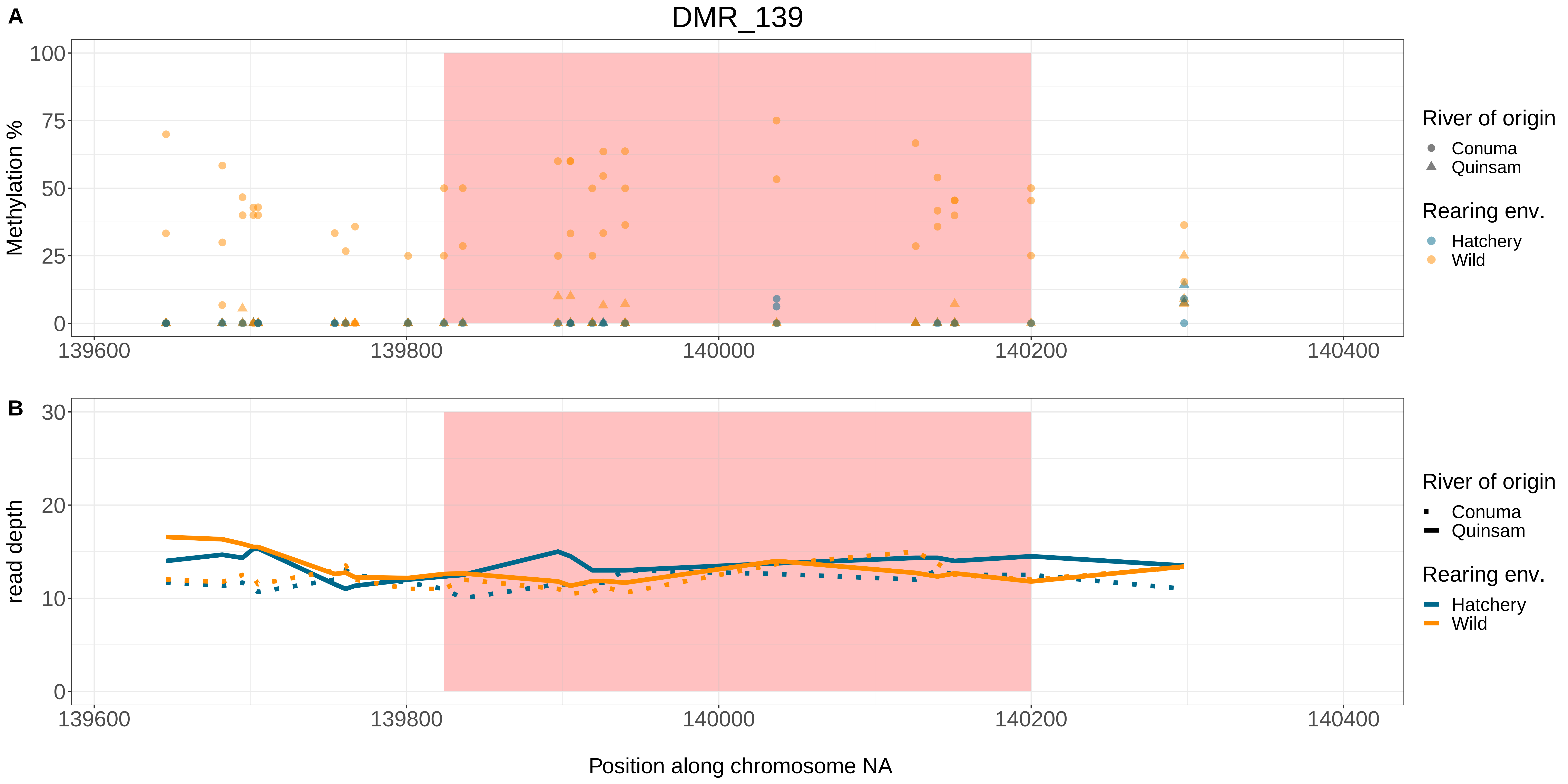

A
DMR_139
Methylation %
100
75
50
25
0
139600
139800
140000
140200
140400
River of origin
Conuma
Quinsam
Rearing env.
Hatchery
Wild
B
read depth
30
20
10
0
139600
139800
140000
140200
140400
River of origin
Conuma
Quinsam
Rearing env.
Hatchery
Wild
Position along chromosome NA

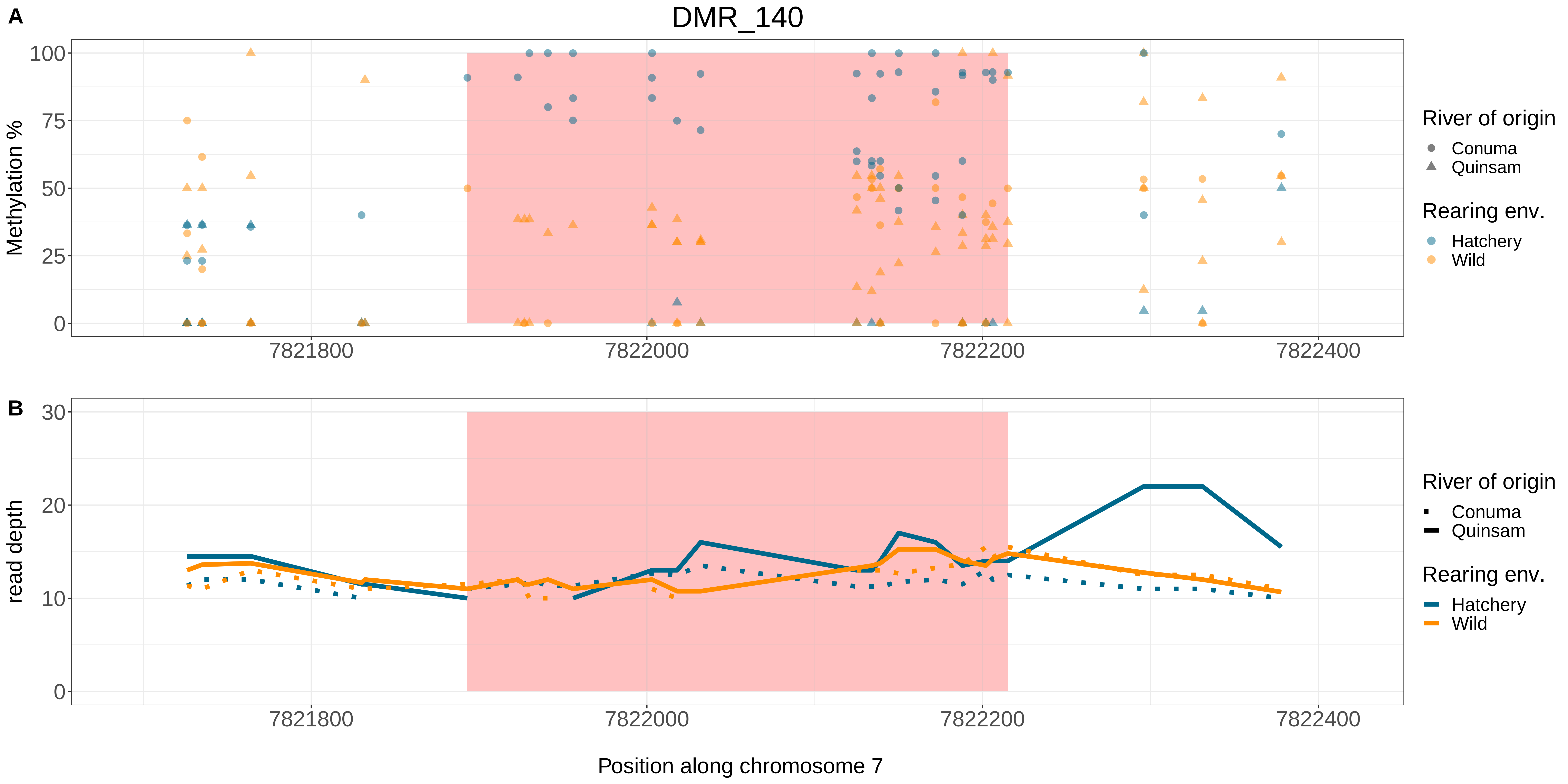
A
DMR_140
Methylation %
100
75
50
25
0
7821800
7822000
7822200
7822400
River of origin
Conuma
Quinsam
Rearing env.
Hatchery
Wild
B
read depth
30
20
10
0
7821800
7822000
7822200
7822400
River of origin
Conuma
Quinsam
Rearing env.
Hatchery
Wild
Position along chromosome 7

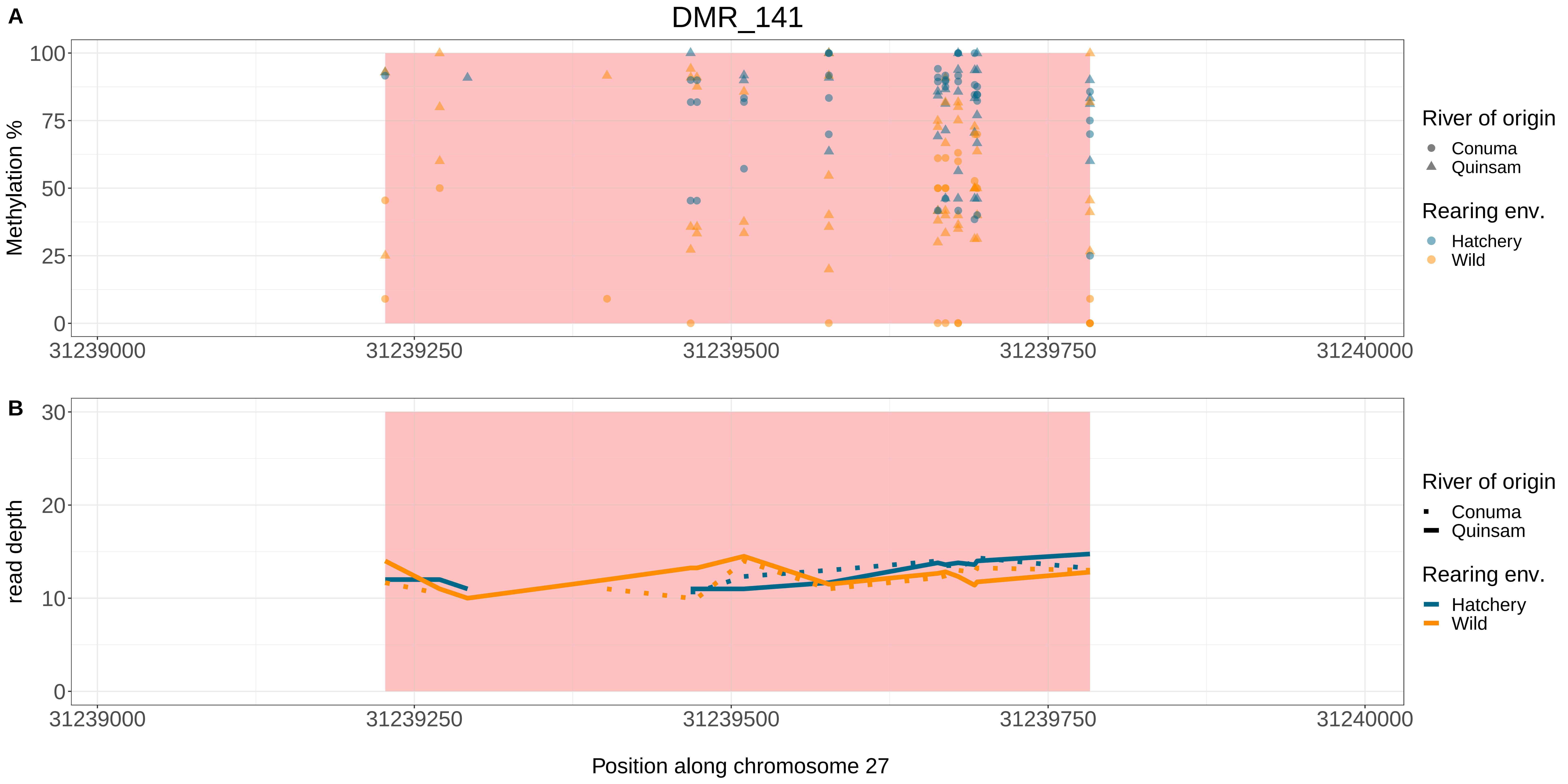
A
DMR_141
Methylation %
100
75
50
25
0
31239000
31239250
31239500
31239750
31240000
River of origin
Conuma
Quinsam
Rearing env.
Hatchery
Wild
B
read depth
30
20
10
0
31239000
31239250
31239500
31239750
31240000
River of origin
Conuma
Quinsam
Rearing env.
Hatchery
Wild
Position along chromosome 27

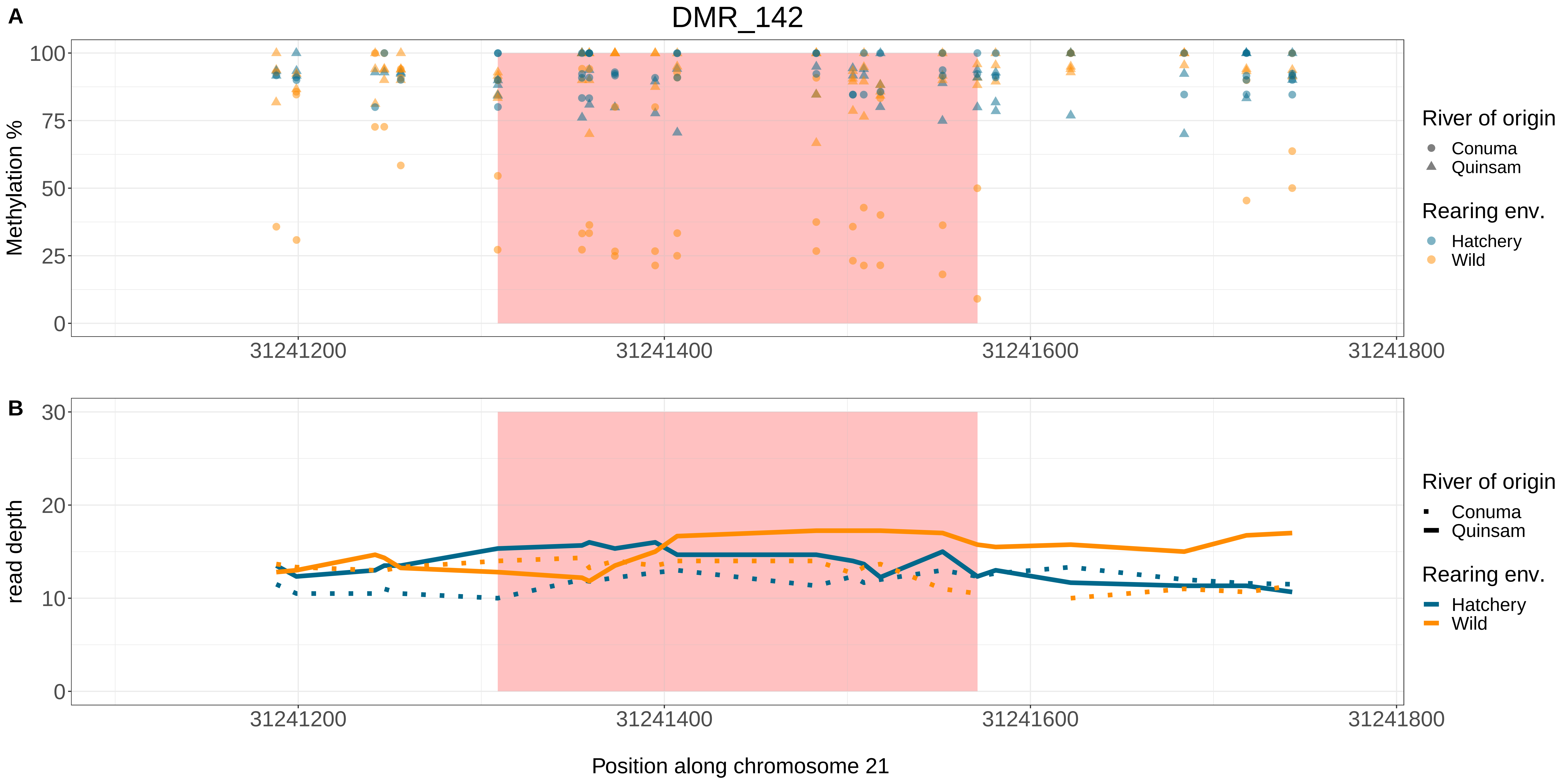

A
DMR_142
Methylation %
100
75
50
25
0
31241200
31241400
31241600
31241800
River of origin
Conuma
Quinsam
Rearing env.
Hatchery
Wild
B
read depth
30
20
10
0
31241200
31241400
31241600
31241800
River of origin
Conuma
Quinsam
Rearing env.
Hatchery
Wild
Position along chromosome 21

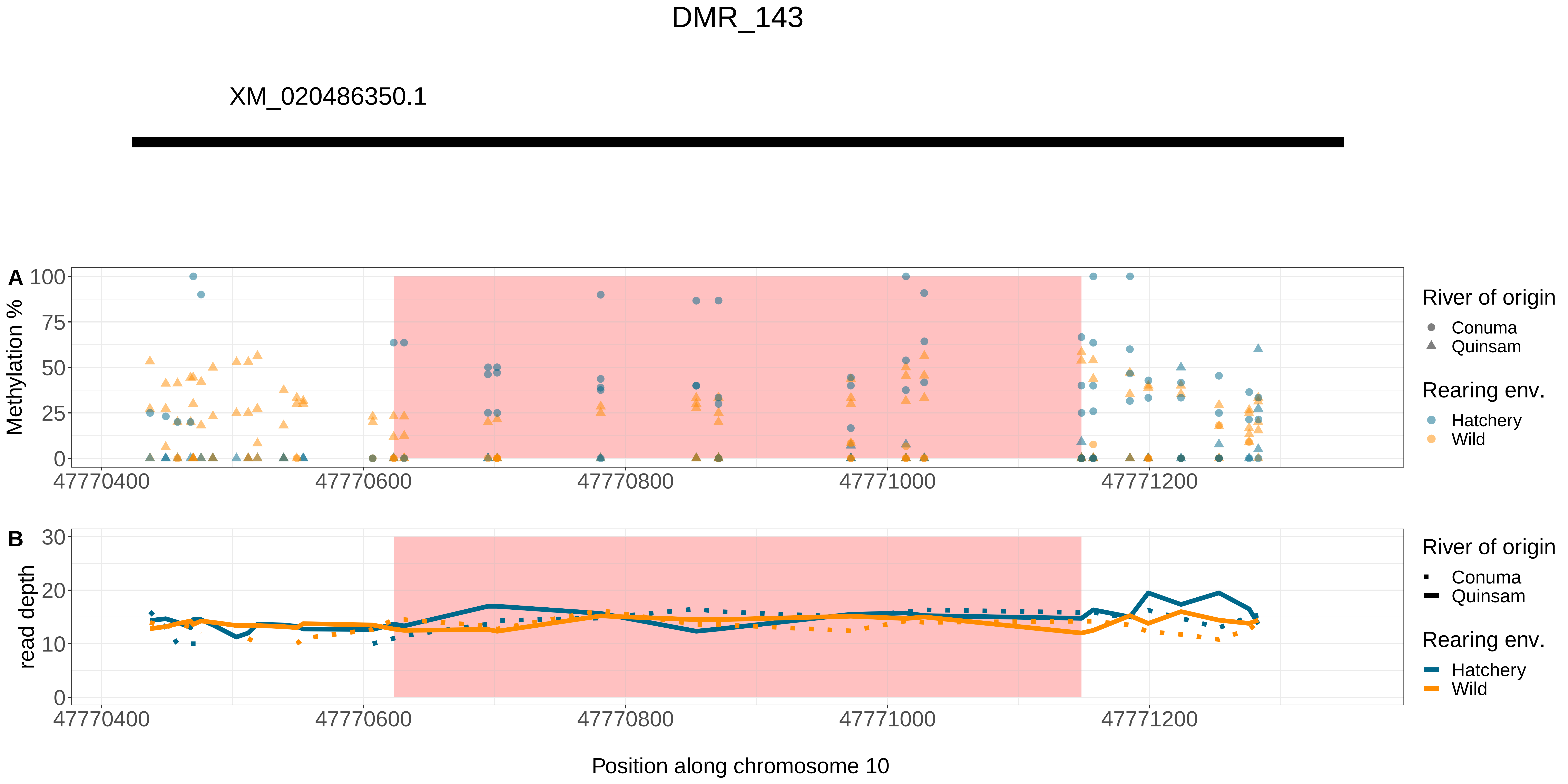
DMR_143
XM_020486350.1
A
Methylation %
100
75
50
25
0
47770400
47770600
47770800
47771000
47771200
River of origin
Conuma
Quinsam
Rearing env.
Hatchery
Wild
B
read depth
30
20
10
0
Position along chromosome 10

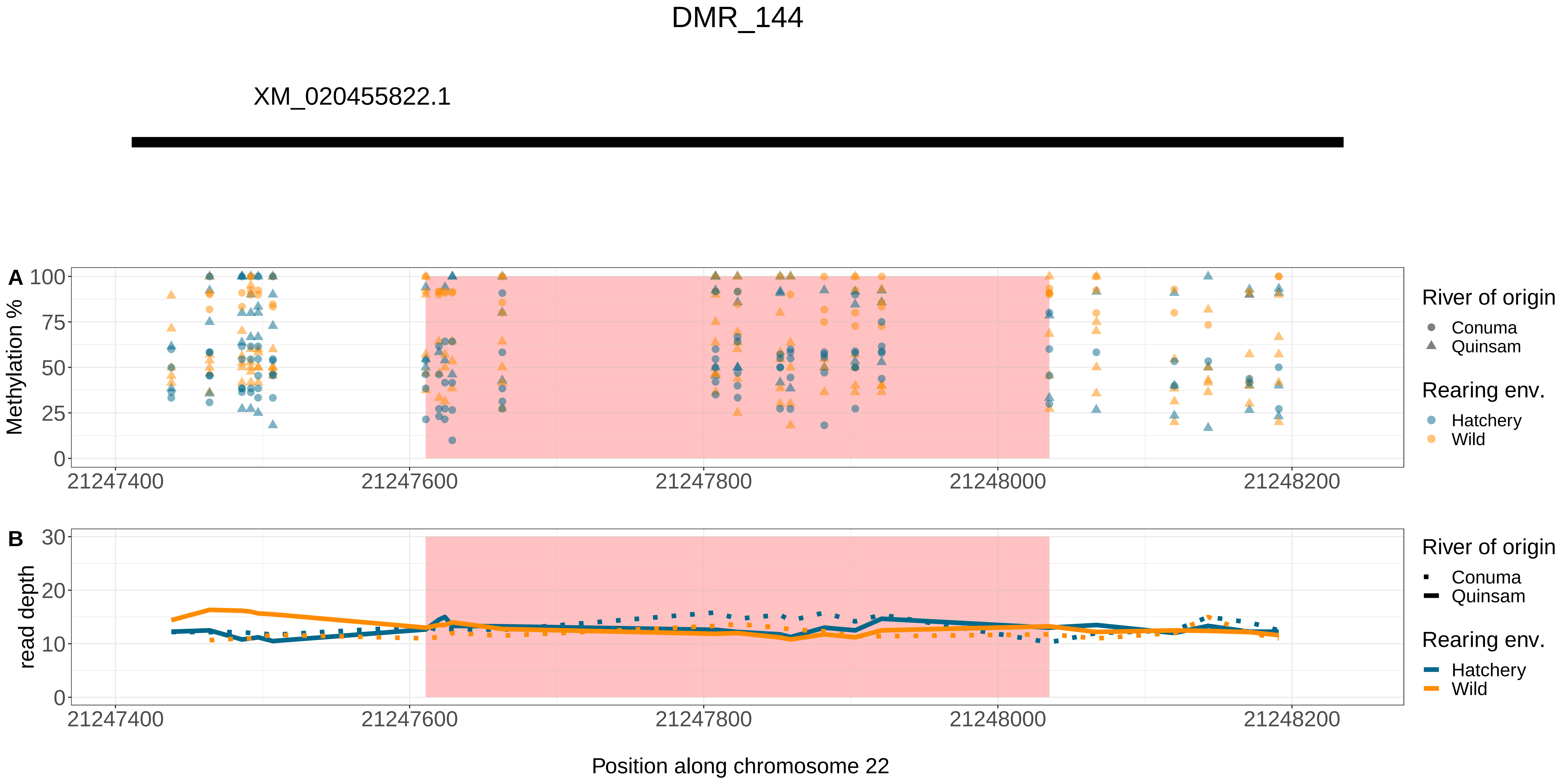
DMR_144
XM_020455822.1
A
Methylation %
100
75
50
25
0
21247400
21247600
21247800
21248000
21248200
River of origin
Conuma
Quinsam
Rearing env.
Hatchery
Wild
B
read depth
30
20
10
0
21247400
21247600
21247800
21248000
21248200
River of origin
Conuma
Quinsam
Rearing env.
Hatchery
Wild
Position along chromosome 22

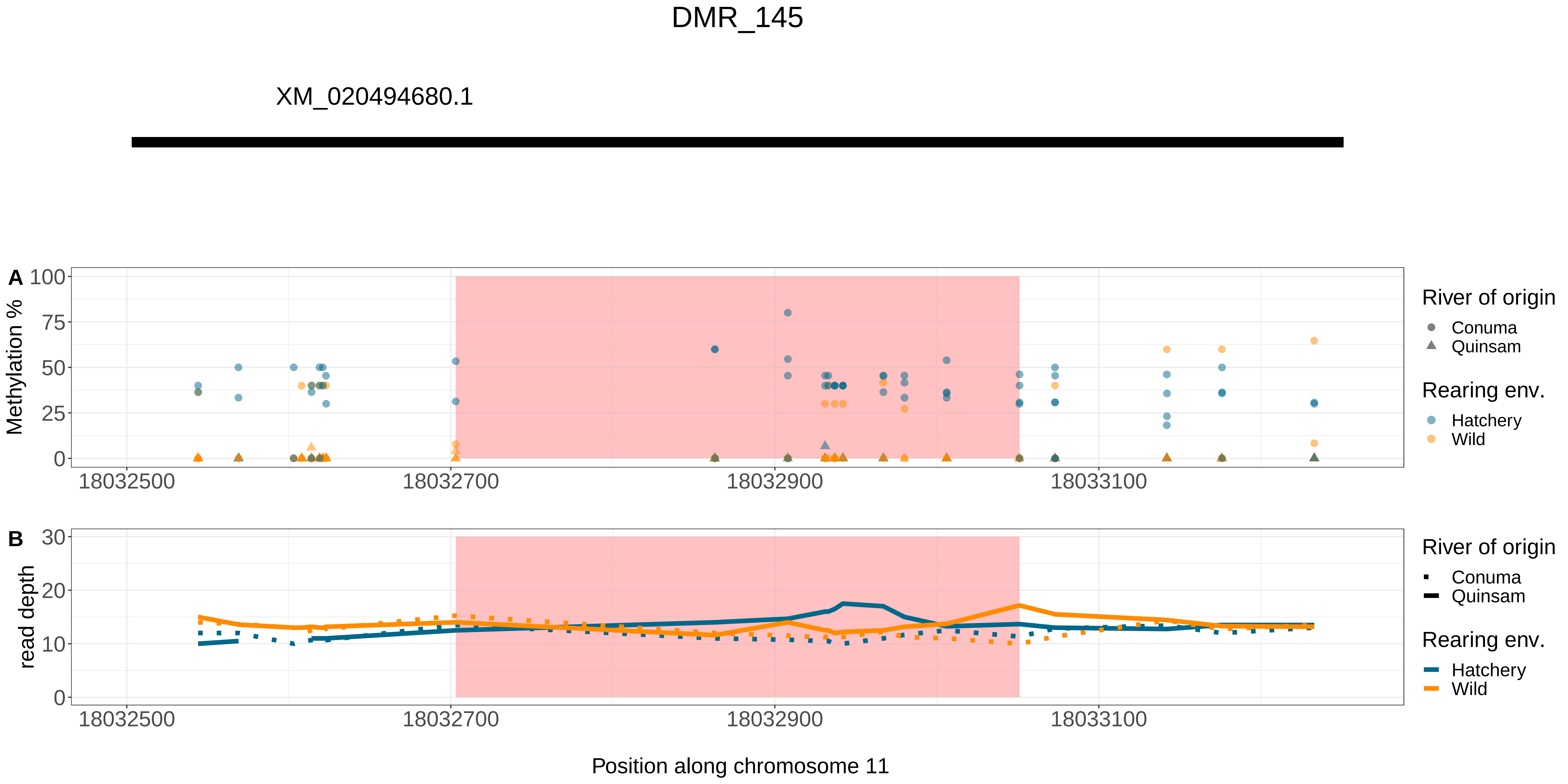

DMR_145
XM_020494680.1
A
Methylation %
100
75
50
25
0
18032500
18032700
18032900
18033100
River of origin
Conuma
Quinsam
Rearing env.
Hatchery
Wild
B
read depth
30
20
10
0
18032500
18032700
18032900
18033100
River of origin
Conuma
Quinsam
Rearing env.
Hatchery
Wild
Position along chromosome 11

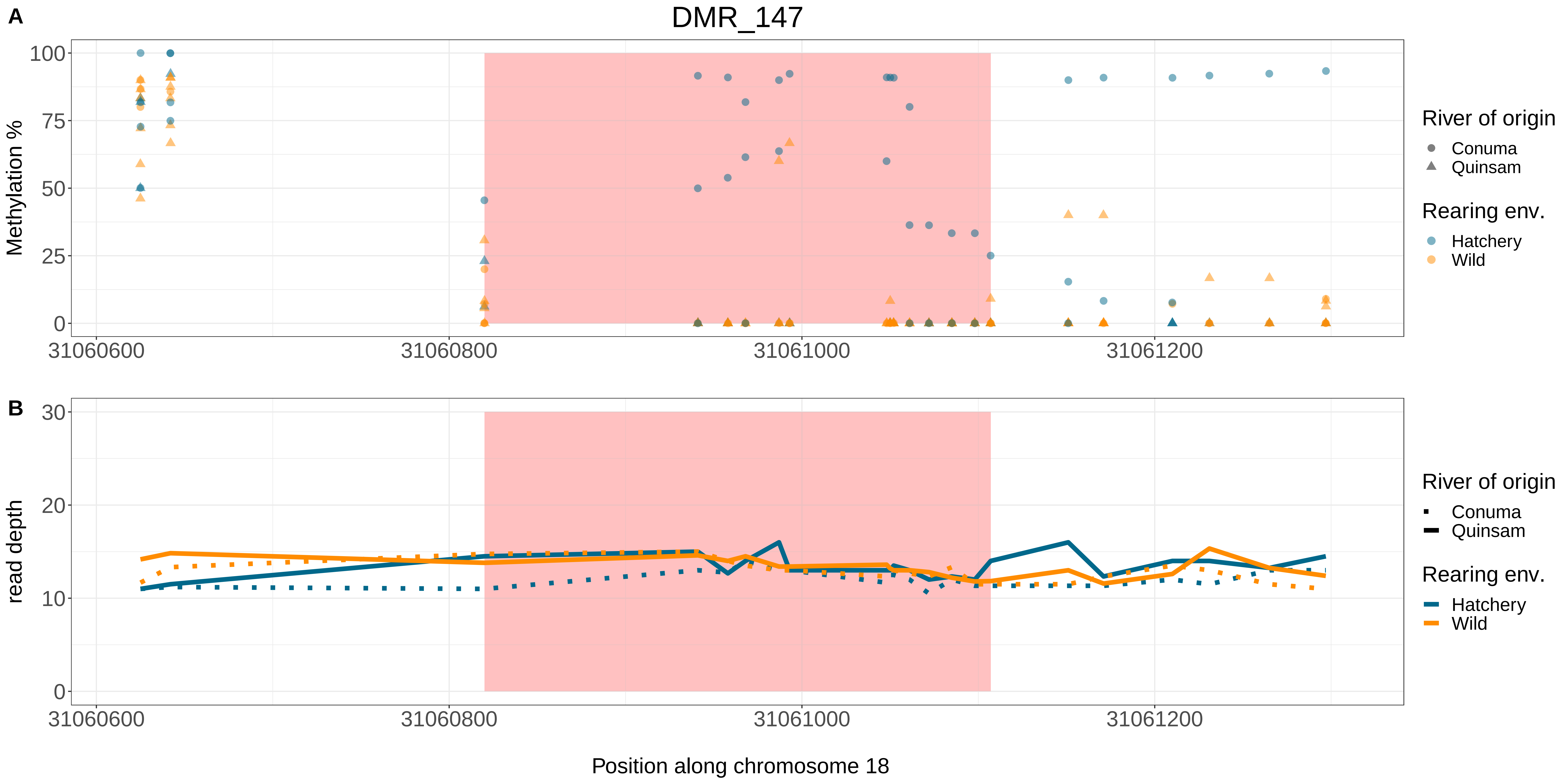

A
DMR_147
Methylation %
100
75
50
25
0
31060600
31060800
31061000
31061200
River of origin
Conuma
Quinsam
Rearing env.
Hatchery
Wild
B
read depth
30
20
10
0
31060600
31060800
31061000
31061200
River of origin
Conuma
Quinsam
Rearing env.
Hatchery
Wild
Position along chromosome 18

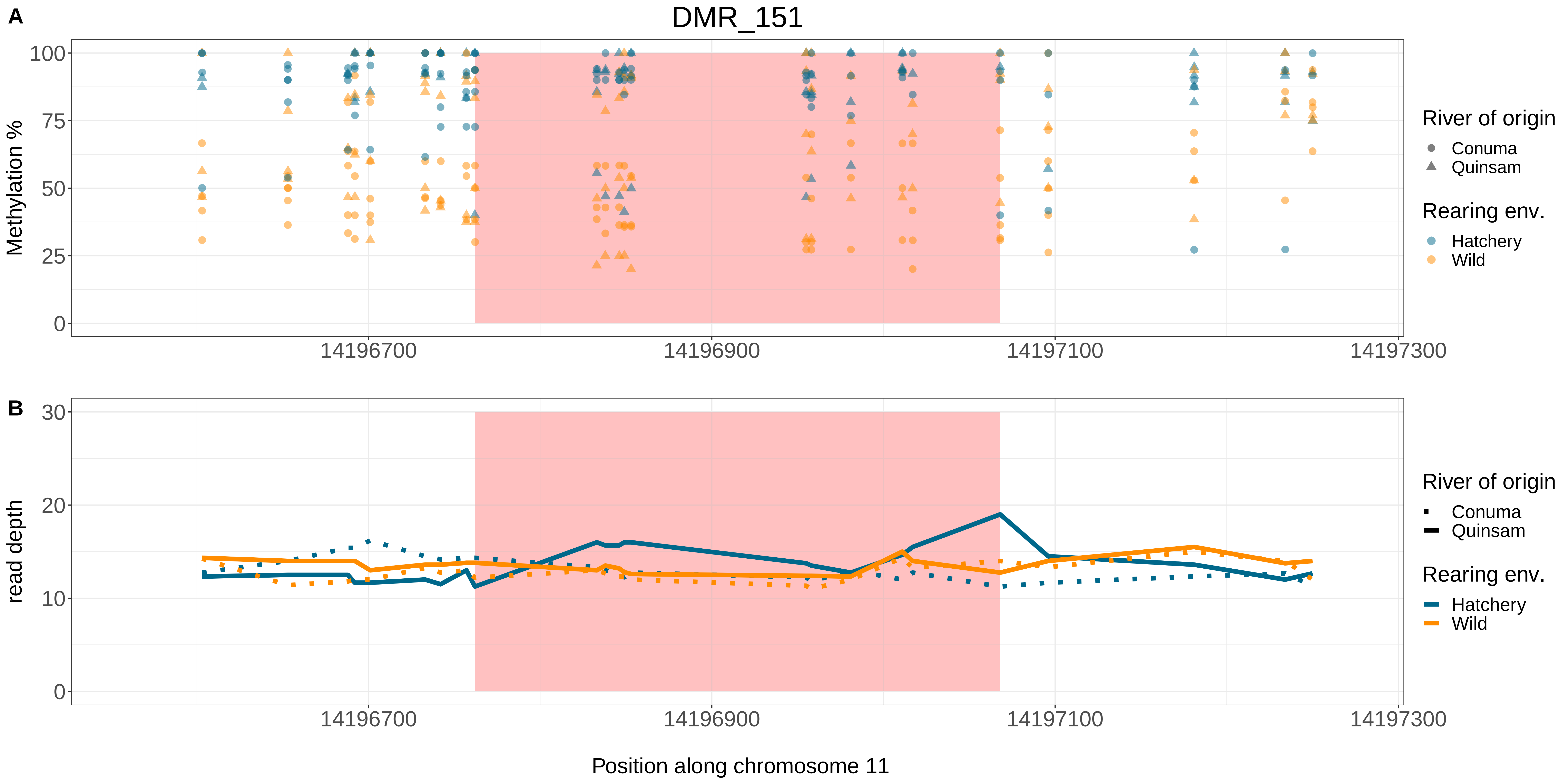

A
DMR_151
Methylation %
100
75
50
25
0
14196700
14196900
14197100
14197300
River of origin
Conuma
Quinsam
Rearing env.
Hatchery
Wild
B
read depth
30
20
10
0
14196700
14196900
14197100
14197300
River of origin
Conuma
Quinsam
Rearing env.
Hatchery
Wild
Position along chromosome 11

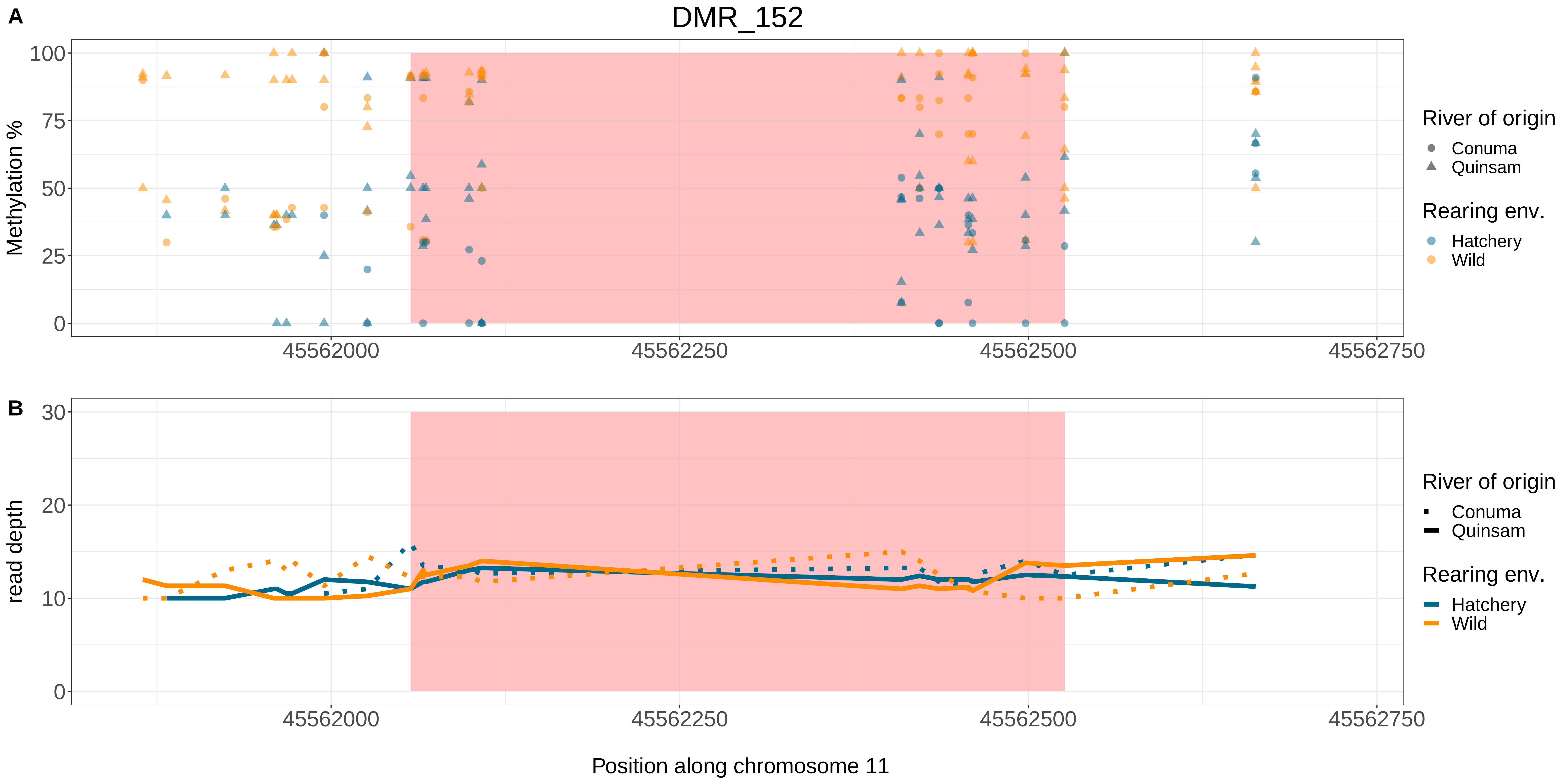

A
DMR_152
Methylation %
100
75
50
25
0
45562000
45562250
45562500
45562750
River of origin
Conuma
Quinsam
Rearing env.
Hatchery
Wild
B
read depth
30
20
10
0
45562000
45562250
45562500
45562750
River of origin
Conuma
Quinsam
Rearing env.
Hatchery
Wild
Position along chromosome 11

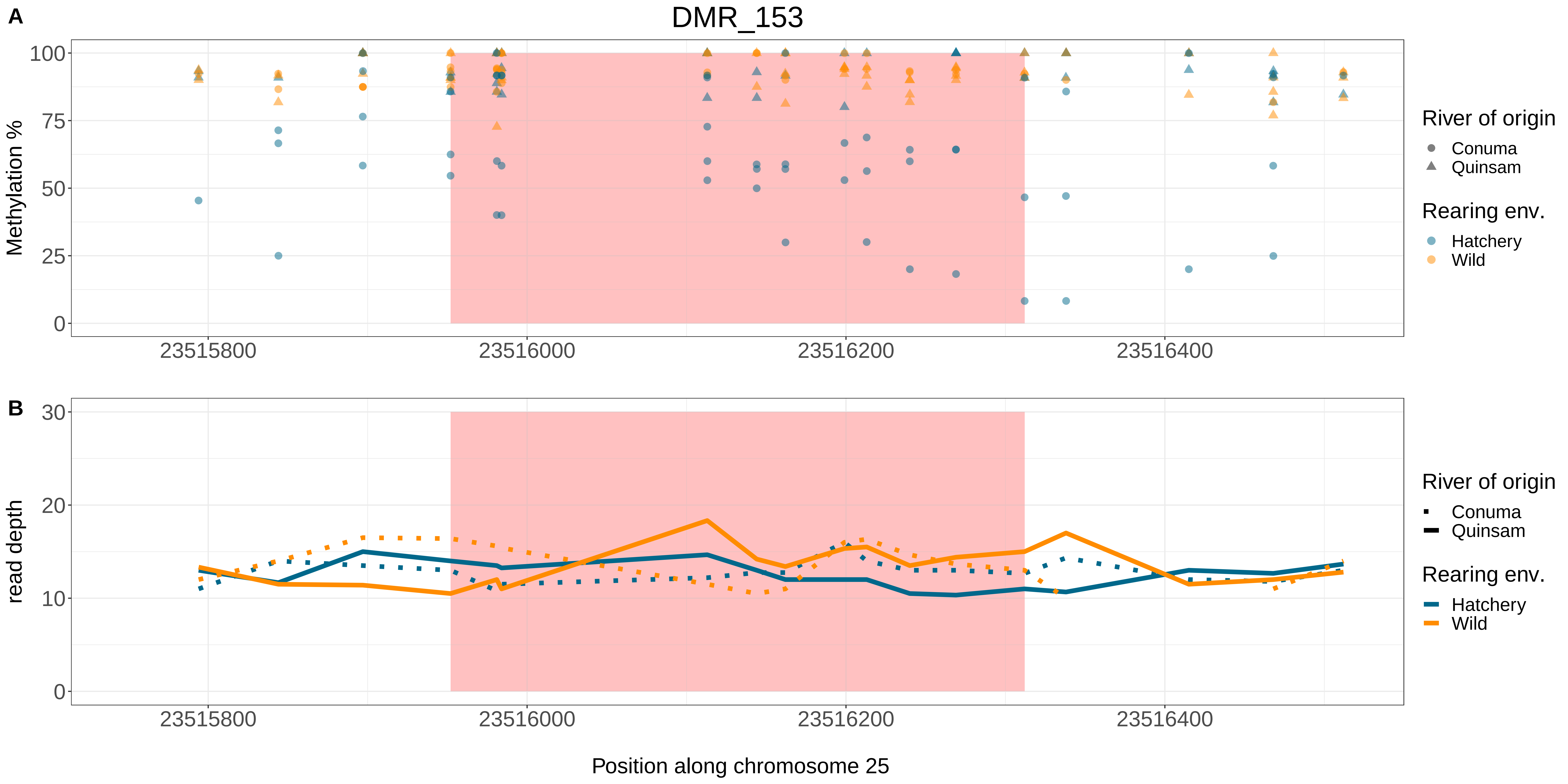
A
DMR_153
Methylation %
100
75
50
25
0
23515800
23516000
23516200
23516400
River of origin
Conuma
Quinsam
Rearing env.
Hatchery
Wild
B
read depth
30
20
10
0
23515800
23516000
23516200
23516400
River of origin
Conuma
Quinsam
Rearing env.
Hatchery
Wild
Position along chromosome 25

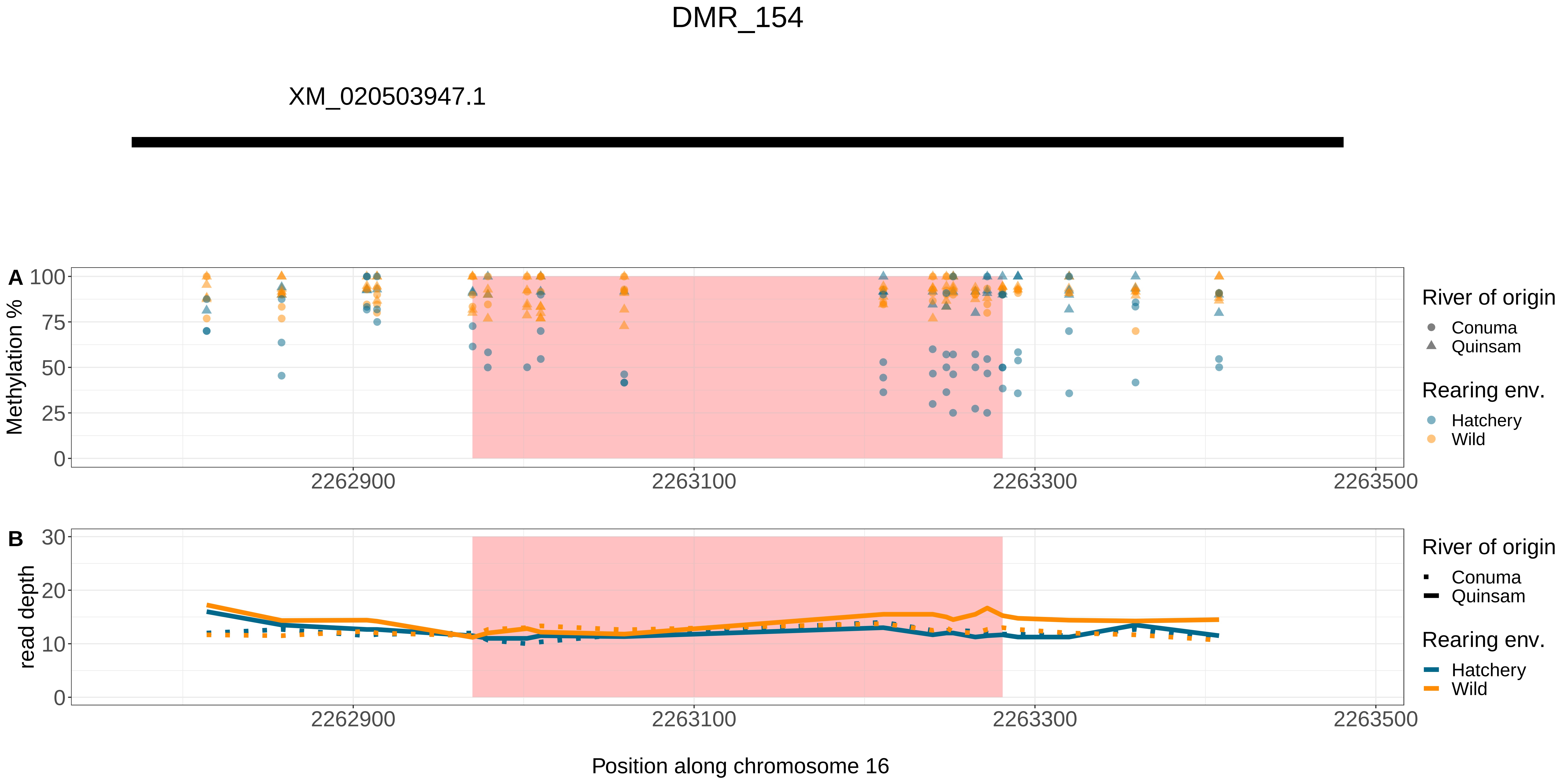
DMR_154
XM_020503947.1
A
Methylation %
100
75
50
25
0
2262900
2263100
2263300
2263500
River of origin
Conuma
Quinsam
Rearing env.
Hatchery
Wild
B
read depth
30
20
10
0
2262900
2263100
2263300
2263500
River of origin
Conuma
Quinsam
Rearing env.
Hatchery
Wild
Position along chromosome 16

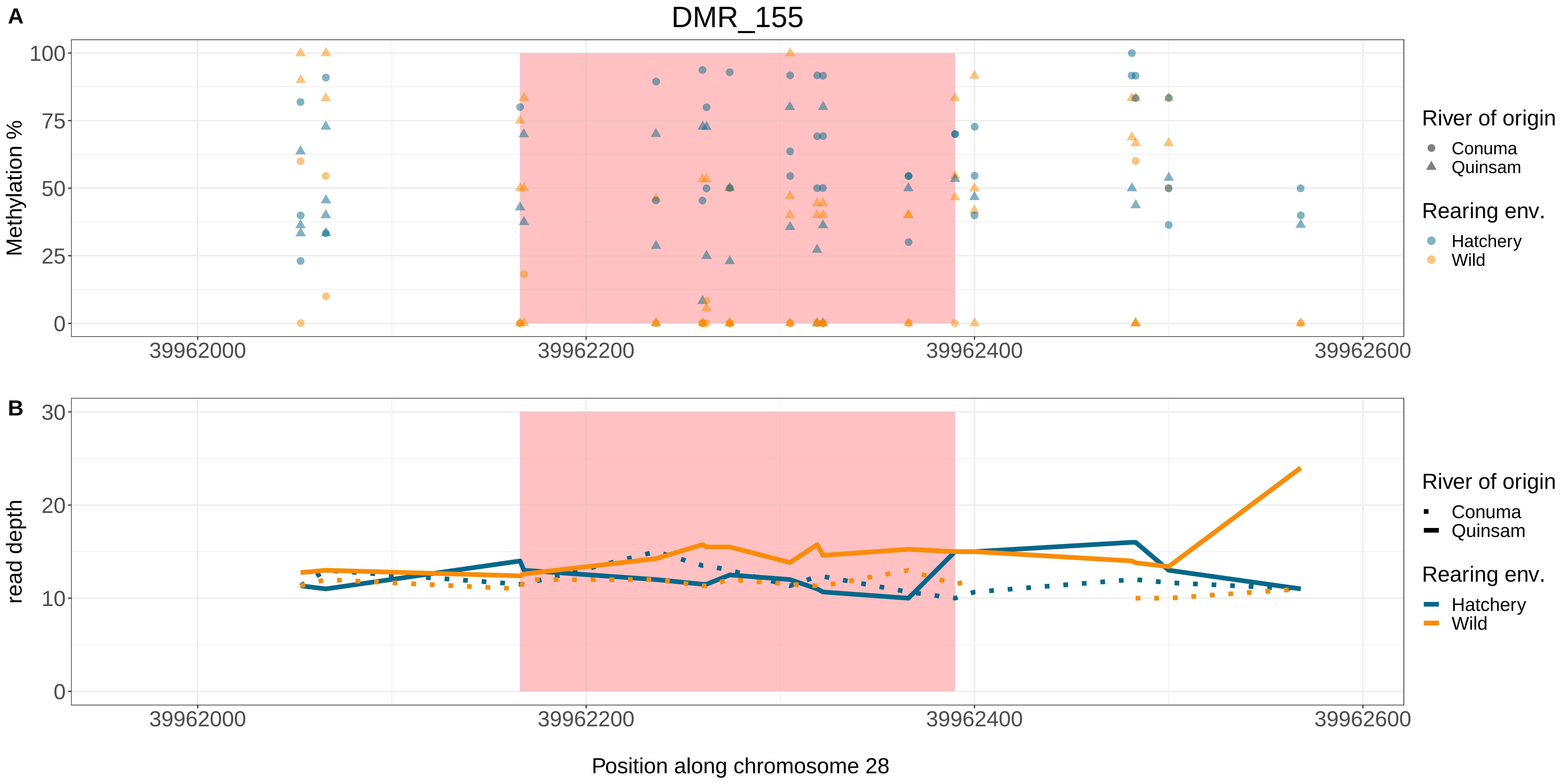

A
DMR_155
Methylation %
100
75
50
25
0
39962000
39962200
39962400
39962600
River of origin
Conuma
Quinsam
Rearing env.
Hatchery
Wild
B
read depth
30
20
10
0
39962000
39962200
39962400
39962600
River of origin
Conuma
Quinsam
Rearing env.
Hatchery
Wild
Position along chromosome 28

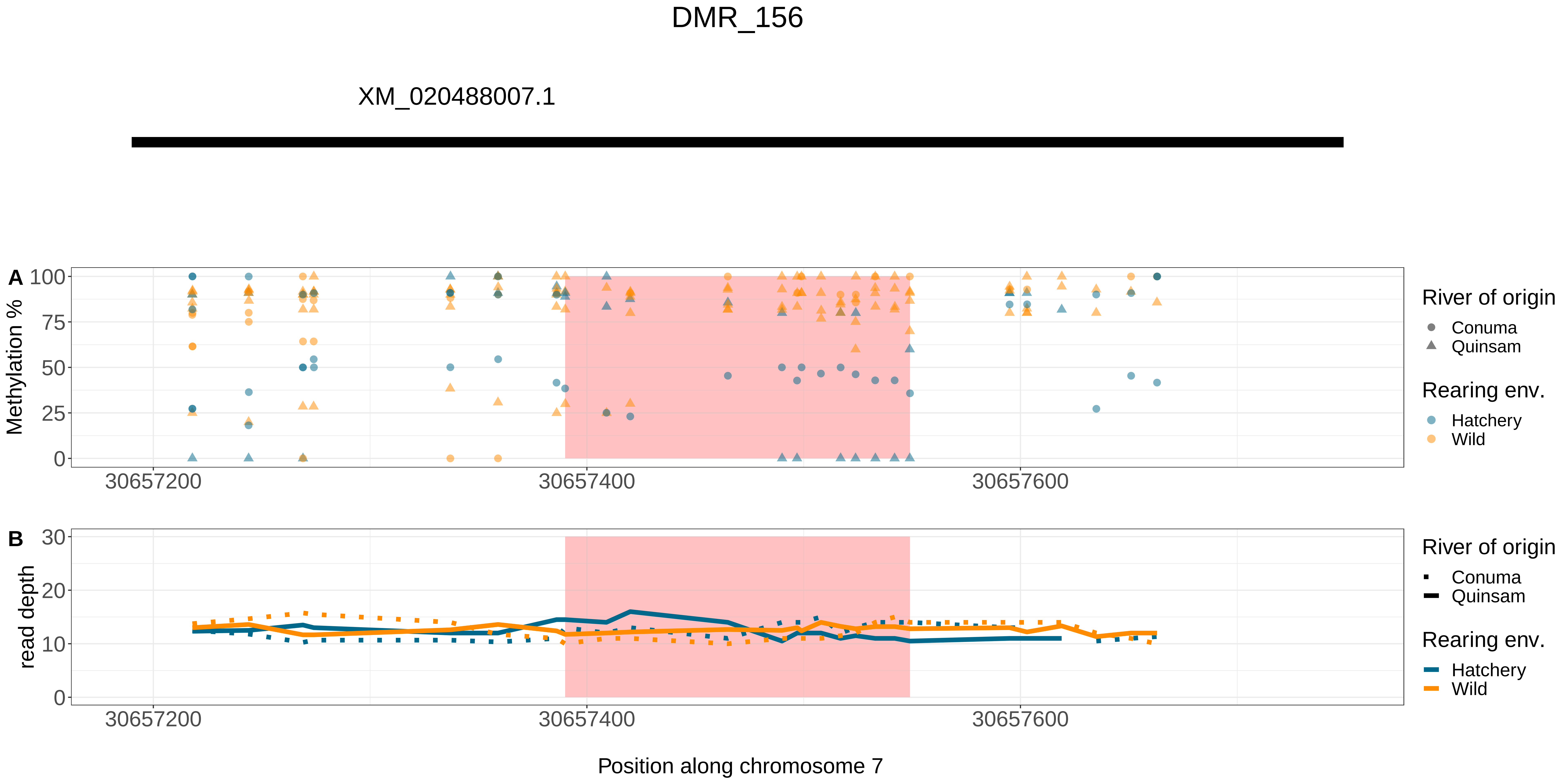

DMR_156
XM_020488007.1
A
Methylation %
100
75
50
25
0
30657200
30657400
30657600
River of origin
Conuma
Quinsam
Rearing env.
Hatchery
Wild
B
read depth
30
20
10
0
30657200
30657400
30657600
River of origin
Conuma
Quinsam
Rearing env.
Hatchery
Wild
Position along chromosome 7

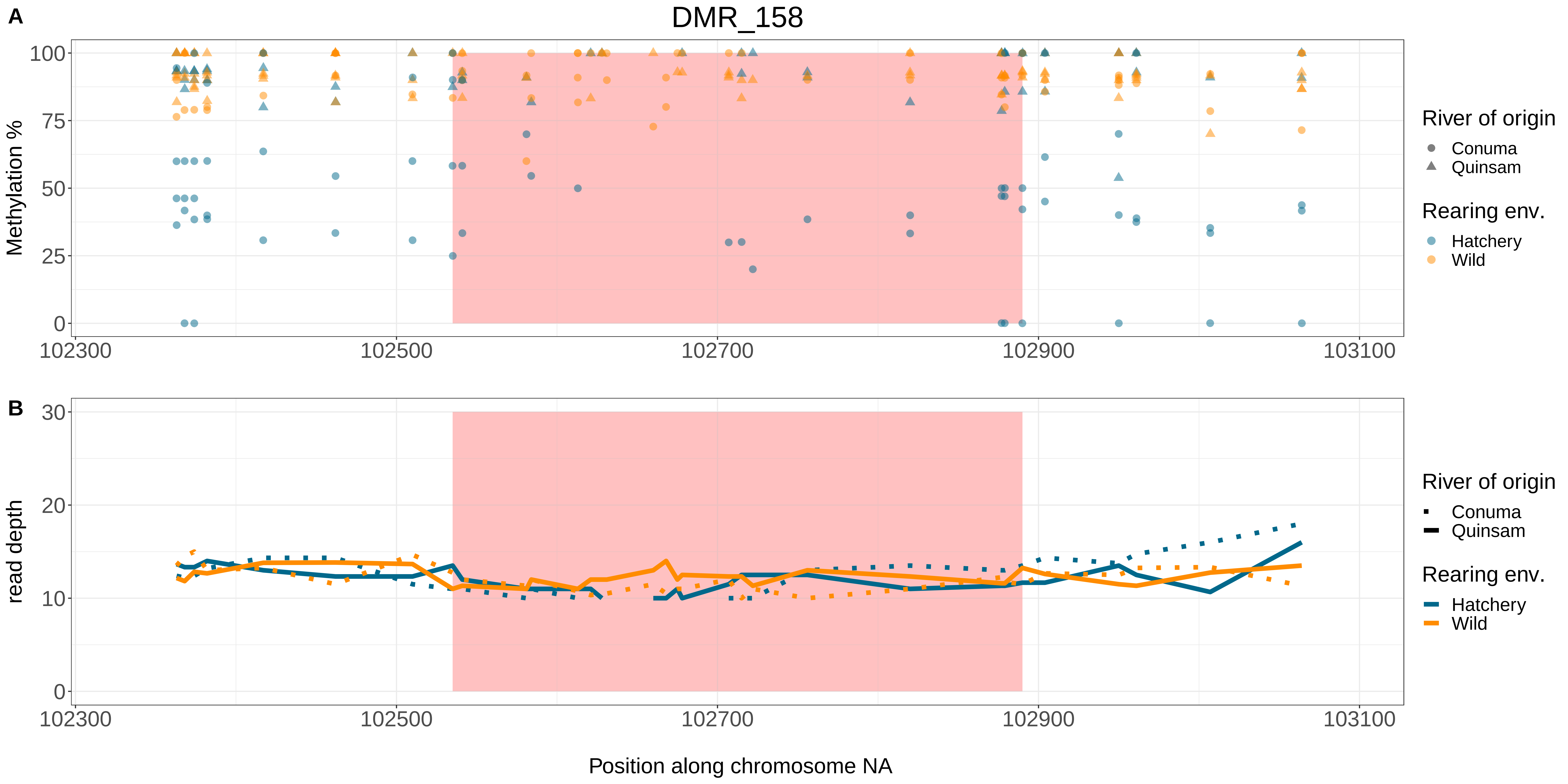

A
DMR_158
Methylation %
100
75
50
25
0
102300
102500
102700
102900
103100
River of origin
Conuma
Quinsam
Rearing env.
Hatchery
Wild
B
read depth
30
20
10
0
102300
102500
102700
102900
103100
River of origin
Conuma
Quinsam
Rearing env.
Hatchery
Wild
Position along chromosome NA

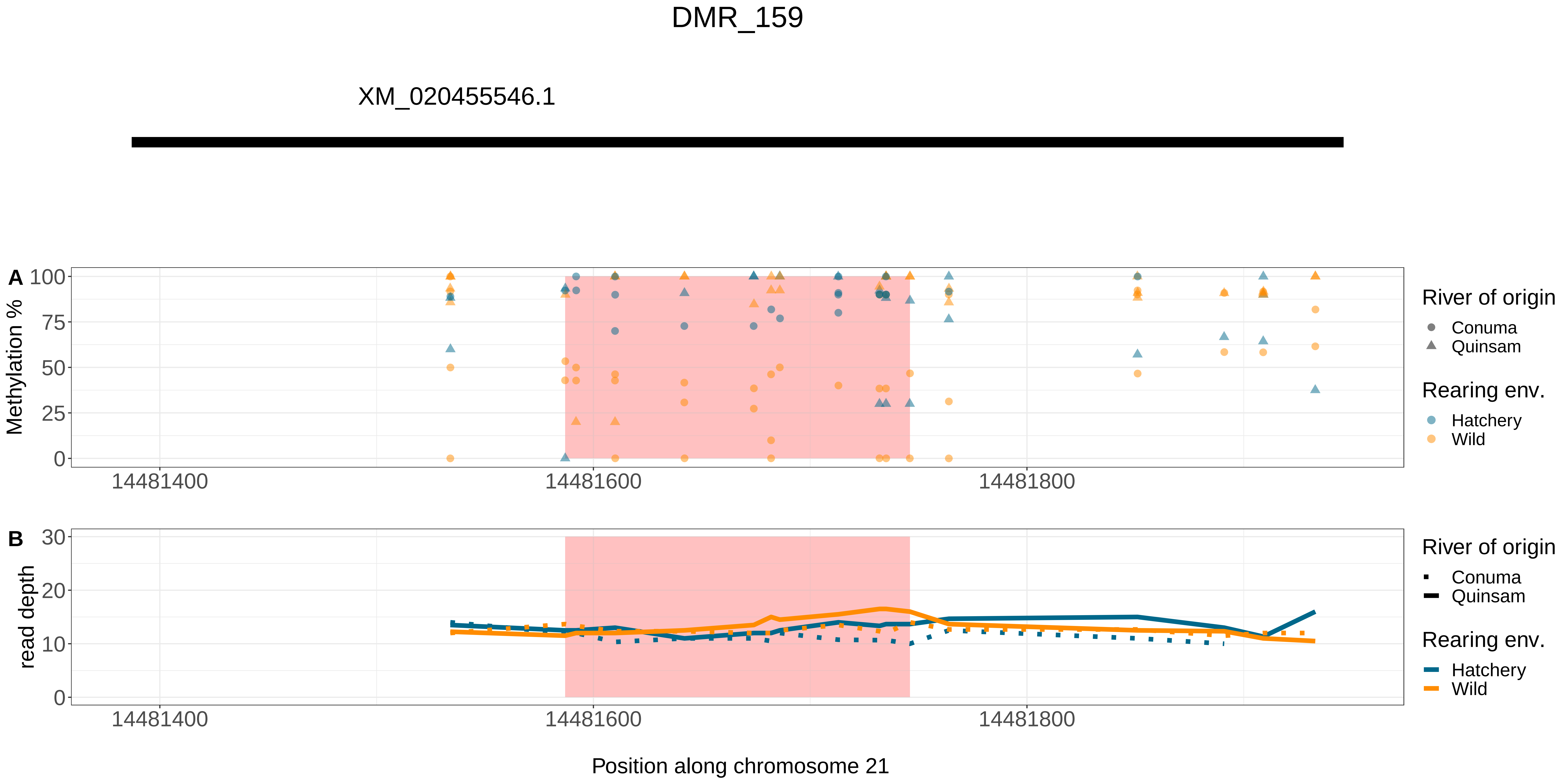
DMR_159
XM_020455546.1
A
B
Methylation %
100
75
50
25
0
read depth
30
20
10
0
14481400
14481600
14481800
Position along chromosome 21
River of origin
Conuma
Quinsam
Rearing env.
Hatchery
Wild

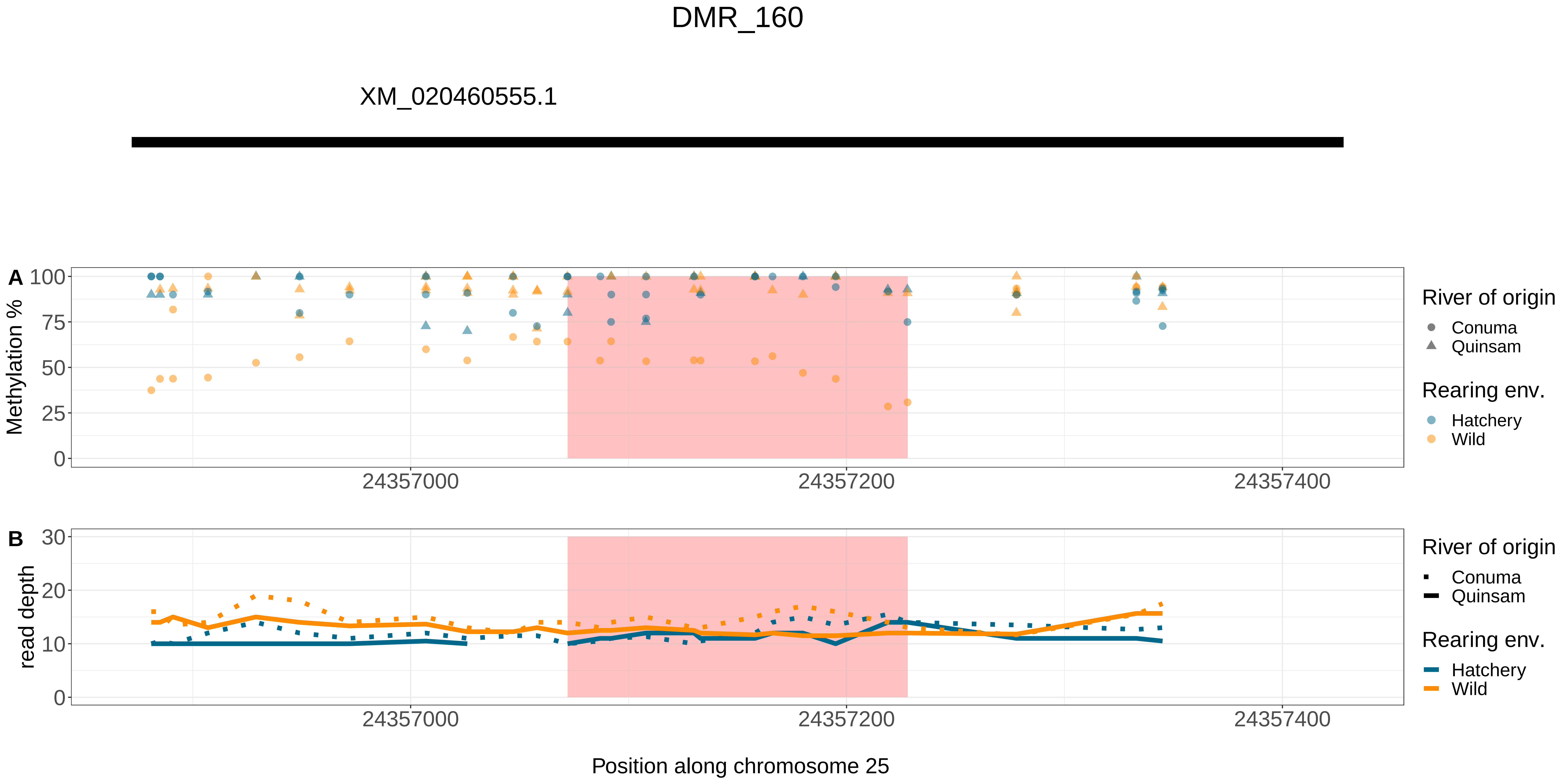
DMR_160
XM_020460555.1
A
Methylation %
100
75
50
25
0
24357000
24357200
24357400
River of origin
Conuma
Quinsam
Rearing env.
Hatchery
Wild
B
read depth
30
20
10
0
24357000
24357200
24357400
River of origin
Conuma
Quinsam
Rearing env.
Hatchery
Wild
Position along chromosome 25

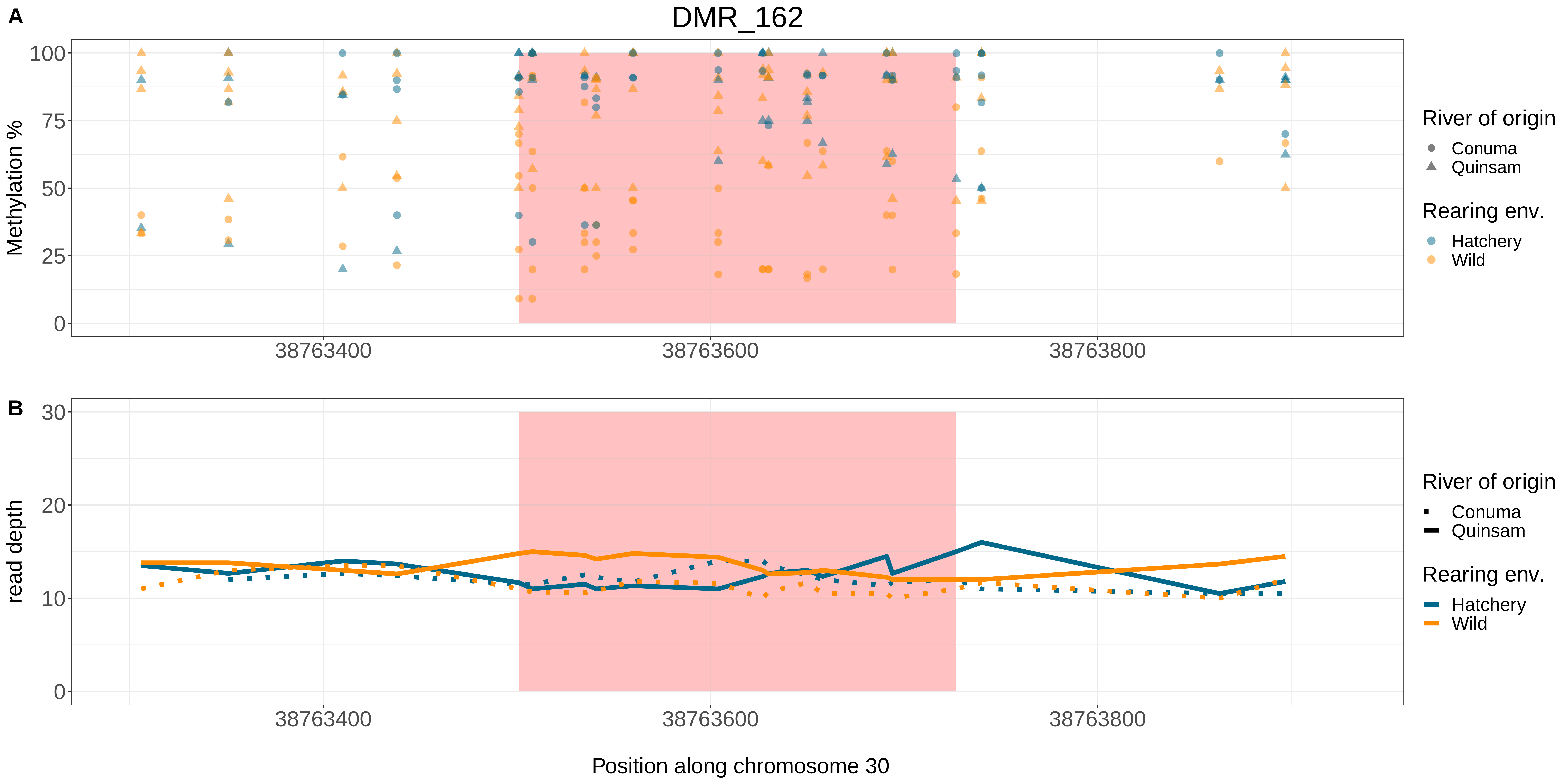
A
DMR_162
Methylation %
100
75
50
25
0
38763400
38763600
38763800
River of origin
Conuma
Quinsam
Rearing env.
Hatchery
Wild
B
read depth
30
20
10
0
38763400
38763600
38763800
River of origin
Conuma
Quinsam
Rearing env.
Hatchery
Wild
Position along chromosome 30

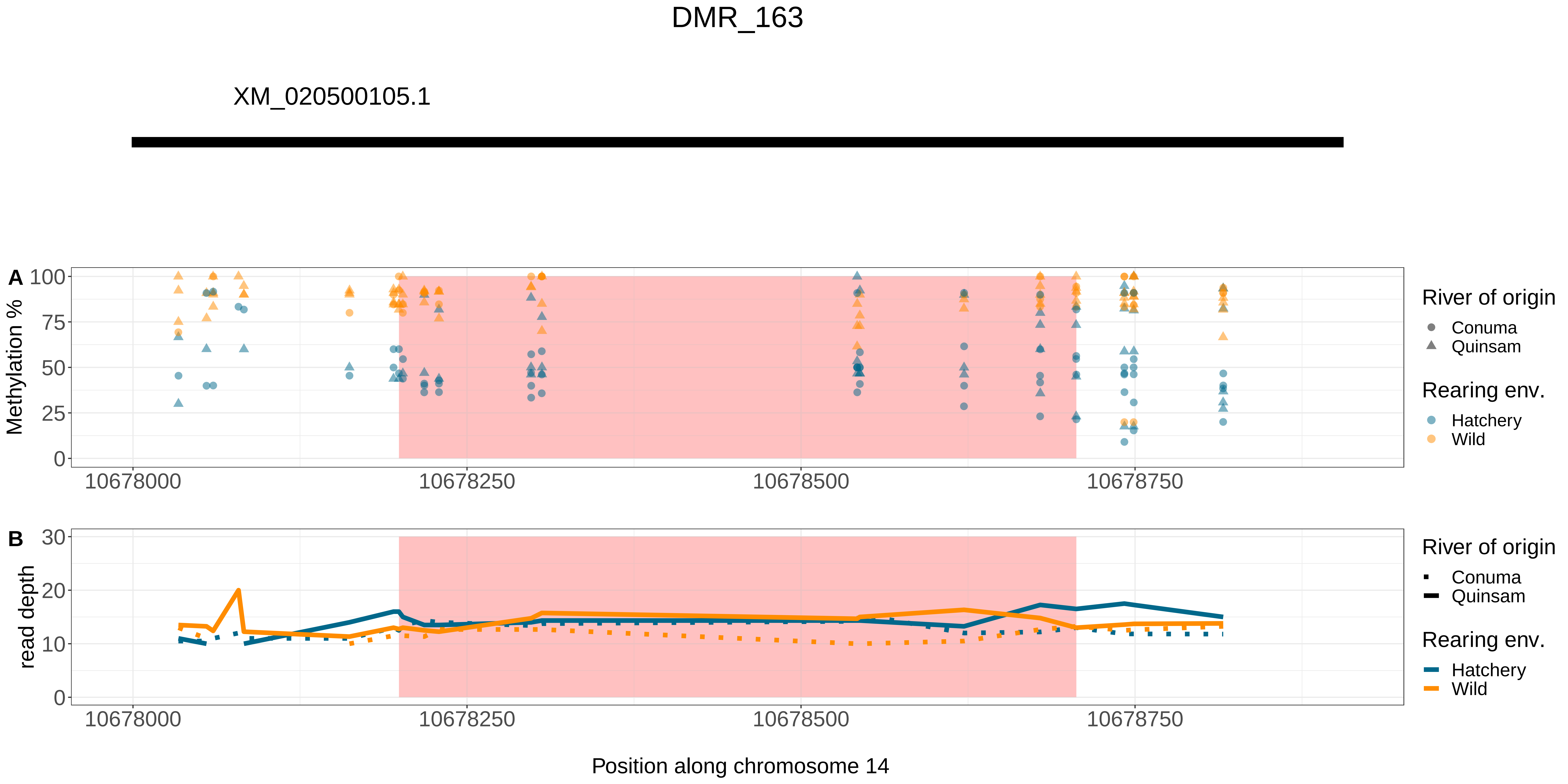
DMR_163
XM_020500105.1
A
Methylation %
100
75
50
25
0
10678000
10678250
10678500
10678750
River of origin
Conuma
Quinsam
Rearing env.
Hatchery
Wild
B
read depth
30
20
10
0
10678000
10678250
10678500
10678750
River of origin
Conuma
Quinsam
Rearing env.
Hatchery
Wild
Position along chromosome 14

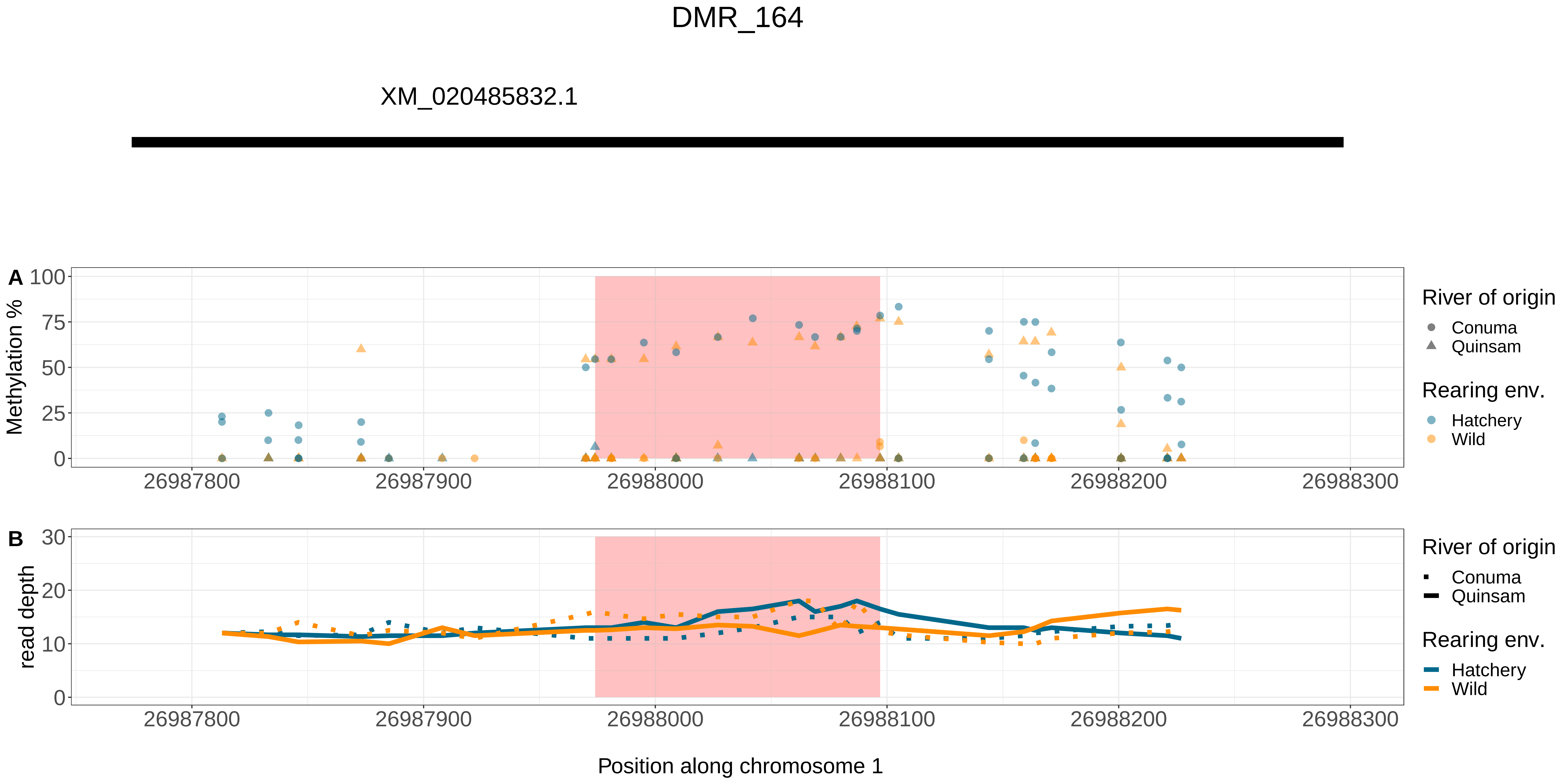
DMR_164
XM_020485832.1
A
Methylation %
100
75
50
25
0
26987800
26987900
26988000
26988100
26988200
26988300
River of origin
Conuma
Quinsam
Rearing env.
Hatchery
Wild
B
read depth
30
20
10
0
26987800
26987900
26988000
26988100
26988200
26988300
River of origin
Conuma
Quinsam
Rearing env.
Hatchery
Wild
Position along chromosome 1

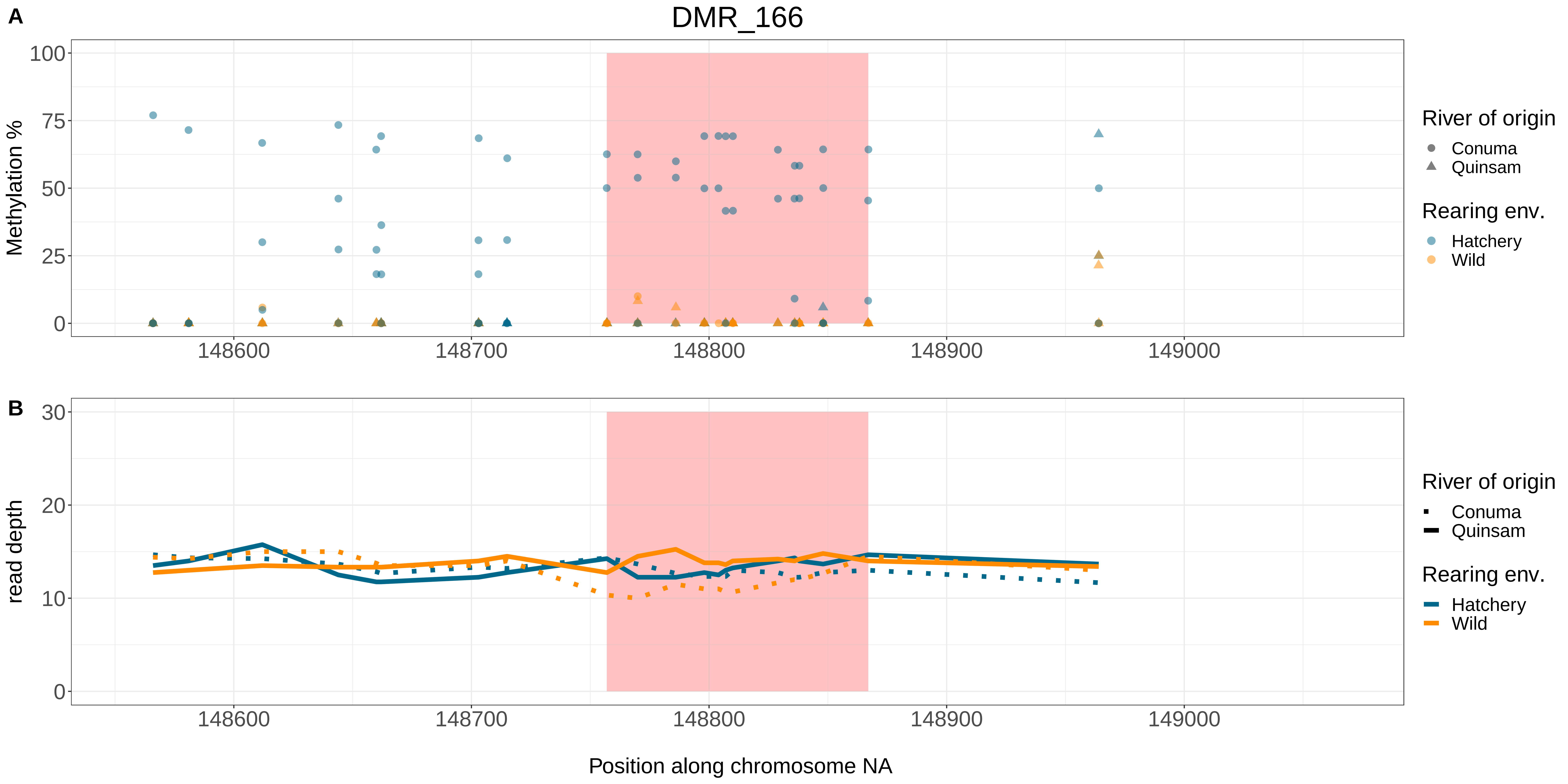
A
DMR_166
Methylation %
100
75
50
25
0
148600
148700
148800
148900
149000
River of origin
Conuma
Quinsam
Rearing env.
Hatchery
Wild
B
read depth
30
20
10
0
Position along chromosome NA

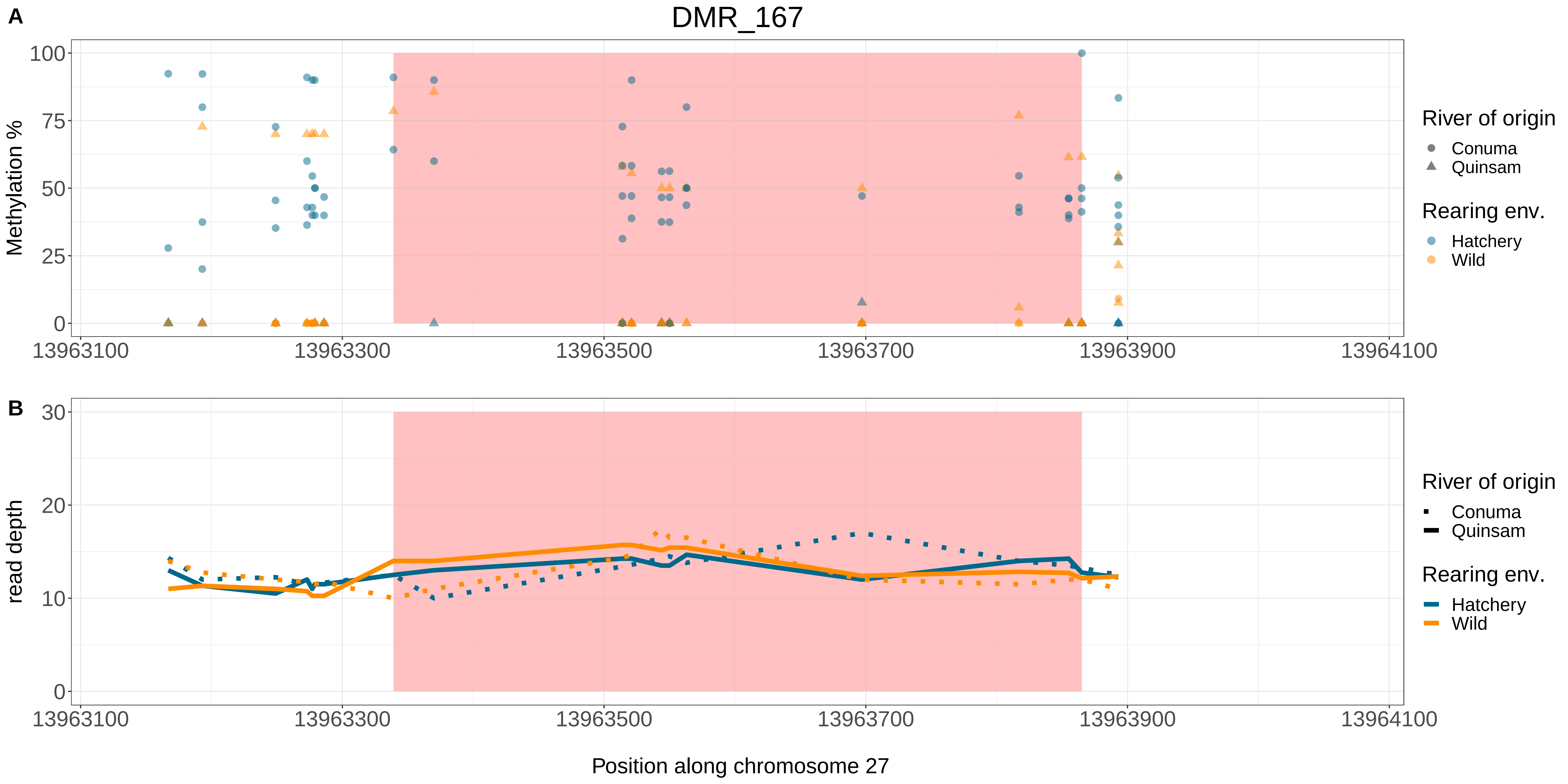

A
DMR_167
Methylation %
100
75
50
25
0
13963100
13963300
13963500
13963700
13963900
13964100
River of origin
Conuma
Quinsam
Rearing env.
Hatchery
Wild
B
read depth
30
20
10
0
13963100
13963300
13963500
13963700
13963900
13964100
River of origin
Conuma
Quinsam
Rearing env.
Hatchery
Wild
Position along chromosome 27

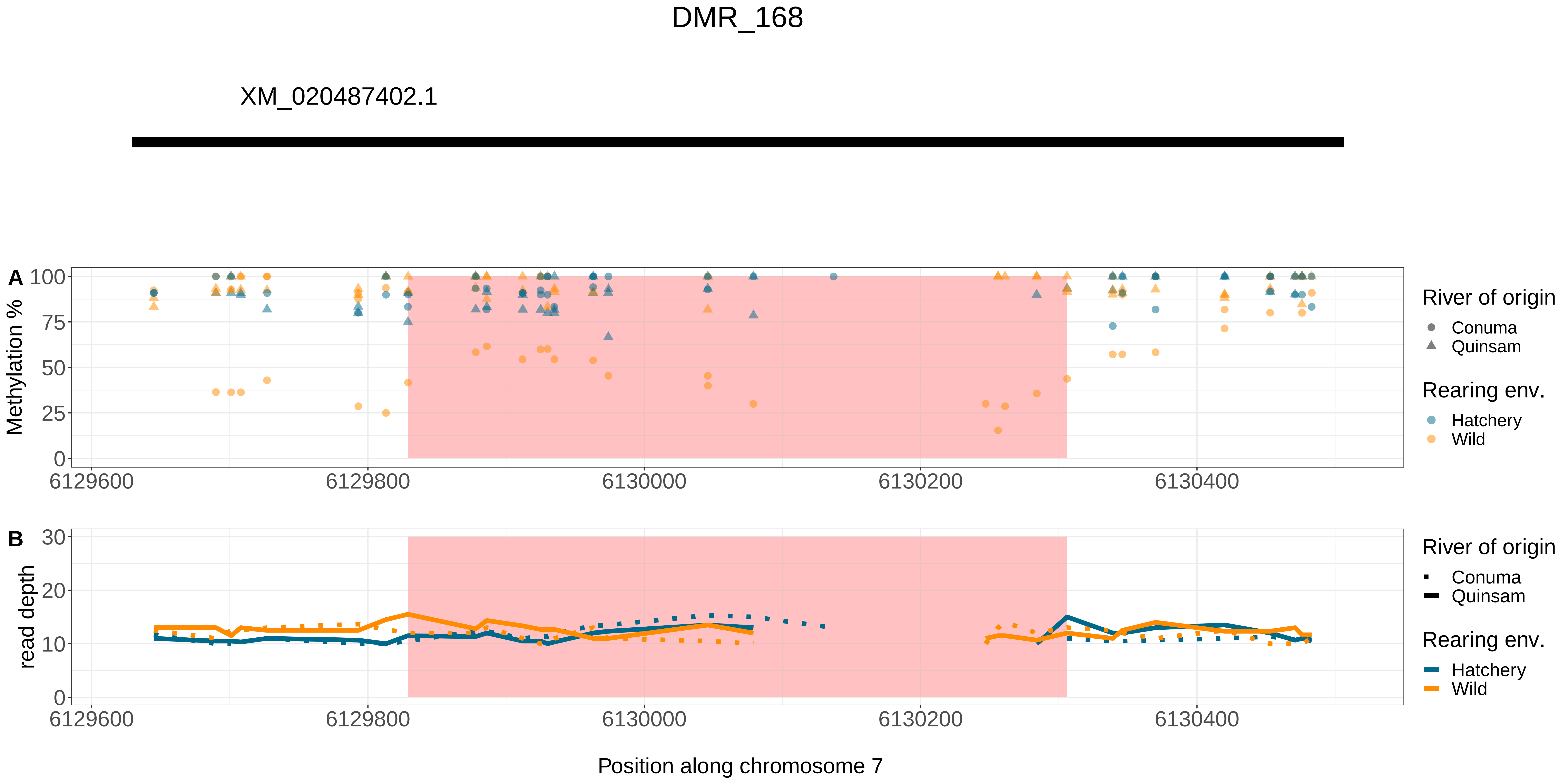
DMR_168
XM_020487402.1
A
Methylation %
100
75
50
25
0
6129600
6129800
6130000
6130200
6130400
River of origin
Conuma
Quinsam
Rearing env.
Hatchery
Wild
B
read depth
30
20
10
0
6129600
6129800
6130000
6130200
6130400
River of origin
Conuma
Quinsam
Rearing env.
Hatchery
Wild
Position along chromosome 7

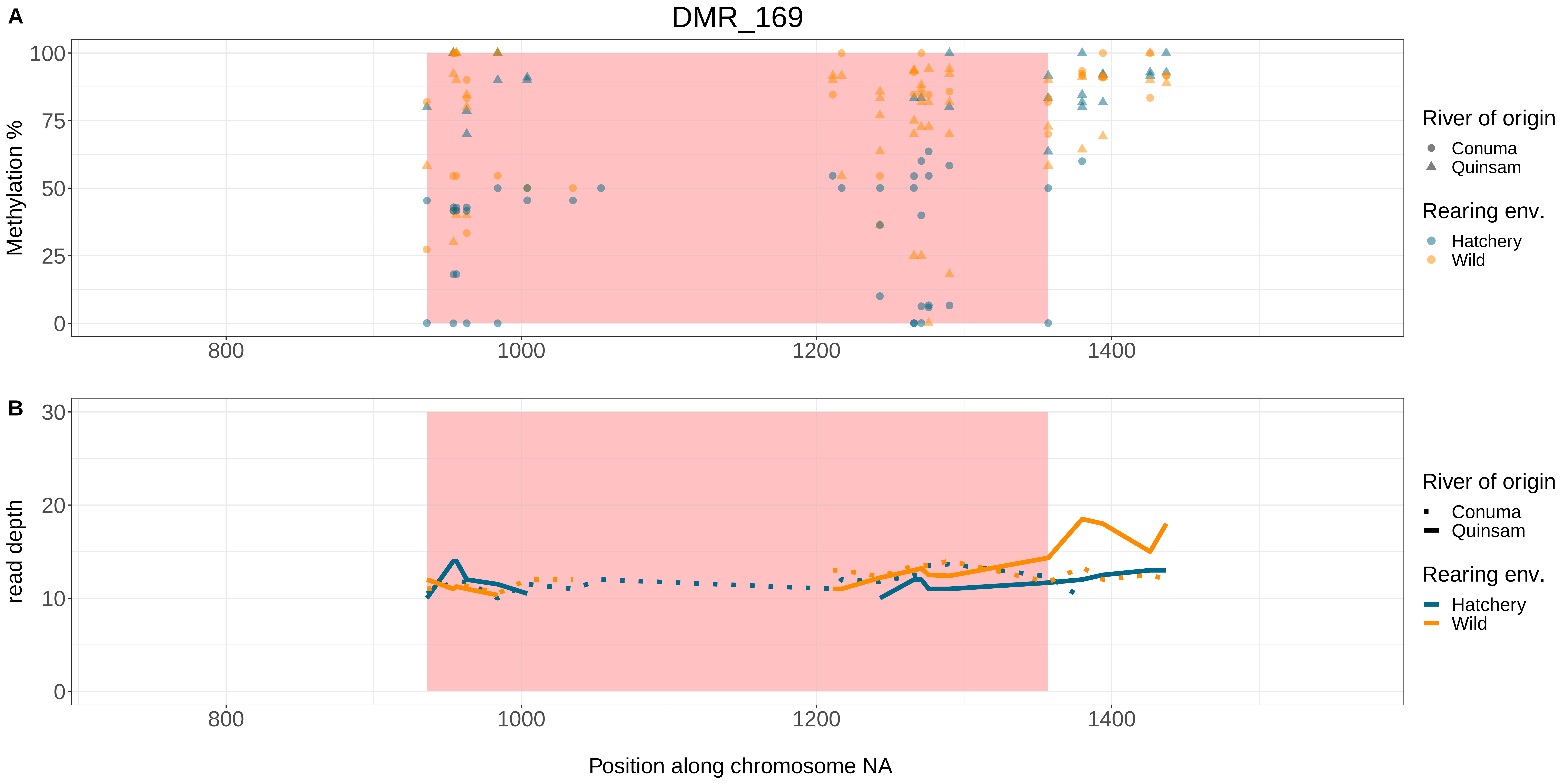

A
DMR_169
Methylation %
100
75
50
25
0
800
1000
1200
1400
River of origin
Conuma
Quinsam
Rearing env.
Hatchery
Wild
B
read depth
30
20
10
0
800
1000
1200
1400
River of origin
Conuma
Quinsam
Rearing env.
Hatchery
Wild
Position along chromosome NA

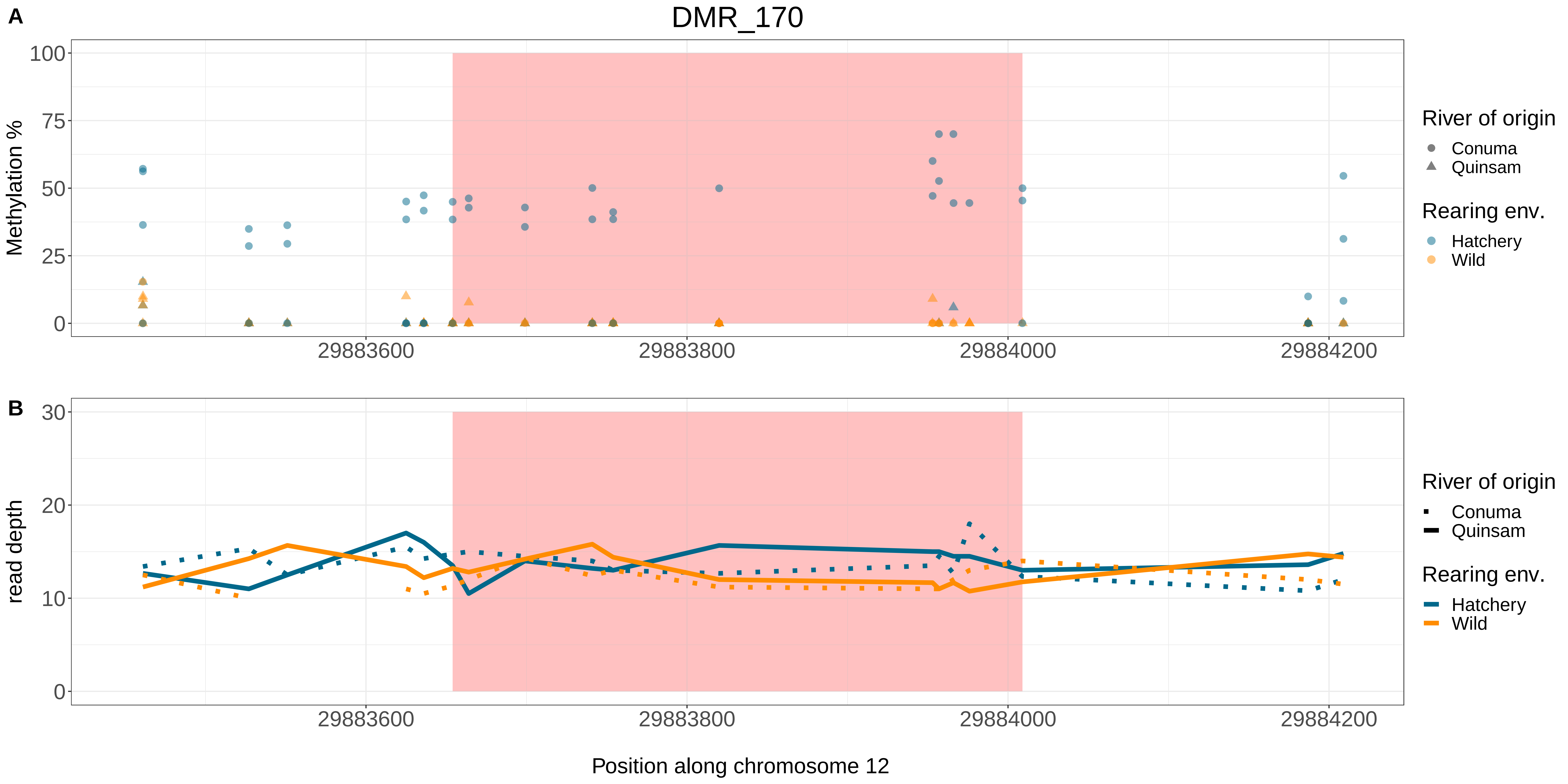

A
DMR_170
Methylation %
100
75
50
25
0
29883600
29883800
29884000
29884200
River of origin
Conuma
Quinsam
Rearing env.
Hatchery
Wild
B
read depth
30
20
10
0
29883600
29883800
29884000
29884200
River of origin
Conuma
Quinsam
Rearing env.
Hatchery
Wild
Position along chromosome 12

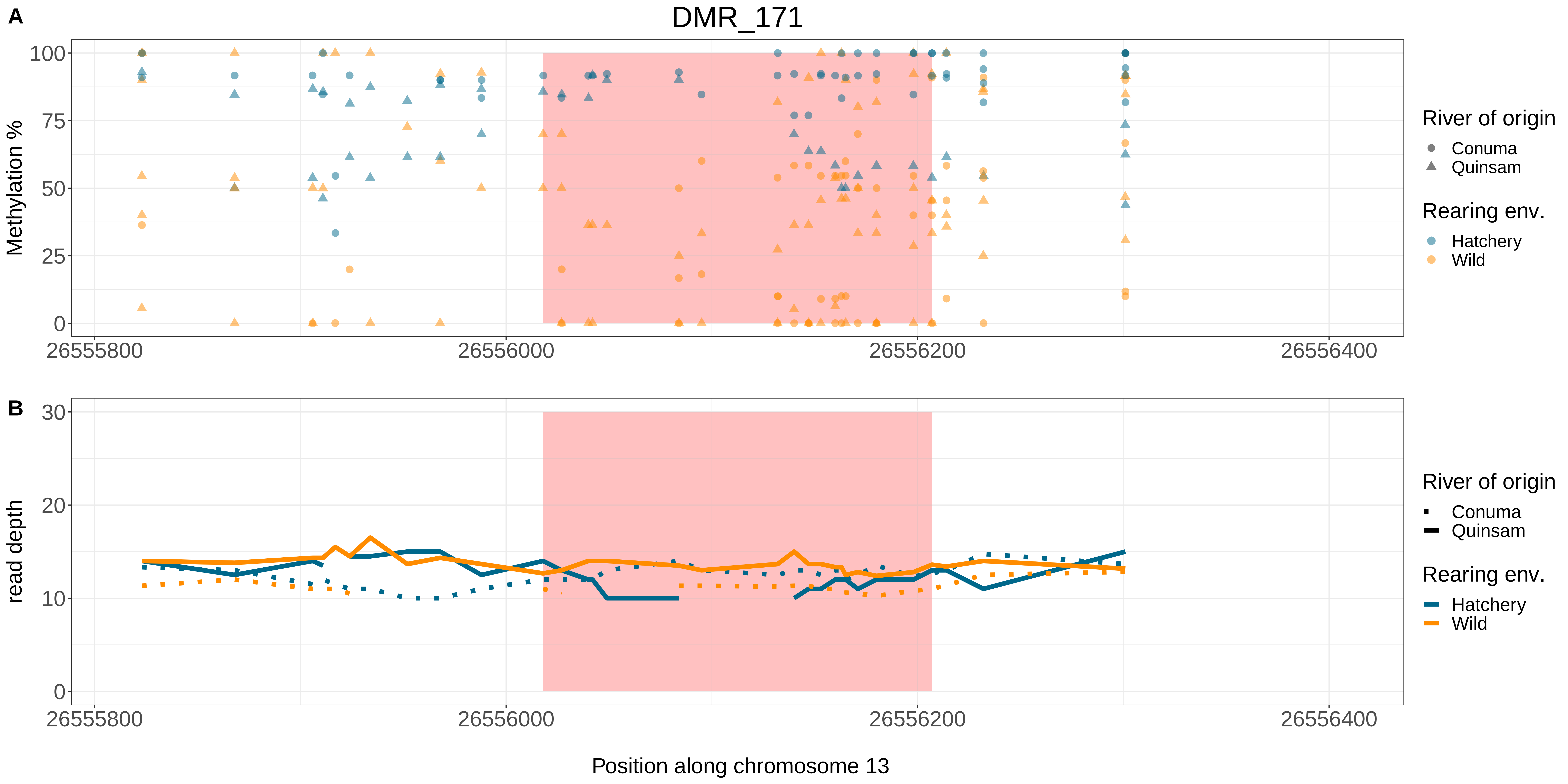
A
DMR_171
Methylation %
100
75
50
25
0
26555800
26556000
26556200
26556400
River of origin
Conuma
Quinsam
Rearing env.
Hatchery
Wild
B
read depth
30
20
10
0
26555800
26556000
26556200
26556400
River of origin
Conuma
Quinsam
Rearing env.
Hatchery
Wild
Position along chromosome 13

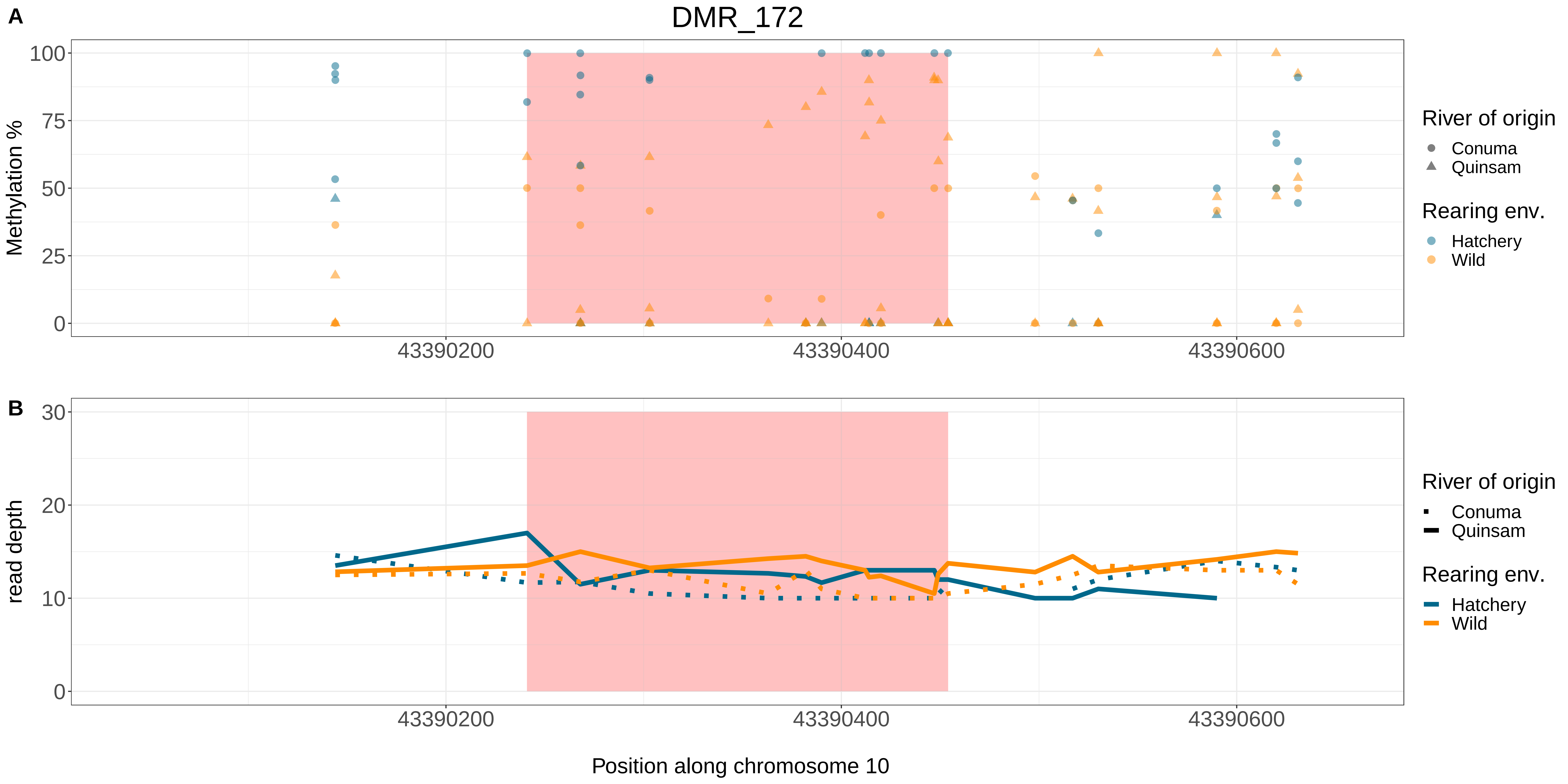

A
DMR_172
Methylation %
100
75
50
25
0
43390200
43390400
43390600
River of origin
Conuma
Quinsam
Rearing env.
Hatchery
Wild
B
read depth
30
20
10
0
43390200
43390400
43390600
River of origin
Conuma
Quinsam
Rearing env.
Hatchery
Wild
Position along chromosome 10

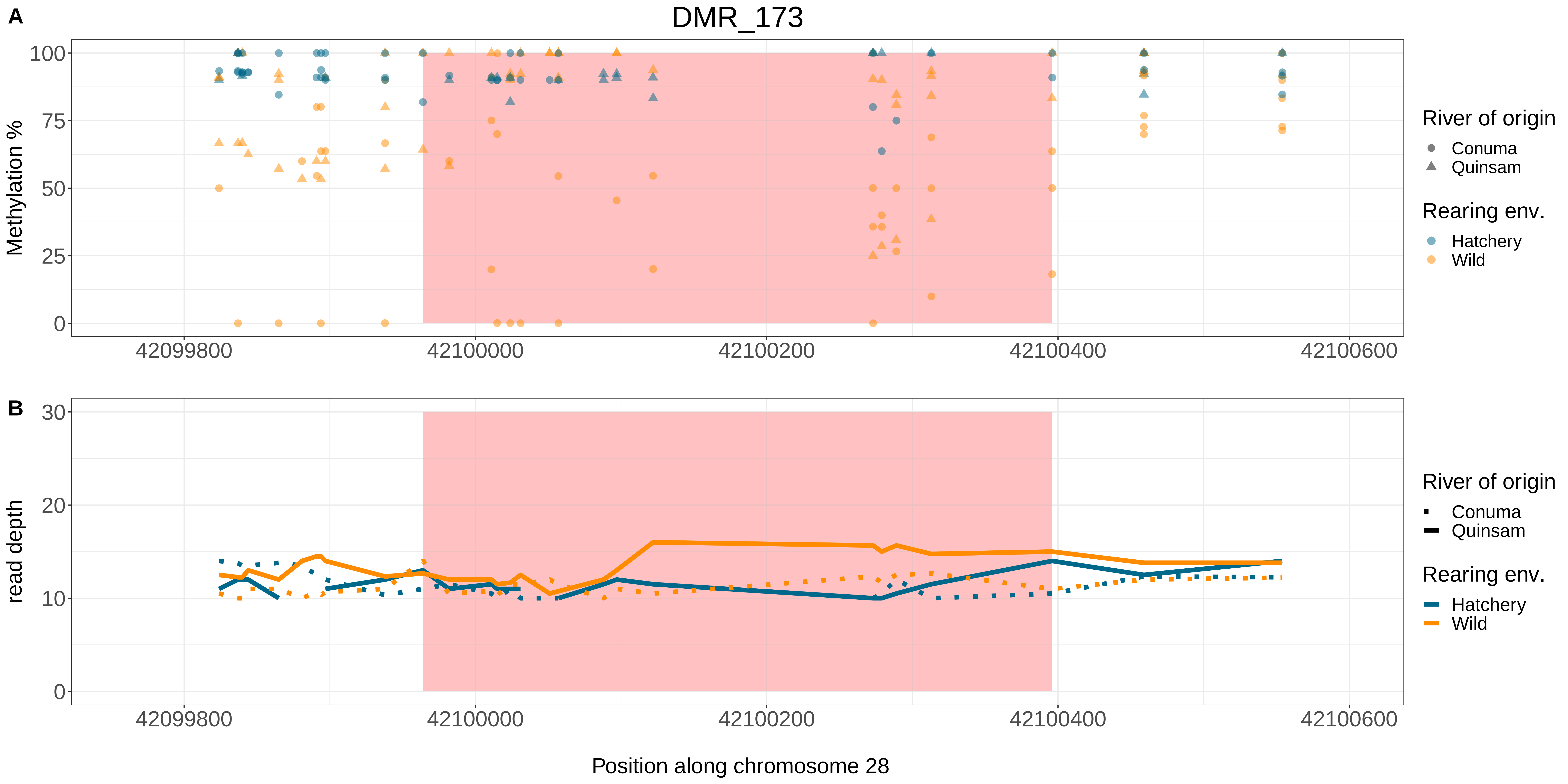

A
DMR_173
Methylation %
100
75
50
25
0
42099800
42100000
42100200
42100400
42100600
River of origin
Conuma
Quinsam
Rearing env.
Hatchery
Wild
B
read depth
30
20
10
0
42099800
42100000
42100200
42100400
42100600
River of origin
Conuma
Quinsam
Rearing env.
Hatchery
Wild
Position along chromosome 28

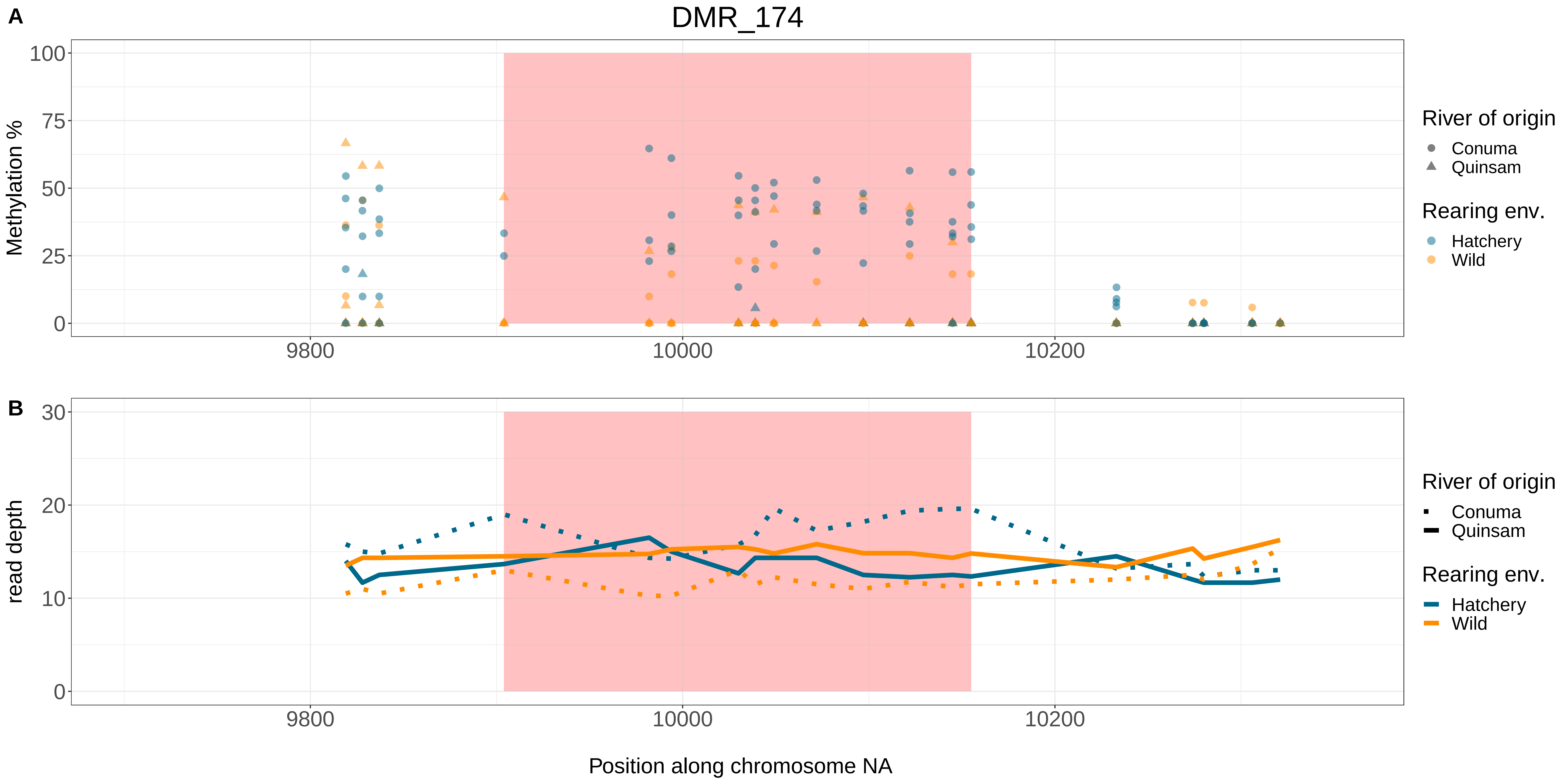
A
DMR_174
Methylation %
100
75
50
25
0
9800
10000
10200
River of origin
Conuma
Quinsam
Rearing env.
Hatchery
Wild
B
read depth
30
20
10
0
9800
10000
10200
River of origin
Conuma
Quinsam
Rearing env.
Hatchery
Wild
Position along chromosome NA

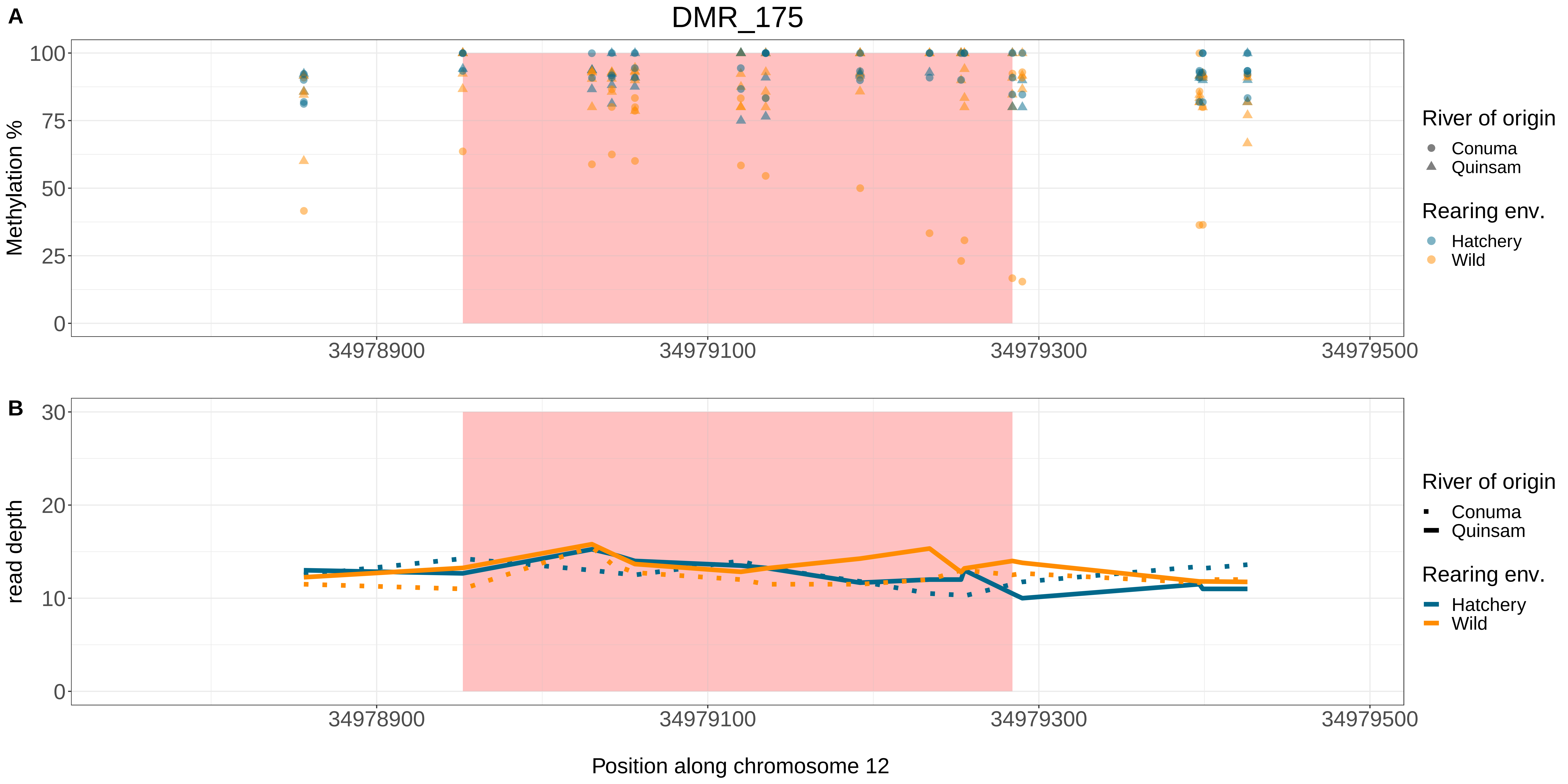
A
DMR_175
Methylation %
100
75
50
25
0
34978900
34979100
34979300
34979500
River of origin
Conuma
Quinsam
Rearing env.
Hatchery
Wild
B
read depth
30
20
10
0
34978900
34979100
34979300
34979500
River of origin
Conuma
Quinsam
Rearing env.
Hatchery
Wild
Position along chromosome 12

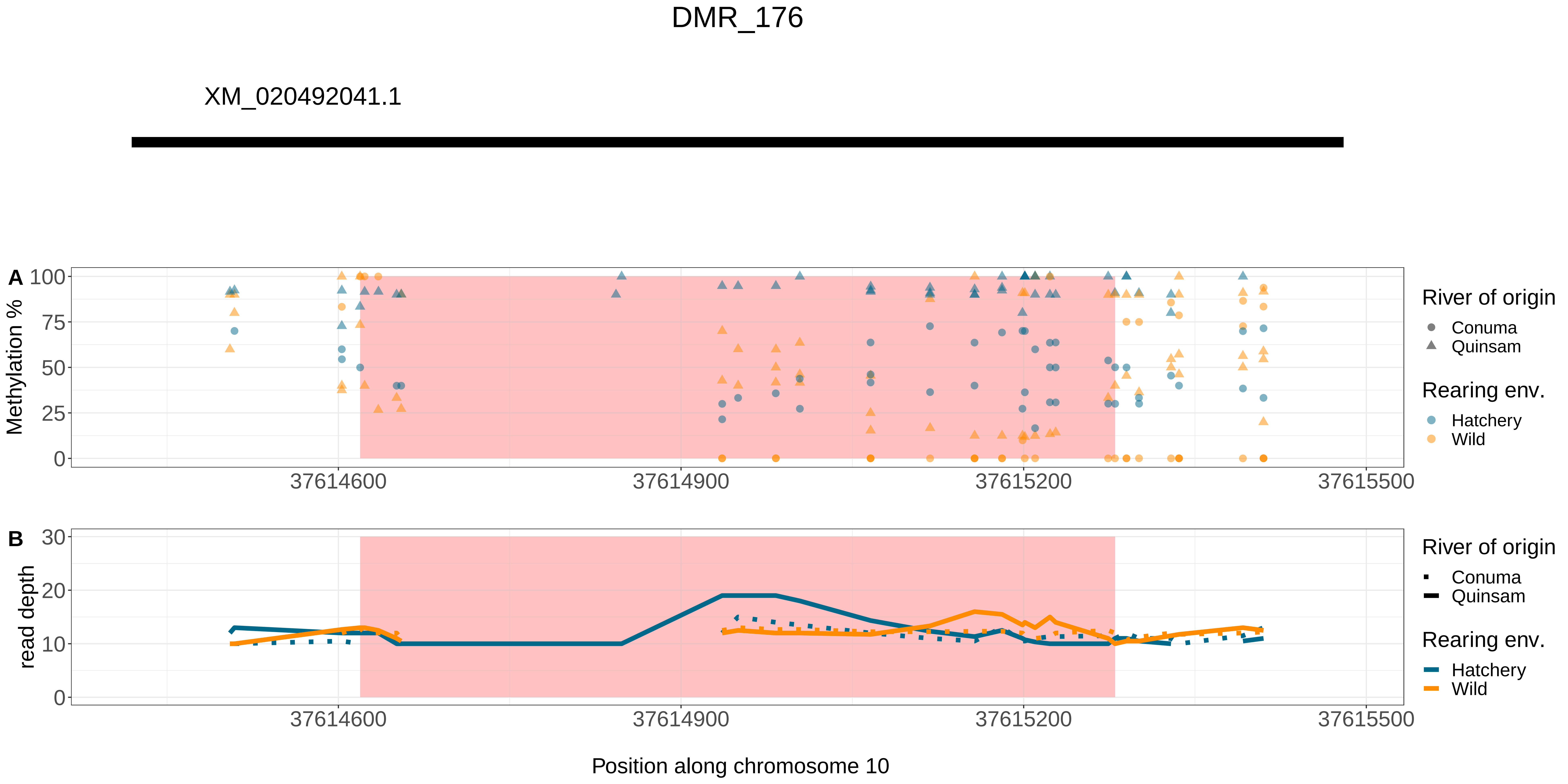

DMR_176
XM_020492041.1
A
Methylation %
100
75
50
25
0
37614600
37614900
37615200
37615500
River of origin
Conuma
Quinsam
Rearing env.
Hatchery
Wild
B
read depth
30
20
10
0
37614600
37614900
37615200
37615500
River of origin
Conuma
Quinsam
Rearing env.
Hatchery
Wild
Position along chromosome 10

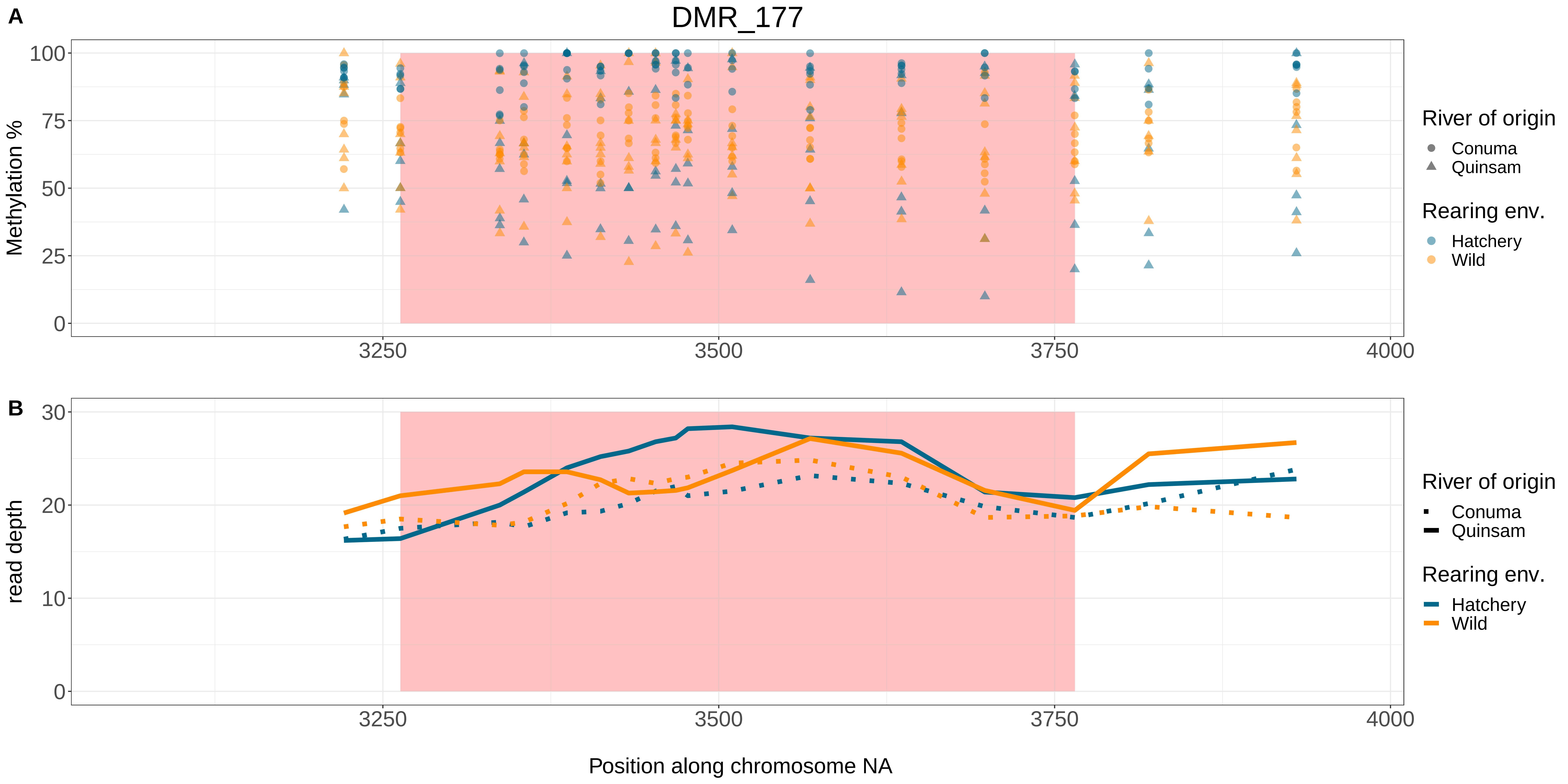

A
DMR_177
Methylation %
100
75
50
25
0
3250
3500
3750
4000
River of origin
Conuma
Quinsam
Rearing env.
Hatchery
Wild
B
read depth
30
20
10
0
3250
3500
3750
4000
River of origin
Conuma
Quinsam
Rearing env.
Hatchery
Wild
Position along chromosome NA

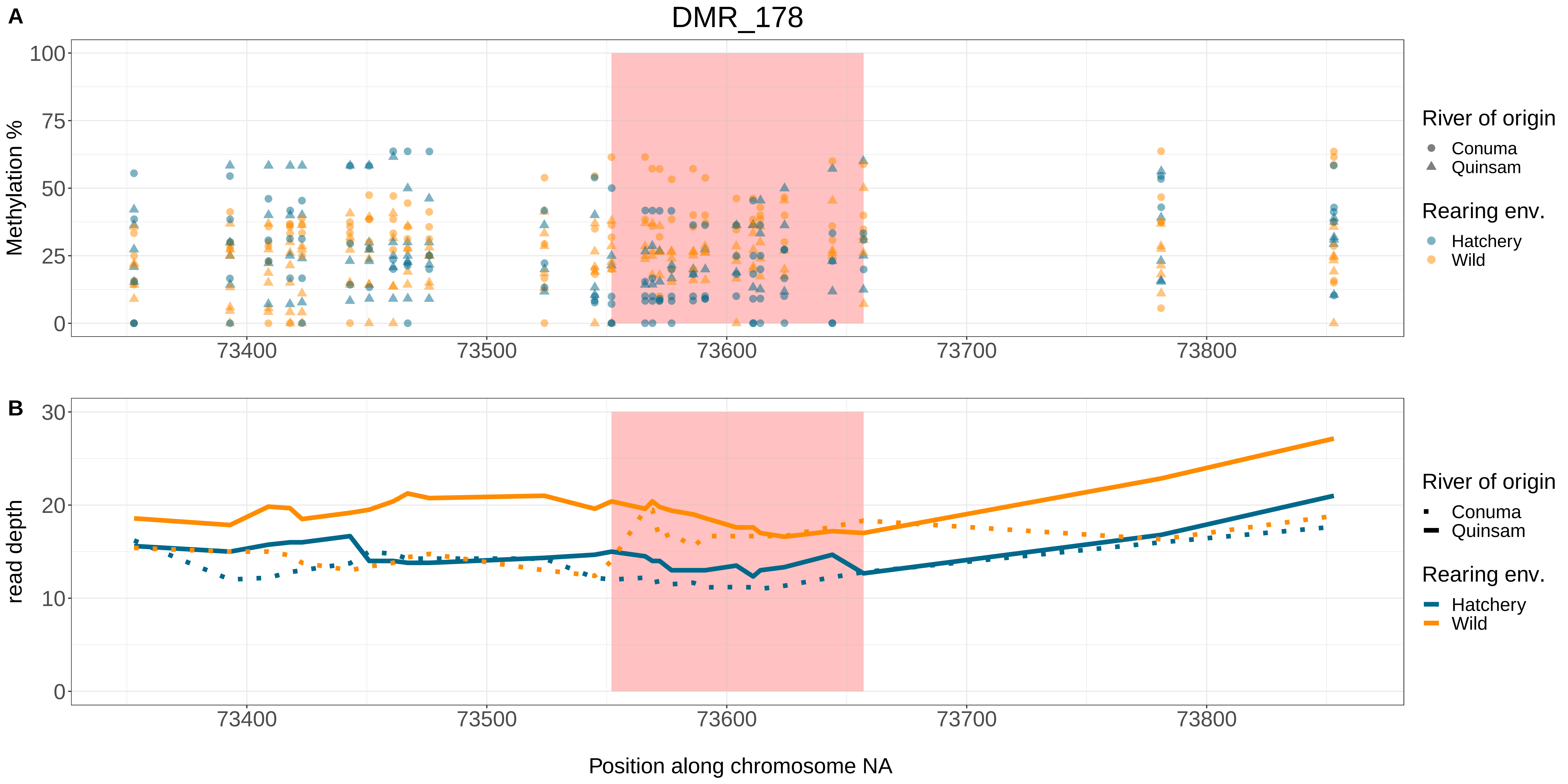
A
DMR_178
Methylation %
100
75
50
25
0
73400
73500
73600
73700
73800
River of origin
Conuma
Quinsam
Rearing env.
Hatchery
Wild
B
read depth
30
20
10
0
73400
73500
73600
73700
73800
River of origin
Conuma
Quinsam
Rearing env.
Hatchery
Wild
Position along chromosome NA

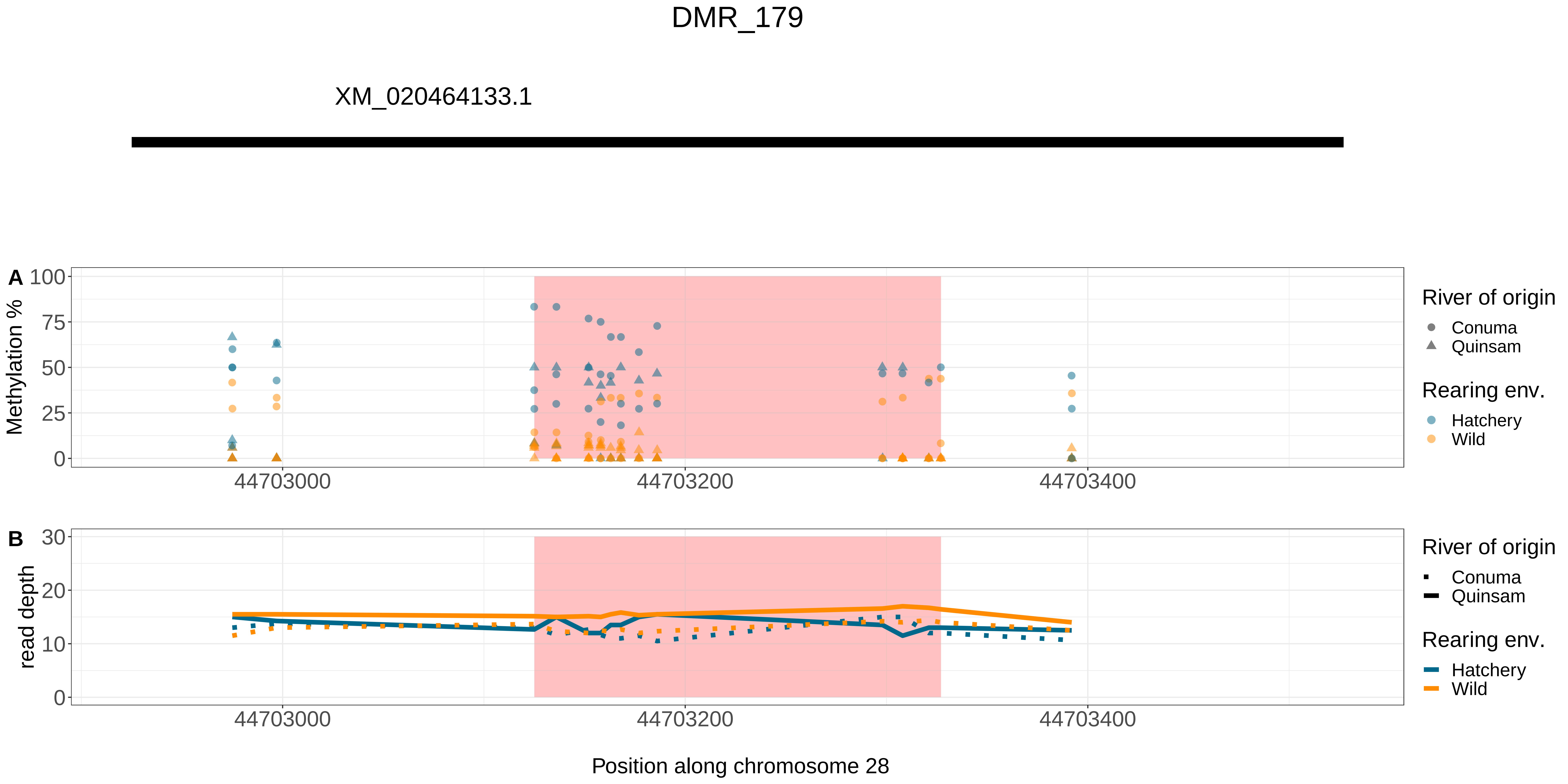

DMR_179
XM_020464133.1
A
Methylation %
100
75
50
25
0
44703000
44703200
44703400
River of origin
Conuma
Quinsam
Rearing env.
Hatchery
Wild
B
read depth
30
20
10
0
44703000
44703200
44703400
River of origin
Conuma
Quinsam
Rearing env.
Hatchery
Wild
Position along chromosome 28

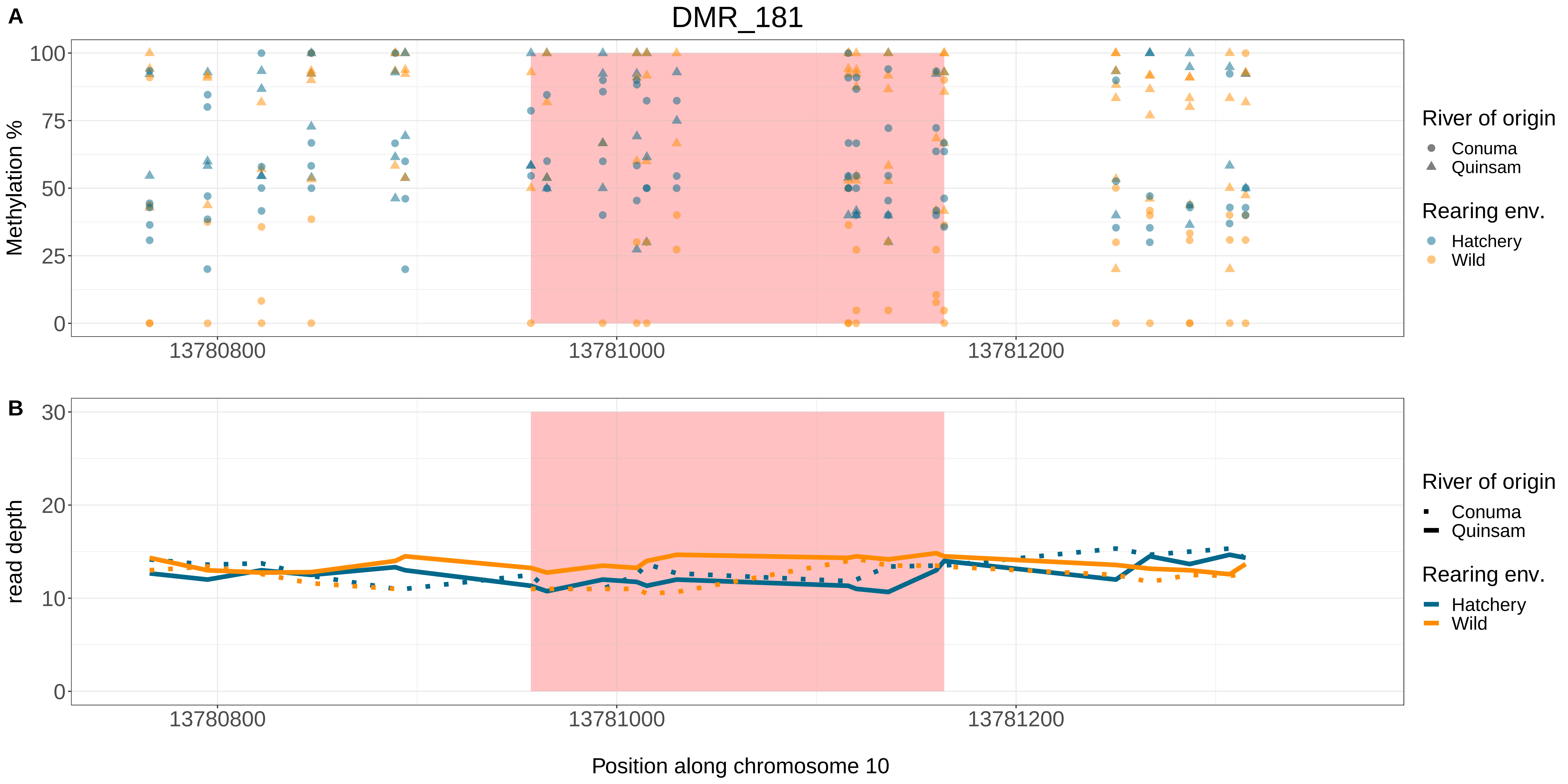

A
DMR_181
Methylation %
100
75
50
25
0
13780800
13781000
13781200
River of origin
Conuma
Quinsam
Rearing env.
Hatchery
Wild
B
read depth
30
20
10
0
13780800
13781000
13781200
River of origin
Conuma
Quinsam
Rearing env.
Hatchery
Wild
Position along chromosome 10

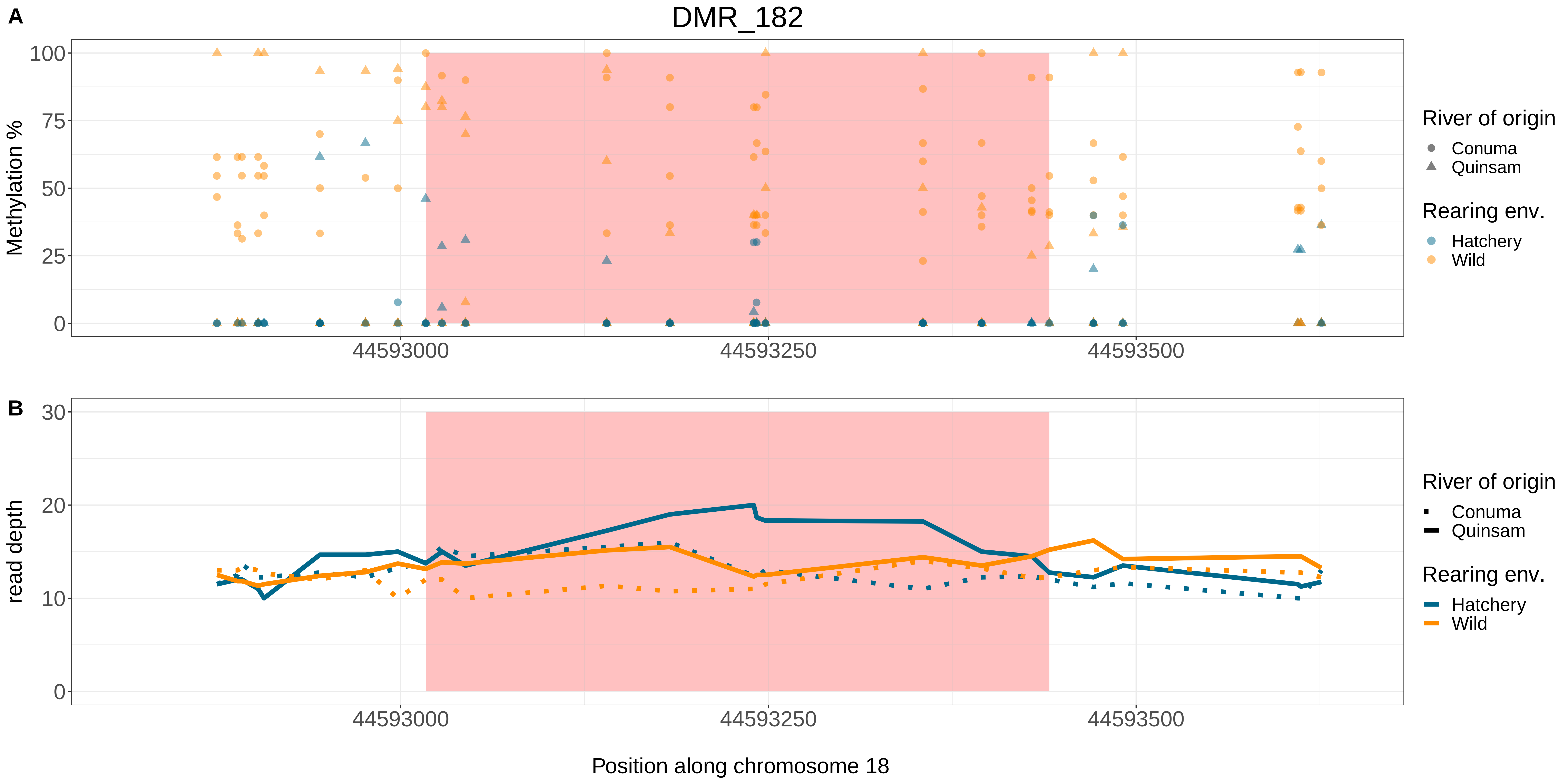

A
DMR_182
Methylation %
100
75
50
25
0
44593000
44593250
44593500
River of origin
Conuma
Quinsam
Rearing env.
Hatchery
Wild
B
read depth
30
20
10
0
44593000
44593250
44593500
River of origin
Conuma
Quinsam
Rearing env.
Hatchery
Wild
Position along chromosome 18

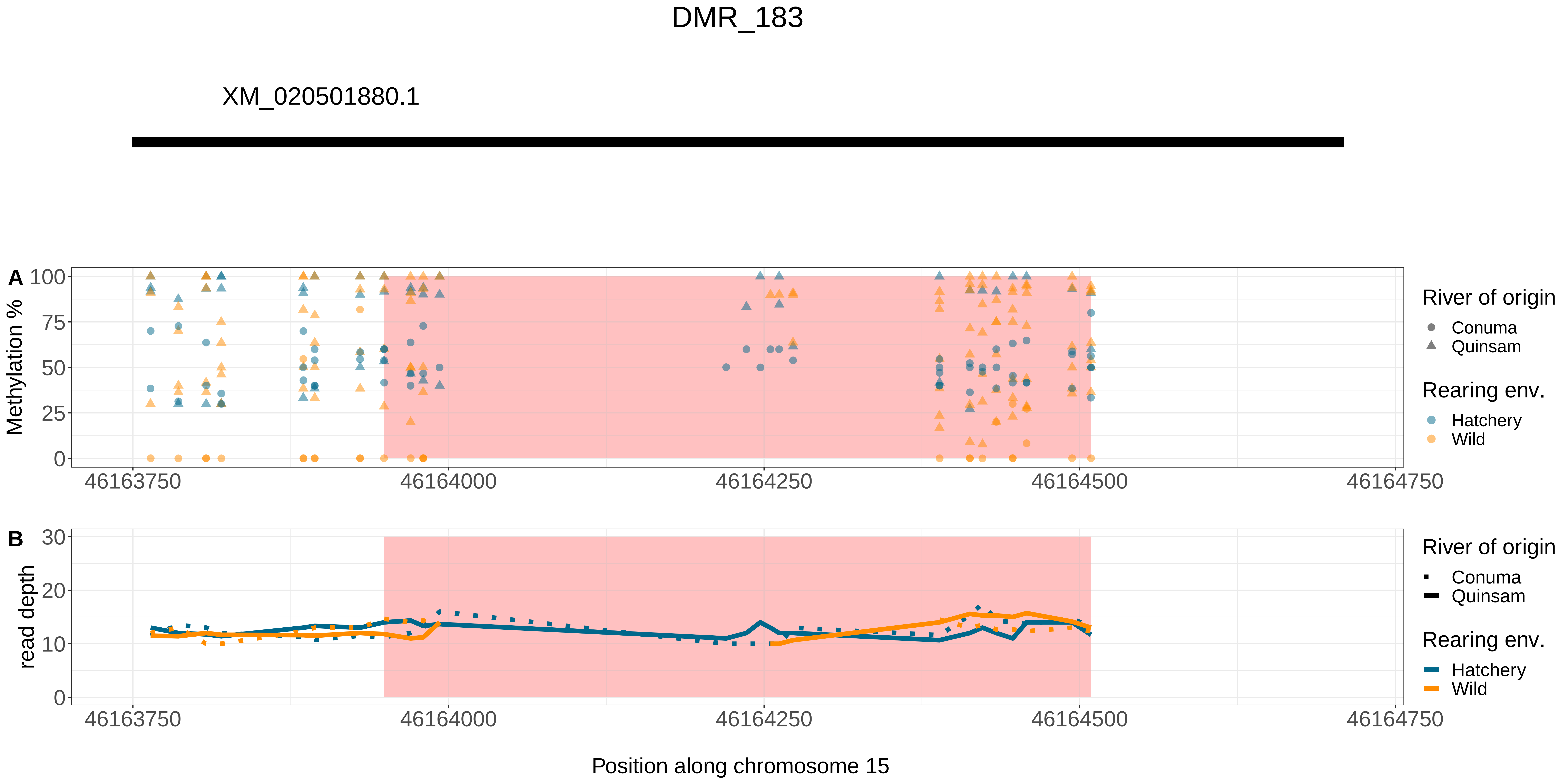

DMR_183
XM_020501880.1
A
Methylation %
100
75
50
25
0
46163750
46164000
46164250
46164500
46164750
River of origin
Conuma
Quinsam
Rearing env.
Hatchery
Wild
B
read depth
30
20
10
0
46163750
46164000
46164250
46164500
46164750
River of origin
Conuma
Quinsam
Rearing env.
Hatchery
Wild
Position along chromosome 15

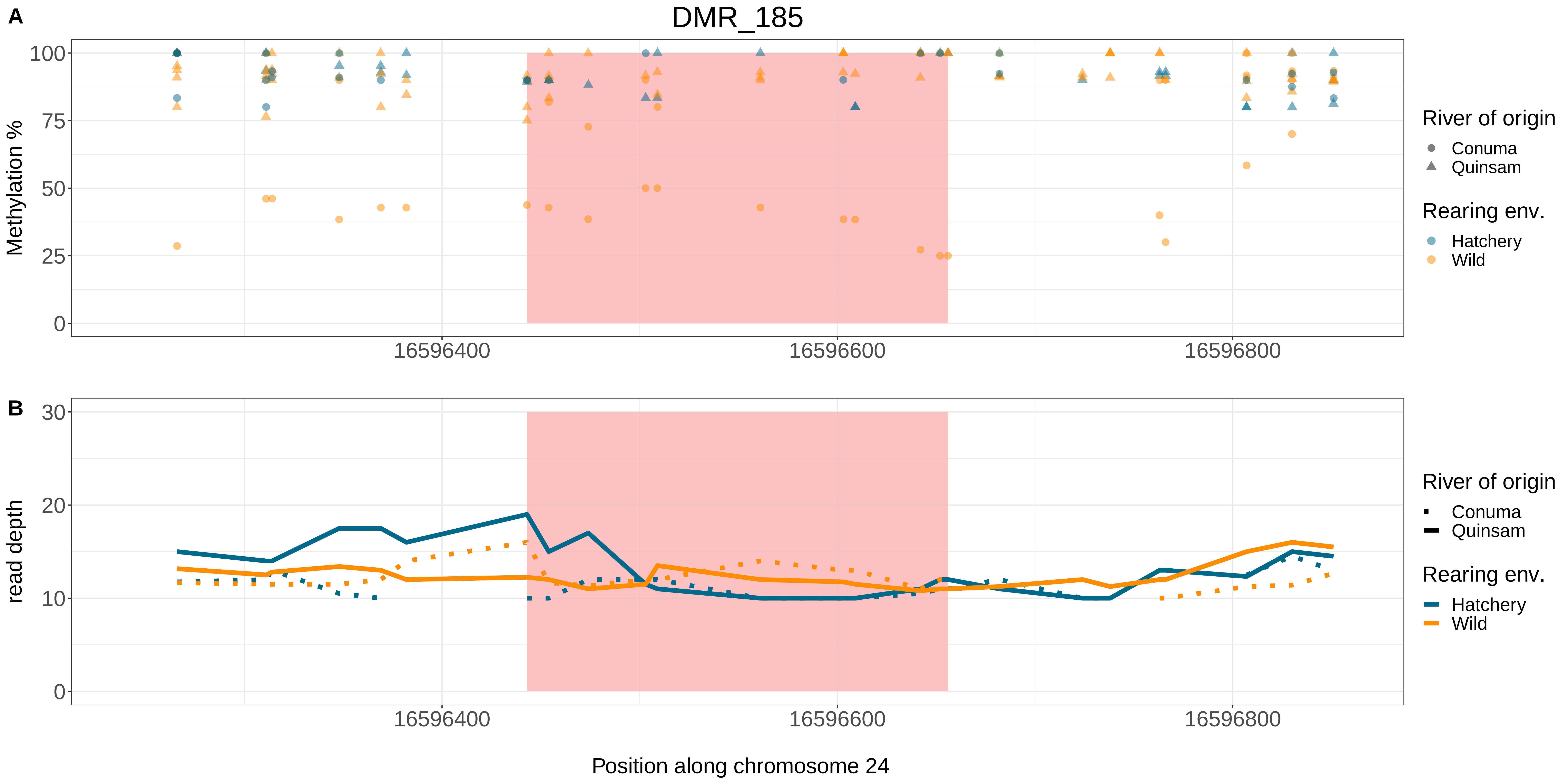

A
DMR_185
Methylation %
100
75
50
25
0
16596400
16596600
16596800
River of origin
Conuma
Quinsam
Rearing env.
Hatchery
Wild
B
read depth
30
20
10
0
16596400
16596600
16596800
River of origin
Conuma
Quinsam
Rearing env.
Hatchery
Wild
Position along chromosome 24

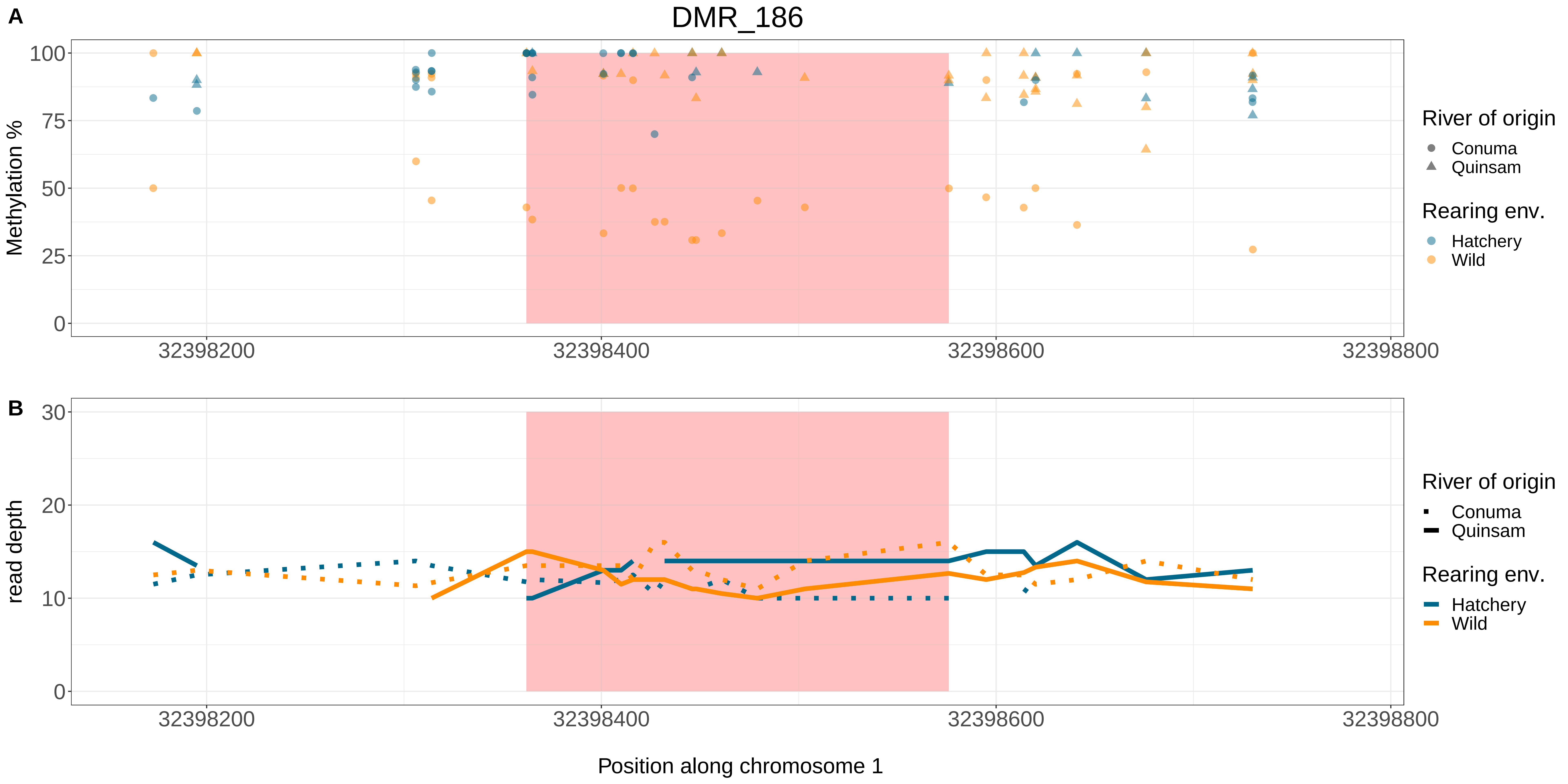

A
DMR_186
Methylation %
100
75
50
25
0
32398200
32398400
32398600
32398800
River of origin
Conuma
Quinsam
Rearing env.
Hatchery
Wild
B
read depth
30
20
10
0
32398200
32398400
32398600
32398800
River of origin
Conuma
Quinsam
Rearing env.
Hatchery
Wild
Position along chromosome 1

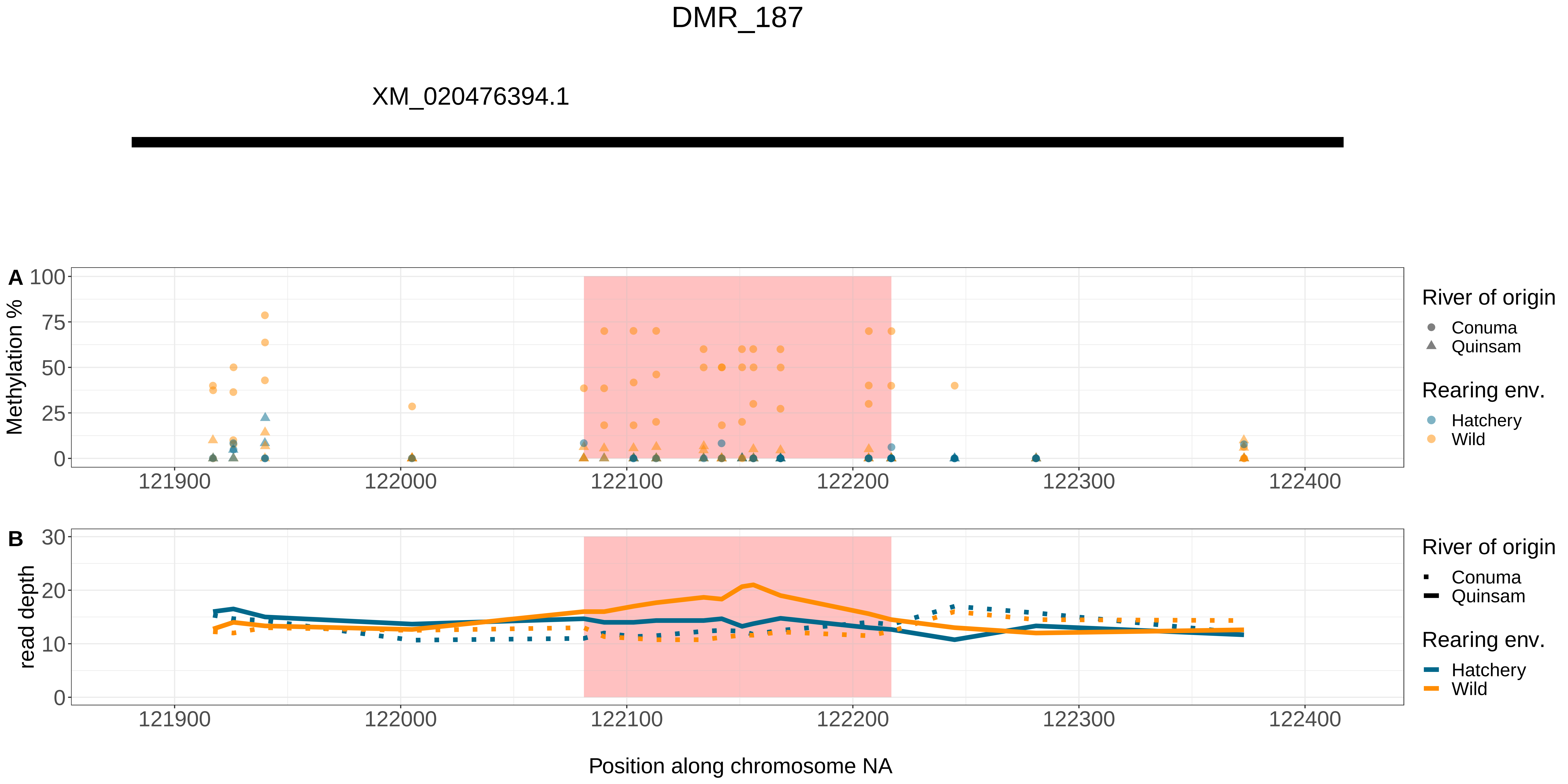
DMR_187
XM_020476394.1
A
Methylation %
100
75
50
25
0
121900
122000
122100
122200
122300
122400
River of origin
Conuma
Quinsam
Rearing env.
Hatchery
Wild
B
read depth
30
20
10
0
121900
122000
122100
122200
122300
122400
River of origin
Conuma
Quinsam
Rearing env.
Hatchery
Wild
Position along chromosome NA

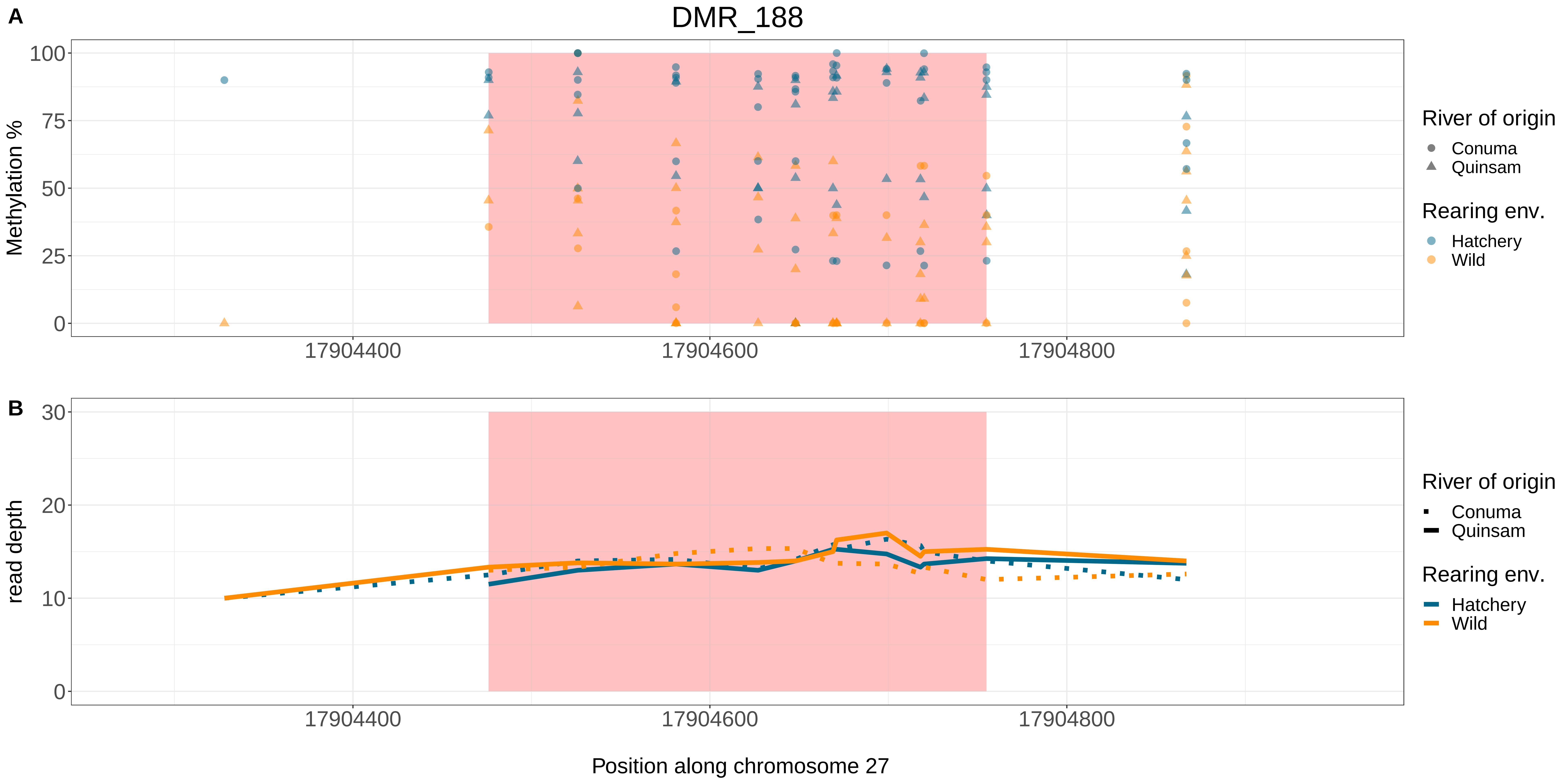
A
DMR_188
Methylation %
100
75
50
25
0
17904400
17904600
17904800
River of origin
Conuma
Quinsam
Rearing env.
Hatchery
Wild
B
read depth
30
20
10
0
17904400
17904600
17904800
River of origin
Conuma
Quinsam
Rearing env.
Hatchery
Wild
Position along chromosome 27

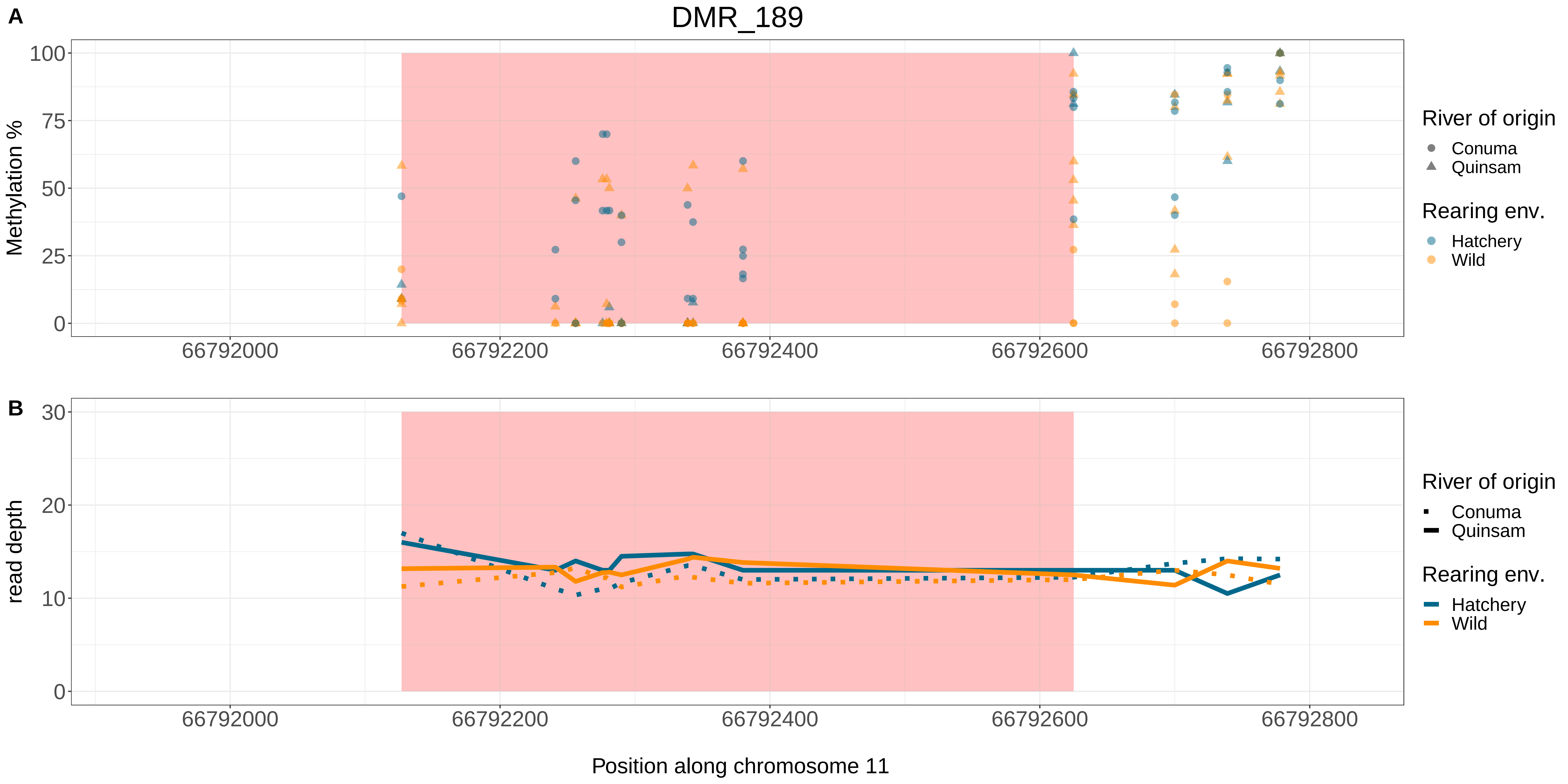
A
DMR_189
Methylation %
100
75
50
25
0
66792000
66792200
66792400
66792600
66792800
River of origin
Conuma
Quinsam
Rearing env.
Hatchery
Wild
B
read depth
30
20
10
0
66792000
66792200
66792400
66792600
66792800
River of origin
Conuma
Quinsam
Rearing env.
Hatchery
Wild
Position along chromosome 11

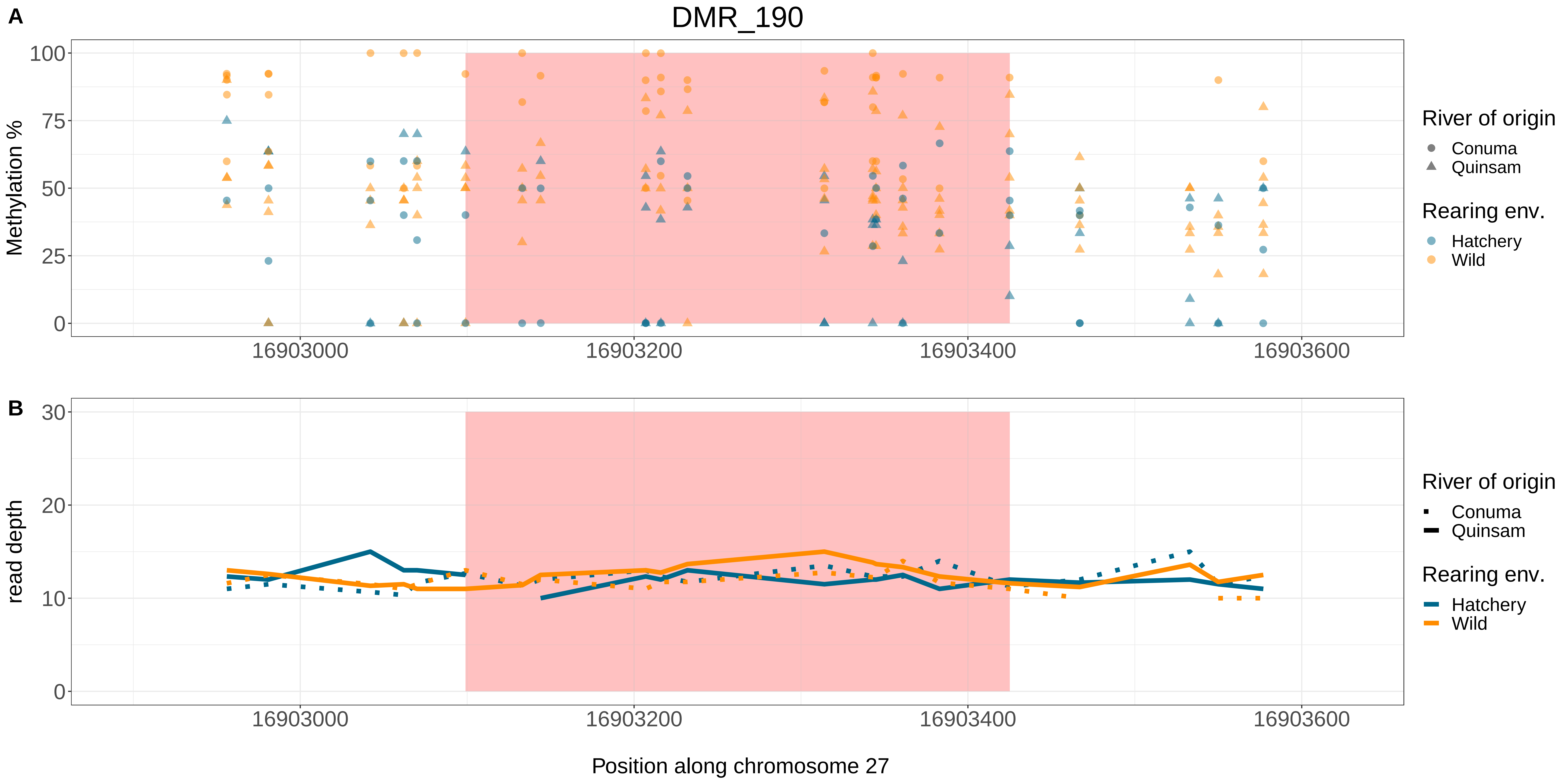
A
DMR_190
Methylation %
100
75
50
25
0
16903000
16903200
16903400
16903600
River of origin
Conuma
Quinsam
Rearing env.
Hatchery
Wild
B
read depth
30
20
10
0
16903000
16903200
16903400
16903600
River of origin
Conuma
Quinsam
Rearing env.
Hatchery
Wild
Position along chromosome 27

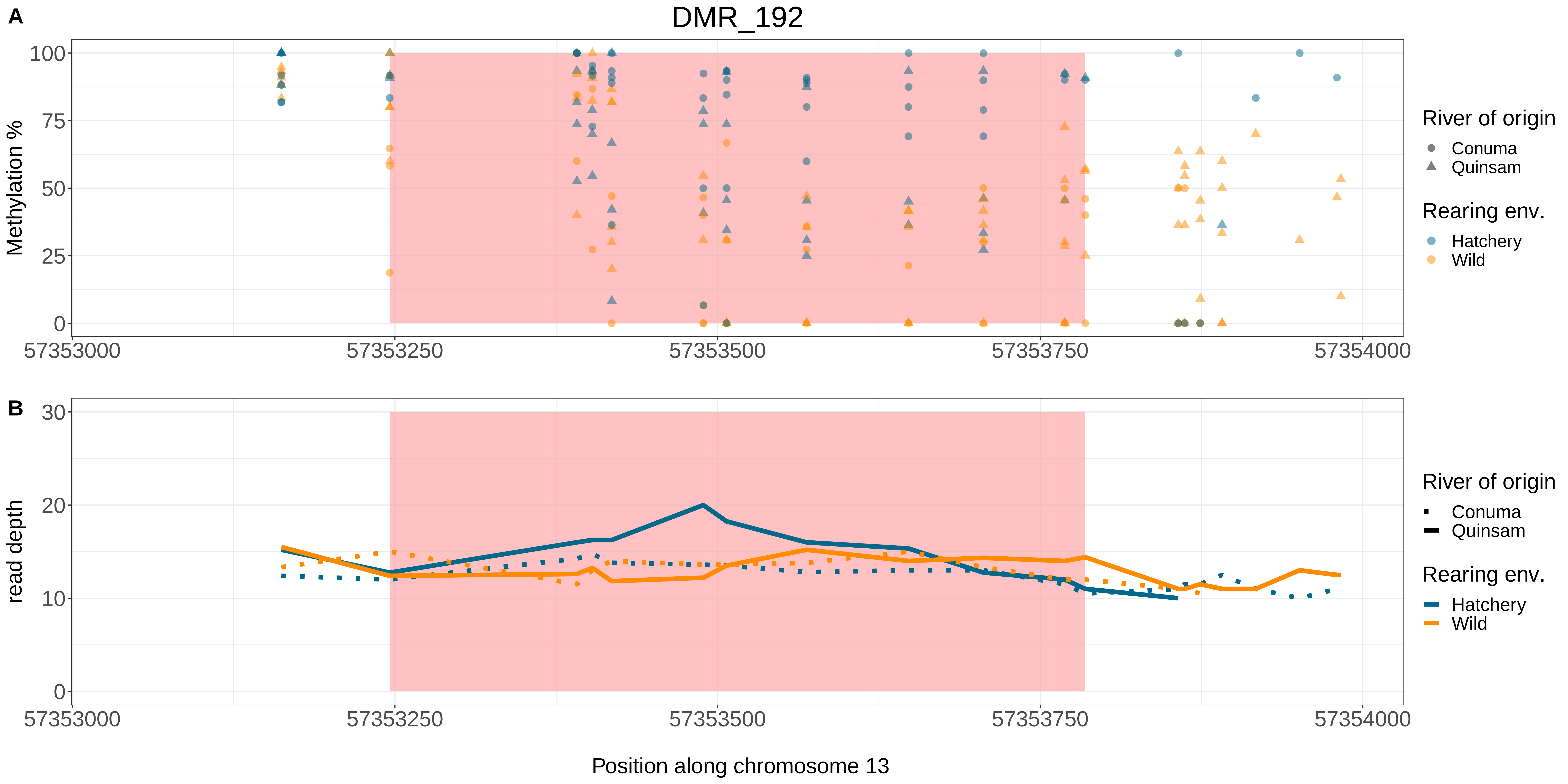
A
DMR_192
Methylation %
100
75
50
25
0
57353000
57353250
57353500
57353750
57354000
River of origin
Conuma
Quinsam
Rearing env.
Hatchery
Wild
B
read depth
30
20
10
0
Position along chromosome 13

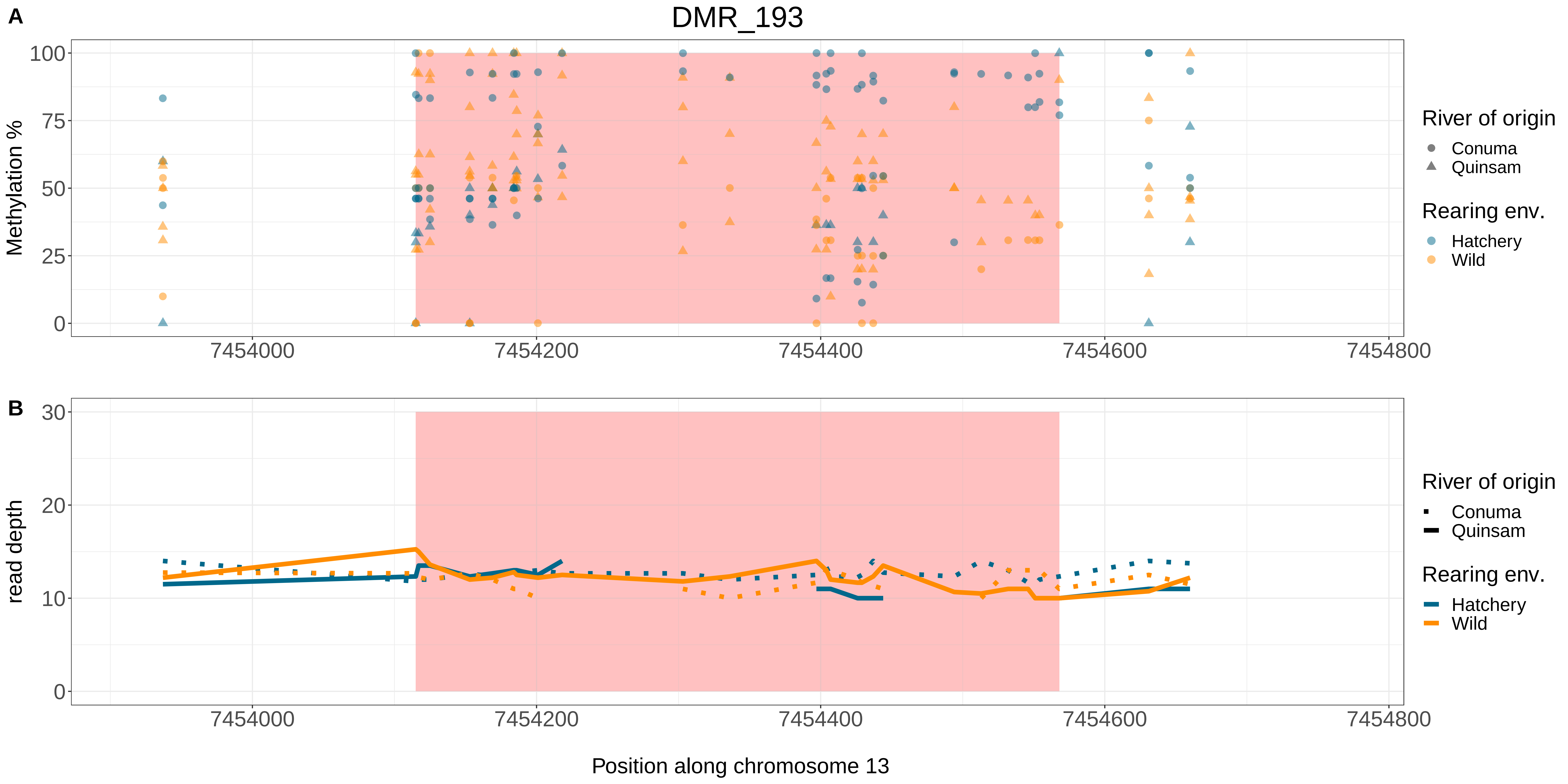

A
DMR_193
Methylation %
100
75
50
25
0
7454000
7454200
7454400
7454600
7454800
River of origin
Conuma
Quinsam
Rearing env.
Hatchery
Wild
B
read depth
30
20
10
0
7454000
7454200
7454400
7454600
7454800
River of origin
Conuma
Quinsam
Rearing env.
Hatchery
Wild
Position along chromosome 13

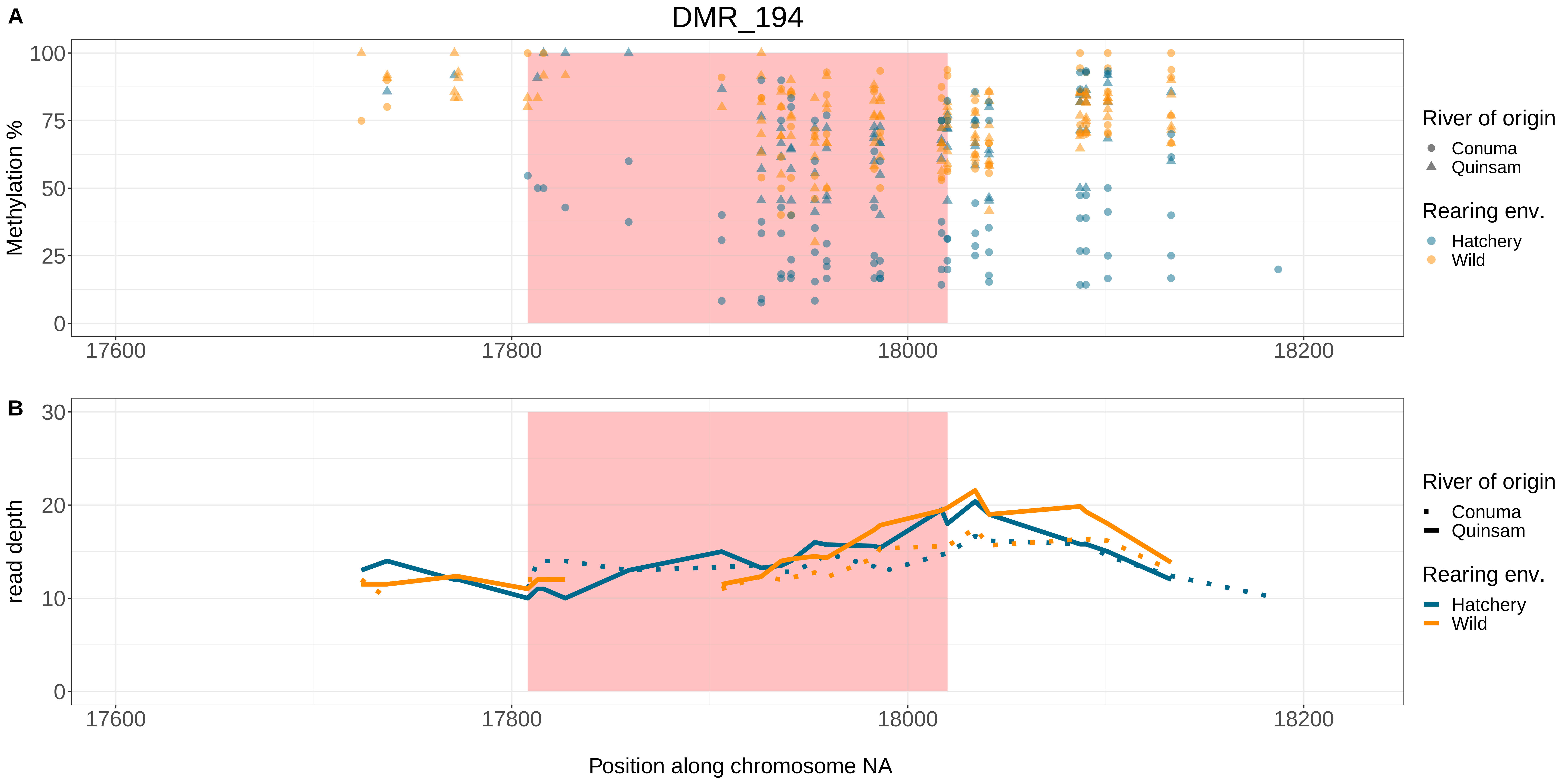
A
DMR_194
Methylation %
100
75
50
25
0
17600
17800
18000
18200
River of origin
Conuma
Quinsam
Rearing env.
Hatchery
Wild
B
read depth
30
20
10
0
17600
17800
18000
18200
River of origin
Conuma
Quinsam
Rearing env.
Hatchery
Wild
Position along chromosome NA

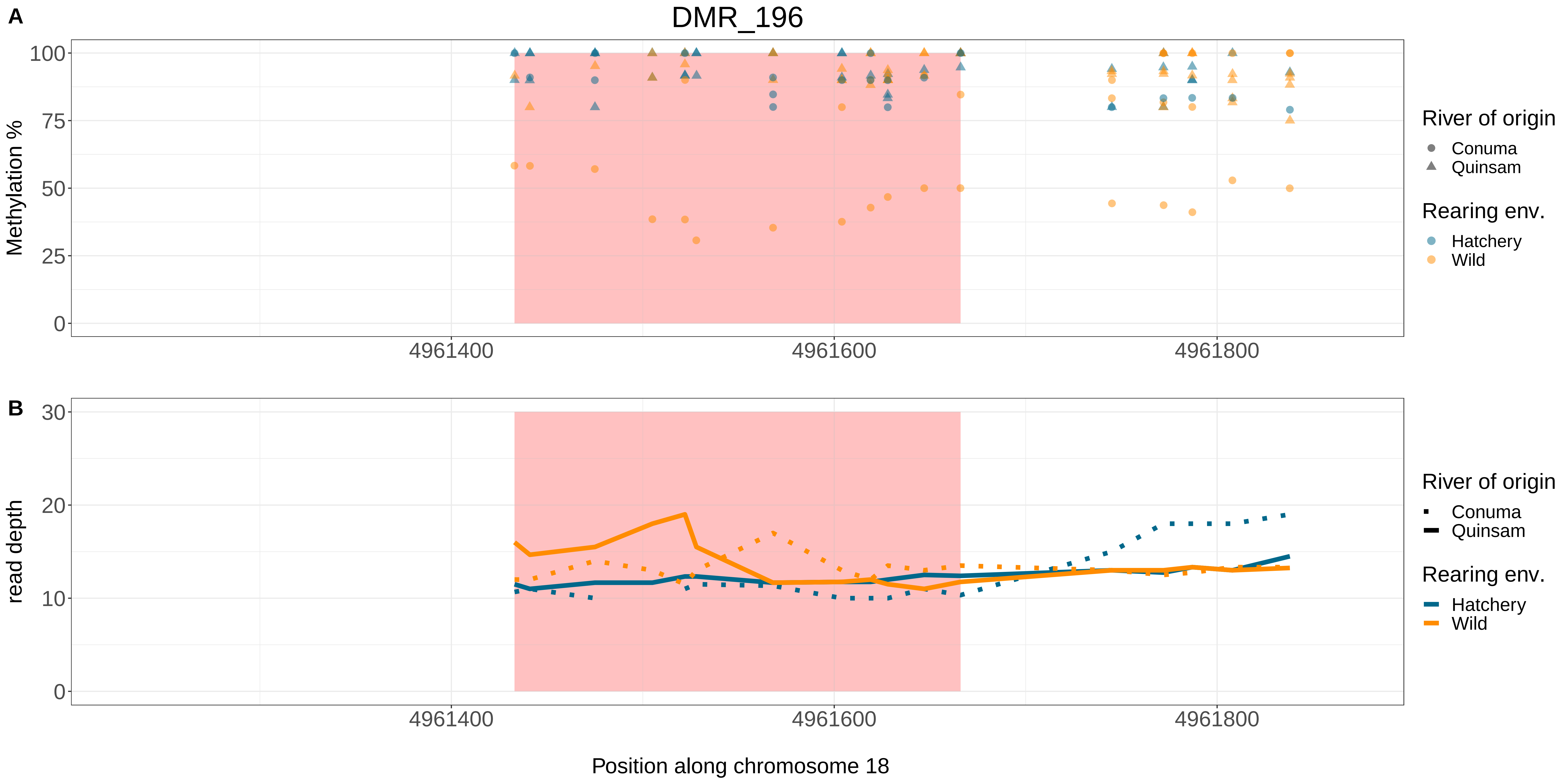

A
DMR_196
Methylation %
100
75
50
25
0
4961400
4961600
4961800
River of origin
Conuma
Quinsam
Rearing env.
Hatchery
Wild
B
read depth
30
20
10
0
4961400
4961600
4961800
River of origin
Conuma
Quinsam
Rearing env.
Hatchery
Wild
Position along chromosome 18

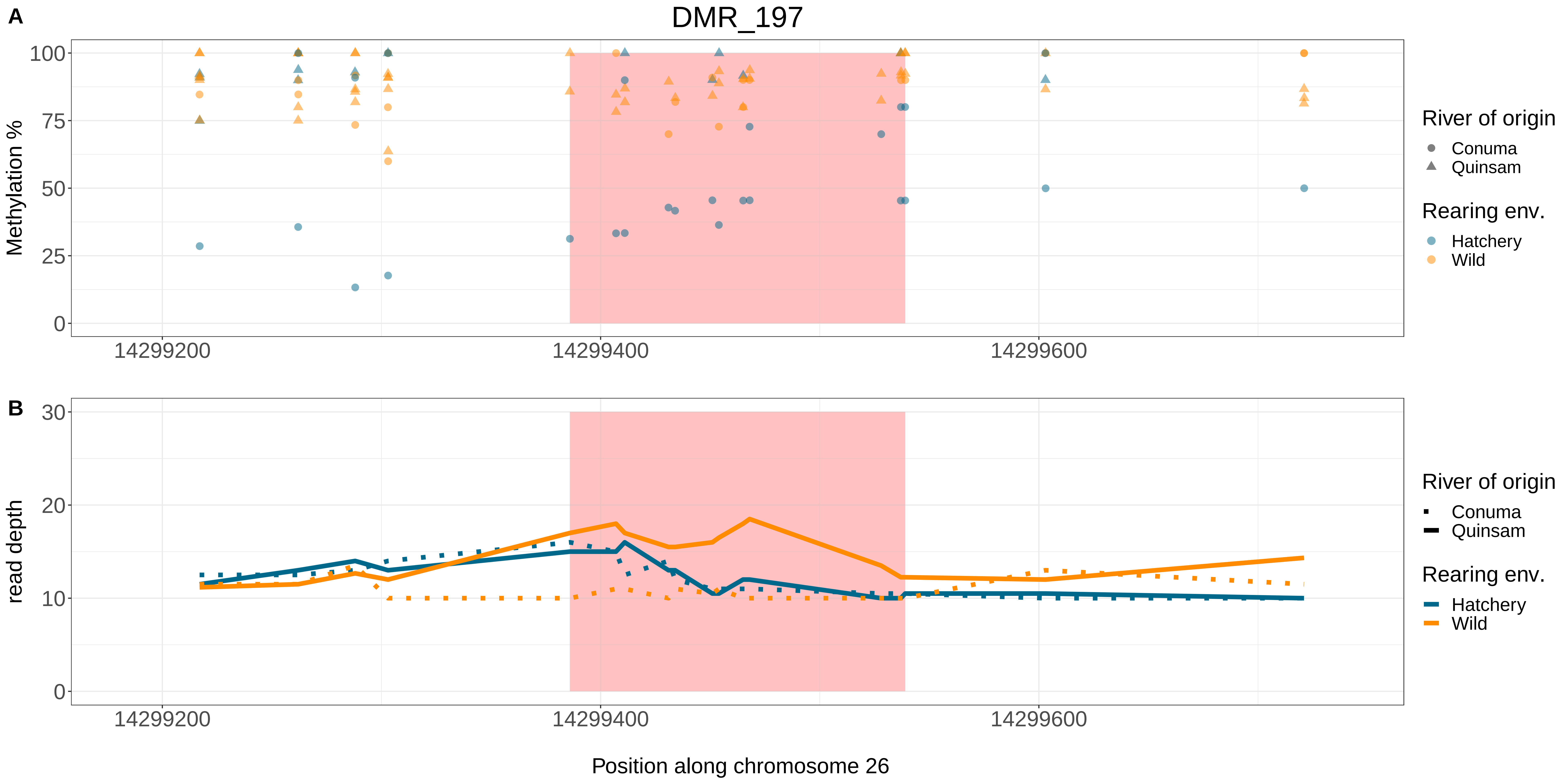
A
DMR_197
Methylation %
100
75
50
25
0
14299200
14299400
14299600
River of origin
Conuma
Quinsam
Rearing env.
Hatchery
Wild
B
read depth
30
20
10
0
14299200
14299400
14299600
River of origin
Conuma
Quinsam
Rearing env.
Hatchery
Wild
Position along chromosome 26

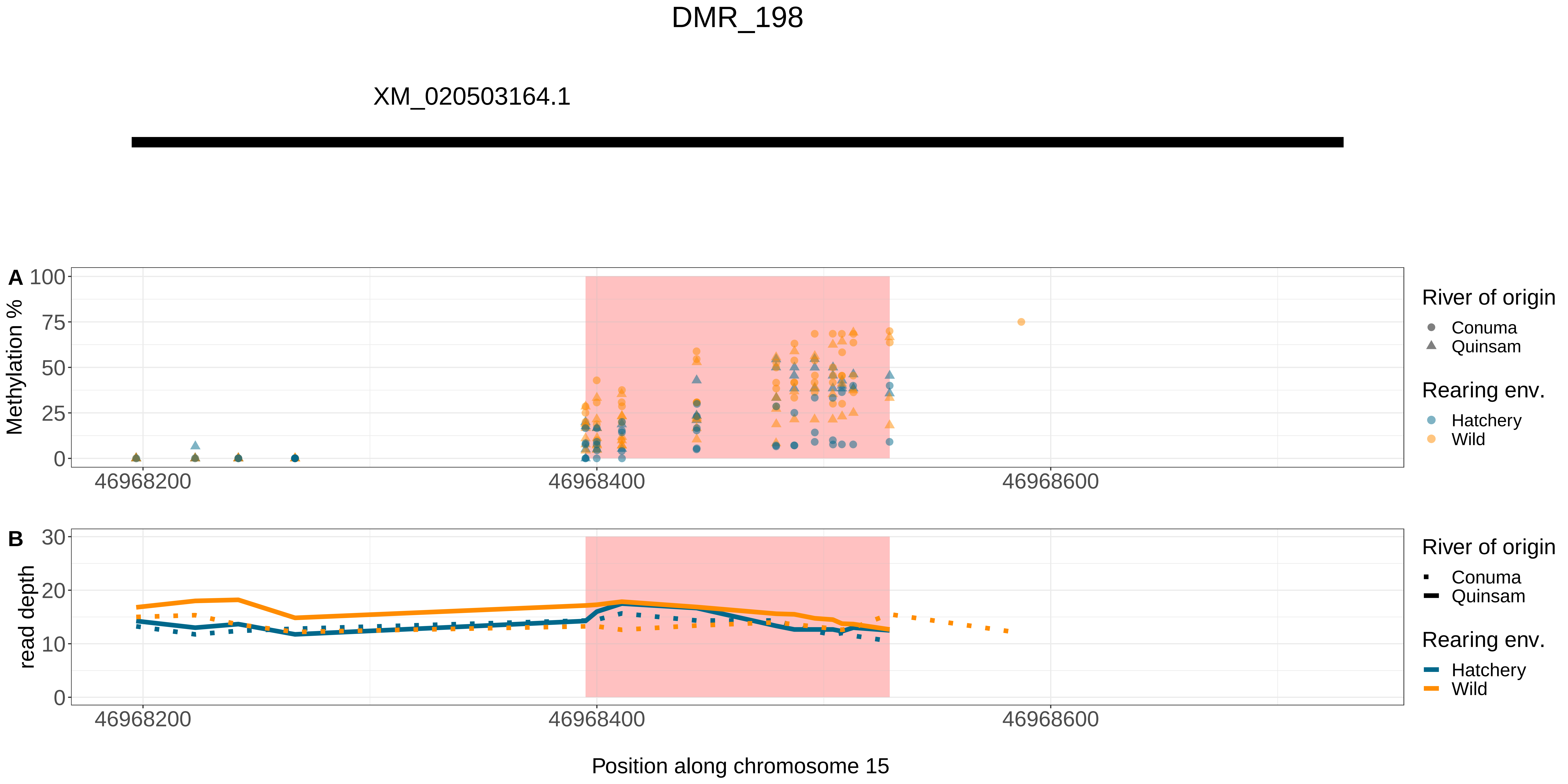
DMR_198
XM_020503164.1
A
Methylation %
100
75
50
25
0
46968200
46968400
46968600
River of origin
Conuma
Quinsam
Rearing env.
Hatchery
Wild
B
read depth
30
20
10
0
46968200
46968400
46968600
River of origin
Conuma
Quinsam
Rearing env.
Hatchery
Wild
Position along chromosome 15

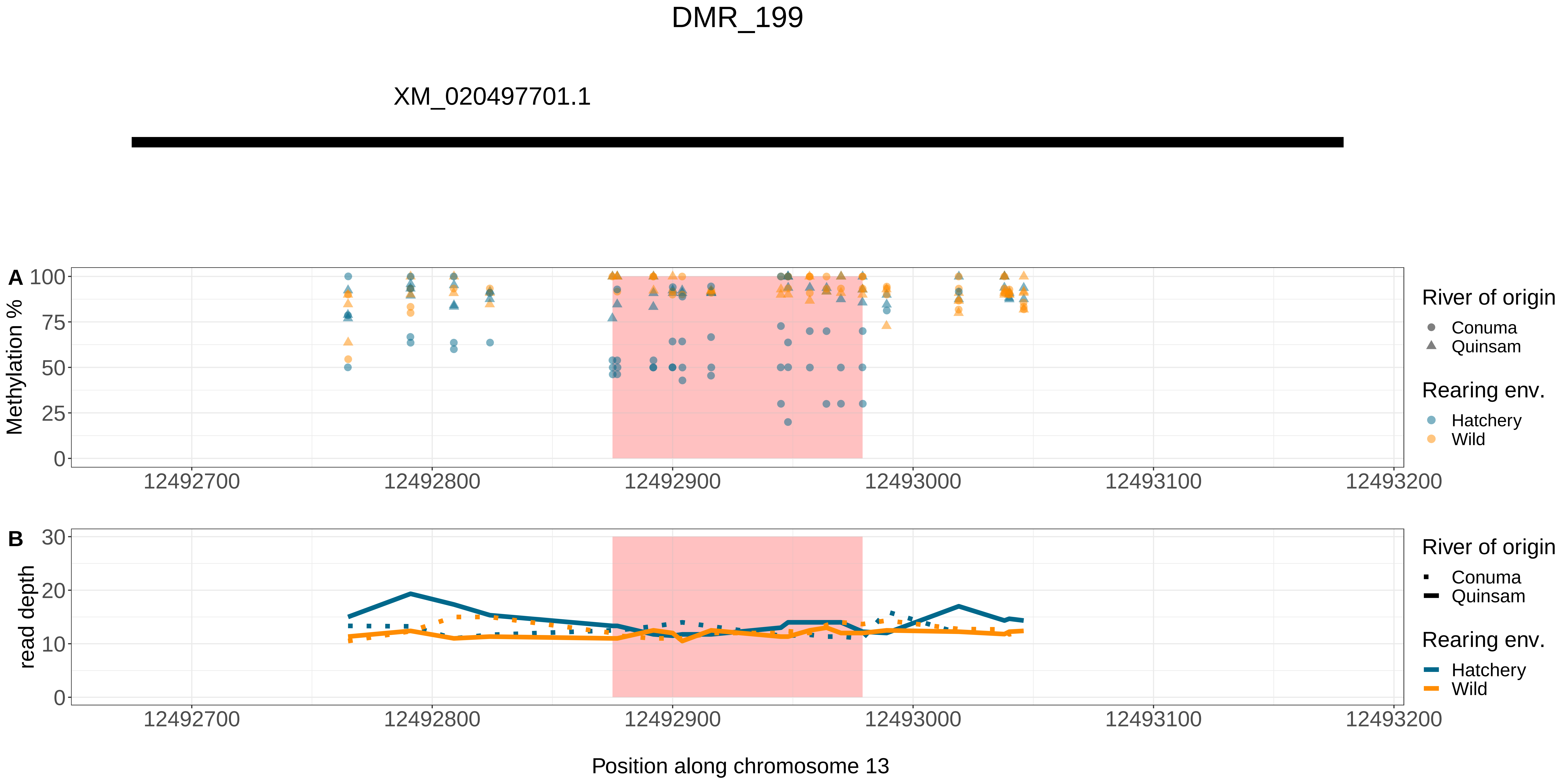
DMR_199
XM_020497701.1
A
B
Methylation %
100
75
50
25
0
read depth
30
20
10
0
12492700
12492800
12492900
12493000
12493100
12493200
Position along chromosome 13
River of origin
Conuma
Quinsam
Rearing env.
Hatchery
Wild

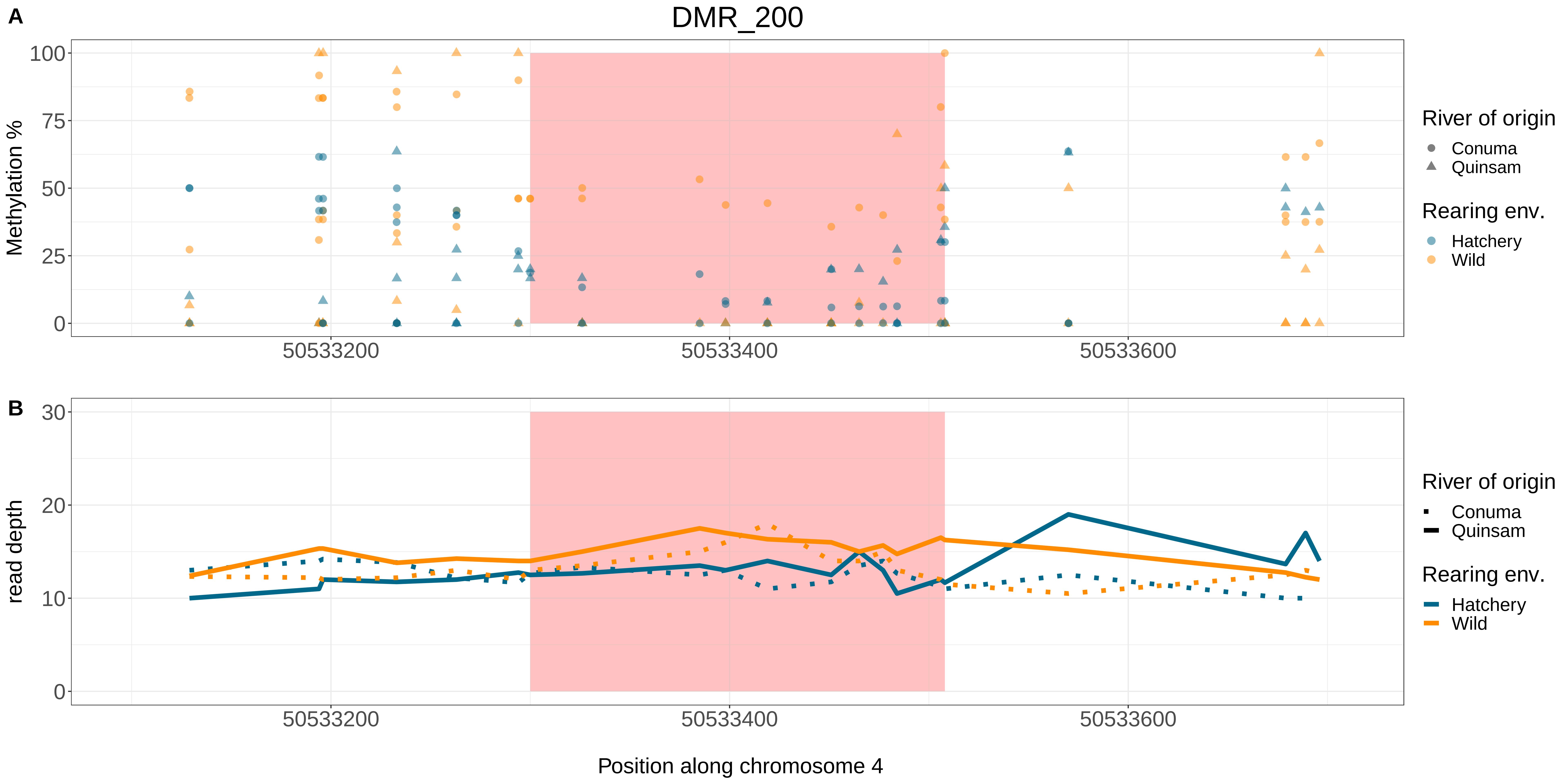

A
DMR_200
Methylation %
100
75
50
25
0
50533200
50533400
50533600
River of origin
Conuma
Quinsam
Rearing env.
Hatchery
Wild
B
read depth
30
20
10
0
50533200
50533400
50533600
Position along chromosome 4
River of origin
Conuma
Quinsam
Rearing env.
Hatchery
Wild

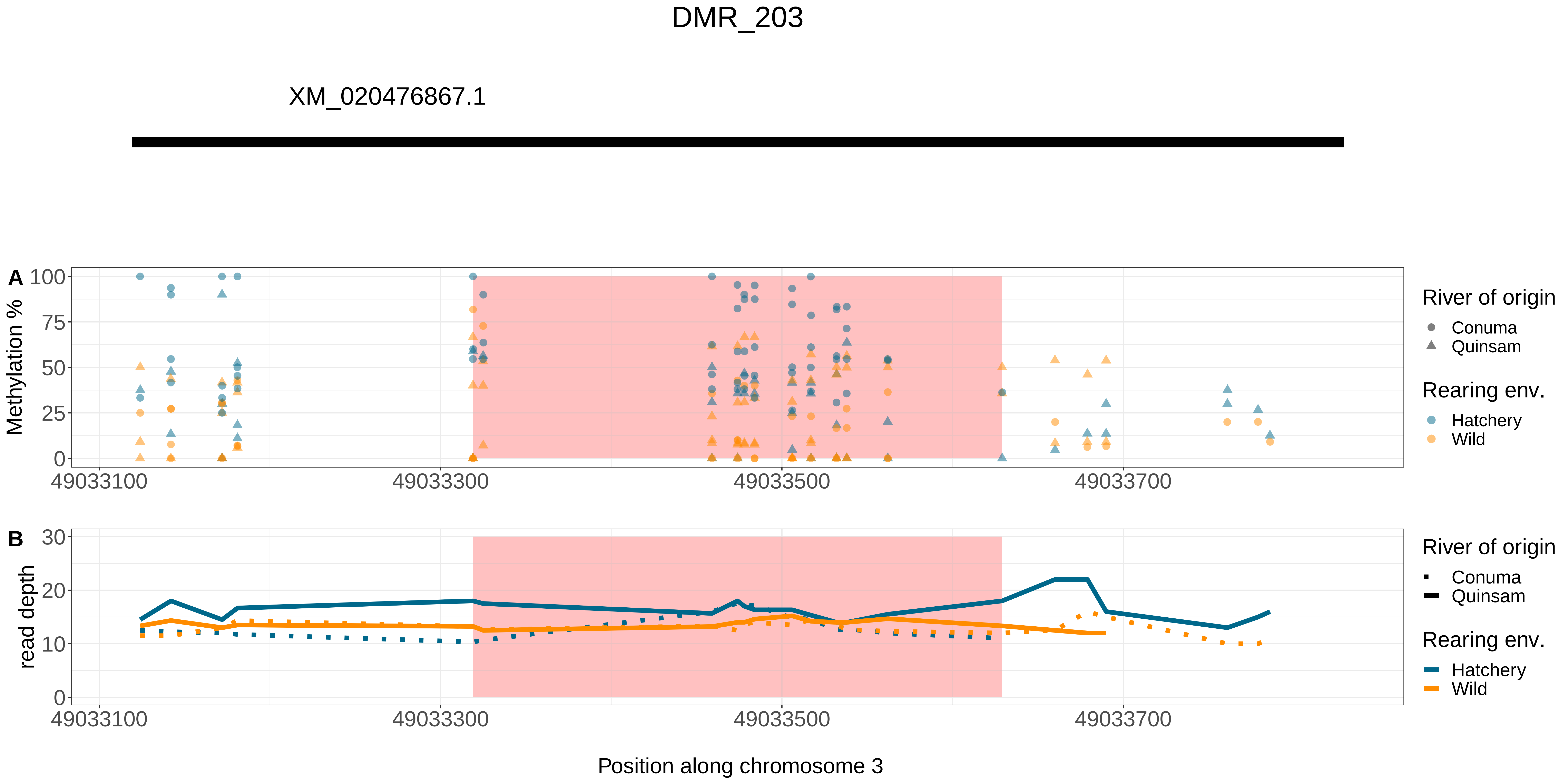

DMR_203
XM_020476867.1
A
Methylation %
100
75
50
25
0
49033100
49033300
49033500
49033700
River of origin
Conuma
Quinsam
Rearing env.
Hatchery
Wild
B
read depth
30
20
10
0
49033100
49033300
49033500
49033700
River of origin
Conuma
Quinsam
Rearing env.
Hatchery
Wild
Position along chromosome 3

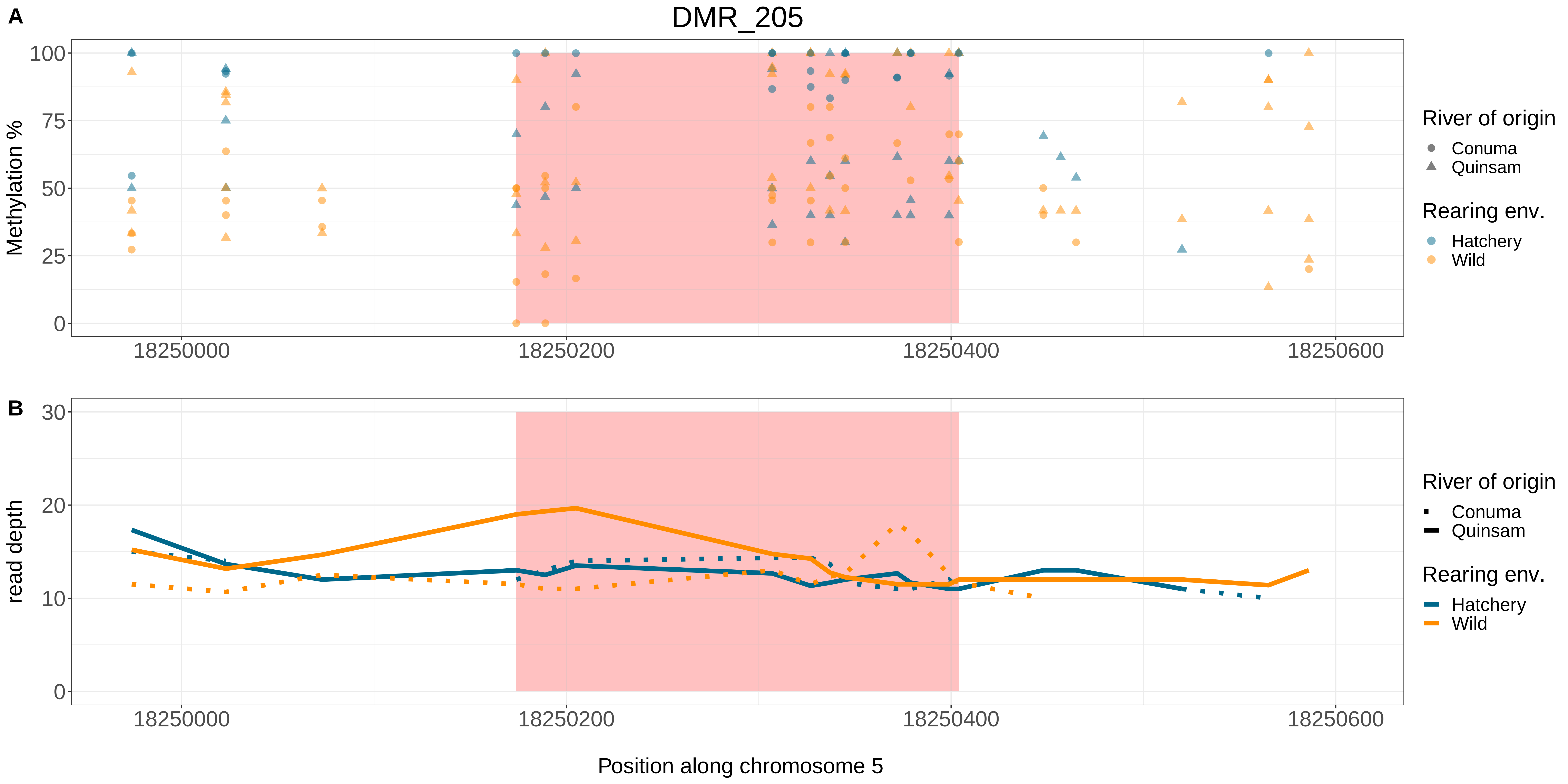
A
DMR_205
Methylation %
100
75
50
25
0
18250000
18250200
18250400
18250600
River of origin
Conuma
Quinsam
Rearing env.
Hatchery
Wild
B
read depth
30
20
10
0
18250000
18250200
18250400
18250600
River of origin
Conuma
Quinsam
Rearing env.
Hatchery
Wild
Position along chromosome 5

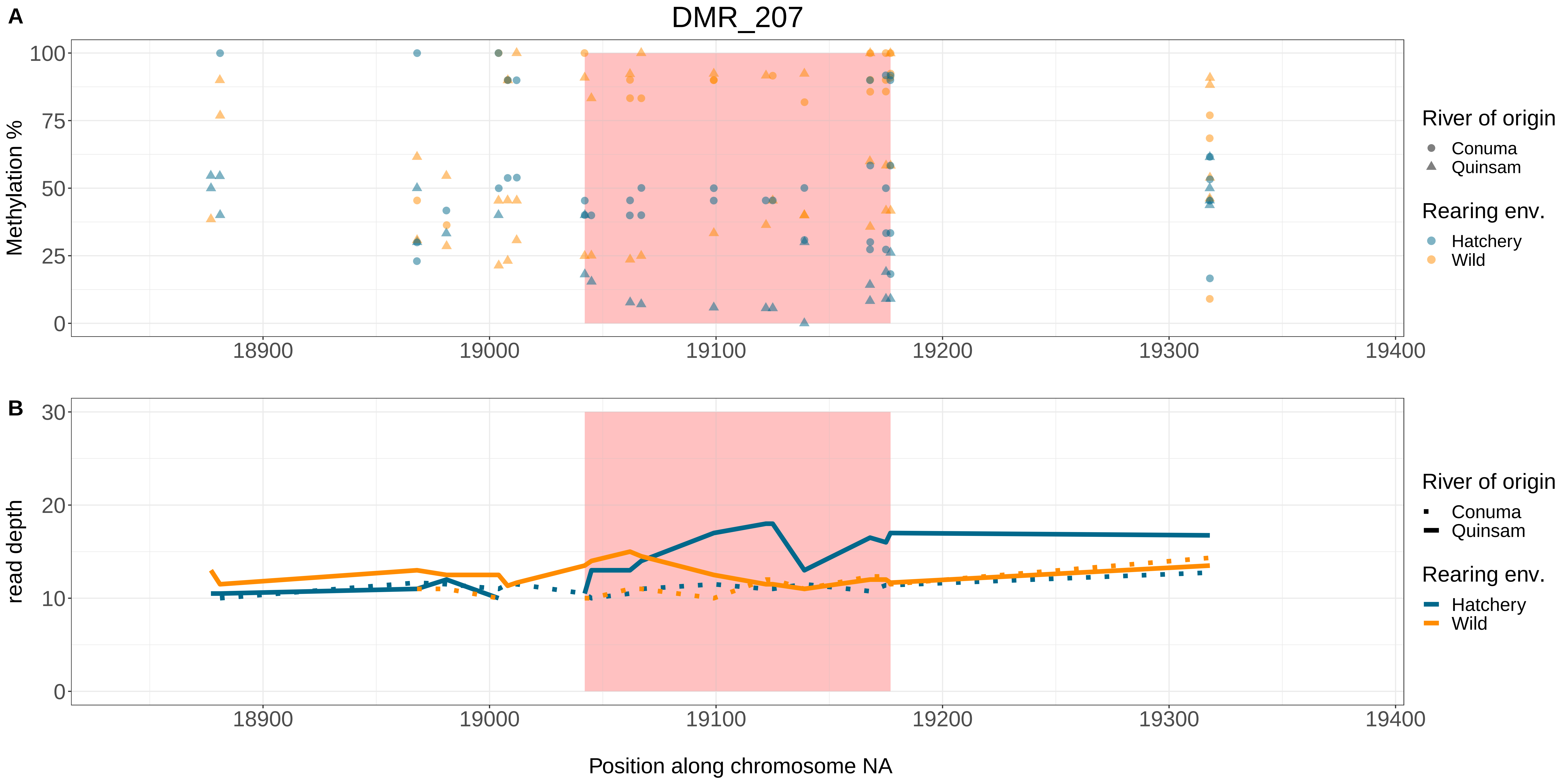
A
DMR_207
Methylation %
100
75
50
25
0
18900
19000
19100
19200
19300
19400
River of origin
Conuma
Quinsam
Rearing env.
Hatchery
Wild
B
read depth
30
20
10
0
Position along chromosome NA

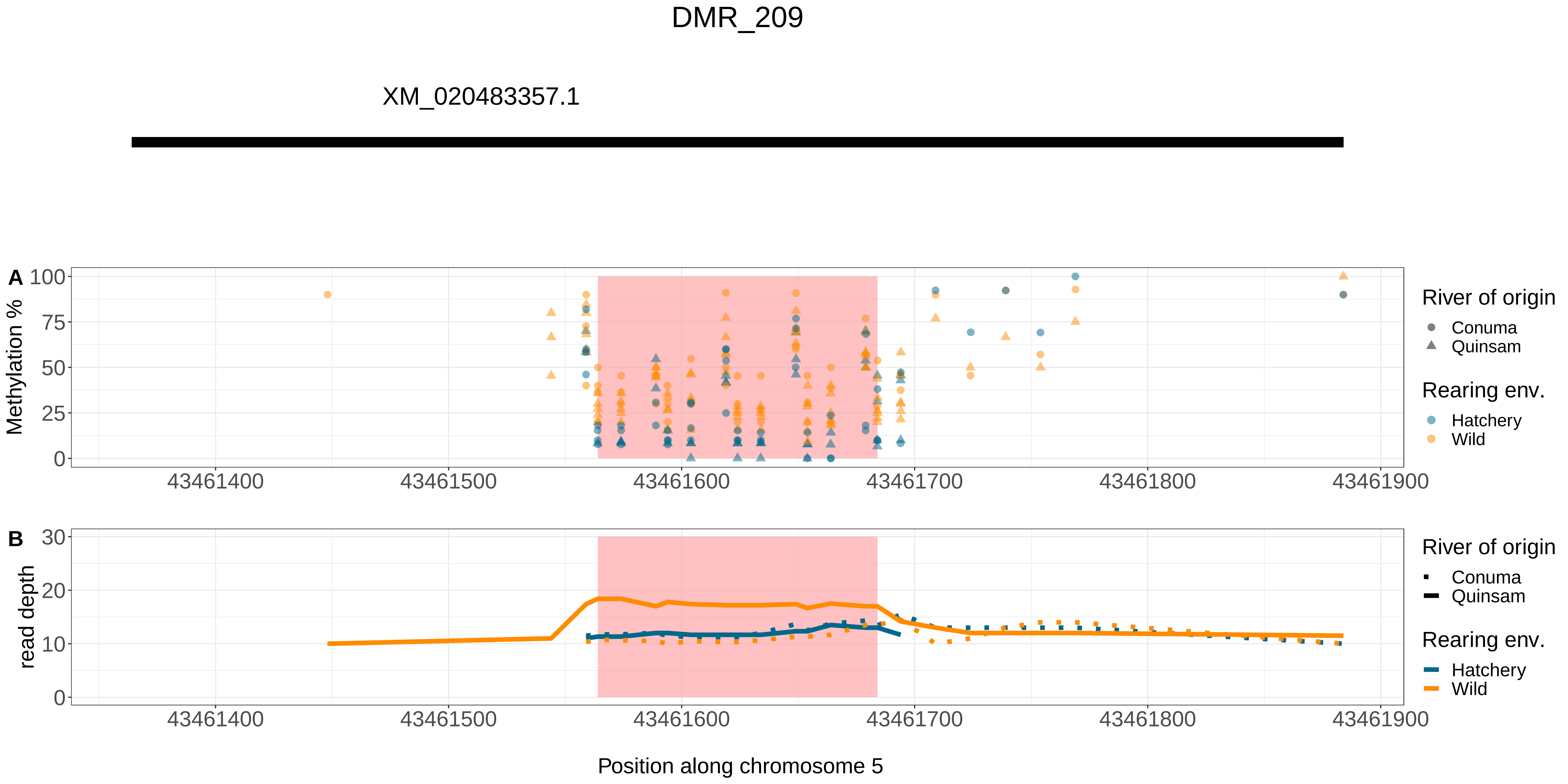
DMR_209
XM_020483357.1
A
B
Methylation %
100
75
50
25
0
read depth
30
20
10
0
43461400
43461500
43461600
43461700
43461800
43461900
River of origin
Conuma
Quinsam
Rearing env.
Hatchery
Wild
Position along chromosome 5

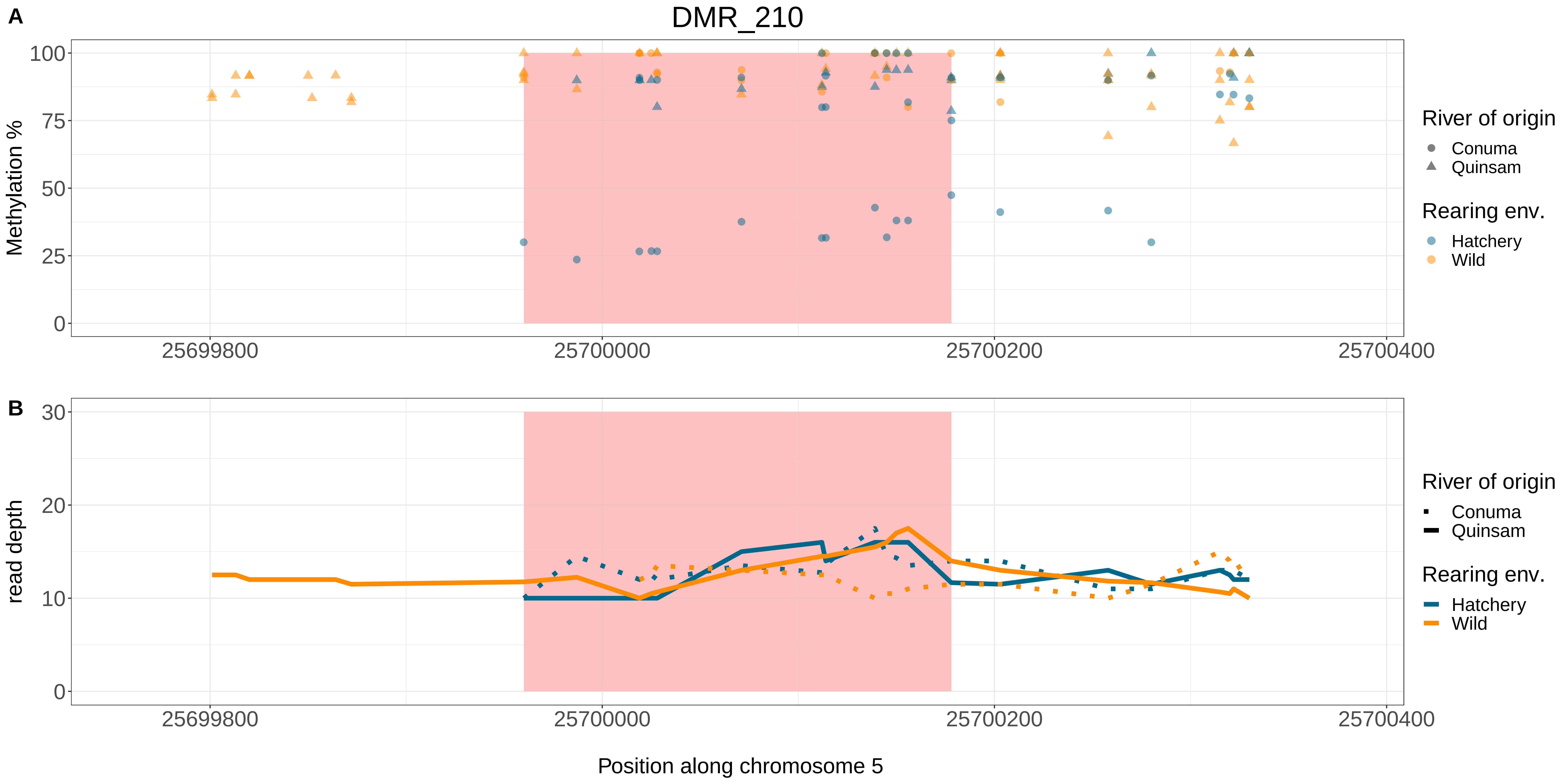

A
DMR_210
Methylation %
100
75
50
25
0
25699800
25700000
25700200
25700400
River of origin
Conuma
Quinsam
Rearing env.
Hatchery
Wild
B
read depth
30
20
10
0
25699800
25700000
25700200
25700400
River of origin
Conuma
Quinsam
Rearing env.
Hatchery
Wild
Position along chromosome 5

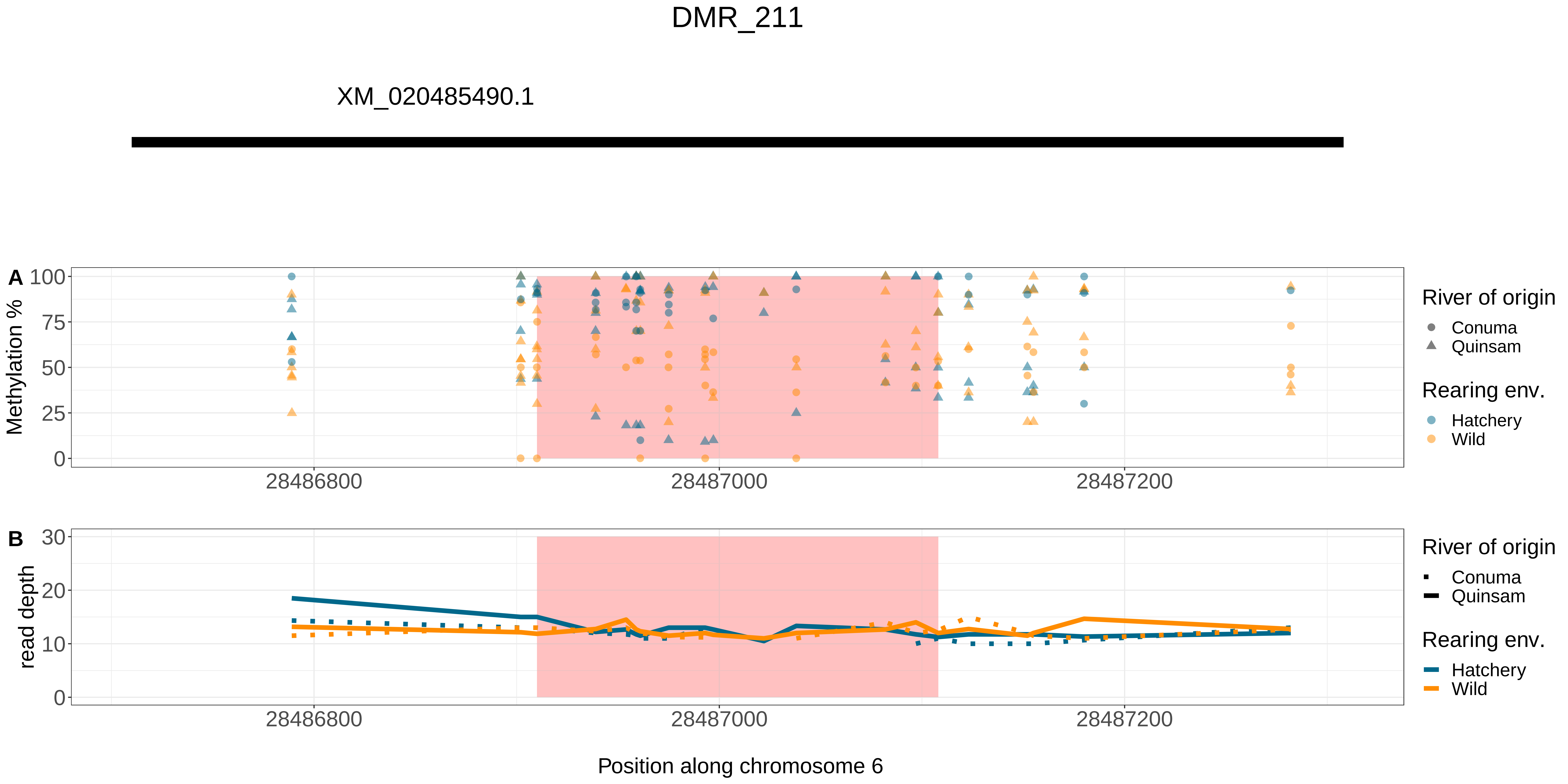
DMR_211
XM_020485490.1
A
Methylation %
100
75
50
25
0
28486800
28487000
28487200
River of origin
Conuma
Quinsam
Rearing env.
Hatchery
Wild
B
read depth
30
20
10
0
28486800
28487000
28487200
River of origin
Conuma
Quinsam
Rearing env.
Hatchery
Wild
Position along chromosome 6
